# Supplementary material for: Nickel-catalyzed switchable 1,3-dienylation and enantioselective allenylation of phosphine oxides
Source: Nat Commun. 2022 Nov 17;13:7049. doi: 10.1038/s41467-022-34764-x (PMC9671958; doi:10.1038/s41467-022-34764-x)
Supplement: Supplementary file 1 — Supplementary Information [file 41467_2022_34764_MOESM1_ESM.pdf]

Supplementary Information

Nickel-Catalyzed Switchable 1,3-Dienylation and Enantioselective Allenylation of Phosphine Oxides

Jiayin Zhang, Xihao Chang, Xianghong Xu, Hongyi Wang, Lingzi Peng, Chang Guo*

Hefei National Laboratory for Physical Sciences at the Microscale, University of Science and Technology of China, Hefei, 230026, China

*e-mail: guochang@ustc.edu.cn

CONTENTS:

1. Supplementary Notes 2
2. Supplementary Discussion 6
3. Supplementary Methods 16
4. Supplementary References...185

1. Supplementary Notes

General information:

Unless otherwise noted, all reagents were purchased from commercial suppliers and used without further purification. All reactions were carried out in flame-dried glassware under a dry nitrogen atmosphere. NMR spectra were recorded on Bruker-400, 500, or 600 MHz spectrometers. Chemical shifts (δ) are given in ppm relative to TMS. The residual solvent signals were used as references and the chemical shifts converted to the TMS scale ($CHCl_3$: δ 7.26 for proton and δ 77.16 for carbon; Acetone: δ 2.49 for proton and δ 39.51 for carbon). Multiplicities were given as: s (singlet); d (doublet); t (triplet); q (quartet); dd (doublet of doublets); dt (doublet of triplets); m (multiplets); brs (broad signal). Coupling constants are reported as a *J* value in Hz. High-resolution mass spectral analysis (HRMS) was performed on Waters XEVO G2 Q-TOF. The measurement of enantiomeric excesses (e.e.) was performed on Waters-Alliance (2998. Photodiode Array Detector, UV detection monitored at 270, 254, 241, or 220 nm). Chiralpak AD-H, IC, IE, OD-H, and IG columns were purchased from Daicel Chemical Industries, LTD. Racemic samples were prepared using 1,3-Bis(diphenylphosphino)-propane (DPPP). The absolute configuration of ligands **L23**, **4b**, and **5** was assigned by the X-ray analysis (CCDC 2106890, 2131762, and 2120142). Optical rotations were determined at 589 nm (sodium D line) using a Perkin-Elmer-343 polarimeter (1 dm path length cell).

Synthesis of BDPP-type ligands

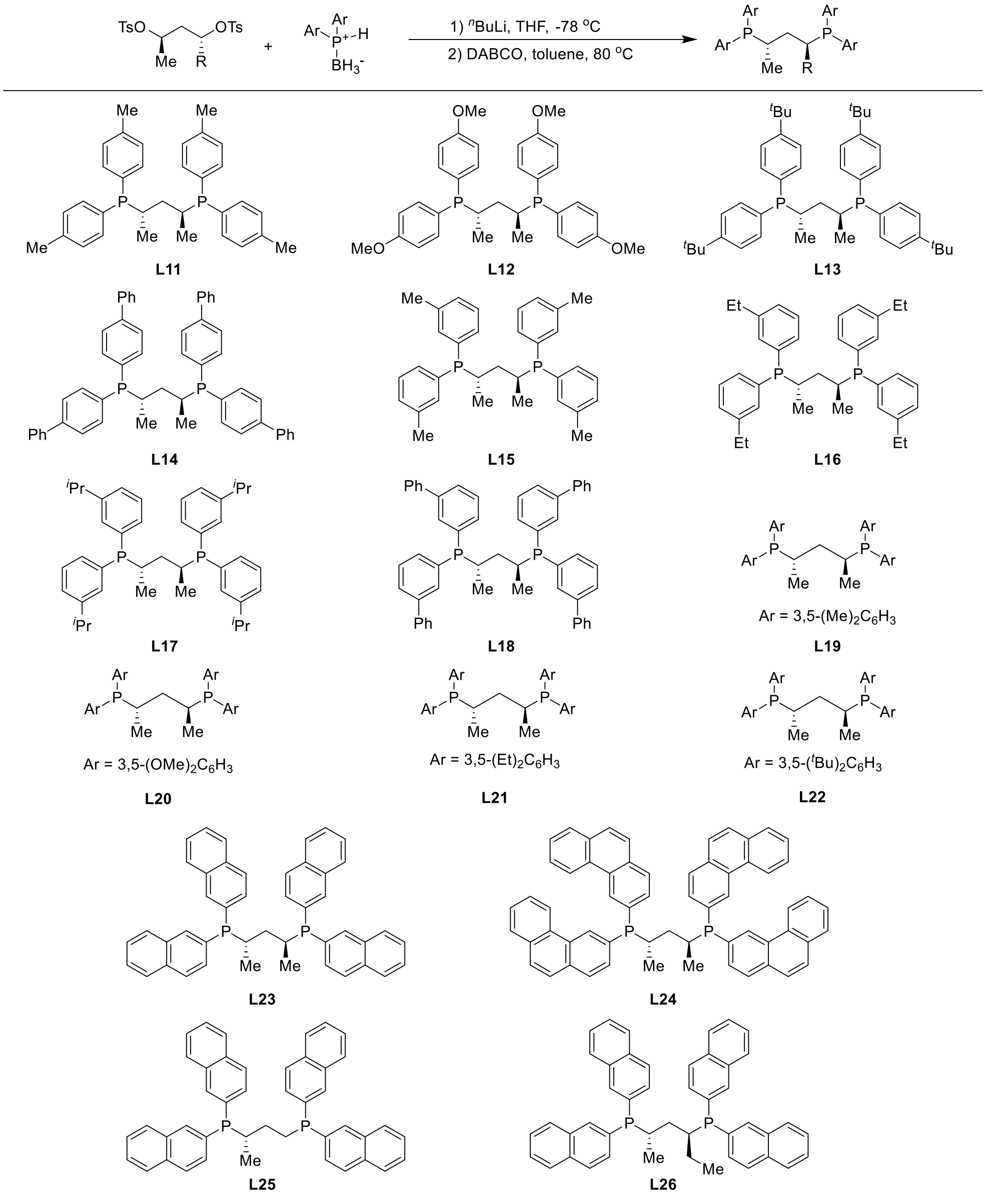

Supplementary Figure 1. Synthesis of BDPP-type ligands (**L11-L26**)

A specific example for the synthesis of ligand **L23**:

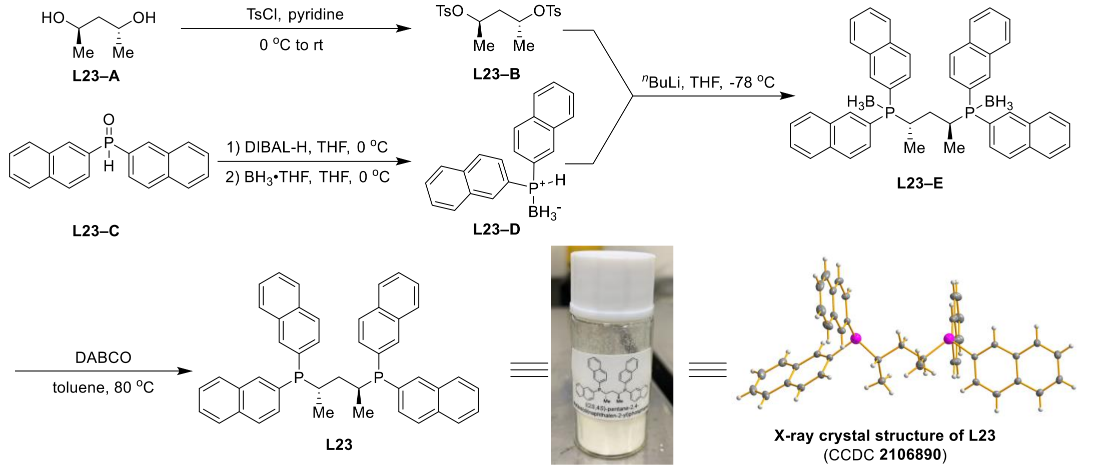

L23-B was prepared according to a modified literature procedure.[1]

(*R*,*R*)-2,4-pentanediol (**L23-A**, 3.12 g, 30.0 mmol) and tosyl chloride (22.8 g, 120.0 mmol) were charged to a round-bottom flask under a nitrogen atmosphere. Anhydrous pyridine (30.0 mL) was added, and the resulting solution was stirred at room temperature for 48 h. Ice cold water and DCM were slowly added, and the layers were separated. The organic phases were washed with 1 N HCl (30 mL), saturated solution of $NaHCO_3$ (60 mL), dried over anhydrous Na_2SO_4, and filtered. The crude residue was concentrated and purified by silica gel column chromatography to afford a white solid (**L23-B**, 7.93 g, 64%).

L23-D was prepared according to a modified literature procedure.[2]

A solution of phosphine oxide **L23-C** (6.04 g, 20.0 mmol) in THF (40.0 mL) was added dropwise slowly to a solution of DIBAL-H (1 M in hexane, 60.0 mL, 60.0 mmol) under a nitrogen atmosphere in a round-bottom flask. The resulting solution was stirred for 3 h at room temperature, then Borane-tetrahydrofuran (1 M solution in THF, 40.0 mL, 40.0 mmol) was added to the vessel, and the solution was allowed to stir under nitrogen for 8 h. A solution of 2 N NaOH (20 mL) was slowly added to the reaction mixture, followed by a saturated solution of Rochelle's salt (20 mL). The reaction was diluted with brine and extracted with EtOAc. The organic layer was separated, dried over anhydrous Na_2SO_4, and filtered. The crude residue was concentrated and purified by silica gel column chromatography to yield **L23-D** (4.18 g, 70%).

L23 was prepared according to a modified literature procedure.[1]

A vigorously stirred solution of phosphine-borane adduct **L23-D** (1.8 g, 6.0 mmol) in 30 mL THF was placed at -78 °C under a nitrogen atmosphere. ^{n}BuLi (2.5 M in hexanes, 2.5 mL) was added to the vessel, and the solution was allowed to stir for 30 min. A solution of 1,3-ditosylatepropane **L23-B** (1.2 g, 2.91 mmol) in THF (4.5 mL) was subsequently added dropwise to the reaction flask. After stirring at -78 °C for 30 min, the solution was allowed to warm to 0 °C over 30 min and kept at room temperature for another 24 h. The reaction mixture was diluted with 10% HCl (20 mL) and extracted with EtOAc (60 mL). The organic layer was separated, dried over anhydrous Na_2SO_4, and filtered. The crude residue was concentrated and purified by silica gel column chromatography to yield **L23-E** (1.27g, 63%).

The purified adduct **L23-E** (1.27g, 1.9 mmol) and DABCO (695 mg, 3.0 equiv) was added to a flame-dried Schlenk tube. The vessel was flushed with Ar (g) and toluene (5 mL) was subsequently added. The vessel was sealed and stirred at 80 °C for 5 hours, after which the toluene was removed in vacuo, and the crude material purified by silica gel column chromatography to yield **L23** (1.04 g, 86%). **^{1}H NMR (500 MHz, $CDCl_3$)** δ 8.04 – 7.93 (m, 4H), 7.84 – 7.65 (m, 10H), 7.62 – 7.53 (m,

2H), 7.51 – 7.32 (m, 12H), 2.91 – 2.64 (m, 2H), 1.69 – 1.52 (m, 2H), 1.19 – 1.08 (m, 6H). **^{13}C NMR (125 MHz, $CDCl_3$)** δ 134.62, 134.60 (d, *J* = 15.1 Hz), 134.44 (d, *J* = 6.5 Hz), 134.27, 134.22 (d, *J* = 14.1 Hz), 133.48, 133.24 (d, *J* = 9.2 Hz), 129.76 (d, *J* = 14.0 Hz), 129.63 (d, *J* = 13.5 Hz), 128.14, 127.93, 127.90, 127.88, 127.86, 127.80, 127.77, 126.71, 126.35 , 37.00 (t, *J* = 18.6 Hz), 27.35 (t, *J* = 11.2 Hz), 16.13 (d, *J* = 16.1 Hz). **^{31}P NMR (202 MHz, $CDCl_3$)** δ 0.68. **APCI-MS:** calculated [$C_{45}H_{38}P_2$ + H]$^+$: 641.2522, found: 641.2528. $[\alpha]^{20}_D$ = -124.3 (c = 0.73, CH_2Cl_2).

((2*S*,4*S*)-pentane-2,4-diyl)bis(bis(3,5-di-tert-butylphenyl)phosphane) (**L22**)

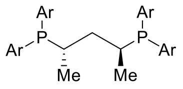

Ar = 3,5-(^{t}Bu)$_2$C$_6$H$_3$

^{1}H NMR (500 MHz, $CDCl_3$) δ 7.40 – 7.34 (m, 6H), 7.32 – 7.29 (m, 2H), 7.27 – 7.23 (m, 4H), 2.61 – 2.44 (m, 2H), 1.47 – 1.39 (m, 2H), 1.31 – 1.19 (m, 72H), 1.02 (dd, *J* = 15.4, 6.7 Hz, 6H). **^{31}P NMR (202 MHz, $CDCl_3$)** δ 3.19. **^{13}C NMR (125 MHz, $CDCl_3$)** δ 150.26 (d, *J* = 7.3 Hz), 150.11 (d, *J* = 6.6 Hz), 136.67, 134.68, 128.35 (d, *J* = 20.4 Hz), 127.65 (d, *J* = 19.1 Hz), 123.22, 122.53, 36.43 (t, *J* = 17.4 Hz), 34.97, 31.62, 31.57, 27.62 (t, *J* = 10.7 Hz), 15.81 (d, *J* = 17.3 Hz). **ESI-MS:** calculated [$C_{61}H_{91}P_2$ + H]$^+$: 889.6904, found: 889.6899. $[\alpha]^{20}_D$ = -0.5 (c = 1.01, CH_2Cl_2).

2. Supplementary Discussion

Supplementary Table 1. Optimization for the 1,3-dienylation.

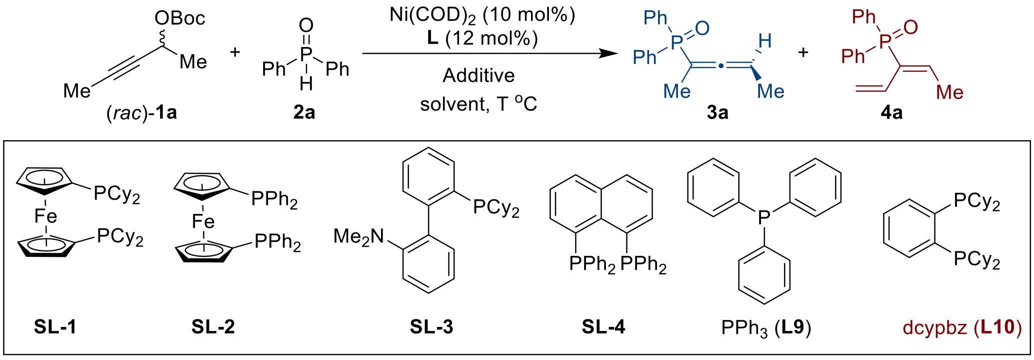

Entry	L	solvent	Additive (0.5 equiv)	T (°C)	**3a/4a**	yield (%)
1	**SL-1**	dioxane	-	80	-	NR
2	**SL-2**	dioxane	-	80	4:1	32
3	**SL-3**	dioxane	-	80	-	NR
4	**SL-4**	dioxane	-	80	-	NR
5	**L9**	dioxane	-	80	3:1	31
6	**L10**	dioxane	-	80	1:3	42
7	**L10**	DMF	$Ph_2P(O)OH$	100	<1:20	78

Reactions were conducted by using $Ni(COD)_2$ (10 mol%), **L** (12 mol%), (*rac*)-**1a** (0.3 mmol), and **2a** (0.1 mmol) for 24 h. Isolated yield of the mixture **3a** and **4a** after chromatography are shown.

Mechanistic studies

Kinetic studies

Synthesis of (*S*)-**1p** and (*R*)-**1p**

(*S*)-**S1** and (*R*)-**S1** was prepared from a modified literature procedure.[3]

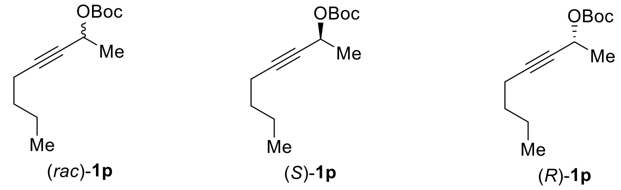

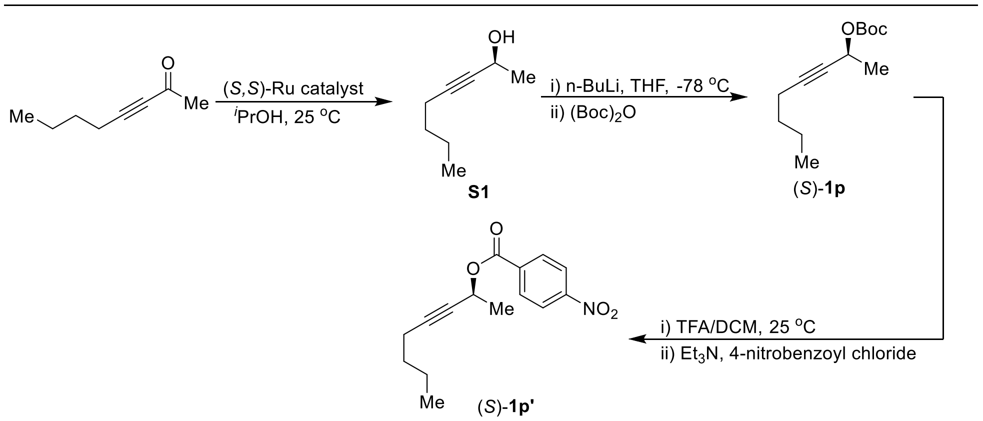

(*S*)-oct-3-yn-2-ol (S1):[3] To a solution of commercially available compound RuCl[(*S*,*S*)-$NTsCH(C_6H_5)CH(C_6H_5)NH_2$]($\eta^6$-mesitylene) (15.9 mg, 0.025 mmol) in *i*-PrOH (2 mL) was added

KOH power (1.7 mg, 0.030 mmol) at room temperature. The mixture was added to a solution of oct-3-yn-2-one (0.62 g, 5.0 mmol) in isopropanol (50 mL) via cannula after stirring for 30 min. The reaction mixture was stirred at room temperature for 12 h. The reaction mixture was concentrated, and the residue was purified by flash column chromatography (PE/EA = 20:1) on silica gel to give the desired product (436 mg, 69%).

(*S*)-tert-butyl oct-3-yn-2-yl carbonate ((*S*)-1p):[4] To a solution of oct-3-yn-2-ol (11.0 mmol) in THF (30.0 mL) was added n-BuLi (2.5 M solution in hexanes, 4.0 mL, 10.0 mmol) at -78℃ and the resulting solution was stirred at the same temperature for 20 min. To this solution was added the solution of BOC anhydride (1.96 g, 9.0 mmol) in THF (8.0 mL) and the reaction was stirred for 2h at room temperature. Aqueous NH_4Cl was added to quench the reaction. The resulting mixture was extracted with EtOAc (3 × 20 mL). The combined organic layers were washed with water (40 mL) and brine (40 mL) successively, dried (anhydrous $MgSO_4$), and concentrated. The residue was purified through silica gel flash column chromatography (hexanes/ethyl acetate = 80/1) to yield the product in 85% yield. **^{1}H NMR (600 MHz, $CDCl_3$)** δ 5.24 (qt, *J* = 6.6, 2.0 Hz, 1H), 2.20 (td, *J* = 7.0, 1.9 Hz, 2H), 1.52 – 1.45 (m, 14H), 1.43 – 1.35 (m, 2H), 0.90 (t, *J* = 7.3 Hz, 3H). **^{13}C NMR (150 MHz, CDCl3)** δ 152.71, 86.15, 82.48, 78.39, 63.88, 30.58, 27.88, 21.96, 21.94, 18.47, 13.71.

(*S*)-**1p**: 93% e.e., The e.e. values of (*S*)-**1p** were determined by chiral HPLC analysis of *p*-nitrobenzoate derivatives (*S*)-**1p′**.

(*S*)-oct-3-yn-2-yl 4-nitrobenzoate ((*S*)-1p′): To a solution of (*S*)-**1p** (0.2 mmol) in DCM (1.0 mL) was added 1.0 mL trifluoroacetic acid at room temperture and the resulting solution was stirred at the same temperature for 4 min. The resulting mixture was concentrated *in vacuo* to remove the solvent. DCM (1.0 mL), Et_3N (1.2 mmol) and 4-nitrobenzoyl chloride (0.3 mmol) was added subsequently. The resulting mixture was stirred at room temperature for 20 min and purified through silica gel flash column chromatography to yield the product in 70% yield. **^{1}H NMR (500 MHz, $CDCl_3$)** δ 8.32 – 8.27 (m, 2H), 8.26 – 8.22 (m, 2H), 5.71 (qt, *J* = 6.6, 1.9 Hz, 1H), 2.23 (td, *J* = 7.1, 2.0 Hz, 2H), 1.62 (d, *J* = 6.6 Hz, 3H), 1.54 – 1.46 (m, 2H), 1.44 – 1.36 (m, 2H), 0.91 (t, *J* = 7.3 Hz, 3H). **^{13}C NMR (125 MHz, $CDCl_3$)** δ 163.84, 150.67, 135.73, 130.99, 123.62, 86.65, 78.09, 62.79, 30.59, 22.04, 21.99, 18.49, 13.70.

The product of (*S*)-**1p′** was analyzed by HPLC to determine the enantiomer.ric excess: 93% e.e. (CHIRALPAK IC, hexane/*i*-PrOH =95/5, detector: 254 nm, T = 25 °C, flow rate: 1 mL/min), t_1(major) = 7.62 min, t_2(minor) = 8.10 min.

(*R*)-**1p**: 96% e.e., Spectral data were in agreement with (*S*)-**1p** reported above, the e.e. values of (*R*)-**1p** were determined by chiral HPLC analysis of *p*-nitrobenzoate derivatives (*R*)-**1p′**.

(*R*)-**1p′**: Spectral data were in agreement with (*S*)-**1p′** reported above. The product of (*R*)-**1p′** was analyzed by HPLC to determine the enantiomeric excess: 96% e.e. (CHIRALPAK IC, hexane/*i*-PrOH =95/5, detector: 254 nm, T = 25 °C, flow rate: 1 mL/min), t_1(minor) = 7.38 min, t_2(major) = 7.80 min.

The reaction with (*rac*)-**1p** monitored over different reaction time

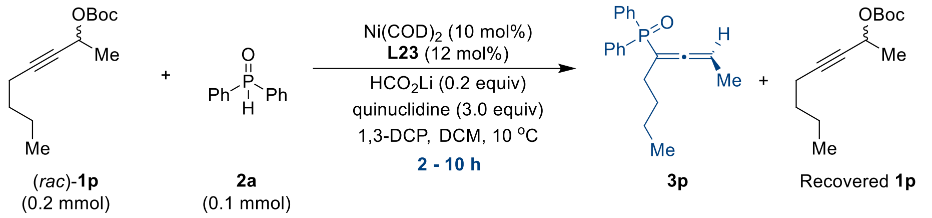

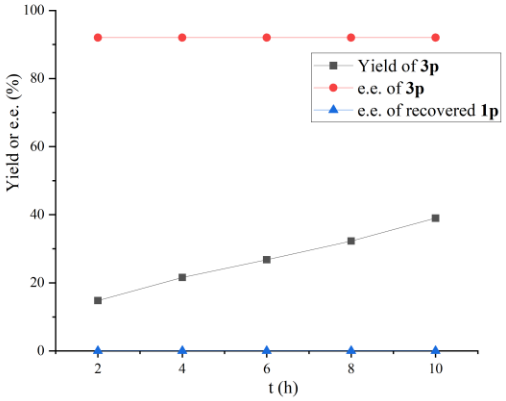

t (h)	Yield (%) of **3p**	e.e. (%) of **3p**	e.e. (%) of recovered **1p**
2	14.8	92	0
4	21.6	92	0
6	26.8	92	0
8	32.3	92	0
10	39.0	92	0

Supplementary Figure 2. The reaction with (*rac*)-**1p** monitored over different reaction times

The reaction with (*S*)-**1p** monitored over different reaction time

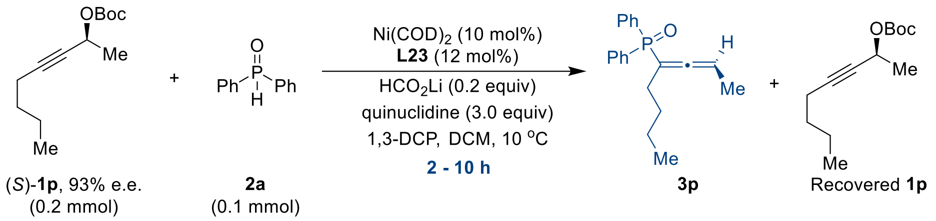

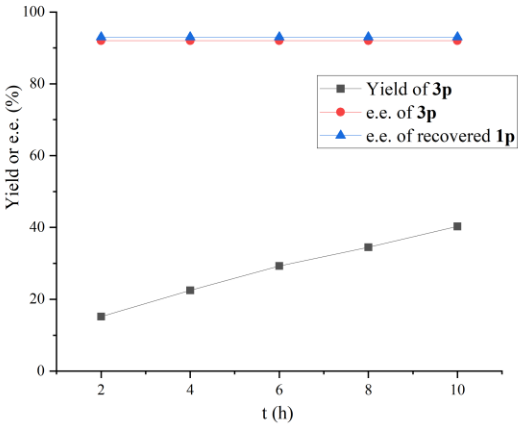

t (h)	Yield (%) of **3p**	e.e. (%) of **3p**	e.e. (%) of recovered **1p**
2	15.2	92	93
4	22.5	92	93
6	29.3	92	93
8	34.5	92	93
10	40.3	92	93

Supplementary Figure 3. The reaction with (*S*)-**1p** monitored over different reaction times

The reaction with (*R*)-**1p** monitored over different reaction time

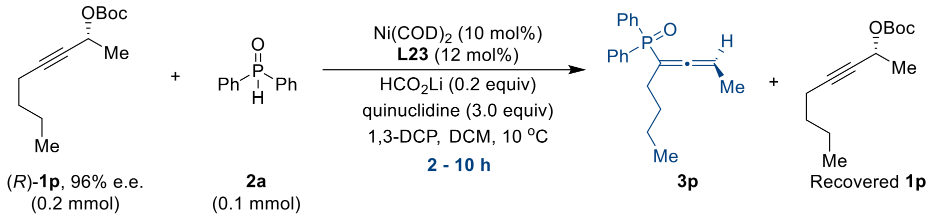

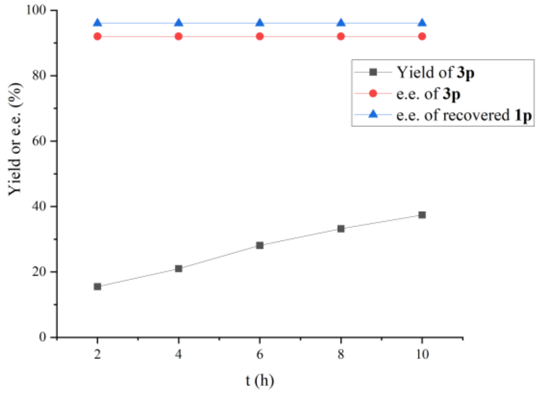

t (h)	Yield (%) of **3p**	e.e. (%) of **3p**	e.e. (%) of recovered **1p**
2	15.5	92	96
4	21.0	92	96
6	28.1	92	96
8	33.2	92	96
10	37.4	92	96

Supplementary Figure 4. The reaction with (*R*)-**1p** monitored over different reaction times

The reaction with (*rac*)-, (*R*)-, and (*S*)-**1p** monitored over different reaction times

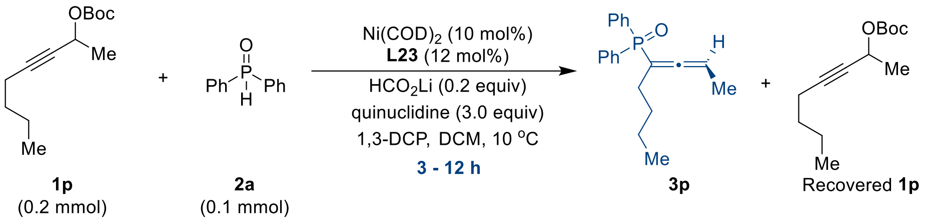

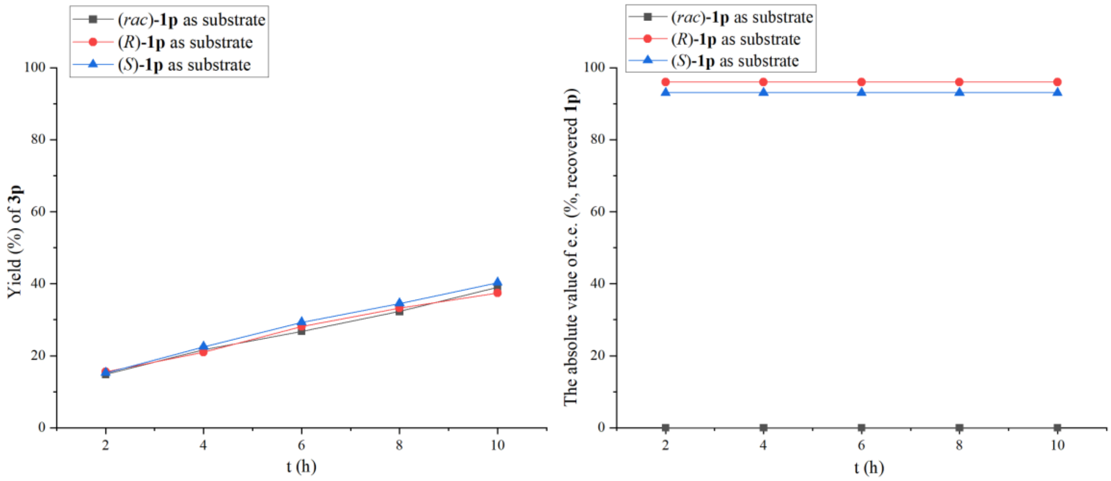

Supplementary Figure 5. Time-course studies of the Ni-catalyzed coupling reactions of **1p**

Nonlinear effect studies

The specified e.e. values of phosphine ligand **L23** were made by combing the certain amounts of optically pure (*S*,*S*)-**L23** with optically pure (*R*,*R*)-**L23**. Seven reactions containing phosphine ligand of racemic, 20%, 40%, 50%, 60%, 80%, and >99% e.e. optical purity were run in parallel (Supplementary Figure 6).

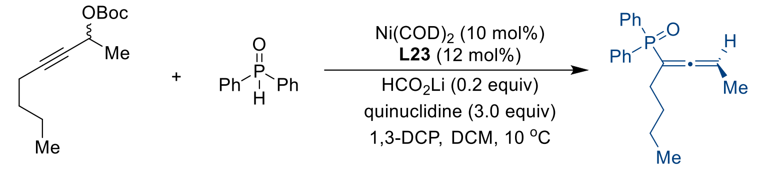

(*rac*)-**1p** (0.3 mmol) **2a** (0.1 mmol) **3p**

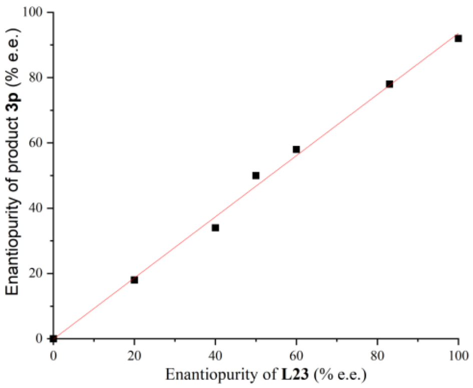

entry	e.e. of **L23**	e.e. of **3p**
1	0	0
2	20	18
3	40	34
4	50	50
5	60	58
6	80	78
7	100	92

Supplementary Figure 6. Nonlinear effect studies

Allenylation pathway leading to **3a** is favored with base additive (Table S2, entries 1,4 vs 2,5), while the acid additive resulted in an improved formation of 1,3-dienylation product **4a** (Table S2, entries 3,6 vs 2,5). To further support our hypothesis, we screened different phosphine ligand in the reaction between **1a** and **2a** with different additive in order to investigate the relationship between the acid and base effect to the product distribution. Using **L3** as the phosphine ligand, we found the ratio of the allenylation product **3a** to the 1,3-dienylation adduct **4a** decreased dramatically to 1:1 (entries 7 vs 8) when the base additive was used. In contrast, with **L8** as the ligand, the acid additive gave increased amount of the 1,3-dienylation adduct **4a** (entries 9 vs 10). These results indicate that the acid additive results in increased amount of **4a**, and the base additive favors the allenylation product **3a**.

Supplementary Table 2 | Additive effect on the outcome of the reaction.

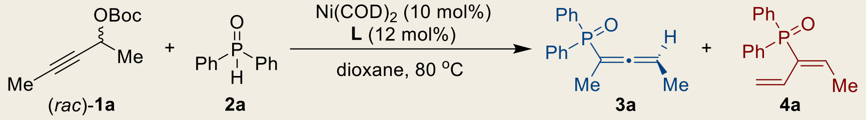

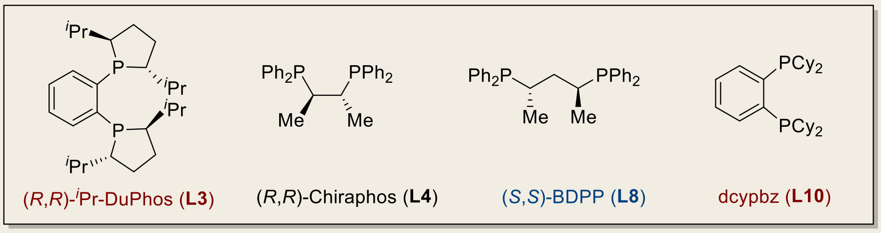

Entry	L	Additive	Yield (%)	3a/4a
1	**L4**	quinuclidine	95	>20:1
2	**L4**	none	70	3:1
3	**L4**	$Ph_2P(O)OH$	39	<1:20
4	**L10**	quinuclidine	93	4:1
5	**L10**	none	42	1:3
6	**L10**	$Ph_2P(O)OH$	40	<1:20
7	**L3**	none	45	<1:20
8	**L3**	quinuclidine	89	1:1
9	**L8**	none	52	>20:1
10	**L8**	$Ph_2P(O)OH$	50	3:1

Reactions were conducted by using $Ni(COD)_2$ (10 mol%), **L** (12 mol%), (*rac*)-**1a** (0.3 mmol), **2a** (0.1 mmol), and quinuclidine (1.5 equiv) or diphenylphosphinic acid (0.5 equiv) in dioxane (1 mL) at 80 °C, 24 h. Isolated yield of the mixture **3a** and **4a** after chromatography are shown.

Deuterium-labeling experiments:

General procedure for deuterium labeling experiments of the allenylation: In a 10 mL Schlenk tube, $Ni(COD)_2$ (2.8 mg, 0.01 mmol, 10 mol%) and **L23** (7.6 mg, 0.012 mmol, 12 mol%) were stirred in anhydrous DCM (1 mL) under argon at room temperature for 20 min. Propargylic carbonate **1b** (0.3 mmol), phosphine oxide **2a** (0.1 mmol, 1.0 equiv), quinuclidine (33.3 mg, 0.3 mmol), HCO_2Li (1.4 mg, 0.02 mmol) and methanol-*d* (1.0 mmol) were then added successively. The reaction mixture was stirred at 25 °C until the reaction was complete (monitored by TLC). The mixture was subjected to silica gel column chromatography directly for purification.

General procedure for deuterium labeling experiments of the 1,3-dienylation: In a 10 mL Schlenk tube, $Ni(COD)_2$ (1.4 mg, 0.005 mmol, 5 mol%) and ^{i}PrDuphos **L3** (2.5 mg, 0.006 mmol, 6 mol%) were stirred in anhydrous DMF (1 mL) under argon at 80 °C for 10 min. Propargylic carbonate **1b** (0.12 mmol) , phosphine oxide **2a** (0.1 mmol, 1.0 equiv) and methanol-*d* (1.0 mmol) were then added successively. The reaction mixture was stirred at 80 °C until the reaction was complete (monitored by TLC). The reaction mixture was concentrated, and the residue was purified by flash column chromatography on silica gel to give the desired product.

Initially, deuteration experiments for the nickel-catalyzed allenylation were conducted (Supplementary Figure 7a and Supplementary Figure 7b). Propargylic carbonate **1b** was subjected to the nickel-catalyzed allenylation system in MeOD. After the reaction, **3g** was obtained as the major stereoisomer, and deuteration of the product **3g-D** was not observed (Supplementary Figure 7a). In addition, the nickel-catalyzed allenylation of propargylic carbonate **1b-D** (Supplementary

Figure 7b), which bears deuterium atom at the propargylic position, provided the product **3g-D** (0.96 D). These results indicate that methanol is not involved in the reaction mechanism of the allenylation reaction.

In addition, deuteration experiments for the nickel-catalyzed 1,3-dienylation were also conducted. The treatment of **1b** with MeOD under the nickel-catalyzed 1,3-dienylation system gave the deuterium-incorporated diene **4b-D$_3$** with a high D/H ratio (Supplementary Figure 7c, 0.7 D; Supplementary Figure 7d, 0.63 D), which is also consistent with the proposed mechanism in the acid condition. In addition, the propargylic carbonate **1b-D** was subject to the 1,3-dienylation reaction, leading to the product **4b-D** with similar deuterium incorporation (Supplementary Figure 7e), which suggested that hydrogen at the propargylic position did not contribute to the protonation event. In addition, we could not observe deuterium-incorporated product when the control experiment using **4b** and MeOD was conducted (Supplementary Figure 7f).

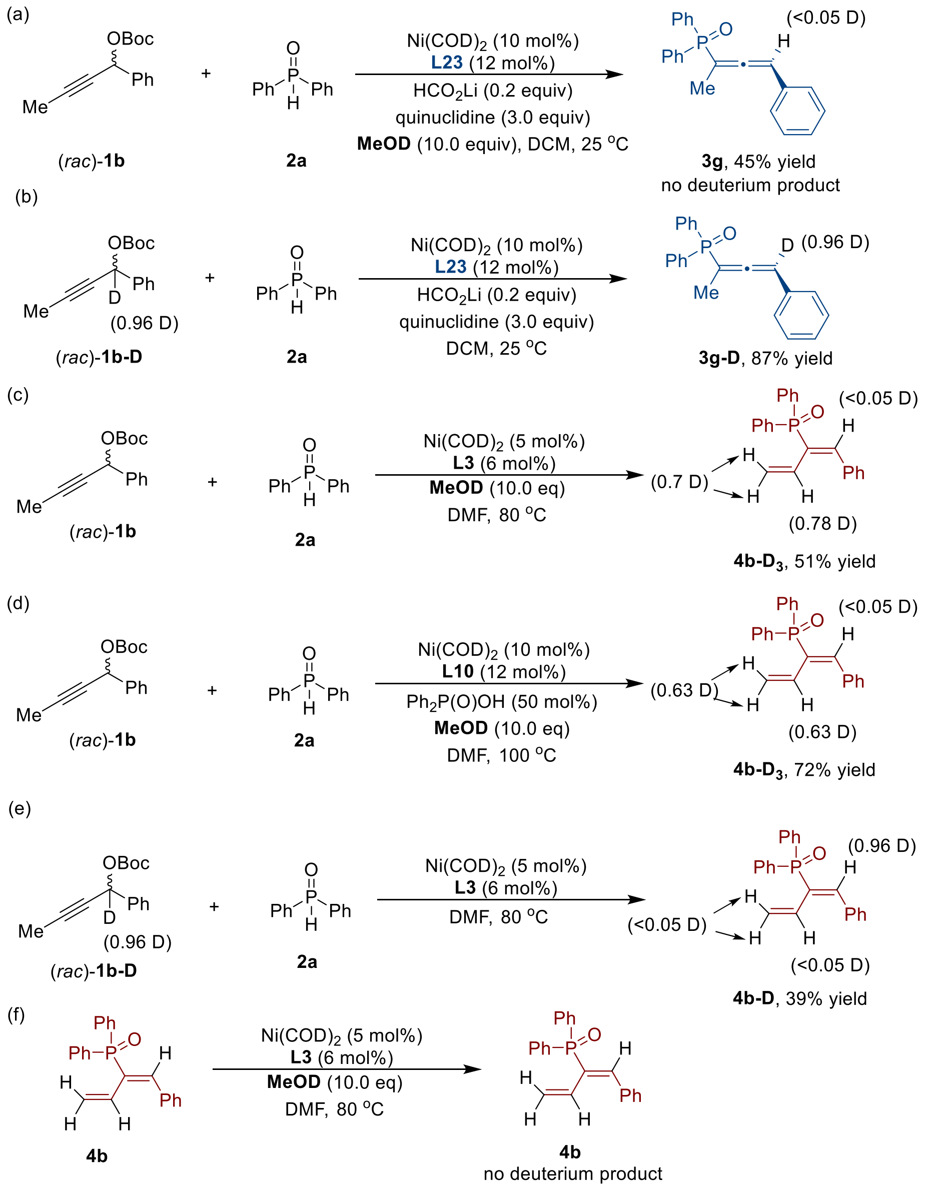

Supplementary Figure 7. Deuteration experiments for the nickel-catalyzed 1,3-dienylation and allenylation of phosphine oxides.

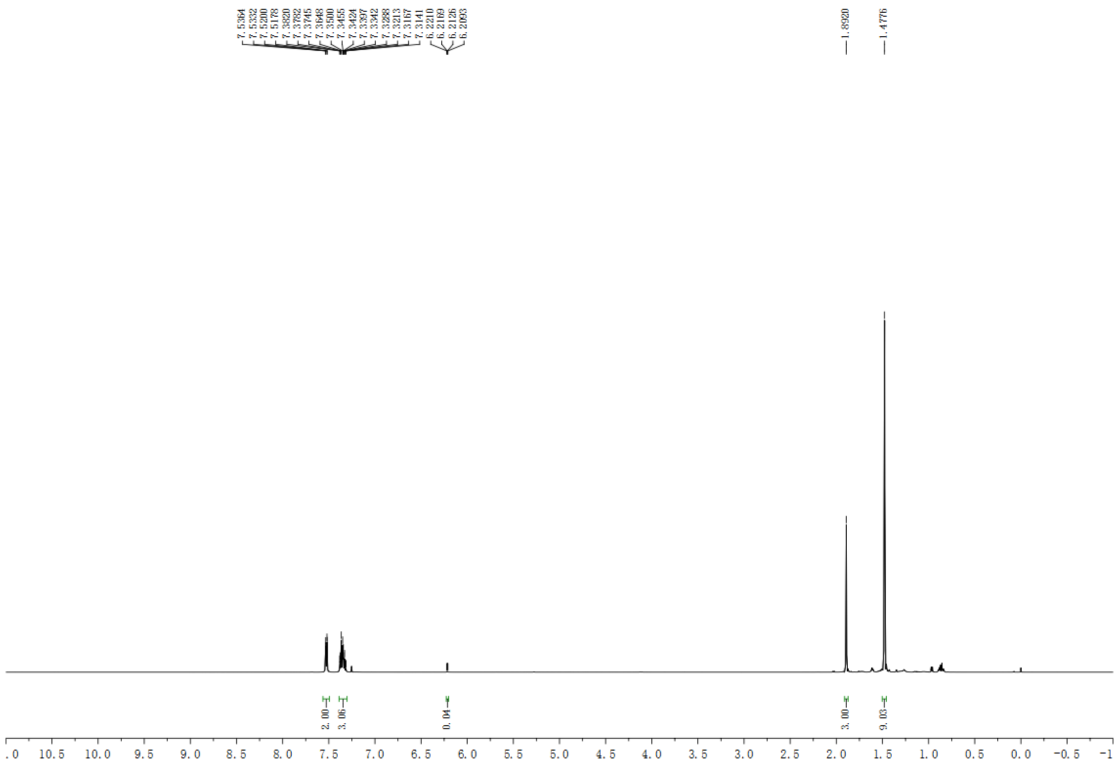

Supplementary Figure 8. ^{1}H NMR spectrum of (*rac*)**-1b-D**

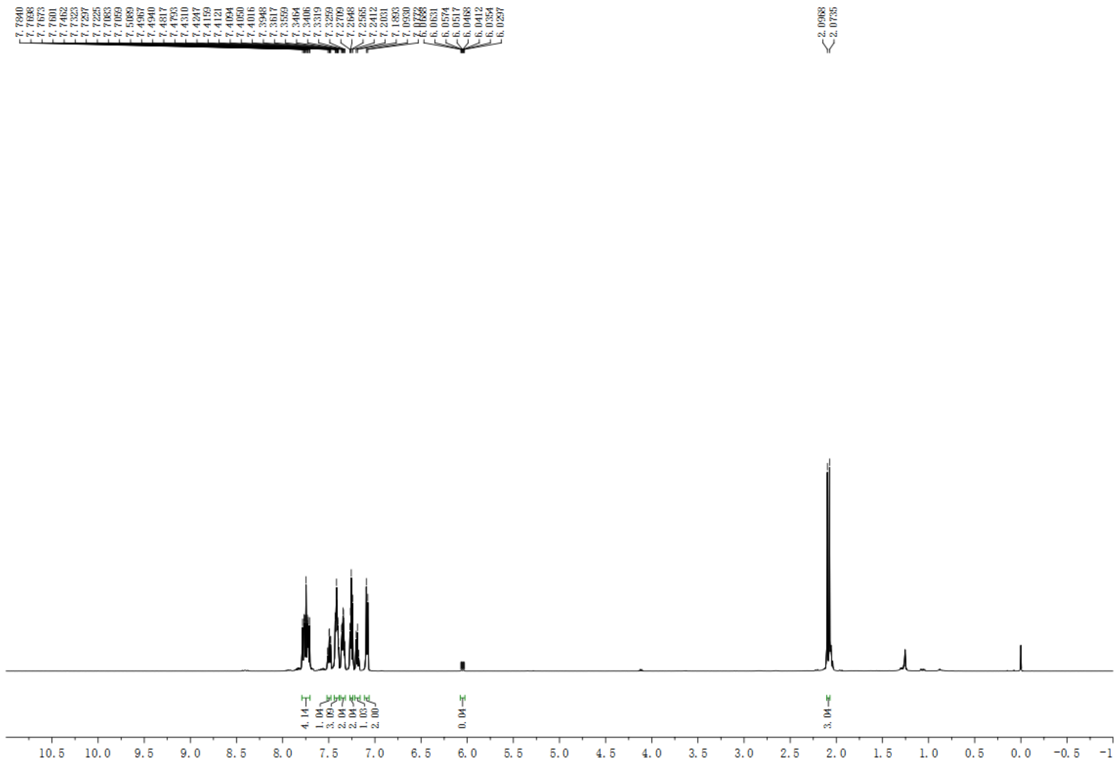

Supplementary Figure 9. ^{1}H NMR spectrum of **3g-D**

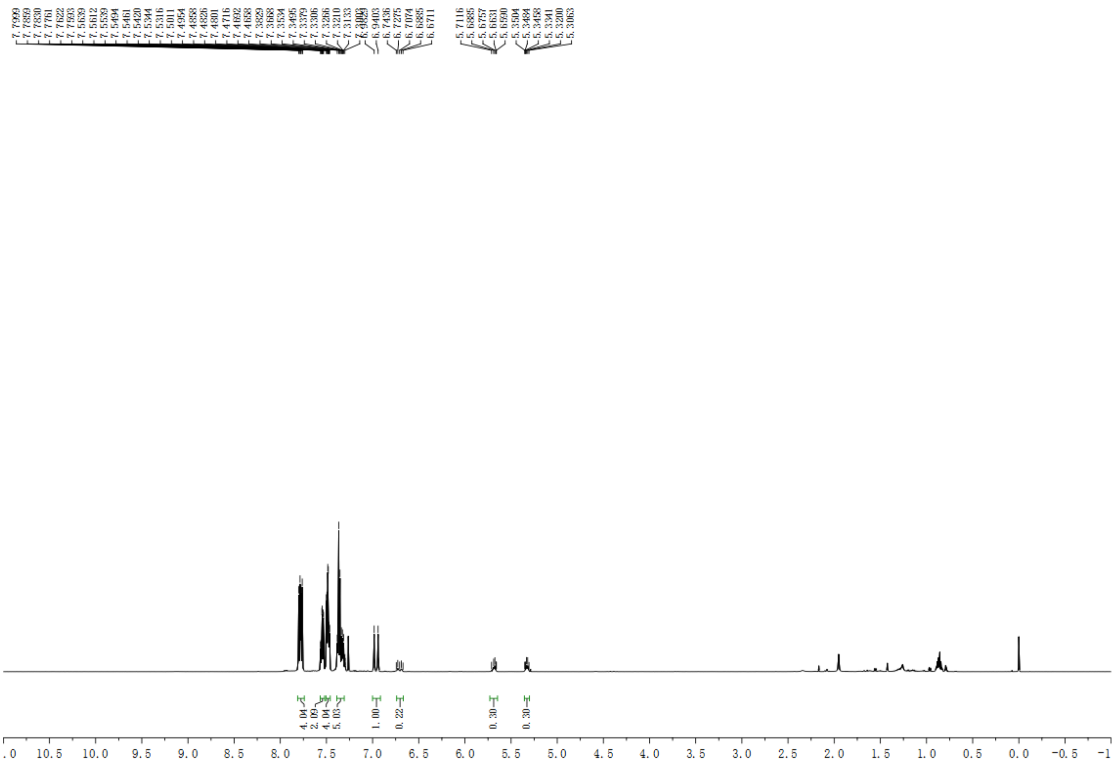

Supplementary Figure 10. ^{1}H NMR spectrum of **4b-D$_3$**

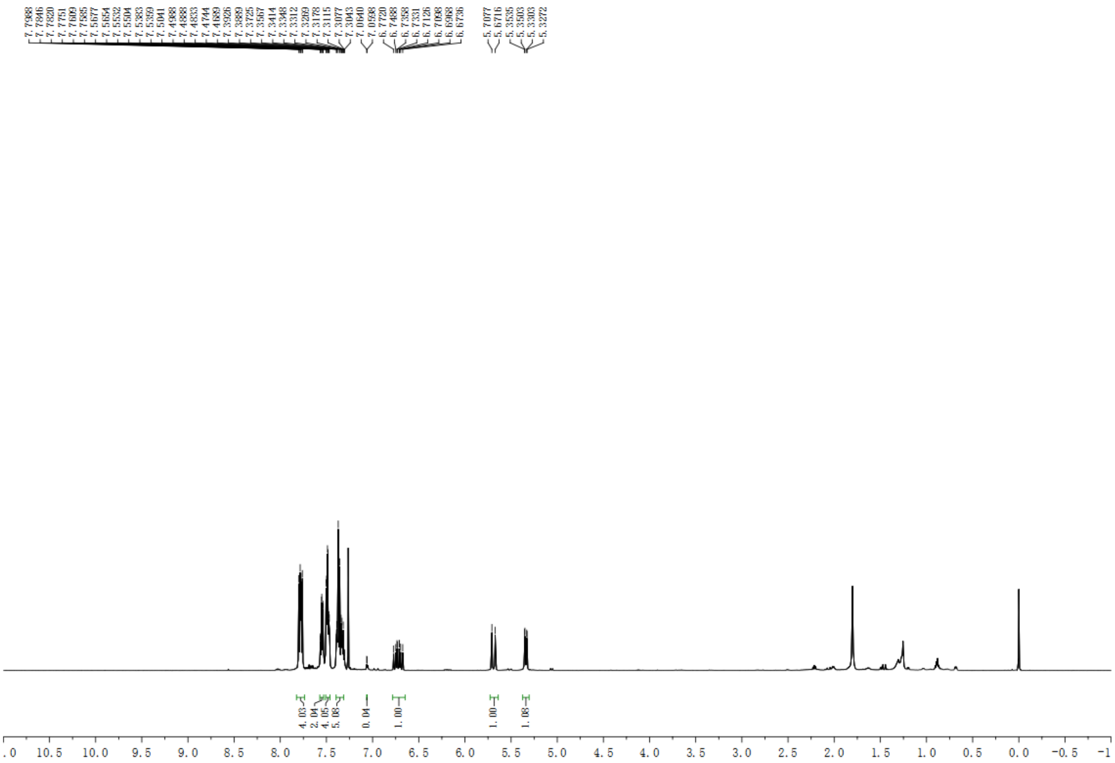

Supplementary Figure 11. ^{1}H NMR spectrum of **4b-D**

ESI-MS studies:

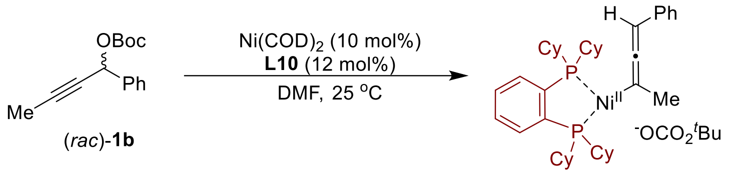

In a 10 mL Schlenk tube, Ni(COD)$_2$ (2.8 mg, 0.01 mmol, 10 mol%) and dcypbz **L10** (5.6 mg, 0.012 mmol, 12 mol%) were stirred in 1 mL anhydrous DMF under argon at 50 °C for 20 min. Tert-butyl (1-phenylbut-2-yn-1-yl) carbonate were then added successively, the mixture were stirred at 25 °C for 30 min and the solution was determined by ESI-MS.

The proposed allenylnickel intermediate in the crude reaction solution was supported by ESI-MS analysis for our standard reaction. The signals at m/z = 657.3283 with its characteristic isotope distribution by mass spectrometry matched with the calculated patterns for the intermediate [Ni(**L10**)(allenyl)]$^+$.

ESI-MS:
$[C_{40}H_{57}NiP_2]^+$
Calcd : 657.3283
Found : 657.3290

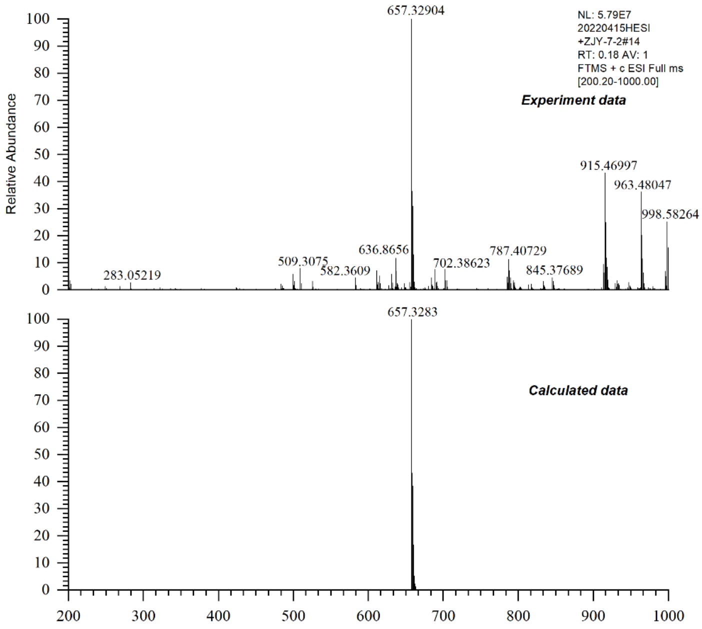

Fit the isotope distribution:

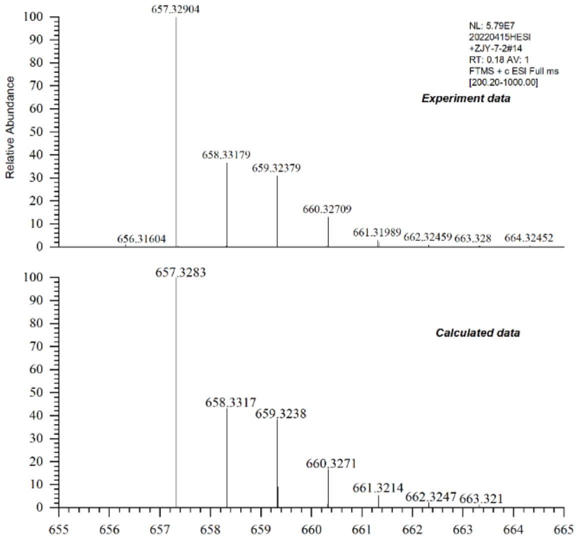

Control experiment of the [2,3]-sigmatropic rearrangement:

To gain more information about the possible intermediates, we turned our attention to study the [2,3]-sigmatropic rearragenment. The enantioenriched propargylic alcohol (*S*-**S1**, 95% e.e.) was selectively converted into the *S*-**3p** in good yield with 95% e.e., indicating the efficient transformation of allenylphosphine oxide from the chiral propargyl phsophinate via the [2,3]-sigmatropic rearrangement.

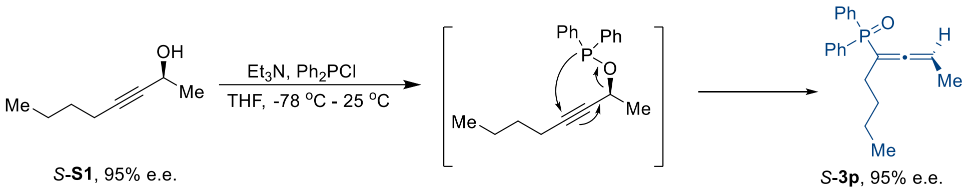

3. Supplementary Methods

General procedure for the synthesis of racemic allene products.

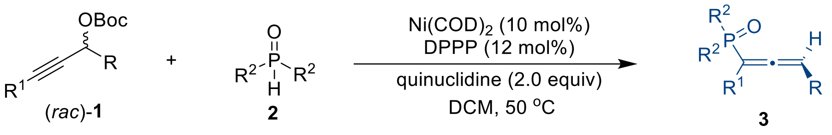

In a 10 mL Schlenk tube, $Ni(COD)_2$ (2.8 mg, 0.01 mmol, 10 mol%) and ligand DPPP (4.9 mg, 0.012 mmol, 12 mol%) were stirred in 1 mL anhydrous DCM under argon at room temperature for 10 min. Propargylic carbonate **1** (0.3 mmol, 3.0 equiv), phosphine oxide **2** (0.1 mmol) and quinuclidine (22.2 mg, 0.2 mmol) were then added successively. The reaction mixture was stirred at 50 °C overnight. The mixture was subjected to silica gel column chromatography directly for purification.

General procedure for the synthesis of chiral allene products.

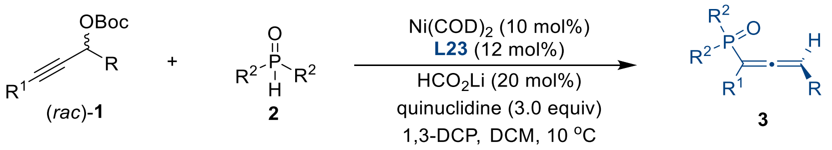

In a 10 mL Schlenk tube, $Ni(COD)_2$ (2.8 mg, 0.01 mmol, 10 mol%), **L23** (7.6 mg, 0.012 mmol, 12 mol%) and 1,3-DCP (30 μL) were stirred in 1 mL anhydrous DCM under argon at room temperature for 20 min. Propargylic carbonate **1** (0.3 mmol), phosphine oxide **2** (0.1 mmol, 1.0 equiv), quinuclidine (33.3 mg, 0.3 mmol), and HCO_2Li (1.4 mg, 0.02 mmol) were then added successively. The reaction mixture was stirred at 10 °C until the reaction was complete (monitored by TLC). The mixture was subjected to silica gel column chromatography directly for purification.

General procedure for the synthesis of diene products.

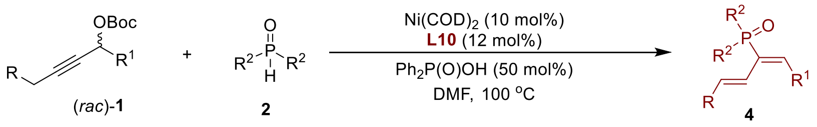

In a 10 mL Schlenk tube, $Ni(COD)_2$ (2.8 mg, 0.01 mmol, 10 mol%) and dcypbz **L10** (5.6 mg, 0.012 mmol, 12 mol%) were stirred in 1 mL anhydrous DMF under argon at 80 °C for 10 min. Propargylic carbonate **1** (0.12 mmol), phosphine oxide **2** (0.1 mmol, 1.0 equiv) and $HOP(O)Ph_2$ (10.9 mg, 0.05 mmol) were then added successively. The reaction mixture was stirred at 100 °C until the reaction

was complete (monitored by TLC). The reaction mixture was concentrated, and the residue was purified by flash column chromatography on silica gel to give the desired product.

(*S*)-penta-2,3-dien-2-yldiphenylphosphine oxide (3a)

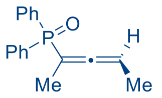

Colorless oil (24.2 mg, 90%); Reaction time: 84 h; **^{1}H NMR (400 MHz, $CDCl_3$)** δ 7.79 – 7.67 (m, 4H), 7.54 – 7.41 (m, 6H), 5.09 – 4.88 (m, 1H), 1.92 (dd, *J* = 11.8, 2.9 Hz, 3H), 1.50 – 1.40 (m, 3H). **^{13}C NMR (100 MHz, $CDCl_3$)** δ 209.89 (d, *J* = 7.1 Hz), 132.25 (d, *J* = 104.3 Hz), 131.83 (d, *J* = 104.0 Hz), 131.81 (d, *J* = 7.8 Hz), 131.79 (d, *J* = 7.5 Hz), 131.70 (d, *J* = 7.6 Hz), 128.34 (d, *J* = 12.1 Hz), 92.58 (d, *J* = 102.7 Hz), 87.15 (d, *J* = 13.7 Hz), 14.41 (d, *J* = 7.2 Hz), 13.03 (d, *J* = 6.1 Hz). **^{31}P NMR (202 MHz, $CDCl_3$)** δ 31.07. **APCI-MS:** calculated $[C_{17}H_{17}OP + H]^+$: 269.1090, found: 269.1091. $[\alpha]^{20}_{D}$ = +55.5 (c = 0.81, CH_2Cl_2). The product was analyzed by HPLC to determine the enantiomeric excess: 90% e.e. (CHIRALPAK AD-H, hexane/*i*-PrOH = 90/10, detector: 220 nm, T = 25 °C, flow rate: 1 mL/min), t_1(major) = 13.23 min, t_2(minor) = 14.47 min.

(*S*)-hexa-2,3-dien-2-yldiphenylphosphine oxide (3b)

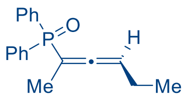

Colorless oil (21.2 mg, 76%); Reaction time: 84 h; **^{1}H NMR (500 MHz, $CDCl_3$)** δ 7.82 – 7.66 (m, 4H), 7.56 – 7.37 (m, 6H), 5.13 – 4.97 (m, 1H), 1.94 (dd, *J* = 11.8, 2.7 Hz, 3H), 1.86 – 1.73 (m, 2H), 0.75 (t, *J* = 7.4 Hz, 3H). **^{13}C NMR (125 MHz, $CDCl_3$)** δ 208.79 (d, *J* = 7.5 Hz), 132.08 (d, *J* = 104.6 Hz), 131.95 (d, *J* = 103.9 Hz), 131.85 (d, *J* = 5.4 Hz), 131.81 (d, *J* = 11.3 Hz), 131.77 (d, *J* = 5.3 Hz), 128.41 (d, *J* = 8.7 Hz), 128.31 (d, *J* = 8.5 Hz), 94.15 (d, *J* = 13.3 Hz), 93.59 (d, *J* = 103.0 Hz), 21.29 (d, *J* = 5.7 Hz), 14.69 (d, *J* = 7.3 Hz), 13.30 (d, *J* = 2.9 Hz). **^{31}P NMR (202 MHz, $CDCl_3$)** δ 30.95. **APCI-MS:** calculated $[C_{18}H_{19}OP+H]^+$: 283.1246, found: 283.1248. $[\alpha]^{20}_{D}$ = +102.4 (c = 0.71, CH_2Cl_2). The product was analyzed by HPLC to determine the enantiomeric excess: 93% e.e. (CHIRALPAK IC, hexane/*i*-PrOH = 80/20, detector: 220 nm, T = 25 °C, flow rate: 1 mL/min), t_1(major) = 17.83 min, t_2(minor) = 19.75 min.

(*S*)-hepta-2,3-dien-2-yldiphenylphosphine oxide (3c)

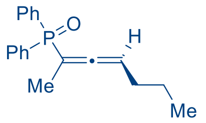

Colorless oil (26.2 mg, 89%); Reaction time: 84 h; **^{1}H NMR (500 MHz, $CDCl_3$)** δ 7.79 – 7.66 (m, 4H), 7.57 – 7.35 (m, 6H), 5.07 – 4.96 (m, 1H), 1.94 (dd, *J* = 11.7, 2.7 Hz, 3H), 1.83 – 1.71 (m, 2H), 1.19 – 1.09 (m, 2H), 0.77 (t, *J* = 7.4 Hz, 3H). **^{13}C NMR (125 MHz, $CDCl_3$)** δ 209.05 (d, *J* = 7.6 Hz), 132.10 (d, *J* = 104.4 Hz), 131.99 (d, *J* = 104.0 Hz), 131.79 (d, *J* = 9.3 Hz), 131.83 (d, *J* = 9.7 Hz), 128.42 (d, *J* = 9.3 Hz), 128.32 (d, *J* = 9.3 Hz), 92.89 (d, *J* = 103.1 Hz), 92.25 (d, *J* = 13.6 Hz), 30.02 (d, *J* = 5.5 Hz), 22.23 (d, *J* = 3.0 Hz), 14.67 (d, *J* = 7.2 Hz), 13.59. **^{31}P NMR (202 MHz, $CDCl_3$)** δ 30.92. **APCI-MS:** calculated $[C_{19}H_{21}OP + H]^+$: 297.1403, found: 297.1403. $[\alpha]^{20}_{D}$ = +114.2 (c = 0.87, CH_2Cl_2). The product was analyzed by HPLC to determine the enantiomeric excess: 91% e.e. (CHIRALPAK IC, hexane/*i*-PrOH = 80/20, detector: 220 nm, T = 25 °C, flow rate: 1 mL/min), t_1(major) = 17.19 min, t_2(minor) = 18.64 min.

(*S*)-octa-2,3-dien-2-yldiphenylphosphine oxide (3d)

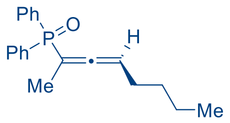

Colorless oil (26.4 mg, 85%); Reaction time: 108 h; **^{1}H NMR (500 MHz, $CDCl_3$)** δ 7.76 – 7.67 (m, 4H), 7.52 – 7.39 (m, 6H), 5.07 – 4.95 (m, 1H), 1.92 (dd, *J* = 11.8, 2.8 Hz, 3H), 1.83 – 1.70 (m, 2H), 1.17 – 1.02 (m, 4H), 0.79 (t, *J* = 7.2 Hz, 3H). **^{13}C NMR (125 MHz, $CDCl_3$)** δ 209.01 (d, *J* = 7.6 Hz), 131.84 (d, *J* = 6.7 Hz), 131.80 (d, *J* = 8.9 Hz), 132.08 (d, *J* = 104.6 Hz), 131.99 (d, *J* = 103.9 Hz),

131.76 (d, J = 6.5 Hz), 128.40 (d, J = 10.3 Hz), 128.31 (d, J = 10.2 Hz), 92.91 (d, J = 103.8 Hz), 92.46 (d, J = 13.4 Hz), 31.08 (d, J = 2.9 Hz), 27.64 (d, J = 5.5 Hz), 22.09, 14.66 (d, J = 7.2 Hz), 13.90. **^{31}P NMR (202 MHz, $CDCl_3$)** δ 30.84. **APCI-MS:** calculated $[C_{20}H_{23}OP + H]^+$: 311.1559, found: 311.1558. $[\alpha]^{20}_D$ = +101.4 (c = 0.88, CH_2Cl_2). The product was analyzed by HPLC to determine the enantiomeric excess: 91% e.e. (CHIRALPAK IC, hexane/*i*-PrOH = 80/20, detector: 220 nm, T = 25 °C, flow rate: 1 mL/min), t_1(major) = 16.84 min, t_2(minor) = 18.33 min.

(*S*)-(5-methylhexa-2,3-dien-2-yl)diphenylphosphine oxide (3e)

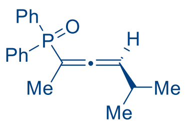

Colorless oil (18 mg, 61%); Reaction time: 84 h; **^{1}H NMR (400 MHz, $CDCl_3$)** δ 7.81 – 7.64 (m, 4H), 7.57 – 7.38 (m, 6H), 5.10 – 4.97 (m, 1H), 2.16 – 2.03 (m, 1H), 1.95 (dd, J = 11.7, 2.9 Hz, 3H), 0.73 (d, J = 6.8 Hz, 6H). **^{13}C NMR (100 MHz, $CDCl_3$)** δ 207.75 (d, J = 7.8 Hz), 132.16 (d, J = 103.7 Hz), 132.00 (d, J = 104.7 Hz), 131.98 (d, J = 9.4 Hz), 131.81 (d, J = 9.3 Hz), 131.81 (d, J = 2.9 Hz), 128.44 (d, J = 10.4 Hz), 128.32 (d, J = 10.4 Hz), 99.74 (d, J = 13.5 Hz), 94.34 (d, J = 103.4 Hz), 27.82 (d, J = 5.2 Hz), 22.41 (d, J = 3.5 Hz), 21.97 (d, J = 2.6 Hz), 14.86 (d, J = 7.3 Hz). **^{31}P NMR (202 MHz, $CDCl_3$)** δ 30.82. **APCI-MS:** calculated $[C_{19}H_{21}OP + H]^+$: 297.1403, found: 297.1405. $[\alpha]^{20}_D$ = +136.3 (c = 0.60, CH_2Cl_2). The product was analyzed by HPLC to determine the enantiomeric excess: 92% e.e. (CHIRALPAK IC, hexane/*i*-PrOH = 80/20, detector: 220 nm, T = 25 °C, flow rate: 1 mL/min), t_1(major) = 16.13 min, t_2(minor) = 18.85 min.

(*S*)-(4-cyclohexylbuta-2,3-dien-2-yl)diphenylphosphine oxide (3f)

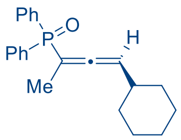

Colorless oil (25.0 mg, 89%); Reaction time: 84 h; **^{1}H NMR (500 MHz, $CDCl_3$)** δ 7.78 – 7.66 (m, 4H), 7.54 – 7.47 (m, 2H), 7.47 – 7.41 (m, 4H), 5.08 – 4.94 (m, 1H), 1.95 (dd, J = 11.7, 2.9 Hz, 3H), 1.82 – 1.71 (m, 1H), 1.62 – 1.53 (m, 3H), 1.51 – 1.44 (m, 1H), 1.39 – 1.34 (m, 1H), 1.20 – 1.09 (m, 2H), 1.08 – 0.97 (m, 1H), 0.82 – 0.72 (m, 1H), 0.72 – 0.61 (m, 1H). **^{13}C NMR (125 MHz, $CDCl_3$)** δ 208.12 (d, J = 7.9 Hz), 132.12 (d, J = 103.1 Hz), 132.00 (d, J = 104.5 Hz), 131.99 (d, J = 9.3 Hz), 131.80 (d, J = 10.0 Hz), 131.78 (d, J = 10.7 Hz), 128.43 (d, J = 12.0 Hz), 128.29 (d, J = 12.1 Hz), 98.34 (d, J = 13.3 Hz), 93.85 (d, J = 103.4 Hz), 36.88 (d, J = 4.9 Hz), 32.85 (d, J = 2.9 Hz), 32.37 (d, J = 2.4 Hz), 26.00, 25.90 (d, J = 6.1 Hz), 14.85 (d, J = 7.4 Hz). **^{31}P NMR (202 MHz, $CDCl_3$)** δ 30.79. **APCI-MS:** calculated $[C_{22}H_{25}OP + H]^+$: 337.1716, found: 337.1717. $[\alpha]^{20}_D$ = +164.3 (c = 0.83, CH_2Cl_2). The product was analyzed by HPLC to determine the enantiomeric excess: 91% e.e. (CHIRALPAK IC, hexane/*i*-PrOH = 80/20, detector: 220 nm, T = 25 °C, flow rate: 1 mL/min), t_1(major) = 18.15 min, t_2(minor) = 20.47 min.

(*S*)-diphenyl(4-phenylbuta-2,3-dien-2-yl)phosphine oxide (3g)

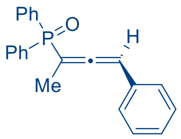

Colorless oil (28.4 mg, 86%); Reaction time: 84 h; **^{1}H NMR (500 MHz, $CDCl_3$)** δ 7.82 – 7.68 (m, 4H), 7.55 – 7.45 (m, 1H), 7.45 – 7.39 (m, 3H), 7.38 – 7.31 (m, 2H), 7.30 – 7.23 (m, 2H), 7.22 – 7.17 (m, 1H), 7.10 – 7.03 (m, 2H), 6.19 – 5.93 (m, 1H), 2.09 (dd, J = 11.7, 2.9 Hz, 3H). **^{13}C NMR (125 MHz, $CDCl_3$)** δ 210.02 (d, J = 6.4 Hz), 132.67 (d, J = 6.7 Hz), 132.08 (d, J = 2.7 Hz), 131.98 (d, J = 2.7 Hz), 131.82 (d, J = 105.2 Hz), 131.78 (d, J = 9.8 Hz), 131.63 (d, J = 9.5 Hz), 131.43 (d, J = 104.0 Hz), 128.48 (d, J = 11.4 Hz), 128.39 (d, J = 11.4 Hz), 128.44, 127.60, 126.99 (d, J = 2.2 Hz), 97.64 (d, J = 99.7 Hz), 95.92 (d, J = 13.7 Hz), 14.58 (d, J = 6.3 Hz). **^{31}P NMR (202 MHz, $CDCl_3$)** δ 30.08. **APCI-MS:** calculated $[C_{22}H_{19}OP + H]^+$: 331.1246, found: 331.1247. $[\alpha]^{20}_D$ = +409.8 (c = 1.00, CH_2Cl_2). The product was analyzed by HPLC to determine the enantiomeric excess: 89% e.e. (CHIRALPAK AD-

H, hexane/*i*-PrOH = 90/10, detector: 254 nm, T = 25 °C, flow rate: 1 mL/min), t_1(major) = 18.83 min, t_2(minor) = 20.55 min.

(*S*)-(4-(4-fluorophenyl)buta-2,3-dien-2-yl)diphenylphosphine oxide (3h)

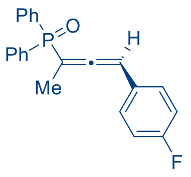

Colorless oil (28.4 mg, 82%); Reaction time: 84 h; **^{1}H NMR (500 MHz, $CDCl_3$)** δ 7.82 – 7.74 (m, 2H), 7.73 – 7.67 (m, 2H), 7.54 – 7.48 (m, 1H), 7.45 – 7.38 (m, 3H), 7.37 – 7.31 (m, 2H), 7.08 – 7.00 (m, 2H), 6.97 – 6.91 (m, 2H), 6.06 – 5.99 (m, 1H), 2.08 (dd, *J* = 11.7, 2.7 Hz, 3H). **^{13}C NMR (125 MHz, $CDCl_3$)** δ 209.75 (dd, J = 6.2, 2.4 Hz), 162.17 (d, J = 247.4 Hz), 132.09 (d, J = 2.7 Hz), 131.95 (d, J = 2.7 Hz), 131.64 (d, J = 9.6 Hz), 131.63 (d, J = 105.5 Hz), 131.49 (d, J = 9.8 Hz), 131.19 (d, J = 104.5 Hz), 128.48 (d, J = 12.3 Hz), 128.38 (d, J = 2.2 Hz), 128.32 (d, J = 2.4 Hz), 128.30 (d, J = 12.5 Hz), 115.66 (d, J = 22.0 Hz), 97.88 (d, J = 99.1 Hz), 94.93 (d, J = 13.7 Hz), 14.50 (d, J = 6.3 Hz). **^{31}P NMR (202 MHz, $CDCl_3$)** δ 30.05. **^{19}F NMR (471 MHz, $CDCl_3$)** δ -114.17. **APCI-MS:** calculated $[C_{22}H_{18}OP + H]^+$: 349.1152, found: 349.1154. $[\alpha]^{20}_D$ = +376.0 (c = 0.71, CH_2Cl_2). The product was analyzed by HPLC to determine the enantiomeric excess: 90% e.e. (CHIRALPAK AD-H, hexane/*i*-PrOH = 90/10, detector: 254 nm, T = 25 °C, flow rate: 1 mL/min), t_1(major) = 21.1min, t_2(minor) = 28.17 min.

(*S*)-(4-(4-chlorophenyl)buta-2,3-dien-2-yl)diphenylphosphine oxide (3i)

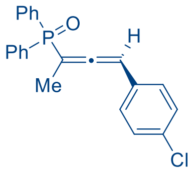

Colorless oil (25.4 mg, 70%); Reaction time: 84 h; **^{1}H NMR (500 MHz, $CDCl_3$)** δ 7.82 – 7.73 (m, 2H), 7.73 – 7.67 (m, 2H), 7.54 – 7.48 (m, 1H), 7.47 – 7.40 (m, 3H), 7.37 – 7.31 (m, 2H), 7.24 – 7.18 (m, 2H), 7.07 – 6.96 (m, 2H), 6.07 – 5.88 (m, 1H), 2.08 (dd, *J* = 11.7, 2.9 Hz, 3H). **^{13}C NMR (125 MHz, $CDCl_3$)** δ 209.86 (d, *J* = 6.2 Hz), 133.24 (d, *J* = 1.6 Hz), 132.20 (d, *J* = 2.7 Hz), 132.08 (d, *J* = 2.6 Hz), 131.74 (d, *J* = 9.5 Hz), 131.67 (d, *J* = 100.4 Hz), 131.58 (d, *J* = 9.9 Hz), 131.24 (d, *J* = 4.6 Hz), 131.24 (d, *J* = 104.0 Hz), 128.94, 128.57 (d, *J* = 12.1 Hz), 128.43 (d, *J* = 12.1 Hz), 128.10 (d, *J* = 1.9 Hz), 98.26 (d, *J* = 98.4 Hz), 95.02 (d, *J* = 13.4 Hz), 14.53 (d, *J* = 6.2 Hz). **^{31}P NMR (202 MHz, $CDCl_3$)** δ 29.95. **APCI-MS:** calculated $[C_{22}H_{18}ClOP + H]^+$: 365.0857, found: 365.0866. $[\alpha]^{20}_D$ = +502.8 (c = 0.85, CH_2Cl_2). The product was analyzed by HPLC to determine the enantiomeric excess: 88% e.e. (CHIRALPAK AD-H, hexane/*i*-PrOH = 90/10, detector: 254 nm, T = 25 °C, flow rate: 1 mL/min), t_1(major) = 23.87 min, t_2(minor) = 28.34 min.

(*S*)-diphenyl(4-(p-tolyl)buta-2,3-dien-2-yl)phosphine oxide (3j)

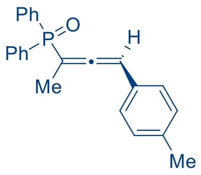

Colorless oil (27.5 mg, 80%); Reaction time: 84 h; **^{1}H NMR (500 MHz, $CDCl_3$)** δ 7.80 – 7.68 (m, 4H), 7.52 – 7.46 (m, 1H), 7.45 – 7.39 (m, 3H), 7.38 – 7.32 (m, 2H), 7.07 (d, *J* = 7.9 Hz, 2H), 6.98 (d, *J* = 7.8 Hz, 2H), 6.08 – 5.97 (m, 1H), 2.32 (s, 3H), 2.07 (dd, *J* = 11.7, 2.9 Hz, 3H). **^{13}C NMR (125 MHz, $CDCl_3$)** δ 210.07 (d, *J* = 6.4 Hz), 137.50 (d, *J* = 1.4 Hz), 132.04 (d, *J* = 2.6 Hz), 131.96 (d, *J* = 2.6 Hz), 131.85 (d, *J* = 104.9 Hz), 131.78 (d, *J* = 9.8 Hz), 131.64 (d, *J* = 9.8 Hz), 131.47 (d, *J* = 104.4 Hz), 129.58 (d, *J* = 7.2 Hz), 129.48, 128.47 (d, *J* = 7.3 Hz), 128.38 (d, *J* = 7.2 Hz), 126.90 (d, *J* = 1.8 Hz), 97.40 (d, *J* = 100.1 Hz), 95.79 (d, *J* = 13.7 Hz), 21.35, 14.64 (d, *J* = 6.5 Hz). **^{31}P NMR (202 MHz, $CDCl_3$)** δ 30.18. **APCI-MS:** calculated $[C_{23}H_{21}OP + H]^+$: 345.1403, found: 345.1405. $[\alpha]^{20}_D$ = +240.1 (c = 0.92, CH_2Cl_2). The product was analyzed by HPLC to determine the enantiomeric excess: 87% e.e. (CHIRALPAK IE, hexane/*i*-PrOH = 70/30, detector: 254 nm, T = 25 °C, flow rate: 1 mL/min), t_1(major) = 18.85 min, t_2(minor) = 21.04 min.

(*S*)-(4-(3-chlorophenyl)buta-2,3-dien-2-yl)diphenylphosphine oxide (3k)

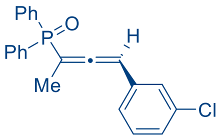

Colorless oil (28.5 mg, 78%); Reaction time: 84 h; **^{1}H NMR (500 MHz, $CDCl_3$)** δ 7.79 – 7.69 (m, 4H), 7.54 – 7.48 (m, 1H), 7.47 – 7.40 (m, 3H), 7.39 – 7.34 (m, 2H), 7.20 – 7.13 (m, 2H), 7.06 – 7.00 (m, 1H), 6.97 – 6.92 (m, 1H), 6.03 – 5.95 (m, 1H), 2.10 (dd, *J* = 11.7, 2.9 Hz, 3H). **^{13}C NMR (125 MHz, $CDCl_3$)** δ 209.79 (d, *J* = 6.4 Hz), 134.71 (d, *J* = 6.8 Hz), 134.62, 132.22 (d, *J* = 2.7 Hz), 132.11 (d, *J* = 2.7 Hz), 131.73 (d, *J* = 9.9 Hz), 131.59 (d, *J* = 9.5 Hz), 131.59 (d, *J* = 105.6 Hz), 131.19 (d, *J* = 104.4 Hz), 128.56 (d, *J* = 12.8 Hz), 128.45 (d, *J* = 12.7 Hz), 128.50, 127.57 (d, *J* = 1.2 Hz), 126.76 (d, *J* = 1.9 Hz), 125.03 (d, *J* = 1.8 Hz), 98.39 (d, *J* = 98.2 Hz), 94.95 (d, *J* = 13.6 Hz), 14.53 (d, *J* = 5.7 Hz). **^{31}P NMR (202 MHz, $CDCl_3$)** δ 29.92. **APCI-MS:** calculated $[C_{22}H_{18}OP + H]^+$: 365.0857, found: 365.0858. $[\alpha]^{20}_D$ = +419.7 (c = 0.95, CH_2Cl_2). The product was analyzed by HPLC to determine the enantiomeric excess: 90% e.e. (CHIRALPAK AD-H, hexane/*i*-PrOH = 90/10, detector: 254 nm, T = 25 °C, flow rate: 1 mL/min), t_1(major) = 19.2 min, t_2(minor) = 26.09 min.

(*S*)-(4-(3-bromophenyl)buta-2,3-dien-2-yl)diphenylphosphine oxide (3l)

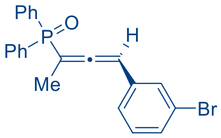

Colorless oil (31.2 mg, 76%); Reaction time: 84 h; **^{1}H NMR (500 MHz, $CDCl_3$)** δ 7.80 – 7.65 (m, 4H), 7.56 – 7.48 (m, 1H), 7.48 – 7.40 (m, 3H), 7.40 – 7.33 (m, 2H), 7.33 – 7.28 (m, 1H), 7.21 – 7.14 (m, 1H), 7.13 – 7.05 (m, 1H), 7.02 – 6.92 (m, 1H), 6.01 – 5.95 (m, 1H), 2.09 (dd, *J* = 11.7, 2.8 Hz, 3H). **^{13}C NMR (125 MHz, $CDCl_3$)** δ 209.77 (d, *J* = 6.3 Hz), 134.98 (d, *J* = 7.0 Hz), 132.22 (d, *J* = 2.7 Hz), 132.13 (d, *J* = 2.7 Hz), 131.72 (d, *J* = 9.5 Hz), 131.59 (d, *J* = 9.7 Hz), 131.56 (d, *J* = 105.5 Hz), 131.19 (d, *J* = 104.9 Hz), 130.47, 130.18, 129.67 (d, *J* = 2.2 Hz), 128.56 (d, *J* = 11.8 Hz), 128.47 (d, *J* = 11.3 Hz), 125.47 (d, *J* = 1.9 Hz), 122.78 (d, *J* = 1.4 Hz), 98.40 (d, *J* = 98.0 Hz), 94.84 (d, *J* = 13.6 Hz), 14.55 (d, *J* = 6.1 Hz). **^{31}P NMR (202 MHz, $CDCl_3$)** δ 29.96. **APCI-MS:** calculated $[C_{22}H_{18}BrOP + H]^+$:409.0351, found: 419.0354. $[\alpha]^{20}_D$ = +332.7 (c = 1.04, CH_2Cl_2). The product was analyzed by HPLC to determine the enantiomeric excess: 90% e.e. (CHIRALPAK AD-H, hexane/*i*-PrOH = 90/10, detector: 254 nm, T = 25 °C, flow rate: 1 mL/min), t_1(major) = 20.34 min, t_2(minor) = 27.33 min.

(*S*)-(4-(furan-3-yl)buta-2,3-dien-2-yl)diphenylphosphine oxide (3m)

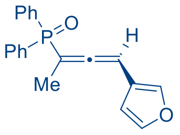

Colorless oil (25.5 mg, 80%); Reaction time: 72 h; **^{1}H NMR (400 MHz, $CDCl_3$)** δ 7.81 – 7.67 (m, 4H), 7.55 – 7.35 (m, 6H), 7.35 – 7.31 (m, 1H), 7.24 – 7.21 (m, 1H), 6.29 – 6.19 (m, 1H), 5.96 (dq, *J* = 10.9, 2.8 Hz, 1H), 2.05 (dd, *J* = 11.7, 2.9 Hz, 3H). **^{13}C NMR (100 MHz, $CDCl_3$)** δ 210.01 (d, *J* = 6.2 Hz), 143.66, 139.86 (d, *J* = 3.6 Hz), 132.02 (d, *J* = 2.8 Hz), 131.94 (d, *J* = 2.8 Hz), 131.87 (d, *J* = 104.8 Hz), 131.71 (d, *J* = 9.6 Hz), 131.55 (d, *J* = 9.6 Hz), 131.37 (d, *J* = 104.3 Hz), 128.46 (d, *J* = 3.4 Hz), 128.34 (d, *J* = 3.4 Hz), 118.06 (d, *J* = 7.7 Hz), 108.80, 96.76 (d, *J* = 99.8 Hz), 86.12 (d, *J* = 13.6 Hz), 14.64 (d, *J* = 6.4 Hz). **^{31}P NMR (162 MHz, $CDCl_3$)** δ 29.89. **ESI-MS:** calculated $[C_{20}H_{17}O_2S + H]^+$: 321.1039, found: 321.1046. $[\alpha]^{20}_D$ = +193.8 (c = 0.85, CH_2Cl_2). The product was analyzed by HPLC to determine the enantiomeric excess: 86% e.e. (CHIRALPAK IC, hexane/*i*-PrOH = 70/30, detector: 254 nm, T = 25 °C, flow rate: 1 mL/min), t_1(major) = 12.91 min, t_2(minor) = 14.73 min.

(*S*)-diphenyl(4-(thiophen-3-yl)buta-2,3-dien-2-yl)phosphine oxide (3n)

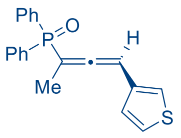

Colorless oil (30.0 mg, 89%); Reaction time: 72 h; **^{1}H NMR (500 MHz, $CDCl_3$)** δ 7.79 – 7.67 (m, 4H), 7.52 – 7.47 (m, 1H), 7.45 – 7.39 (m, 3H), 7.38 – 7.33 (m, 2H), 7.26 – 7.20 (m, 1H), 6.92 – 6.86 (m, 2H), 6.12 (dq, *J* = 10.9, 2.8 Hz, 1H), 2.06 (dd, *J* = 11.7, 2.9 Hz, 3H). **^{13}C NMR (125 MHz, $CDCl_3$)** δ 210.37 (d, *J* = 6.0 Hz), 133.17 (d, *J* = 7.3 Hz), 131.97 (d, *J* = 2.7 Hz), 131.88 (d, *J* = 2.7 Hz), 131.75 (d, *J* = 104.8 Hz), 131.64 (d, *J* = 9.8 Hz), 131.47 (d, *J* = 9.5 Hz), 131.27 (d, *J* = 102.7 Hz), 128.42, 128.33 (d, *J* = 3.3 Hz), 128.25, 126.09 (d, *J* = 10.4 Hz), 121.69 (d, *J* = 3.4 Hz), 96.76 (d, *J* = 99.4 Hz), 90.37 (d, *J* = 13.7 Hz), 14.62 (d, *J* = 6.4 Hz). **^{31}P NMR** (202 MHz, $CDCl_3$) δ 29.74. **ESI-MS:** calculated [$C_{20}H_{17}OPS$ + H]$^{+}$: 337.0810, found: 337.0816. $[\alpha]^{20}_{D}$ = +345.2 (c = 1.02, CH_2Cl_2). The product was analyzed by HPLC to determine the enantiomeric excess: 86% e.e. (CHIRALPAK IC, hexane/*i*-PrOH = 70/30, detector: 254 nm, T = 25 °C, flow rate: 1 mL/min), t_1(major) = 13.57 min, t_2(minor) = 15.91 min.

(*S*)-hexa-3,4-dien-3-yldiphenylphosphine oxide (3o)

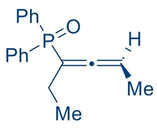

Colorless oil (21.5 mg, 76%); Reaction time: 84 h; **^{1}H NMR (500 MHz, $CDCl_3$)** δ 7.78 – 7.67 (m, 4H), 7.53 – 7.41 (m, 6H), 5.18 – 4.99 (m, 1H), 2.32 – 2.17 (m, 2H), 1.47 (t, *J* = 6.9 Hz, 3H), 1.08 (t, *J* = 7.3 Hz, 3H). **^{13}C NMR (125 MHz, $CDCl_3$)** δ 209.16 (d, *J* = 7.0 Hz), 132.67 (d, *J* = 104.4 Hz), 132.24 (d, *J* = 104.0 Hz), 131.85, 131.77, 131.75 (d, *J* = 10.0 Hz), 131.69, 128.32 (d, *J* = 12.0 Hz), 99.76 (d, *J* = 102.2 Hz), 89.22 (d, *J* = 13.9 Hz), 21.00 (d, *J* = 7.4 Hz), 13.22 (d, *J* = 6.3 Hz), 12.94 (d, *J* = 6.5 Hz). **^{31}P NMR (202 MHz, $CDCl_3$)** δ 30.32. **APCI-MS:** calculated [$C_{18}H_{19}OP$ + H]$^{+}$: 283.1246, found: 283.1248. $[\alpha]^{20}_{D}$ = +54.5 (c = 0.72, CH_2Cl_2). The product was analyzed by HPLC to determine the enantiomeric excess: 94% e.e. (CHIRALPAK AD-H, hexane/*i*-PrOH = 97/3, detector: 220 nm, T = 25 °C, flow rate: 1 mL/min), t_1(major) = 46.36 min, t_2(minor) = 49.31 min.

(*S*)-octa-2,3-dien-4-yldiphenylphosphine oxide (3p)

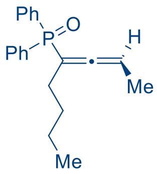

Colorless oil (27.8 mg, 90%); Reaction time: 84 h; **^{1}H NMR (500 MHz, $CDCl_3$)** δ 7.79 – 7.65 (m, 4H), 7.58 – 7.34 (m, 6H), 5.16 – 4.95 (m, 1H), 2.31 – 2.07 (m, 2H), 1.56 – 1.37 (m, 5H), 1.36 – 1.23 (m, 2H), 0.85 (t, *J* = 7.3 Hz, 3H). **^{13}C NMR (125 MHz, $CDCl_3$)** δ 209.26 (d, *J* = 7.0 Hz), 132.62 (d, *J* = 104.3 Hz), 132.19 (d, *J* = 100.8 Hz), 131.79, 131.73, 131.71, 131.64, 131.63, 128.25 (d, *J* = 11.9 Hz), 98.07 (d, *J* = 101.9 Hz), 88.61 (d, *J* = 14.0 Hz), 30.51 (d, *J* = 5.8 Hz), 27.20 (d, *J* = 6.9 Hz), 22.26, 13.93, 13.10 (d, *J* = 6.1 Hz). **^{31}P NMR (202 MHz, $CDCl_3$)** δ 30.63. **APCI-MS:** calculated [$C_{20}H_{23}OP$ + H]$^{+}$: 311.1559, found: 311.1559. $[\alpha]^{20}_{D}$ = +46.2 (c = 1.09, CH_2Cl_2). The product was analyzed by HPLC to determine the enantiomeric excess: 92% e.e. (CHIRALPAK IC-IC, hexane/*i*-PrOH = 90/10, detector: 220 nm, T = 25 °C, flow rate: 0.8 mL/min), t_1(major) = 70.66 min, t_2(minor) = 73.77 min.

(*S*)-diphenyl(1-phenylhexa-3,4-dien-3-yl)phosphine oxide (3q)

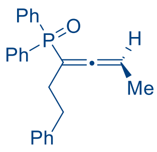

Colorless oil (28.9 mg, 81%); Reaction time: 84 h; **^{1}H NMR (500 MHz, $CDCl_3$)** δ 7.75 – 7.61 (m, 4H), 7.52 – 7.46 (m, 2H), 7.45 – 7.38 (m, 4H), 7.25 – 7.19 (m, 2H), 7.18 – 7.09 (m, 3H), 5.09 – 4.97 (m, 1H), 2.90 – 2.75 (m, 2H), 2.65 – 2.47 (m, 2H), 1.38 (t, *J* = 6.9 Hz, 3H). **^{13}C NMR (125 MHz, $CDCl_3$)** δ 209.53 (d, *J* = 6.7 Hz), 141.21, 132.46 (d, *J* = 104.3 Hz), 132.04 (d, *J* = 103.9 Hz), 131.83, 131.80 (d, *J* = 2.9 Hz), 131.75, 131.72, 131.68, 128.72, 128.36, 128.28 (d, *J* = 4.6 Hz), 126.01, 97.37 (d, *J* = 101.9 Hz), 89.21 (d, *J* = 13.8 Hz), 34.38 (d, *J* = 6.1 Hz), 29.01 (d, *J* = 7.6 Hz), 13.06 (d, *J* = 6.3 Hz). **^{31}P NMR (202 MHz,**

$CDCl_3$) δ 30.13. **APCI-MS:** calculated $[C_{24}H_{23}OP + H]^+$: 359.1559, found: 359.1559. $[\alpha]^{20}_D$ = +23.4 (c = 0.96, CH_2Cl_2). The product was analyzed by HPLC to determine the enantiomeric excess: 90% e.e. (CHIRALPAK IE, hexane/*i*-PrOH = 80/20, detector: 220 nm, T = 25 °C, flow rate: 1 mL/min), t_1(minor) = 36.43 min, t_2(major) = 38.79 min.

(*S*)-octa-4,5-dien-4-yldiphenylphosphine oxide (3r)

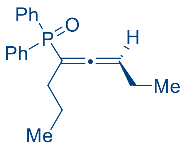

Colorless oil (28.3 mg, 91%); Reaction time: 84 h; **^{1}H NMR (500 MHz, $CDCl_3$)** δ 7.80 – 7.63 (m, 4H), 7.56 – 7.37 (m, 6H), 5.13 (tt, *J* = 9.7, 4.8 Hz, 1H), 2.31 – 2.11 (m, 2H), 1.89 – 1.71 (m, 2H), 1.60 – 1.44 (m, 2H), 0.91 (t, *J* = 7.4 Hz, 3H), 0.75 (t, *J* = 7.4 Hz, 3H). **^{13}C NMR (125 MHz, $CDCl_3$)** δ 208.19 (d, *J* = 7.3 Hz), 132.45 (d, *J* = 104.6 Hz), 132.29 (d, *J* = 103.7 Hz), 131.83 (d, *J* = 3.1 Hz), 131.76 (d, *J* = 3.3 Hz), 131.68 (d, *J* = 2.7 Hz), 128.34 (d, *J* = 10.5 Hz), 128.24 (d, *J* = 10.3 Hz), 98.95 (d, *J* = 102.6 Hz), 95.67 (d, *J* = 13.9 Hz), 29.70 (d, *J* = 7.2 Hz), 21.77 (d, *J* = 5.8 Hz), 21.36 (d, *J* = 5.6 Hz), 13.81, 13.33 (d, *J* = 3.3 Hz). **^{31}P NMR (202 MHz, $CDCl_3$)** δ 30.31. **APCI-MS:** calculated $[C_{20}H_{23}OP + H]^+$: 311.1559, found: 311.1560. $[\alpha]^{20}_D$ = +77.9 (c = 0.81, CH_2Cl_2). The product was analyzed by HPLC to determine the enantiomeric excess: 92% e.e. (CHIRALPAK IC, hexane/*i*-PrOH = 90/10, detector: 220 nm, T = 25 °C, flow rate: 1 mL/min), t_1(major) = 29.29 min, t_2(minor) = 32.07 min.

(*S*)-nona-3,4-dien-5-yldiphenylphosphine oxide (3s)

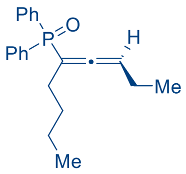

Colorless oil (30.7 mg, 95%); Reaction time: 84 h; **^{1}H NMR (500 MHz, $CDCl_3$)** δ 7.81 – 7.65 (m, 4H), 7.54 – 7.36 (m, 6H), 5.18 – 5.06 (m, 1H), 2.35 – 2.17 (m, 2H), 1.89 – 1.74 (m, 2H), 1.57 – 1.43 (m, 2H), 1.39 – 1.28 (m, 2H), 0.85 (t, *J* = 7.3 Hz, 3H), 0.75 (t, *J* = 7.4 Hz, 3H). **^{13}C NMR (125 MHz, $CDCl_3$)** δ 208.12 (d, *J* = 7.3 Hz), 132.45 (d, *J* = 104.2 Hz), 132.29 (d, *J* = 103.7 Hz), 131.81 (d, *J* = 3.5 Hz), 131.74 (d, *J* = 3.1 Hz), 131.66 (d, *J* = 2.6 Hz), 128.32 (d, *J* = 9.8 Hz), 128.22 (d, *J* = 10.0 Hz), 99.10 (d, *J* = 102.4 Hz), 95.70 (d, *J* = 14.1 Hz), 30.57 (d, *J* = 6.1 Hz), 27.30 (d, *J* = 7.1 Hz), 22.33, 21.35 (d, *J* = 5.7 Hz), 13.95, 13.31 (d, *J* = 3.2 Hz). **^{31}P NMR (202 MHz, $CDCl_3$)** δ 30.34. **APCI-MS:** calculated $[C_{21}H_{25}OP + H]^+$: 325.1716, found: 325.1717. $[\alpha]^{20}_D$ = +74.9 (c = 0.91, CH_2Cl_2). The product was analyzed by HPLC to determine the enantiomeric excess: 90% e.e. (CHIRALPAK IC, hexane/*i*-PrOH = 90/10, detector: 220 nm, T = 25 °C, flow rate: 1 mL/min), t_1(major) = 27.02 min, t_2(minor) = 29.22 min.

(*S*)-bis(4-fluorophenyl)(penta-2,3-dien-2-yl)phosphine oxide (3t)

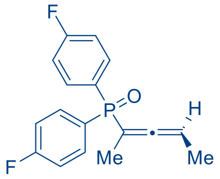

Colorless oil (26.1 mg, 86%); Reaction time: 84 h; **^{1}H NMR (500 MHz, $CDCl_3$)** δ 7.80 – 7.60 (m, 4H), 7.21 – 7.09 (m, 4H), 5.08 – 4.95 (m, 1H), 1.92 (dd, *J* = 12.0, 2.8 Hz, 3H), 1.47 (t, *J* = 7.0 Hz, 3H). **^{13}C NMR (125 MHz, $CDCl_3$)** δ 209.95 (d, *J* = 7.3 Hz), 165.16 (dd, *J* = 253.3, 3.3 Hz), 165.10 (dd, *J* = 253.3, 3.3 Hz), 134.12 (dd, *J* = 19.7, 9.1 Hz), 127.96 (dd, *J* = 107.9, 3.2 Hz), 127.51 (dd, *J* = 107.4, 3.1 Hz), 115.91 (dd, *J* = 13.1, 5.5 Hz), 115.74 (dd, *J* = 13.2, 5.6 Hz), 92.52 (d, *J* = 104.6 Hz), 87.42 (d, *J* = 13.8 Hz), 14.29 (d, *J* = 7.3 Hz), 13.04 (d, *J* = 6.3 Hz). **^{31}P NMR (202 MHz, $CDCl_3$)** δ 29.64. **^{19}F NMR (471 MHz, $CDCl_3$)** δ -106.77, -106.89. **APCI-MS:** calculated $[C_{17}H_{15}F_2OP + H]^+$: 305.0901, found: 305.0903. $[\alpha]^{20}_D$ = +61.8 (c = 0.80, CH_2Cl_2). The product was analyzed by HPLC to determine the enantiomeric excess: 91% e.e. (CHIRALPAK OD-H, hexane/*i*-PrOH = 97/3, detector: 220 nm, T = 25 °C, flow rate: 1 mL/min), t_1(major) = 10.77 min, t_2(minor) = 11.87 min.

(*S*)-bis(4-fluorophenyl)(hexa-3,4-dien-3-yl)phosphine oxide (3u)

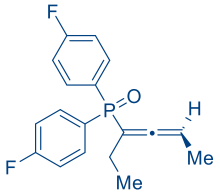

Colorless oil (24.8 mg, 78%); Reaction time: 84 h; **^{1}H NMR (400 MHz, CDCl$_3$)** δ 7.83 – 7.61 (m, 4H), 7.24 – 7.08 (m, 4H), 5.21 – 5.05 (m, 1H), 2.35 – 2.13 (m, 2H), 1.49 (t, *J* = 7.0 Hz, 3H), 1.08 (t, *J* = 7.3 Hz, 3H). **^{13}C NMR (100 MHz, CDCl$_3$)** δ 209.22 (d, *J* = 7.3 Hz), 165.14 (dd, *J* = 253.2, 3.3 Hz), 165.07 (dd, *J* = 253.1, 3.3 Hz), 134.13 (dd, *J* = 17.2, 10.9 Hz), 134.13 (d, *J* = 10.9 Hz), 128.42 (dd, *J* = 107.7, 3.3 Hz), 127.98 (dd, *J* = 107.2, 3.4 Hz), 115.90 (dd, *J* = 13.3, 4.8 Hz), 115.69 (dd, *J* = 13.3, 4.8 Hz), 99.74 (d, *J* = 104.0 Hz), 89.47 (d, *J* = 14.1 Hz), 20.95 (d, *J* = 7.5 Hz), 13.22 (d, *J* = 6.3 Hz), 12.92 (d, *J* = 6.6 Hz). **^{31}P NMR (162 MHz, CDCl$_3$)** δ 28.83. **^{19}F NMR (471 MHz, CDCl$_3$)** δ -106.87, -107.00. **APCI-MS:** calculated [$C_{18}H_{17}F_2OP$ + H]$^+$: 319.1058, found: 319.1060. $[\alpha]^{20}_{D}$ = +40.3 (c = 0.83, CH_2Cl_2). The product was analyzed by HPLC to determine the enantiomeric excess: 92% e.e. (CHIRALPAK IG, hexane/*i*-PrOH = 95/5, detector: 220 nm, T = 25 °C, flow rate: 1 mL/min), t_1(minor) = 57.93 min, t_2(major) = 61.15 min.

(*S*)-bis(4-chlorophenyl)(hexa-3,4-dien-3-yl)phosphine oxide (3v)

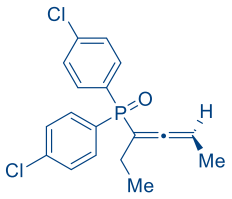

Colorless oil (18.0 mg, 51%); Reaction time: 84 h; **^{1}H NMR (500 MHz, CDCl$_3$)** δ 7.73 – 7.58 (m, 4H), 7.50 – 7.36 (m, 4H), 5.22 – 5.07 (m, 1H), 2.29 – 2.15 (m, 2H), 1.50 (t, *J* = 7.0 Hz, 3H), 1.08 (t, *J* = 7.3 Hz, 3H). **^{13}C NMR (125 MHz, CDCl$_3$)** δ 209.27 (d, *J* = 7.4 Hz), 138.63 (d, *J* = 3.5 Hz), 138.54 (d, *J* = 3.2 Hz), 133.10 (d, *J* = 10.6 Hz), 133.01 (d, *J* = 10.8 Hz), 130.85 (d, *J* = 105.7 Hz), 130.44 (d, *J* = 105.4 Hz), 128.87 (d, *J* = 3.3 Hz), 128.77 (d, *J* = 3.4 Hz), 99.34 (d, *J* = 104.3 Hz), 89.70 (d, *J* = 14.2 Hz), 20.98 (d, *J* = 7.4 Hz), 13.23 (d, *J* = 6.3 Hz), 12.93 (d, *J* = 6.5 Hz). **^{31}P NMR (202 MHz, CDCl$_3$)** δ 28.69. **APCI-MS:** calculated [$C_{18}H_{17}Cl_2OP$ + H]$^+$: 351.0467, found: 351.0471. $[\alpha]^{20}_{D}$ = +45.2 (c = 0.60, CH_2Cl_2). The product was analyzed by HPLC to determine the enantiomeric excess: 91% e.e. (CHIRALPAK IC, hexane/*i*-PrOH = 90/10, detector: 220 nm, T = 25 °C, flow rate: 1 mL/min), t_1(major) = 22.86 min, t_2(minor) = 24.31 min.

(*S*)-di([1,1'-biphenyl]-4-yl)(hexa-3,4-dien-3-yl)phosphine oxide (3w)

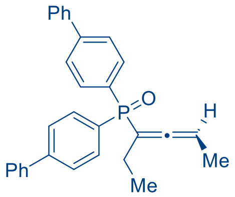

Colorless oil (37.2 mg, 85%); Reaction time: 120 h; **^{1}H NMR (500 MHz, CDCl$_3$)** δ 7.87 – 7.77 (m, 4H), 7.72 – 7.66 (m, 4H), 7.64 – 7.58 (m, 4H), 7.49 – 7.43 (m, 4H), 7.41 – 7.35 (m, 2H), 5.21 – 5.08 (m, 1H), 2.38 – 2.24 (m, 2H), 1.52 (t, *J* = 6.9 Hz, 3H), 1.12 (t, *J* = 7.3 Hz, 3H). **^{13}C NMR (125 MHz, CDCl$_3$)** δ 209.16 (d, *J* = 7.2 Hz), 144.56 (d, *J* = 2.7 Hz), 144.49 (d, *J* = 2.7 Hz), 140.14, 132.31 (d, *J* = 10.3 Hz), 132.22 (d, *J* = 10.8 Hz), 131.33 (d, *J* = 105.6 Hz), 130.87 (d, *J* = 105.4 Hz), 129.05, 128.19, 127.36, 127.03 (d, *J* = 12.4 Hz), 99.82 (d, *J* = 102.7 Hz), 89.38 (d, *J* = 13.9 Hz), 21.08 (d, *J* = 7.4 Hz), 13.32 (d, *J* = 6.3 Hz), 12.98 (d, *J* = 6.6 Hz). **^{31}P NMR (202 MHz, CDCl$_3$)** δ 30.02. **APCI-MS:** calculated [$C_{30}H_{27}OP$ + H]$^+$: 435.1872, found: 435.1882. $[\alpha]^{20}_{D}$ = +26.0 (c = 0.80, CH_2Cl_2). The product was analyzed by HPLC to determine the enantiomeric excess: 90% e.e. (CHIRALPAK IG, hexane/*i*-PrOH = 70/30, detector: 270 nm, T = 25 °C, flow rate: 1 mL/min), t_1(minor) = 47.83 min, t_2(major) = 52.22 min.

(*S*)-hexa-3,4-dien-3-yldi-p-tolylphosphine oxide (3x)

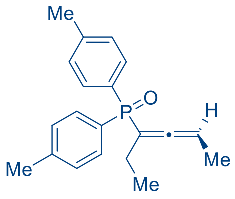

Colorless oil (31.2 mg, 93%); Reaction time: 84 h; **^{1}H NMR (400 MHz, $CDCl_3$)** δ 7.65 – 7.53 (m, 4H), 7.27 – 7.15 (m, 4H), 5.15 – 5.01 (m, 1H), 2.39 (s, 3H), 2.38 (s, 3H), 2.29 – 2.18 (m, 2H), 1.49 (t, *J* = 6.9 Hz, 3H), 1.07 (t, *J* = 7.3 Hz, 3H). **^{13}C NMR (100 MHz, $CDCl_3$)** δ 208.92 (d, *J* = 7.1 Hz), 142.04 (d, *J* = 2.8 Hz), 141.96 (d, *J* = 2.9 Hz), 131.78 (d, *J* = 9.0 Hz), 131.69 (d, *J* = 9.0 Hz), 129.58 (d, *J* = 112.2 Hz), 129.14 (d, *J* = 106.3 Hz), 129.01 (d, *J* = 12.5 Hz), 99.96 (d, *J* = 102.1 Hz), 88.97 (d, *J* = 13.8 Hz), 21.69, 21.00 (d, *J* = 7.5 Hz), 13.30 (d, *J* = 6.1 Hz), 12.92 (d, *J* = 6.6 Hz). **^{31}P NMR (162 MHz, $CDCl_3$)** δ 30.41. **APCI-MS:** calculated $[C_{20}H_{23}OP + H]^+$: 311.1559, found: 311.1562. $[\alpha]^{20}_D$ = +32.1 (c = 0.78, CH_2Cl_2). The product was analyzed by HPLC to determine the enantiomeric excess: 88% e.e. (CHIRALPAK IC, hexane/*i*-PrOH = 80/20, detector: 220 nm, T = 25 °C, flow rate: 1 mL/min), t_1(major) = 23.92 min, t_2(minor) = 25.75 min.

(*S*)-hexa-3,4-dien-3-ylbis(4-methoxyphenyl)phosphine oxide (3y)

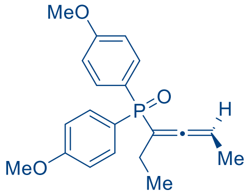

Colorless oil (26.7 mg, 78%); Reaction time: 120 h; **^{1}H NMR (500 MHz, $CDCl_3$)** δ 7.66 – 7.58 (m, 4H), 6.97 – 6.92 (m, 4H), 5.13 – 5.05 (m, 1H), 3.84 (s, 3H), 3.83 (s, 3H), 2.26 – 2.17 (m, 2H), 1.50 (t, *J* = 6.9 Hz, 3H), 1.07 (t, *J* = 7.3 Hz, 3H). **^{13}C NMR (125 MHz, $CDCl_3$)** δ 208.90 (d, *J* = 7.2 Hz), 162.32 (d, *J* = 2.9 Hz), 162.27 (d, *J* = 2.9 Hz), 133.55 (d, *J* = 9.5 Hz), 133.46 (d, *J* = 9.5 Hz), 124.18 (d, *J* = 111.0 Hz), 123.70 (d, *J* = 110.7 Hz), 113.83 (d, *J* = 12.6 Hz), 100.25 (d, *J* = 102.9 Hz), 88.85 (d, *J* = 13.8 Hz), 55.39, 20.99 (d, *J* = 7.6 Hz), 13.33 (d, *J* = 6.3 Hz), 12.92 (d, *J* = 6.5 Hz). **^{31}P NMR (202 MHz, $CDCl_3$)** δ 30.22. **APCI-MS:** calculated $[C_{20}H_{23}O_3P + H]^+$: 343.1458, found: 343.1461. $[\alpha]^{20}_D$ = +41.7 (c = 0.89, CH_2Cl_2). The product was analyzed by HPLC to determine the enantiomeric excess: 90% e.e. (CHIRALPAK IE, hexane/*i*-PrOH = 70/30, detector: 241 nm, T = 25 °C, flow rate: 1 mL/min), t_1(minor) = 43.11 min, t_2(major) = 46.11 min.

(*S*)-bis(3-fluorophenyl)(hexa-3,4-dien-3-yl)phosphine oxide (3z)

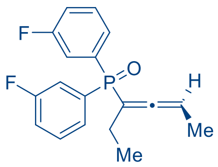

Colorless oil (26.5 mg, 83%); Reaction time: 84 h; **^{1}H NMR (500 MHz, $CDCl_3$)** δ 7.57 – 7.34 (m, 6H), 7.26 – 7.17 (m, 2H), 5.31 – 5.09 (m, 1H), 2.35 – 2.15 (m, 2H), 1.51 (t, *J* = 7.0 Hz, 3H), 1.10 (t, *J* = 7.3 Hz, 3H). **^{13}C NMR (125 MHz, $CDCl_3$)** δ 209.34 (d, *J* = 7.4 Hz), 162.61 (d, *J* = 250.2 Hz), 162.47 (d, *J* = 250.0 Hz), 134.89 (dd, *J* = 103.7, 5.5 Hz), 134.50 (dd, *J* = 103.2, 5.4 Hz), 130.46 (d, *J* = 7.3 Hz), 130.35 (d, *J* = 7.2 Hz), 127.43 (dd, *J* = 9.0, 3.3 Hz), 127.33 (dd, *J* = 9.1, 3.4 Hz), 119.26 (dd, *J* = 14.1, 2.2 Hz), 119.09 (dd, *J* = 14.1, 2.2 Hz), 118.57 (dd, *J* = 21.3, 11.0 Hz), 118.48 (dd, *J* = 21.4, 11.0 Hz), 99.06 (d, *J* = 104.4 Hz), 89.84 (d, *J* = 14.4 Hz), 20.97 (d, *J* = 7.7 Hz), 13.12 (d, *J* = 6.3 Hz), 12.89 (d, *J* = 6.8 Hz). **^{31}P NMR (202 MHz, $CDCl_3$)** δ 28.18 (t, *J* = 4.8 Hz). **^{19}F NMR (471 MHz, CDCl3)** δ -111.32 (d, J = 5.3 Hz), -111.39 (d, J = 5.5 Hz). **APCI-MS:** calculated $[C_{18}H_{17}F_2OP + H]^+$: 319.1058, found: 319.1061. $[\alpha]^{20}_D$ = +43.6 (c = 0.88, CH_2Cl_2). The product was analyzed by HPLC to determine the enantiomeric excess: 91% e.e. (CHIRALPAK IC, hexane/*i*-PrOH = 90/10, detector: 220 nm, T = 25 °C, flow rate: 1 mL/min), t_1(major) = 23.83 min, t_2(minor) = 25.49 min.

(*S*)-bis(3-chlorophenyl)(hexa-3,4-dien-3-yl)phosphine oxide (3aa)

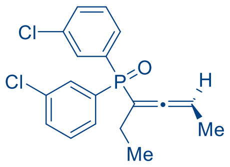

Colorless oil (25.4 mg, 73%); Reaction time: 84 h; **^{1}H NMR (500 MHz, $CDCl_3$)** δ 7.74 – 7.67 (m, 2H), 7.64 – 7.58 (m, 1H), 7.58 – 7.48 (m, 3H), 7.45 – 7.38 (m, 2H), 5.36 – 5.08 (m, 1H), 2.41 – 2.02 (m, 2H), 1.53 (t, *J* = 7.1 Hz, 3H), 1.10 (t, *J* = 7.3 Hz, 3H). **^{13}C NMR (100 MHz, $CDCl_3$)** δ 209.45 (d, *J* = 7.4 Hz), 135.06 (d, *J* = 2.3 Hz), 134.91 (d, *J* = 2.4 Hz), 134.46 (d, *J* = 102.6 Hz), 133.98 (d, *J* = 102.1 Hz), 132.30 (d, *J* = 2.6 Hz), 132.19 (d, *J* = 2.6 Hz), 131.62 (d, *J* = 6.5 Hz), 131.51 (d, *J* = 6.5 Hz), 130.00 (d, *J* = 1.9 Hz), 129.87 (d, *J* = 1.9 Hz), 129.79 (d, *J* = 9.1 Hz), 129.61 (d, *J* = 9.1 Hz), 98.98 (d, *J* = 104.4 Hz), 90.05 (d, *J* = 14.4 Hz), 20.98 (d, *J* = 7.6 Hz), 13.18 (d, *J* = 6.3 Hz), 12.94 (d, *J* = 6.7 Hz). **^{31}P NMR (202 MHz, $CDCl_3$)** δ 28.00. **APCI-MS:** calculated $[C_{18}H_{17}Cl_2OP + H]^+$: 351.0467, found: 351.0470. $[\alpha]^{20}_{D}$ = +34.9 (c = 0.50, CH_2Cl_2). The product was analyzed by HPLC to determine the enantiomeric excess: 86% e.e. (CHIRALPAK AD-H, hexane/*i*-PrOH = 90/10, detector: 220 nm, T = 25 °C, flow rate: 1 mL/min), t_1(major) = 8.45 min, t_2(minor) = 9.38 min.

(*S*)-hexa-3,4-dien-3-ylbis(3-methoxyphenyl)phosphine oxide (3ab)

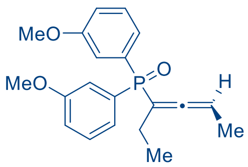

Colorless oil (25.7 mg, 75%); Reaction time: 84 h; **^{1}H NMR (400 MHz, $CDCl_3$)** δ 7.36 – 7.32 (m, 3H), 7.31 – 7.26 (m, 1H), 7.26 – 7.17 (m, 2H), 7.08 – 6.98 (m, 2H), 5.17 – 5.04 (m, 1H), 3.82 (s, 3H), 3.82 (s, 3H), 2.34 – 2.18 (m, 2H), 1.49 (t, *J* = 6.9 Hz, 3H), 1.08 (t, *J* = 7.3 Hz, 3H). **^{13}C NMR (100 MHz, $CDCl_3$)** δ 209.13 (d, *J* = 7.2 Hz), 159.67 (d, *J* = 1.6 Hz), 159.52 (d, *J* = 1.6 Hz), 134.00 (d, *J* = 103.5 Hz), 133.60 (d, *J* = 103.2 Hz), 129.51 (d, *J* = 4.7 Hz), 129.37 (d, *J* = 4.7 Hz), 124.05 (d, *J* = 4.9 Hz), 123.95 (d, *J* = 4.9 Hz), 118.14 (d, *J* = 2.7 Hz), 118.07 (d, *J* = 2.7 Hz), 116.56 (d, *J* = 8.0 Hz), 116.46 (d, *J* = 8.1 Hz), 99.67 (d, *J* = 102.7 Hz), 89.25 (d, *J* = 14.0 Hz), 55.57, 21.06 (d, *J* = 7.5 Hz), 13.26 (d, *J* = 6.2 Hz), 12.95 (d, *J* = 6.6 Hz). **^{31}P NMR (162 MHz, $CDCl_3$)** δ 30.54. **APCI-MS:** calculated $[C_{20}H_{23}O_3P + H]^+$: 343.1458, found: 343.1461. $[\alpha]^{20}_{D}$ = +22.5 (c = 0.85, CH_2Cl_2). The product was analyzed by HPLC to determine the enantiomeric excess: 90% e.e. (CHIRALPAK IE, hexane/*i*-PrOH = 70/30, detector: 220 nm, T = 25 °C, flow rate: 1 mL/min), t_1(minor) = 27.9 min, t_2(major) = 30.8 min.

(*S*)-bis(2,2-difluorobenzo[d][1,3]dioxol-5-yl)(hexa-3,4-dien-3-yl)phosphine oxide (3ac)

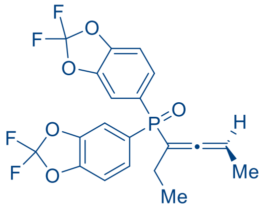

Colorless oil (29.6 mg, 67%); Reaction time: 84 h; **^{1}H NMR (500 MHz, $CDCl_3$)** δ 7.53 – 7.45 (m, 2H), 7.44 – 7.35 (m, 2H), 7.20 – 7.15 (m, 2H), 5.49 – 4.99 (m, 1H), 2.40 – 2.05 (m, 2H), 1.55 (t, *J* = 7.1 Hz, 3H), 1.10 (t, *J* = 7.3 Hz, 3H). **^{13}C NMR (125 MHz, $CDCl_3$)** δ 209.42 (d, *J* = 7.4 Hz), 146.54 (d, *J* = 2.9 Hz), 146.46 (d, *J* = 2.7 Hz), 144.09 (d, *J* = 2.9 Hz), 143.94 (d, *J* = 2.9 Hz), 131.66 (t, *J* = 257.5 Hz), 128.44 (d, *J* = 107.0 Hz), 128.34 (d, *J* = 13.5 Hz), 128.26 (d, *J* = 13.7 Hz), 128.00 (d, *J* = 106.2 Hz), 112.52 (d, *J* = 12.0 Hz), 112.45 (d, *J* = 12.0 Hz), 109.84 (d, *J* = 14.5 Hz), 99.25 (d, *J* = 105.7 Hz), 89.97 (d, *J* = 14.5 Hz), 21.07 (d, *J* = 7.5 Hz), 13.24 (d, *J* = 6.3 Hz), 12.93 (d, *J* = 6.6 Hz). **^{31}P NMR (202 MHz, $CDCl_3$)** δ 28.40. **^{19}F NMR (470 MHz, $CDCl_3$)** δ -49.59, -49.60, -49.67, -49.72. **APCI-MS:** calculated $[C_{20}H_{15}F_4O_5P + H]^+$: 443.0666, found: 443.0670. $[\alpha]^{20}_{D}$ = +36.6 (c = 0.53, CH_2Cl_2). The product was analyzed by HPLC to determine the enantiomeric excess: 87% e.e. (CHIRALPAK OD-H, hexane/*i*-PrOH = 99/1, detector: 220 nm, T = 25 °C, flow rate: 1 mL/min), t_1(minor) = 20.33 min, t_2(major) = 22.33 min.

(*E*)-penta-1,3-dien-3-yldiphenylphosphine oxide (4a)

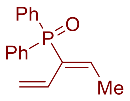

Colorless oil, (20.8 mg, 78%); Reaction time: 24 h; **^{1}H NMR (500 MHz, $CDCl_3$)** δ 7.73 – 7.67 (m, 4H), 7.55 – 7.50 (m, 2H), 7.49 – 7.42 (m, 4H), 6.51 (m, 1H), 6.23 (dq, *J* = 21.0, 7.0 Hz, 1H), 5.52 (d, *J* = 17.9 Hz, 1H), 5.33 (d, *J* = 11.5, 1H), 1.93 (dd, *J* = 7.0, 3.0 Hz, 3H). **^{13}C NMR (125 MHz, $CDCl_3$)** δ 143.30 (d, *J* = 10.2 Hz), 133.13 (d, *J* = 98.9 Hz), 132.61, 132.03 (d, *J* = 9.5 Hz), 131.78 (d, *J* = 2.0 Hz), 129.51 (d, *J* = 10.2 Hz), 128.49 (d, *J* = 12.0 Hz), 121.67 (d, *J* = 6.7 Hz), 15.21 (d, *J* = 14.9 Hz). **^{31}P NMR (202 MHz, $CDCl_3$)** δ 30.75. **ESI-MS:** calculated $[C_{17}H_{17}OP + H]^+$: 269.1090, found: 269.1093.

(*E*)-diphenyl(1-phenylbuta-1,3-dien-2-yl)phosphine oxide (4b)

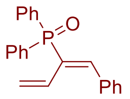

White solid, (28.1 mg, 85%); Reaction time: 24 h; **^{1}H NMR (600 MHz, $CDCl_3$)** δ 7.81 – 7.72 (m, 4H), 7.55 – 7.50 (m, 2H), 7.50 – 7.43 (m, 4H), 7.37 – 7.27 (m, 5H), 6.94 (d, *J* = 21.3 Hz, 1H), 6.71 (m, 1H), 5.69 (d, *J* = 18.0 Hz, 1H), 5.32 (d, *J* = 11.5 Hz, 1H). **^{13}C NMR (150 MHz, $CDCl_3$)** δ 144.00 (d, *J* = 10.9 Hz), 135.33 (d, *J* = 17.6 Hz), 132.48 (d, *J* = 97.5 Hz), 132.18 (d, *J* = 9.3 Hz), 131.99 (d, *J* = 1.8 Hz), 131.98 (d, *J* = 103.8 Hz), 131.08 (d, *J* = 8.3 Hz), 130.02, 128.99, 128.63 (d, *J* = 12.3 Hz), 128.43, 122.68 (d, *J* = 5.6 Hz). **^{31}P NMR (243 MHz, $CDCl_3$)** δ 32.95. **ESI-MS:** calculated $[C_{22}H_{19}OP + H]^+$: 331.1246, found: 331.1250.

(*E*)-(1-(4-chlorophenyl)buta-1,3-dien-2-yl)diphenylphosphine oxide (4c)

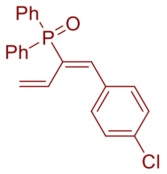

Colorless oil, (29.6 mg, 81%); Reaction time: 24 h; **^{1}H NMR (500 MHz, $CDCl_3$)** δ 7.81 – 7.72 (m, 4H), 7.59 – 7.53 (m, 2H), 7.52 – 7.45 (m, 4H), 7.35 – 7.29 (m, 4H), 6.94 (d, *J* = 21.0 Hz, 1H), 6.64 (m, 1H), 5.67 (d, *J* = 18.0 Hz, 1H), 5.36 (d, *J* = 11.6 Hz, 1H). **^{13}C NMR (150 MHz, $CDCl_3$)** δ 142.45 (d, *J* = 10.9 Hz), 134.95, 133.83, 133.71, 133.27 (d, *J* = 95.3 Hz), 132.24, 132.17, 132.12, 131.78 (d, *J* = 103.7 Hz), 131.34, 130.82 (d, *J* = 7.8 Hz), 128.73 (d, *J* = 3.5 Hz), 128.66, 123.24 (d, *J* = 5.5 Hz). **^{31}P NMR (202 MHz, $CDCl_3$)** δ 31.68. **ESI-MS: calculated** $[C_{22}H_{28}ClOP + H]^+$: 365.0857, found: 365.0859.

(*E*)-diphenyl(1-(p-tolyl)buta-1,3-dien-2-yl)phosphine oxide (4d)

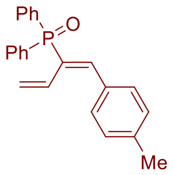

Colorless oil, (32.0 mg, 93%); Reaction time: 24 h; **^{1}H NMR (500 MHz, $CDCl_3$)** δ 7.82 – 7.73 (m, 4H), 7.58 – 7.52 (m, 2H), 7.50 – 7.44 (m, 4H), 7.31 – 7.25 (m, 2H), 7.21 – 7.14 (m, 2H), 6.93 (d, *J* = 21.3 Hz, 1H), 6.73 (m, 1H), 5.67 (d, *J* = 18.0 Hz, 1H), 5.32 (d, *J* = 11.6 Hz, 1H), 2.35 (s, 3H). **^{13}C NMR (150 MHz, $CDCl_3$)** δ 144.06 (d, *J* = 11.1 Hz), 139.30, 132.28 (d, *J* = 99.1 Hz), 132.23 (d, *J* = 9.7 Hz), 132.13 (d, *J* = 103.1 Hz), 131.31 (d, *J* = 8.1 Hz), 131.16, 130.13, 129.17, 128.66, 128.58, 122.32 (d, *J* = 5.5 Hz), 21.51. **^{31}P NMR (243 MHz, $CDCl_3$)** δ 33.08. **ESI-MS:** calculated $[C_{23}H_{21}OP + H]^+$: 345.1403, found: 345.1408.

(*E*)-(1-(3-chlorophenyl)buta-1,3-dien-2-yl)diphenylphosphine oxide (4e)

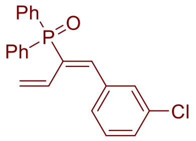

Colorless oil, (27.6 mg, 76%); Reaction time: 24 h; **^{1}H NMR (500 MHz, $CDCl_3$)** δ 7.81 – 7.74 (m, 4H), 7.59 – 7.53 (m, 2H), 7.52 – 7.46 (m, 4H), 7.36 – 7.33 (m, 1H), 7.35 – 7.21 (m, 4H), 6.90 (d, *J* = 20.9 Hz, 1H), 6.65 (m, 1H), 5.70 (d, *J* = 18.0 Hz, 1H), 5.38 (d, *J* = 11.5 Hz, 1H). **^{13}C NMR (150 MHz, $CDCl_3$)** δ 142.17 (d, *J* = 11.4 Hz), 137.10 (d, *J* = 18.3 Hz), 134.41, 134.17 (d, *J* = 94.8 Hz), 132.22, 132.16, 131.70 (d, *J* = 103.9 Hz), 130.60 (d, *J* = 7.7 Hz), 129.74 (d, *J* = 5.4 Hz), 128.94, 128.77, 128.69, 128.14, 123.59 (d, *J* = 5.6 Hz). **^{31}P NMR (202 MHz, $CDCl_3$)** δ 31.69. **ESI-MS:** calculated $[C_{22}H_{28}ClOP +$

H]$^+$: 365.0857, found: 365.0862.

(*E*)-(1-(3-methoxyphenyl)buta-1,3-dien-2-yl)diphenylphosphine oxide (4f)

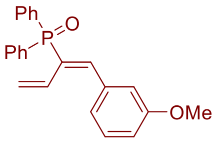

Colorless oil, (25.1 mg, 70%); Reaction time: 24 h; **^{1}H NMR (600 MHz, CDCl$_3$)** δ 7.79 – 7.72 (m, 4H), 7.55 – 7.51 (m, 2H), 7.49 – 7.44 (m, 4H), 7.27 – 7.23 (m, 2H), 6.96 – 6.88 (m, 3H), 6.88 – 6.82 (m, 1H), 6.71 (m, 1H), 5.67 (d, *J* = 18.0 Hz, 1H), 5.32 (dd, *J* = 11.6, 1.0 Hz, 1H), 3.77 (s, 3H). **^{13}C NMR (150 MHz, CDCl$_3$)** δ 159.44, 143.86 (d, *J* = 11.3 Hz), 136.64 (d, *J* = 18.2 Hz), 132.77 (d, *J* = 95.5 Hz), 132.19 (d, *J* = 9.7 Hz), 132.01 (d, *J* = 2.0 Hz), 131.94 (d, *J* = 103.7 Hz), 131.14 (d, *J* = 7.9 Hz), 129.46, 128.64 (d, *J* = 12.0 Hz), 122.68 (d, *J* = 5.5 Hz), 122.51, 115.29, 114.62, 55.40. **^{31}P NMR (243 MHz, CDCl$_3$)** δ 32.86. **ESI-MS:** calculated [$C_{23}H_{21}OP$ + H]$^+$: 361.1352, found: 361.1354.

(*E*)-diphenyl(1-(thiophen-3-yl)buta-1,3-dien-2-yl)phosphine oxide (4g)

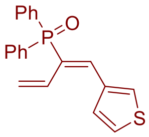

Colorless oil, (21.0 mg, 63%); Reaction time: 18 h; **^{1}H NMR (400 MHz, CDCl$_3$)** δ 7.81 – 7.70 (m, 4H), 7.60 – 7.38 (m, 7H), 7.36 – 7.28 (m, 1H), 7.26 – 7.17 (m, 1H), 6.99 (d, *J* = 20.8 Hz, 1H), 6.87 – 6.63 (m, 1H), 5.67 – 5.54 (m, 1H), 5.43 – 5.31 (m, 1H). **^{13}C NMR (100 MHz, CDCl$_3$)** δ 137.24 (d, *J* = 11.5 Hz), 137.01 (d, *J* = 18.7 Hz), 132.23 (d, *J* = 9.7 Hz), 132.03 (d, *J* = 110.4 Hz), 131.95 (d, *J* = 2.7 Hz), 131.56 (d, *J* = 1.3 Hz), 130.95 (d, *J* = 97.1 Hz), 128.91, 128.58 (d, *J* = 12.1 Hz), 128.20, 125.85, 122.55 (d, *J* = 5.9 Hz). **^{31}P NMR (162 MHz, CDCl$_3$)** δ 31.69. **ESI-MS:** calculated [$C_{20}H_{17}OPS$ + H] $^+$: 337.0810, found: 337.0817.

((1*E*,3*E*)-1,4-diphenylbuta-1,3-dien-2-yl)diphenylphosphine oxide (4h)

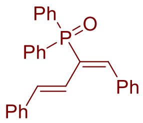

White solid, (25.9 mg, 64%); Reaction time: 18 h; **^{1}H NMR (500 MHz, CDCl$_3$)** δ 7.84 – 7.77 (m, 4H), 7.56 – 7.51 (m, 2H), 7.50 – 7.45 (m, 4H), 7.42 – 7.32 (m, 5H), 7.25 – 7.17 (m, 5H), 7.17 – 7.12 (m, 2H), 6.96 (d, *J* = 21.0 Hz, 1H). **^{13}C NMR (125 MHz, CDCl$_3$)** δ 143.81 (d, *J* = 11.7 Hz), 137.21 (d, *J* = 1.2 Hz), 136.26 (d, *J* = 5.3 Hz), 135.80, 135.66, 132.25 (d, *J* = 9.5 Hz), 132.16 (d, *J* = 103.5 Hz), 132.06 (d, *J* = 2.5 Hz), 131.79, 130.12, 128.75, 128.65 (d, *J* = 1.6 Hz), 128.60, 128.55 (d, *J* = 110.0 Hz), 126.78, 123.33 (d, *J* = 7.8 Hz). **^{31}P NMR (202 MHz, CDCl$_3$)** δ 32.89. **ESI-MS:** calculated [$C_{28}H_{23}OP$ + Na]$^+$: 429.1379, found: 429.1383.

(*E*)-bis(4-methoxyphenyl)(penta-1,3-dien-3-yl)phosphine oxide (4i)

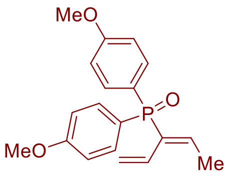

Colorless oil, (14.8 mg, 45%); Reaction time: 48 h; **^{1}H NMR (500 MHz, CDCl$_3$)** δ 7.63 – 7.57 (m, 4H), 6.99 – 6.93 (m, 4H), 6.49 (m, 1H), 6.20 (dq, *J* = 20.8, 6.9 Hz, 1H), 5.51 (d, *J* = 17.8 Hz, 1H), 5.32 (d, *J* = 11.4 Hz, 1H), 3.84 (s, 6H), 1.91 (dd, *J* = 6.9, 2.8 Hz, 3H). **^{13}C NMR (150 MHz, CDCl$_3$)** δ 162.28 (d, *J* = 2.3 Hz), 142.75 (d, *J* = 10.1 Hz), 133.84 (d, *J* = 10.8 Hz), 133.71 (d, *J* = 99.7 Hz), 129.71 (d, *J* = 10.2 Hz), 123.70 (d, *J* = 109.4 Hz), 121.45 (d, *J* = 6.9 Hz), 114.03 (d, *J* = 13.0 Hz), 55.41, 15.19 (d, *J* = 14.7 Hz). **^{31}P NMR (202 MHz, CDCl$_3$)** δ 30.36. **ESI-MS:** calculated [$C_{19}H_{21}O_3P$ + H]$^+$: 329.1301, found: 329.1309.

(*E*)-bis(3-methoxyphenyl)(penta-1,3-dien-3-yl)phosphine oxide (4j)

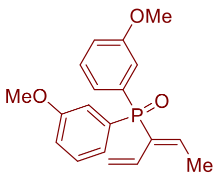

Colorless oil, (17.3 mg, 53%); Reaction time: 48 h; **^{1}H NMR (500 MHz, CDCl$_3$)** δ 7.38 – 7.33 (m, 2H), 7.32 – 7.31 (m, 1H), 7.30 – 7.28 (m, 1H), 7.22 – 7.16 (m, 2H), 7.07 – 7.03 (m, 2H), 6.51 (m, 1H), 6.19 (dq, *J* = 21.1, 7.0 Hz, 1H), 5.56 (d, *J* = 17.9 Hz, 1H), 5.35 (d, *J* = 11.5 Hz, 1H), 3.81 (s, 6H), 1.92 (dd, *J* = 7.0, 3.0 Hz, 3H). **^{13}C NMR (150 MHz, CDCl$_3$)** δ 159.62 (d, *J* = 14.5 Hz), 143.39 (d, *J* = 10.5 Hz), 133.41 (d, *J* = 102.4 Hz), 132.95 (d, *J* = 99.5 Hz), 129.68 (d, *J* = 14.4 Hz), 129.45 (d, *J* = 10.1 Hz), 124.26 (d, *J* = 9.6 Hz), 121.68 (d, *J* = 6.4 Hz), 118.08 (d, *J* = 2.1 Hz), 116.66 (d, *J* = 10.4 Hz), 55.54, 15.25 (d, *J* = 15.3 Hz). **^{31}P NMR (202 MHz, CDCl$_3$)** δ 31.18. **ESI-MS:** calculated [$C_{19}H_{21}O_3P$ + H]$^+$: 329.1301, found: 329.1311.

(*E*)-di([1,1'-biphenyl]-4-yl)(1-phenylbuta-1,3-dien-2-yl)phosphine oxide (4k)

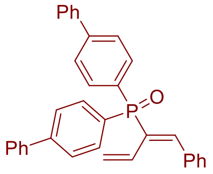

White solid, (39.4 mg, 82%); Reaction time: 48 h; **^{1}H NMR (600 MHz, CDCl$_3$)** δ 7.91 – 7.80 (m, 4H), 7.75 – 7.69 (m, 4H), 7.64 – 7.61 (m, 4H), 7.48 – 7.44 (m, 4H), 7.43 – 7.35 (m, 6H), 7.34 – 7.30 (m, 1H), 7.07 (d, *J* = 21.3 Hz, 1H), 6.78 (m, 1H), 5.79 (d, *J* = 18.1 Hz, 1H), 5.39 (d, *J* = 11.6 Hz, 1H). **^{13}C NMR (150 MHz, CDCl$_3$)** δ 144.73, 144.05 (d, *J* = 11.2 Hz), 139.99, 135.45, 135.32, 132.73 (d, *J* = 9.8 Hz), 132.56 (d, *J* = 96.1 Hz), 131.15 (d, *J* = 8.4 Hz), 130.67 (d, *J* = 104.8 Hz), 130.11, 129.07, 128.48, 128.27, 127.39, 127.31, 122.83 (d, *J* = 5.3 Hz). **^{31}P NMR (243 MHz, CDCl$_3$)** δ 32.69. **ESI-MS:** calculated [$C_{34}H_{27}OP$ + H]$^+$: 483.1872, found: 483.1887.

(*E*)-(1-phenylbuta-1,3-dien-2-yl)di-p-tolylphosphine oxide (4l)

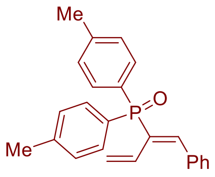

Colorless oil, (32.5 mg, 91%); Reaction time: 48 h; **^{1}H NMR (600 MHz, CDCl$_3$)** δ 7.66 – 7.59 (m, 4H), 7.37 – 7.32 (m, 4H), 7.31 – 7.29 (m, 1H), 7.28 – 7.25 (m, 4H), 6.93 (d, *J* = 21.2 Hz, 1H), 6.69 (m, 1H), 5.69 (m, 1H), 5.31 (m, 1H), 2.39 (s, 6H). **^{13}C NMR (150 MHz, CDCl$_3$)** δ 143.65 (d, *J* = 11.3 Hz), 142.36 (d, *J* = 2.2 Hz), 135.52 (d, *J* = 17.7 Hz), 132.96 (d, *J* = 95.9 Hz), 132.20 (d, *J* = 10.0 Hz), 131.20 (d, *J* = 7.9 Hz), 130.03, 129.37 (d, *J* = 12.2 Hz), 128.92 (d, *J* = 106.2 Hz), 128.86, 128.40, 122.55 (d, *J* = 5.4 Hz), 21.76. **^{31}P NMR (243 MHz, CDCl$_3$)** δ 33.11. **ESI-MS:** calculated [$C_{24}H_{23}OP$ + H]$^+$: 359.15559, found: 359.1565.

(*E*)-bis(4-methoxyphenyl)(1-phenylbuta-1,3-dien-2-yl)phosphine oxide (4m)

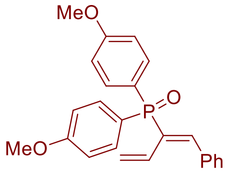

Colorless oil, (28.5 mg, 73%); Reaction time: 48 h; **^{1}H NMR (600 MHz, CDCl$_3$)** δ 7.69 – 7.63 (m, 4H), 7.37 – 7.31 (m, 4H), 7.30 – 7.26 (m, 1H), 6.98 – 6.95 (m, 4H), 6.92 (d, *J* = 21.2 Hz, 1H), 6.69 (m, 1H), 5.67 (d, *J* = 18.0 Hz, 1H), 5.31 (d, *J* = 11.6 Hz, 1H), 3.83 (s, 6H). **^{13}C NMR (150 MHz, CDCl$_3$)** δ 162.40, 143.52 (d, *J* = 11.2 Hz), 135.51 (d, *J* = 17.6 Hz), 133.99 (d, *J* = 11.0 Hz), 133.20 (d, *J* = 96.7 Hz), 131.25 (d, *J* = 8.4 Hz), 130.01, 128.83, 128.39, 123.48 (d, *J* = 110.1 Hz), 122.47 (d, *J* = 5.5 Hz), 114.14 (d, *J* = 13.0 Hz), 55.43. **^{31}P NMR (243 MHz, CDCl$_3$)** δ 32.63. **ESI-MS:** calculated [$C_{24}H_{23}O_3P$ + H]$^+$: 391.1458, found: 391.1468.

(*E*)-bis(3-methoxyphenyl)(1-phenylbuta-1,3-dien-2-yl)phosphine oxide (4n)

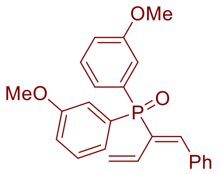

Colorless oil, (28.6 mg, 73%); Reaction time: 48 h; **^{1}H NMR (400 MHz, $CDCl_3$)** δ 7.41 – 7.31 (m, 8H), 7.30 – 7.27 (m, 1H), 7.27 – 7.24 (m, 1H), 7.10 – 7.04 (m, 2H), 6.92 (d, *J* = 21.4 Hz, 1H), 6.80 – 6.64 (m, 1H), 5.82 – 5.69 (m, 1H), 5.42 – 5.28 (m, 1H), 3.83 (s, 6H). **^{13}C NMR (150 MHz, $CDCl_3$)** δ 159.71 (d, *J* = 15.2 Hz), 143.97 (d, *J* = 11.5 Hz), 135.34 (d, *J* = 18.0 Hz), 133.27 (d, *J* = 102.7 Hz), 132.41 (d, *J* = 96.6 Hz), 131.04 (d, *J* = 8.0 Hz), 130.04, 129.78 (d, *J* = 14.4 Hz), 129.00, 128.45, 124.39 (d, *J* = 10.0 Hz), 122.68 (d, *J* = 5.5 Hz), 118.29, 116.77 (d, *J* = 10.4 Hz), 55.57 . **^{31}P NMR (162 MHz, $CDCl_3$)** δ 32.73. **ESI-MS:** calculated $[C_{24}H_{23}O_3P + H]^+$: 391.1458, found: 391.1468.

((1*E*,3*E*)-1,4-diphenylbuta-1,3-dien-2-yl)bis(4-methoxyphenyl)phosphine oxide (4o)

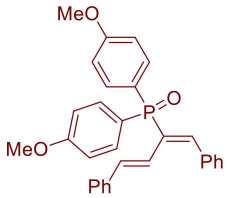

White solid, (19.6 mg, 42%); Reaction time: 48 h; **^{1}H NMR (600 MHz, CD_3OD)** δ 7.71 – 7.62 (m, 4H), 7.43 – 7.31 (m, 5H), 7.25 – 7.21 (m, 2H), 7.21 – 7.15 (m, 3H), 7.15 – 7.07 (m, 5H), 7.00 (d, *J* = 16.8 Hz, 1H), 6.89 (d, *J* = 21.2 Hz, 1H), 3.84 (s, 6H). **^{1}H NMR (600 MHz, $CDCl_3$)** δ 7.73 – 7.66 (m, 4H), 7.40 – 7.34 (m, 4H), 7.34 – 7.28 (m, 1H), 7.27 – 7.21 (m, 4H), 7.21 – 7.11 (m, 3H), 6.99 – 6.96 (m, 4H), 6.92 (d, *J* = 20.9 Hz, 1H), 3.83 (s, 6H). **^{13}C NMR (150 MHz, $CDCl_3$)** δ 162.44, 143.36 (d, J = 11.7 Hz), 137.32, 136.01 (d, J = 5.2 Hz), 135.92, 135.92, 135.80, 134.02 (d, J = 11.0 Hz), 132.85 (d, J = 96.6 Hz), 130.10, 128.82, 128.58 (d, J = 9.7 Hz), 128.00, 126.78, 123.58 (d, J = 110.4 Hz), 123.51 (d, J = 8.0 Hz), 114.21 (d, J = 13.1 Hz), 55.45. **^{31}P NMR (243 MHz, $CDCl_3$)** δ 33.39. **ESI-MS:** calculated $[C_{30}H_{27}O_3P + Na]^+$: 489.1590, found: 489.1596.

Synthetic transformations of 3a and 4b

Derivatization of the product **3a**

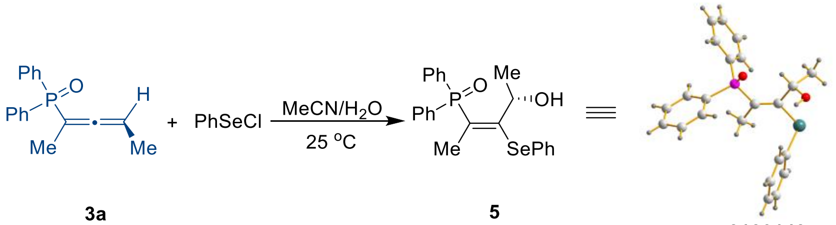

Compound 5 was prepared from a modified literature procedure.[5]

(*S*,*E*)-(4-hydroxy-3-(phenylselanyl)pent-2-en-2-yl)diphenylphosphine oxide (5)

To a solution of PhSeCl (29.0 mg, 0.15 mmol) in 1 mL of MeCN was added 0.1 mL of H_2O. Then a solution of **3a** (26.8 mg, 0.1 mmol) in 1 mL of MeCN was added and the resulting mixture was stirred at room temperature for 10 min. After complete consumption of the starting material as monitored by TLC (eluent: petroleum ether/ethyl acetate=1:1), the residue was concentrated *in vacuo*, and purified by flash column chromatography (PE/EA = 3:1) on silica gel afforded **(*S*,*E*)-5** (37.9 mg, 86%) as a white solid. **^{1}H NMR (500 MHz, $CDCl_3$)** δ 7.78 – 7.66 (m, 2H), 7.67 – 7.44 (m, 10H), 7.34 – 7.27 (m, 3H), 6.13 (s, 1H), 4.75 (s, 1H), 1.75 (d, *J* = 13.8 Hz, 3H), 1.51 (d, *J* = 6.6 Hz, 3H). **^{31}P NMR (202 MHz, $CDCl_3$)** δ 33.88. **^{13}C NMR (125 MHz, $CDCl_3$)** δ 162.41 (d, *J* = 6.8 Hz), 134.06, 132.37 (d, *J* = 2.6 Hz), 132.17 (d, *J* = 2.5 Hz), 132.08 (d, *J* = 105.6 Hz), 132.02 (d, *J* = 1.6 Hz), 131.94 (d, *J* = 2.3 Hz), 131.49 (d, *J* = 103.3 Hz), 129.61, 129.56, 128.92 (d, *J* = 12.1 Hz), 128.75 (d, *J* = 12.3 Hz), 128.16, 124.93 (d, *J* = 86.4 Hz), 70.70 (d, *J* = 6.4 Hz), 23.76 (d, *J* = 14.0

Hz), 23.14. **ESI-MS:** calculated [$C_{23}H_{23}O_2PSe$ + Na]$^+$: 465.0493, found: 465.0503. $[\alpha]^{20}_D$ = +67.3 (c = 0.58, CH_2Cl_2). The product was analyzed by HPLC to determine the enantiomeric excess: 90% e.e. (CHIRALPAK IC, hexane/*i*-PrOH = 70/30, detector: 230 nm, T = 25 °C, flow rate: 1 mL/min), t_1(major) = 15.86 min, t_2(minor) = 17.77 min.

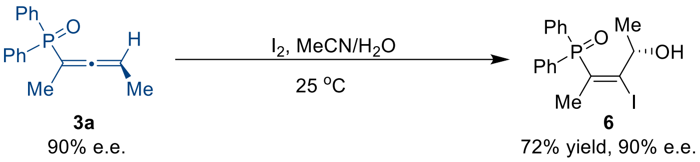

(*S*,*E*)-(4-hydroxy-3-iodopent-2-en-2-yl)diphenylphosphine oxide (6) [6]

A solution of I_2 (102 mg, 0.4 mmol) and **3a** (26.8 mg, 0.1 mmol) in 0.5 mL of MeCN was added 2.5 mL of H_2O. The resulting mixture was stirred at room temperature for 30 min. After complete consumption of the starting material as monitored by TLC (eluent: petroleum ether/ethyl acetate=1:1), the residue was concentrated *in vacuo*, and purified by flash column chromatography (PE/EA = 3:1) on silica gel afforded **(*S*,*E*)-6** (29.5 mg, 72%) as a colorless oil. **^{1}H NMR (400 MHz, $CDCl_3$)** δ 7.73 – 7.65 (m, 2H), 7.65 – 7.44 (m, 8H), 5.03 – 4.94 (m, 1H), 4.51 (s, 1H), 1.88 (d, *J* = 12.9 Hz, 3H), 1.29 (d, *J* = 6.2 Hz, 3H). **^{13}C NMR (100 MHz, $CDCl_3$)** δ 141.37 (d, *J* = 9.9 Hz), 133.10 (d, *J* = 71.2 Hz), 132.57 (d, *J* = 2.8 Hz), 132.38 (d, *J* = 2.8 Hz), 132.17 (d, *J* = 97.4 Hz), 131.80 (d, *J* = 9.9 Hz), 131.62 (d, *J* = 10.2 Hz), 131.54 (d, *J* = 103.0 Hz), 129.02 (d, *J* = 12.3 Hz), 128.86 (d, *J* = 12.3 Hz), 71.92 (d, *J* = 6.8 Hz), 31.65 (d, *J* = 13.6 Hz), 24.36. **^{31}P NMR (162 MHz, $CDCl_3$)** δ 29.96. **ESI-MS:** calculated [$C_{17}H_{18}IO_2P$ + Na]$^+$: 434.9981, found: 434.9992. $[\alpha]^{20}_D$ = -1.7 (c = 1.13, CH_2Cl_2). The product was analyzed by HPLC to determine the enantiomeric excess: 90% e.e. (CHIRALPAK IC, hexane/*i*-PrOH = 70/30, detector: 220 nm, T = 25 °C, flow rate: 1 mL/min), t_1(major) = 22.12 min, t_2(minor) = 24.04 min.

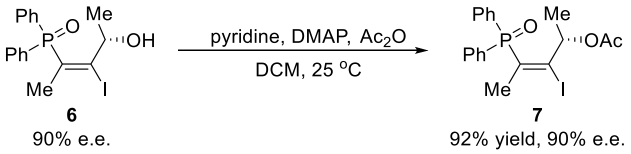

(*S*,*E*)-4-(diphenylphosphoryl)-3-iodopent-3-en-2-yl acetate (7)

To a solution of Pyridine (33.2 mg, 0.42 mmol), Ac_2O (42.8 mg, 0.42 mmol) and DMAP (12.2 mg, 0.1 mmol)in 2 mL of DCM was added **6** (148 mg, 0.35 mmol) and the resulting mixture was stirred at room temperature for 2 h. After complete consumption of the starting material as monitored by TLC (eluent: petroleum ether/acetone=3:1), the residue was concentrated *in vacuo*, and purified by flash column chromatography on silica gel afforded **(*S*,*E*)-7** (146 mg, 92%) as a white solid. **^{1}H NMR (400 MHz, $CDCl_3$)** δ 7.84 – 7.73 (m, 2H), 7.70 – 7.62 (m, 2H), 7.62 – 7.45 (m, 6H), 6.41 (qd, *J* = 6.1, 1.7 Hz, 1H), 2.05 (s, 3H), 1.85 (d, *J* = 12.5 Hz, 3H), 1.38 (d, *J* = 6.2 Hz, 3H). **^{13}C NMR (100 MHz, $CDCl_3$)** δ 169.63, 135.83 (d, *J* = 9.3 Hz), 133.71 (d, *J* = 79.3 Hz), 132.38 (d, *J* = 2.8 Hz), 132.25 (d, *J* = 104.3 Hz), 132.22, 132.19, 132.08, 131.88 (d, *J* = 9.7 Hz), 131.15 (d, *J* = 104.3 Hz), 128.80 (d, *J* = 12.3 Hz), 71.47 (d, *J* = 5.8 Hz), 30.74 (d, *J* = 13.3 Hz), 21.64 (d, *J* = 0.9 Hz), 21.44. **^{31}P NMR (162 MHz, $CDCl_3$)** δ 28.80. **ESI-MS:** calculated [$C_{19}H_{20}IO_3P$ + H]$^+$: 455.0268, found: 455.0280. $[\alpha]^{20}_D$ = -27.4 (c = 0.95, CH_2Cl_2). The prod uct was analyzed by HPLC to determine the enantiomeric excess: 90% e.e. (CHIRALPAK AD-H, hexane/*i*-PrOH = 70/30, detector: 220 nm, T = 25 °C, flow rate: 1 mL/min), t_1(major) = 6.20 min, t_2(minor) = 11.83 min.

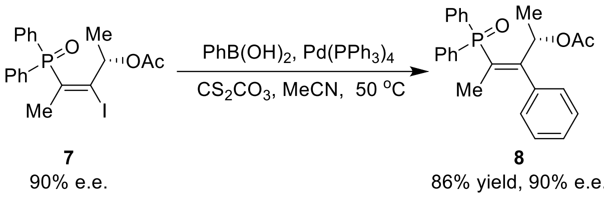

(*S*,*Z*)-4-(diphenylphosphoryl)-3-phenylpent-3-en-2-yl acetate (8)[7]

To a 10 mL tube were added **7** (45.4 mg, 0.1 mmol), $Pd(PPh_3)_4$ (5.8 mg, 0.005 mmol), CS_2CO_3 (65.2 mg, 0.2 mmol), boronic acid (14.6 mg, 0.12 mmol) and acetonitrile (2 mL) under argon. Then the resulting mixture was stirred at 50 °C for 48 h. After complete consumption of the starting material as monitored by TLC, the residue was concentrated *in vacuo*, and purified by flash column chromatography (PE/EA = 4:1) on silica gel afforded **(*S*,*Z*)-8** (34.7 mg, 86%) as a colorless oil. **^{1}H NMR (400 MHz, $CDCl_3$)** δ 7.92 – 7.80 (m, 2H), 7.78 – 7.67 (m, 2H), 7.61 – 7.44 (m, 6H), 7.43 – 7.29 (m, 3H), 7.16 – 7.04 (m, 2H), 6.75 (q, *J* = 6.5 Hz, 1H), 1.77 (s, 3H), 1.39 (d, *J* = 6.8 Hz, 3H), 1.37 (d, *J* = 13.9 Hz, 3H). **^{13}C NMR (100 MHz, $CDCl_3$)** δ 169.88, 157.84 (d, *J* = 6.9 Hz), 138.15 (d, *J* = 14.6 Hz), 133.05 (d, *J* = 102.8 Hz), 132.60 (d, *J* = 102.1 Hz), 132.06, 132.02, 131.99, 131.97 (d, *J* = 1.7 Hz), 131.88, 131.80 (d, *J* = 2.7 Hz), 128.68 (d, *J* = 3.5 Hz), 128.56 (d, *J* = 3.3 Hz), 128.33, 127.49, 126.79 (d, *J* = 91.0 Hz), 70.79 (d, *J* = 7.4 Hz), 21.06, 20.24 (d, *J* = 13.7 Hz), 19.88. **^{31}P NMR (162 MHz, $CDCl_3$)** δ 31.15. **ESI-MS:** calculated [$C_{25}H_{25}O_3P$ + H] $^+$: 405.1614, found: 405.1614. $[\alpha]^{20}_D$ = -59.9 (c = 1.00, CH_2Cl_2). The product was analyzed by HPLC to determine the enantiomeric excess: 90% e.e. (CHIRALPAK OD-H, hexane/*i*-PrOH = 95/5, detector: 220 nm, T = 25 °C, flow rate: 1 mL/min), t_1(major) = 8.79 min, t_2(minor) = 10.36 min.

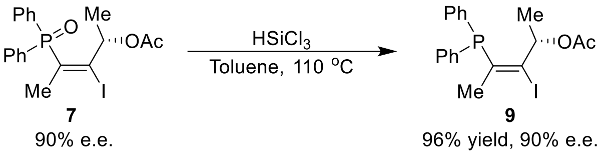

(*S*,*E*)-4-(diphenylphosphaneyl)-3-iodopent-3-en-2-yl acetate (9)[8]

To a solution of **7** (45.4 mg, 0.1 mmol) in toluene (2 mL) was added $HSiCl_3$ (40.6 mg, 0.3 mmol) slowly under a nitrogen atmosphere. Then the resulting mixture was stirred at 110 °C for 4 h. After complete consumption of the starting material as monitored by TLC, the residue was concentrated *in vacuo*, and purified by flash column chromatography (PE/EA = 30:1) on silica gel afforded **(*S*,*E*)-9** (42.0 mg, 96%) as a colorless oil. **^{1}H NMR (500 MHz, $CDCl_3$)** δ 7.43 – 7.29 (m, 10H), 6.33 (dq, *J* = 9.0, 6.4 Hz, 1H), 2.09 (s, 3H), 1.84 (d, *J* = 2.5 Hz, 3H), 1.29 (d, *J* = 6.4 Hz, 3H). **^{13}C NMR (125 MHz, $CDCl_3$)** δ 169.79, 140.33 (d, *J* = 30.2 Hz), 136.02 (d, *J* = 14.9 Hz), 135.54 (d, *J* = 13.8 Hz), 132.99 (d, *J* = 19.3 Hz), 132.66 (d, *J* = 18.7 Hz), 128.84, 128.83, 128.78, 128.56 (d, *J* = 6.4 Hz), 126.27 (d, *J* = 28.2 Hz), 72.49, 72.12, 29.43 (d, *J* = 3.0 Hz), 21.43 (d, *J* = 7.5 Hz). **^{31}P NMR (202 MHz, $CDCl_3$)** δ -3.29. **ESI-MS:** calculated [$C_{19}H_{20}IO_2P$ + H] $^+$: 439.0318, found: 439.0320. $[\alpha]^{20}_D$ = -23.5 (c = 1.05, CH_2Cl_2). The product was analyzed by HPLC to determine the enantiomeric excess: 90% e.e. (CHIRALPAK AD-H, hexane/*i*-PrOH = 95/5, detector: 220 nm, T = 25 °C, flow rate: 1 mL/min), t_1(major) = 4.00 min, t_2(minor) = 6.14 min.

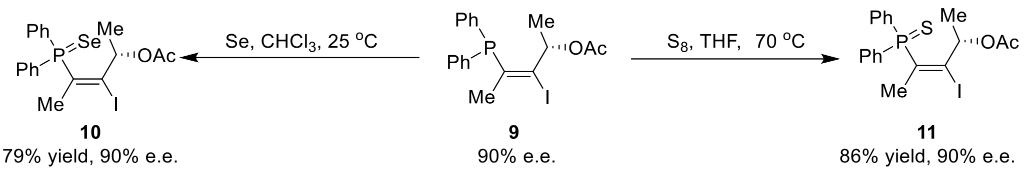

(*S*,*E*)-4-(diphenylphosphoroselenoyl)-3-iodopent-3-en-2-yl acetate (10)[9]

To a solution of **9** (43.8 mg, 0.1 mmol) in chloroform (2 mL) was added selenium (23.7 mg, 0.3 mmol) under nitrogen atmosphere. Then the resulting mixture was stirred at 25 °C for 2 h. After complete consumption of the starting material as monitored by TLC, the residue was concentrated *in vacuo*, and purified by flash column chromatography (PE/EA = 20:1) on silica gel afforded **(*S*,*E*)-10** (40.7 mg, 79%) as a white solid. **^{1}H NMR (400 MHz, $CDCl_3$)** δ 8.02 – 7.93 (m, 2H), 7.91 – 7.82 (m, 2H), 7.56 – 7.41 (m, 6H), 5.25 (qd, *J* = 5.9, 1.5 Hz, 1H), 2.00 (d, *J* = 13.3 Hz, 3H), 1.87 (s, 3H), 1.20 (d, *J* = 5.9 Hz, 3H). **^{13}C NMR (100 MHz, $CDCl_3$)** δ 168.45, 135.30 (d, *J* = 54.8 Hz), 132.77 (d, *J* = 10.8 Hz), 132.59 (d, *J* = 11.0 Hz), 132.19 (d, *J* = 3.1 Hz), 131.91 (d, *J* = 3.0 Hz), 131.32 (d, *J* = 75.3 Hz), 130.94 (d, *J* = 73.6 Hz), 128.77 (d, *J* = 3.5 Hz), 128.64 (d, *J* = 3.7 Hz), 128.29 (d, *J* = 8.1 Hz), 72.06 (d, *J* = 10.5 Hz), 31.99 (d, *J* = 13.1 Hz), 21.11, 20.92. **^{31}P NMR (162 MHz, $CDCl_3$)** δ 32.23. **ESI-MS:** calculated [$C_{19}H_{20}IO_2PSe$ + Na]$^+$: 540.9303, found: 540.9315. $[\alpha]^{20}_D$ = -10.9 (c = 0.95, CH_2Cl_2). The product was analyzed by HPLC to determine the enantiomeric excess: 90% e.e. (CHIRALPAK IC, hexane/*i*-PrOH = 95/5, detector: 220 nm, T = 25 °C, flow rate: 1 mL/min), t_1(major) = 5.47 min, t_2(minor) = 6.62 min.

(*S*,*E*)-4-(diphenylphosphorothioyl)-3-iodopent-3-en-2-yl acetate (11)[10]

To a solution of **9** (43.8 mg, 0.1 mmol) in tetrahydrofuran (2 mL) was added sulfur (51.4 mg, 0.2 mmol) under nitrogen atmosphere. Then the resulting mixture was stirred at 70 °C for 24 h. After complete consumption of the starting material as monitored by TLC, the residue was concentrated *in vacuo*, and purified by flash column chromatography (PE/EA = 20:1) on silica gel afforded **(*S*,*E*)-10** (40.2 mg, 86%) as a white solid. **^{1}H NMR (400 MHz, $CDCl_3$)** δ 7.95 – 7.87 (m, 2H), 7.86 – 7.78 (m, 2H), 7.56 – 7.42 (m, 6H), 5.35 (qd, *J* = 5.9, 1.6 Hz, 1H), 1.99 (d, *J* = 13.1 Hz, 3H), 1.87 (s, 3H), 1.21 (d, *J* = 6.0 Hz, 3H). **^{13}C NMR (100 MHz, $CDCl_3$)** δ 168.50, 136.89 (d, *J* = 62.7 Hz), 132.82 (d, *J* = 84.2 Hz), 132.34 (d, *J* = 82.2 Hz), 132.13 (d, *J* = 3.7 Hz), 132.08 (d, *J* = 5.7 Hz), 131.98 (d, *J* = 5.8 Hz), 131.87 (d, *J* = 3.0 Hz), 129.17 (d, *J* = 8.7 Hz), 128.78 (d, *J* = 3.4 Hz), 128.66 (d, *J* = 3.6 Hz), 72.17 (d, *J* = 10.1 Hz), 32.07 (d, *J* = 12.8 Hz), 21.22, 21.12. **^{31}P NMR (162 MHz, $CDCl_3$)** δ 41.06. **ESI-MS:** calculated [$C_{19}H_{20}IO_2PS$ + Na]$^+$: 492.9859, found: 492.9869. $[\alpha]^{20}_D$ = -23.9 (c = 1.03, CH_2Cl_2). The product was analyzed by HPLC to determine the enantiomeric excess: 90% e.e. (CHIRALPAK AD-H, hexane/*i*-PrOH = 95/5, detector: 220 nm, T = 25 °C, flow rate: 1 mL/min), t_1(major) = 4.89 min, t_2(minor) = 5.46 min.

Derivatization of the product **4b**

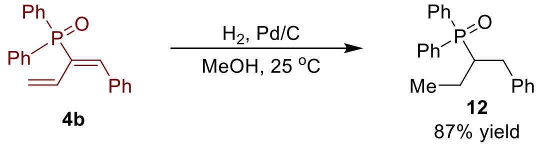

Diphenyl(1-phenylbutan-2-yl)phosphine oxide (12)

(*E*)-diphenyl(1-phenylbuta-1,3-dien-2-yl)phosphine oxide **4b** (66.0 mg, 0.02 mmol) and Pd/C (5 wt%, 6.5 mg) was suspended in anhydrous methanol (3 mL), then H_2 gas was gently bubbled directly through the solution via a stainless-steel needle at room temperature. After stirring for 3 hours, the reaction was complete (monitored by TLC). The solvent was removed in vacuo and the product was dissolved in ethyl acetate. The resulting solution was filtrated through a plug of celite and followed by evaporation of solvent. The crude material was purified by column chromatography to give compound **12** (58.5 mg, 87%). **^{1}H NMR (500 MHz, $CDCl_3$)** δ 7.91 – 7.78 (m, 4H), 7.55 – 7.39 (m, 6H), 7.25 – 7.05 (m, 5H), 3.09 – 2.93 (m, 1H), 2.91 – 2.76 (m, 1H), 2.65 – 2.49 (m, 1H), 1.84 – 1.66 (m, 1H), 1.64 – 1.53 (m, 1H), 0.79 (t, *J* = 7.5 Hz, 3H). **^{13}C NMR (125 MHz, $CDCl_3$)** δ 140.08 (d, *J* = 13.1 Hz), 133.06 (d, *J* = 94.3 Hz), 132.99 (d, *J* = 93.9 Hz), 131.57 (d, *J* = 2.3 Hz), 131.53 (d, *J* = 2.7 Hz), 131.00 (d, *J* = 3.2 Hz), 130.94 (d, *J* = 3.3 Hz), 128.91, 128.75 (d, *J* = 3.3 Hz), 128.66 (d, *J* = 3.2 Hz), 128.47, 126.30, 40.80 (d, *J* = 69.8 Hz), 33.15, 20.58, 12.52 (d, *J* = 6.8 Hz). **^{31}P NMR (202 MHz, $CDCl_3$)** δ 35.36. **ESI-MS:** calculated [$C_{22}H_{23}OP$ + H]$^+$: 335.1559, found: 335.1575.

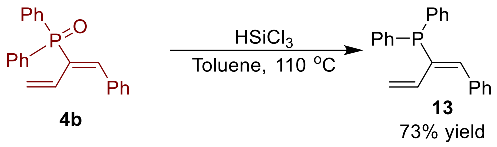

4b

13
73% yield

(*E*)-diphenyl(1-phenylbuta-1,3-dien-2-yl)phosphane (13)[8]

To a solution of **4b** (33.0 mg, 0.1 mmol) in toluene (2 mL) was added $HSiCl_3$ (40.6 mg, 0.3 mmol) slowly under a nitrogen atmosphere. Then the resulting mixture was stirred at 110 °C for 4 h. After complete consumption of the starting material as monitored by TLC, the residue was concentrated *in vacuo*, and purified by flash column chromatography (PE/EA = 30:1) on silica gel afforded **(*E*)-13** (23.0 mg, 73%) as a colorless oil. **^{1}H NMR (500 MHz, $CDCl_3$)** δ 7.37 – 7.31 (m, 4H), 7.30 – 7.26 (m, 6H), 7.25 – 7.21 (m, 2H), 7.20 – 7.15 (m, 3H), 6.91 – 6.79 (m, 1H), 6.13 (d, *J* = 6.4 Hz, 1H), 5.72 – 5.65 (m, 1H), 5.18 – 5.13 (m, 1H). **^{13}C NMR (125 MHz, $CDCl_3$)** δ 138.48 (d, *J* = 5.6 Hz), 137.75 (d, *J* = 16.5 Hz), 137.44 (d, *J* = 3.6 Hz), 135.74 (d, *J* = 10.6 Hz), 135.27 (d, *J* = 22.3 Hz), 134.39 (d, *J* = 20.0 Hz), 129.44, 129.04, 128.67 (d, *J* = 7.0 Hz), 128.23, 127.51, 118.83 (d, *J* = 20.9 Hz). **^{31}P NMR (202 MHz, $CDCl_3$)** δ -5.72. **ESI-MS:** calculated [$C_{22}H_{19}P$ + H]$^+$: 315.1297, found: 315.1297.

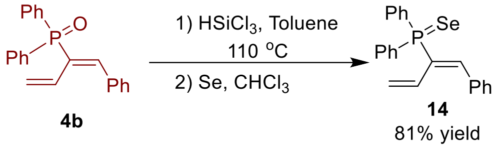

4b

14
81% yield

(*E*)-diphenyl(1-phenylbuta-1,3-dien-2-yl)phosphine selenide (14)[9]

To a solution of **4b** (33.0 mg, 0.1 mmol) in toluene (2 mL) was added $HSiCl_3$ (40.6 mg, 0.3 mmol) slowly under a nitrogen atmosphere. Then the resulting mixture was stirred at 110 °C for 4 h. After complete consumption of the starting material as monitored by TLC, the residue was concentrated *in vacuo* and redissolve in chloroform (2 mL), then selenium (23.7 mg, 0.3 mmol) was added under nitrogen atmosphere. Then the resulting mixture was stirred at 25 °C for 2 h. After complete consumption of the starting material as monitored by TLC, the residue was concentrated *in vacuo*, and purified by flash column chromatography (PE/EA = 20:1) on silica gel afforded **(*E*)-14** (32.0 mg, 81%) as a colorless oil. **^{1}H NMR (400 MHz, $CDCl_3$)** δ 7.96 – 7.83 (m, 4H), 7.54 – 7.42 (m,

6H), 7.41 – 7.28 (m, 5H), 6.95 (d, *J* = 23.5 Hz, 1H), 6.71 – 6.55 (m, 1H), 5.68 – 5.56 (m, 1H), 5.40 – 5.30 (m, 1H). **^{13}C NMR (100 MHz, $CDCl_3$)** δ 143.98 (d, *J* = 11.7 Hz), 135.33 (d, *J* = 18.3 Hz), 133.09 (d, *J* = 10.6 Hz), 131.77 (d, *J* = 3.0 Hz), 131.14 (d, *J* = 68.8 Hz), 130.72 (d, *J* = 66.9 Hz), 130.30, 130.11 (d, *J* = 1.0 Hz), 129.05, 128.69 (d, *J* = 12.4 Hz), 128.44, 123.30 (d, *J* = 7.1 Hz). **^{31}P NMR (162 MHz, $CDCl_3$)** δ 37.41. **ESI-MS:** calculated [$C_{22}H_{19}PSe$ + H]$^+$: 395.0462, found: 395.0464.

Confirmation of absolute configuration

(*S*)-**S1** and (*S*)-**S2** were prepared according to a modified literature procedure.[3,5,11-12]

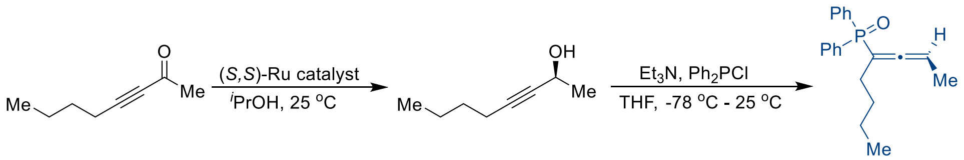

S1 S2

(*S*)-octa-2,3-dien-4-yldiphenylphosphine oxide (S2):[5,6-7] To an oven-dried 25 mL round-bottom flask equipped with a magnetic stir bar and sealed with a rubber septum under an atmosphere of nitrogen were added THF (6 mL), Et_3N (0.42 mL, 3.0 mmol, 1.5 equiv) and (*S*)-oct-3-yn-2-ol **S1** (245 mg, 2.0 mmol, 1.0 equiv) sequentially. Then Ph_2PCl (0.54 mL, 3.0 mmol) was added to the reaction mixture at -78 °C. The reaction mixture was then allowed to warm up to room temperature naturally. After complete conversion of the corresponding propargylic alcohol as monitored by TLC, the mixture was filtered off. Evaporation of the solvent and flash chromatography on silica gel afforded **S2** (453 mg, 73%). $[\alpha]^{20}_D$ = +49.5 (c = 0.97, CH_2Cl_2). The product was analyzed by HPLC to determine the enantiomeric excess: 95% e.e. (CHIRALPAK IC-IC, hexane/*i*-PrOH = 90/10, detector: 220 nm, T = 25 °C, flow rate: 0.8 mL/min), t_1(major) = 70.01 min, t_2(minor) = 73.47 min. **Specific rotation of 3p:** $[\alpha]^{20}_D$ = +46.2 (c = 1.09, CH_2Cl_2). **S2:** $[\alpha]^{20}_D$ = +49.5 (c = 0.97, CH_2Cl_2).]

X-ray single crystal data

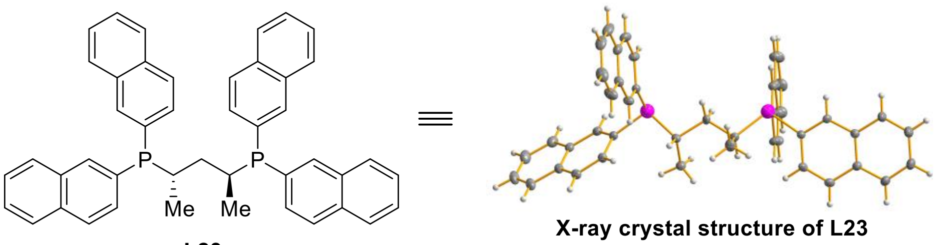

L23

X-ray crystal structure of L23
(CCDC **2106890**)

Chemical formula	$C_{45}H_{38}P_2$
Formula weight	640.69
Space group	C 1 2 1
Z	4
α, Å	29.2810(19)
b, Å	11.3776(7)
c, Å	11.2605(6)
α, °	90
β, °	107.518(4)
γ, °	90
V, Å^3	3577.4(4)

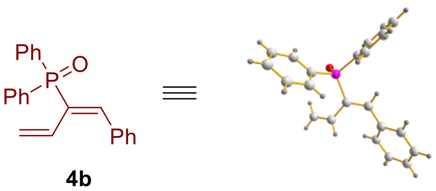

4b

CCDC (**2131762**)

Chemical formula	$C_{22}H_{19}OP$
Formula weight	330.34
Space group	P-1
Z	4
α, Å	9.926(2)
b, Å	11.807(2)
c, Å	16.325(3)
α, °	78.199(11)
β, °	84.329(12)
γ, °	89.635(10)
V, Å^3	1845.3(7)

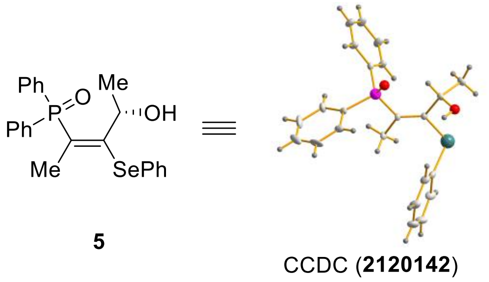

5

CCDC (**2120142**)

Chemical formula	$C_{23}H_{23}O_2P_2Se$
Formula weight	441.34
Space group	P 1 2 1
Z	4
α, Å	14.5606(4)
b, Å	8.7005(3)
c, Å	16.6209(5)
α, º	90
β, º	93.316(2)
γ, º	90
V, $Å^3$	2102.08(11)

NMR spectra

^{1}H NMR of 3a

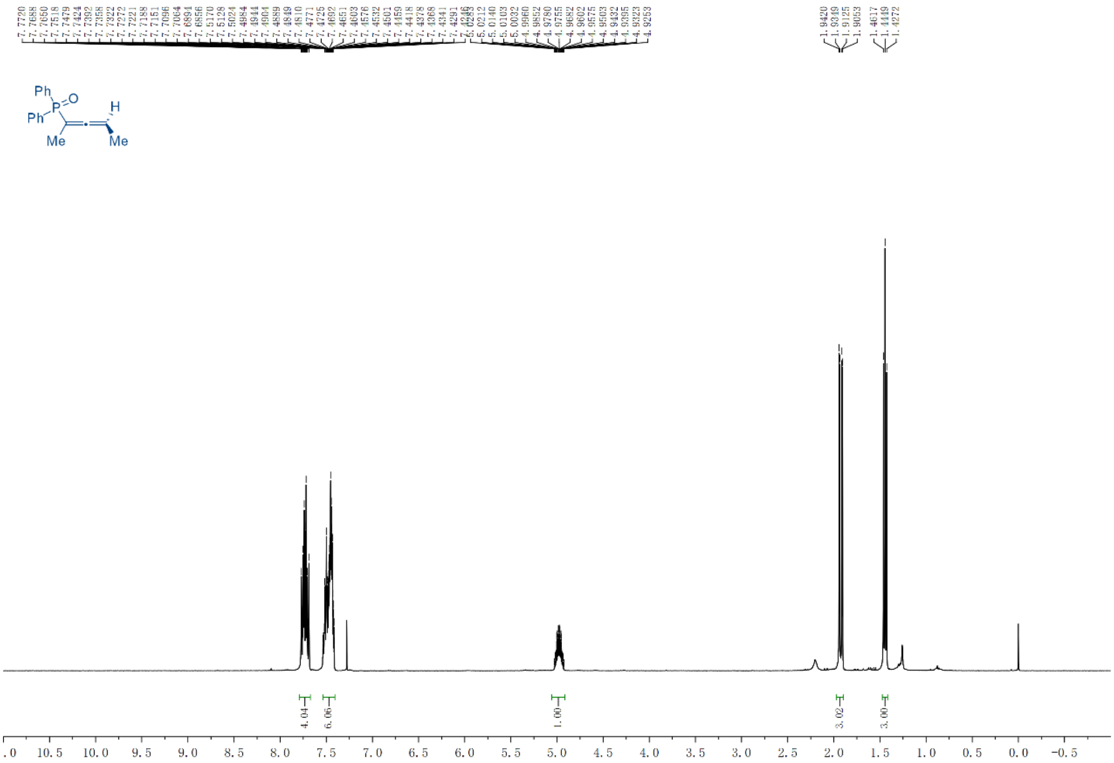

^{13}C NMR of 3a

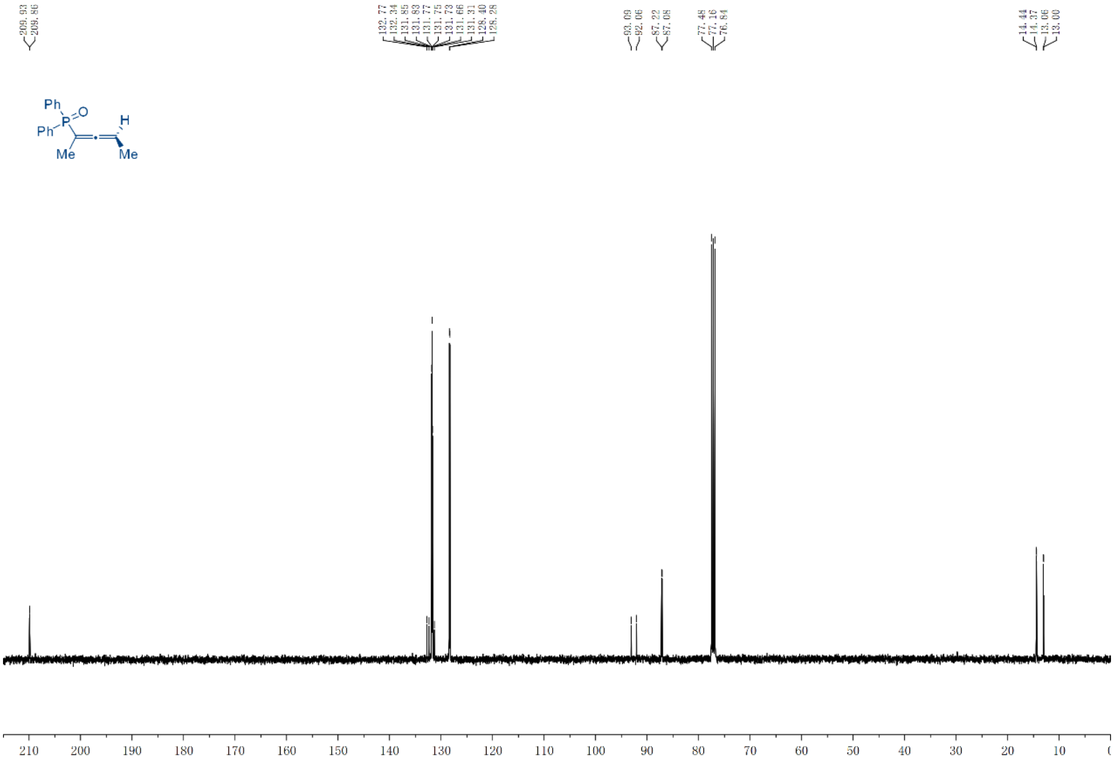

^{31}P NMR of 3a

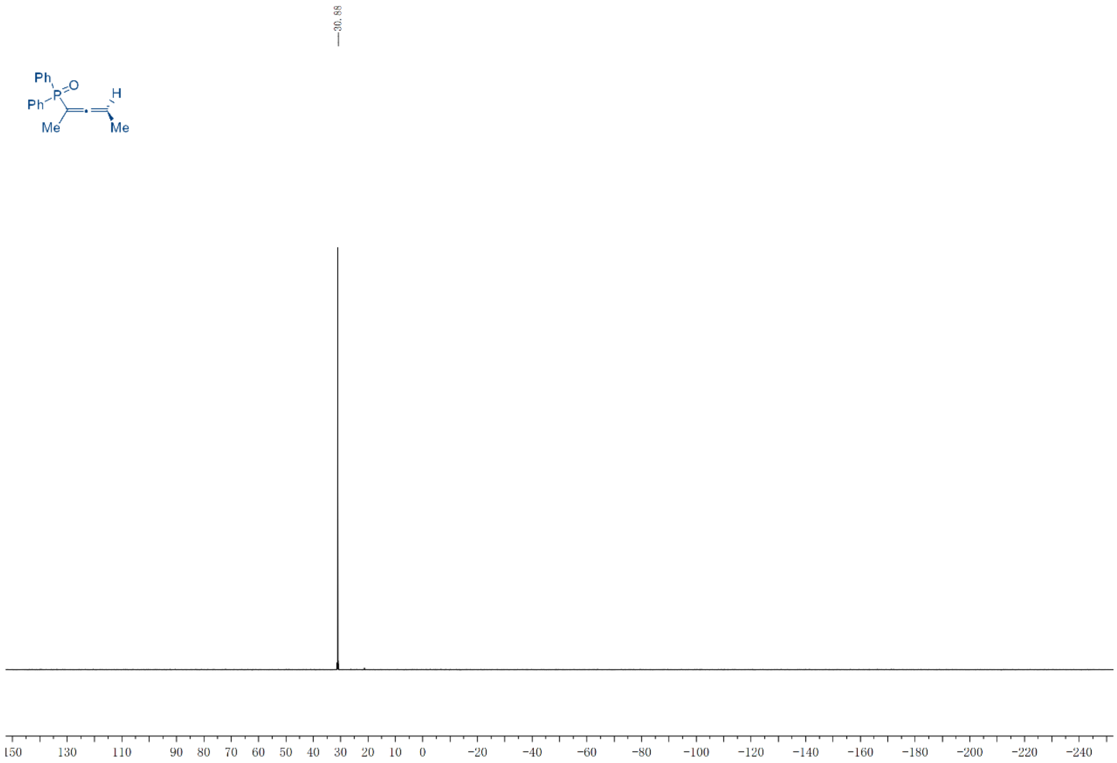

Supplementary Figure 12. ^{1}H NMR, ^{13}C NMR, and ^{31}P NMR spectra of compound **3a**

^{1}H NMR of 3b

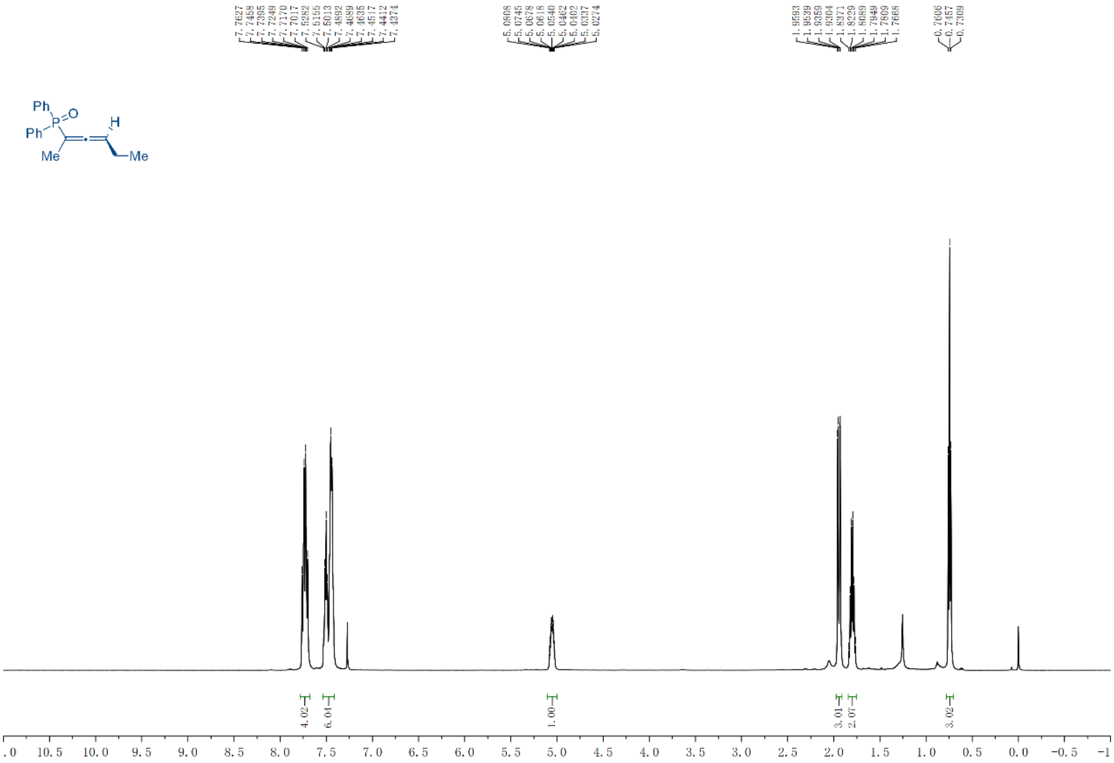

^{13}C NMR of 3b

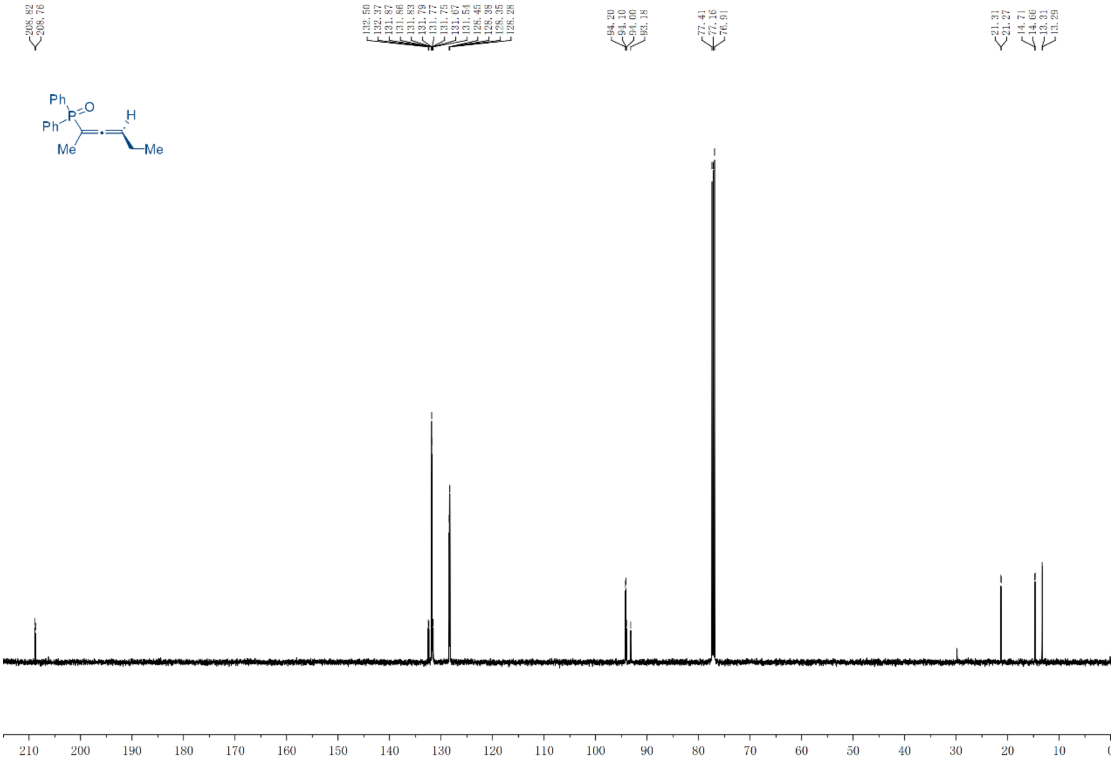

^{31}P NMR of 3b

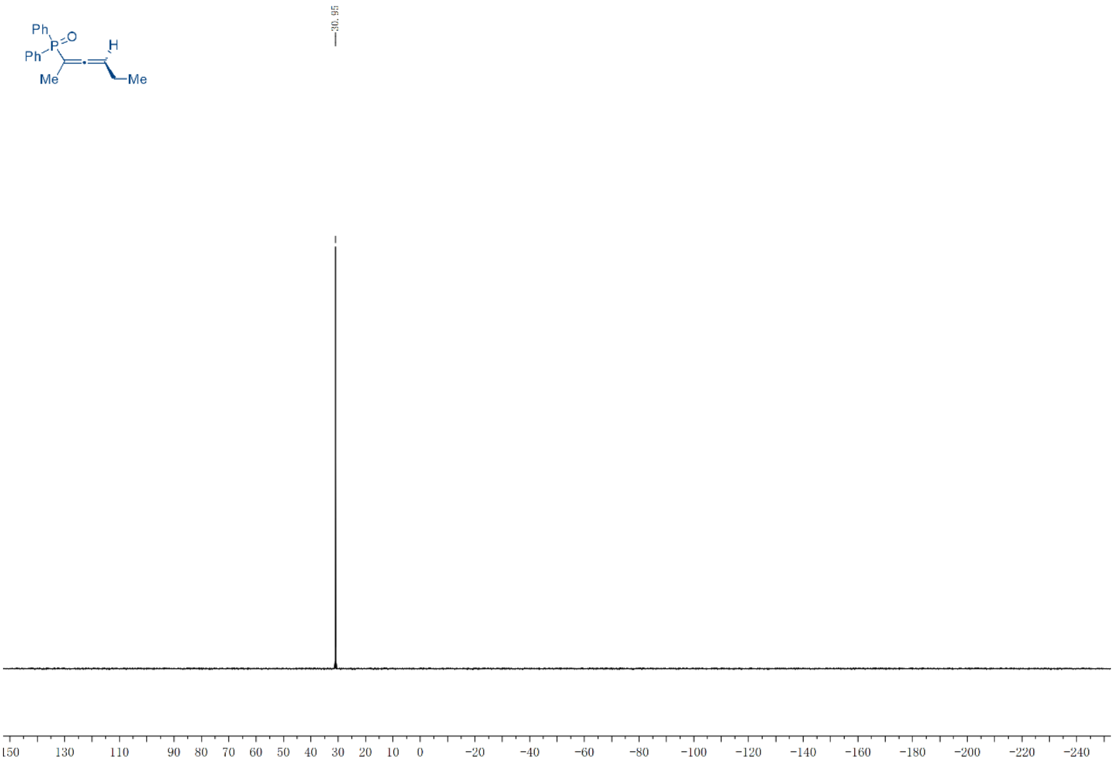

Supplementary Figure 13. ^{1}H NMR, ^{13}C NMR, and ^{31}P NMR spectra of compound **3b**

^{1}H NMR of 3c

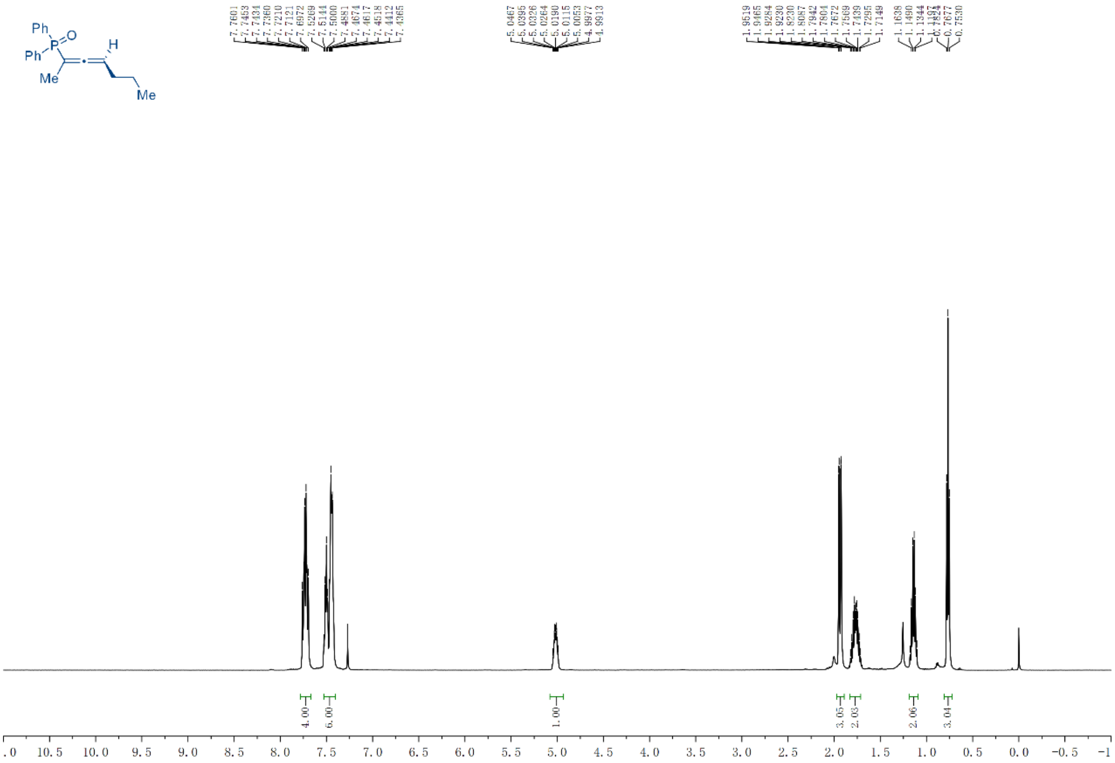

^{13}C NMR of 3c

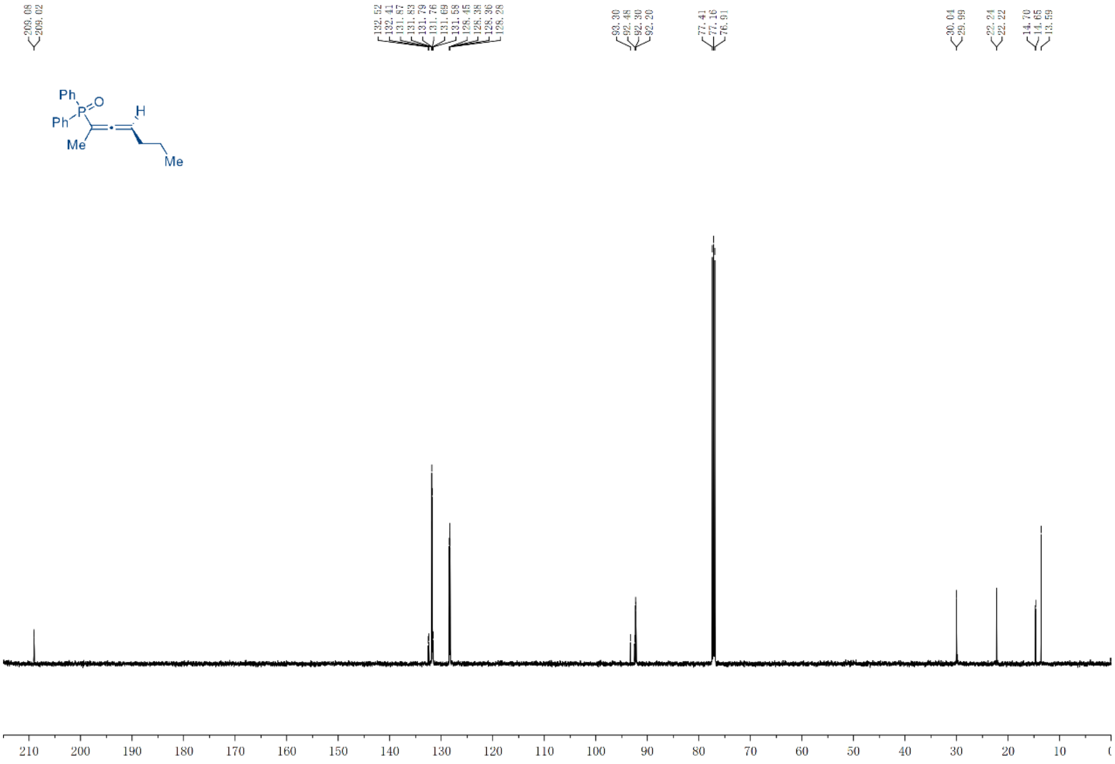

^{31}P NMR of 3c

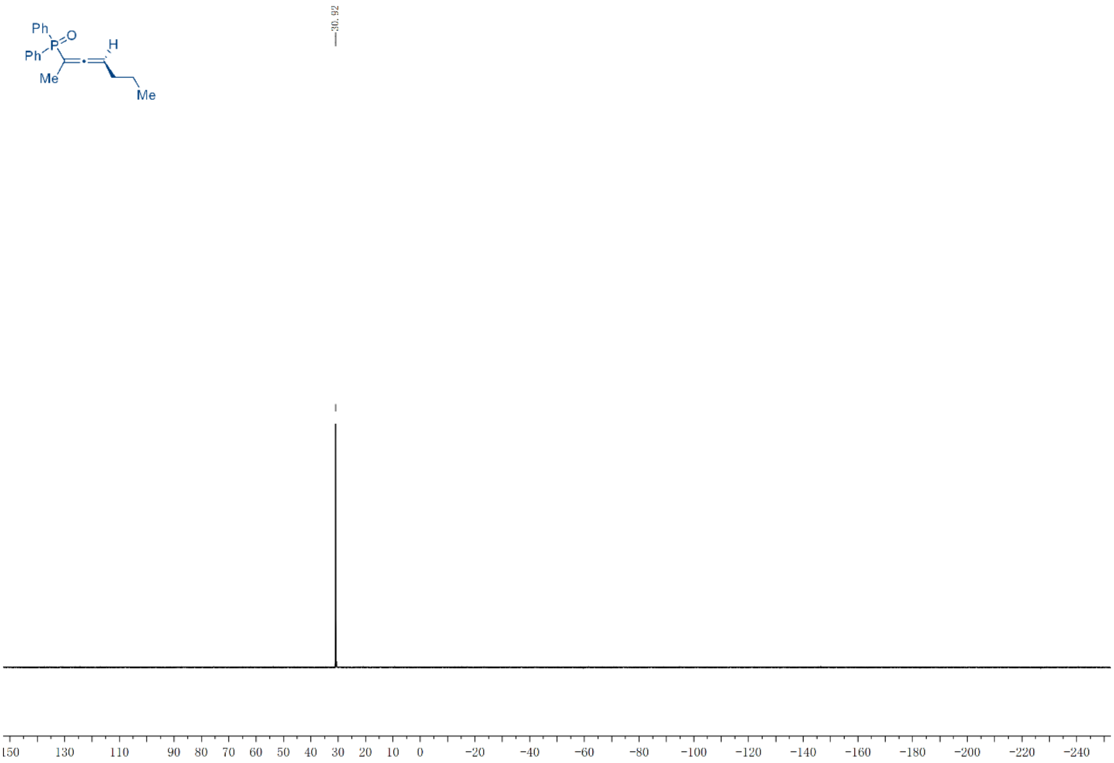

Supplementary Figure 14. ^{1}H NMR, ^{13}C NMR, and ^{31}P NMR spectra of compound **3c**

^{1}H NMR of 3d

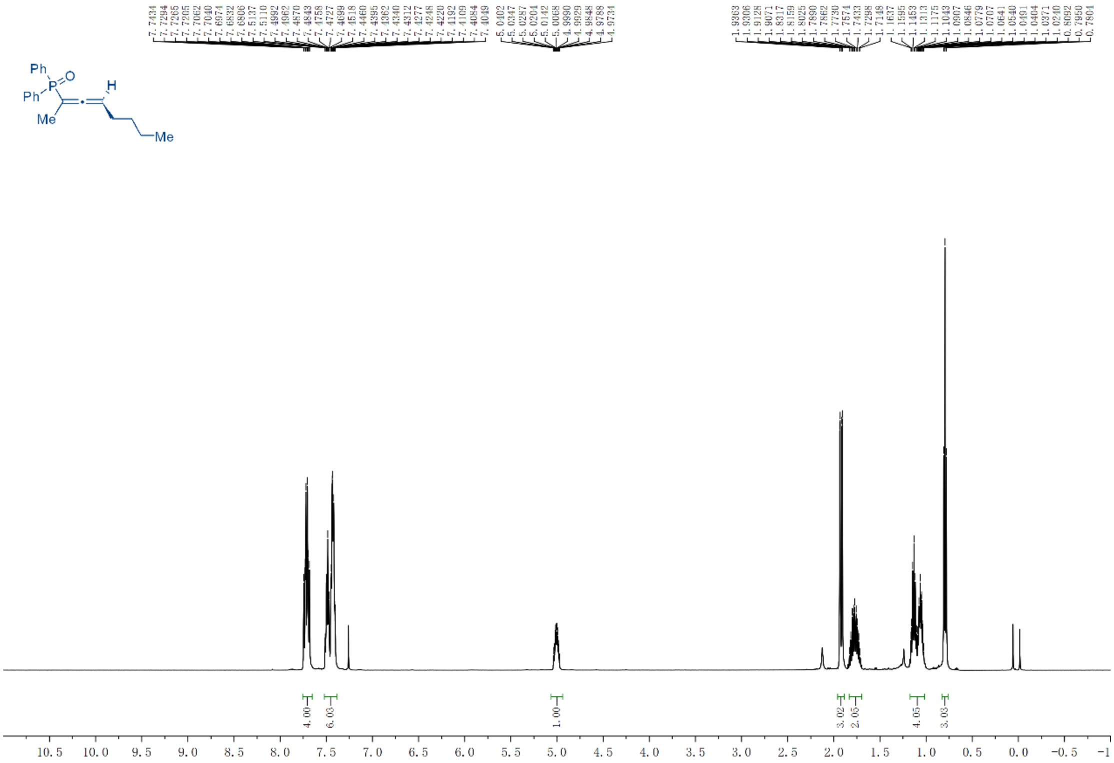

^{13}C NMR of 3d

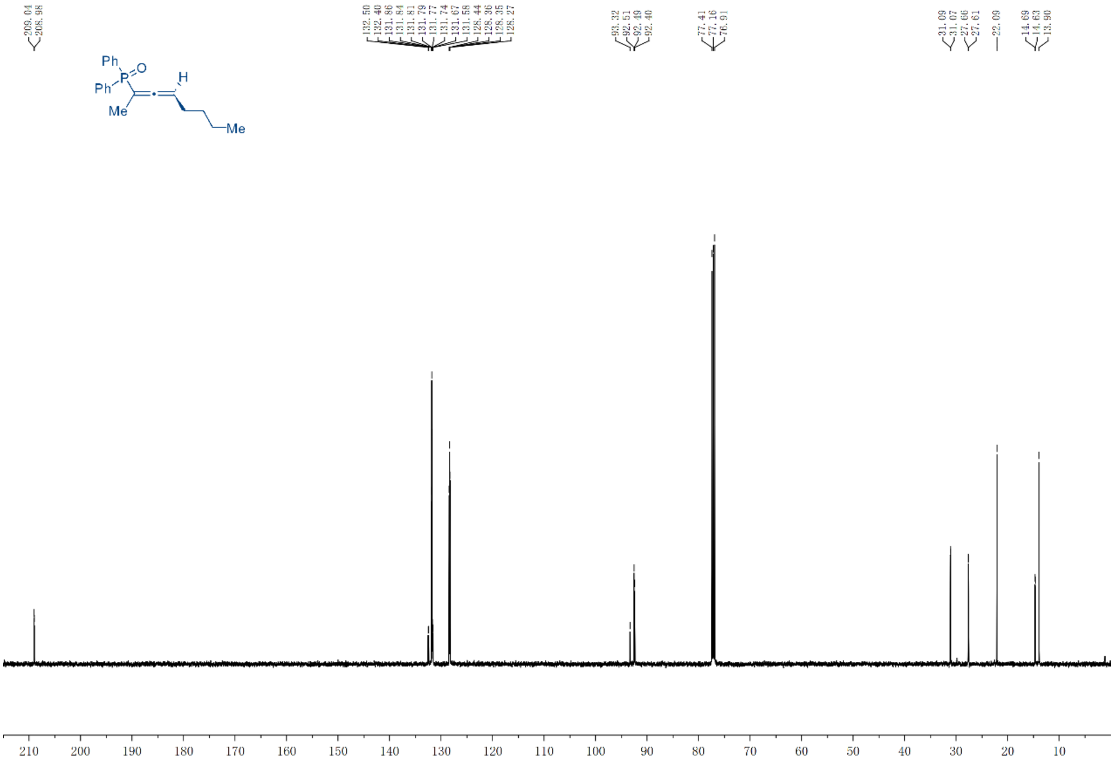

^{31}P NMR of 3d

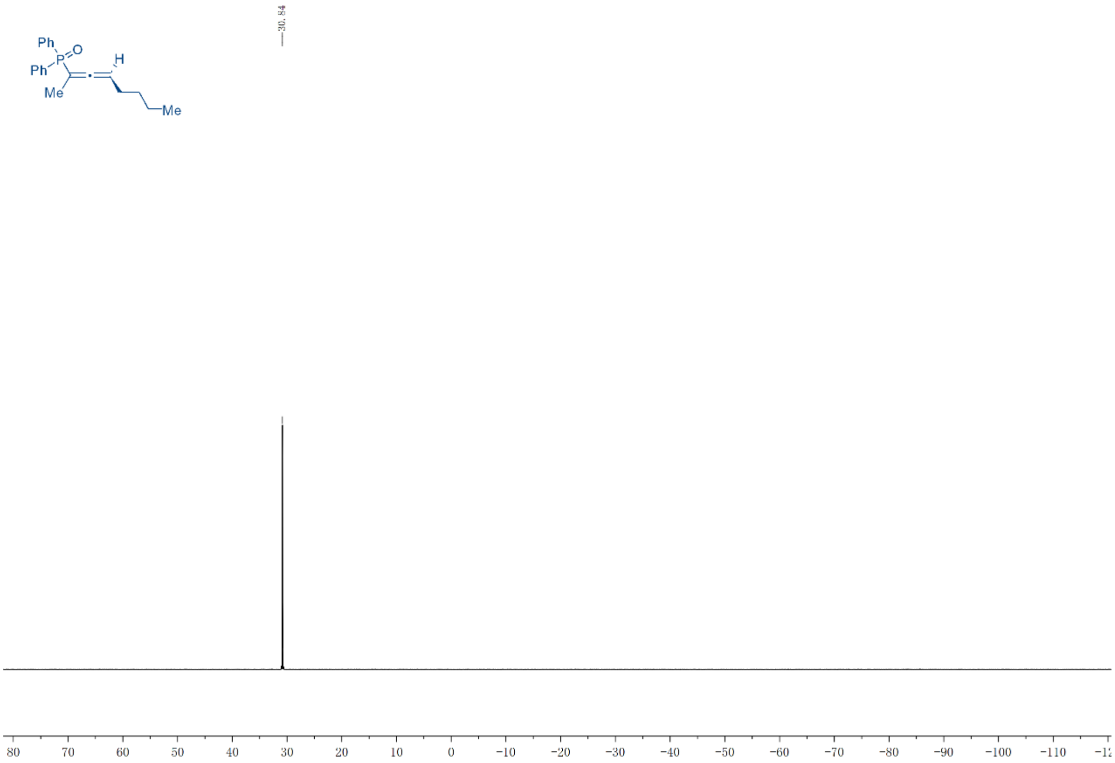

Supplementary Figure 15. ^{1}H NMR, ^{13}C NMR, and ^{31}P NMR spectra of compound **3d**

^{1}H NMR of 3e

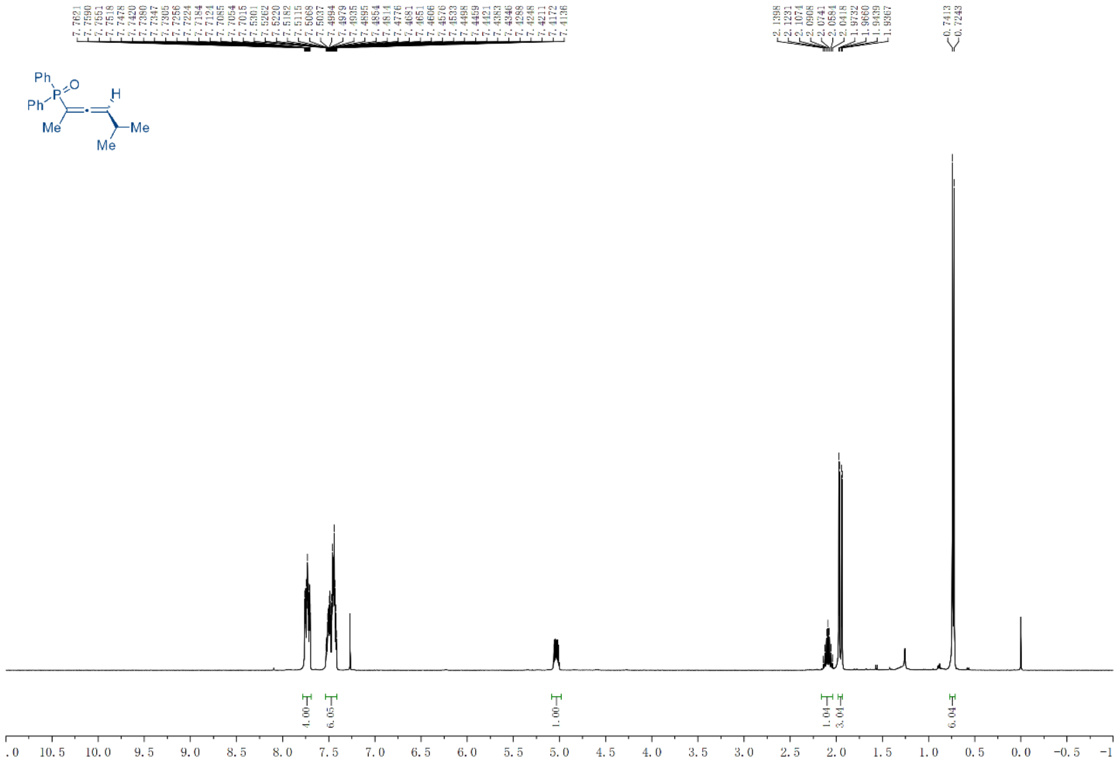

^{13}C NMR of 3e

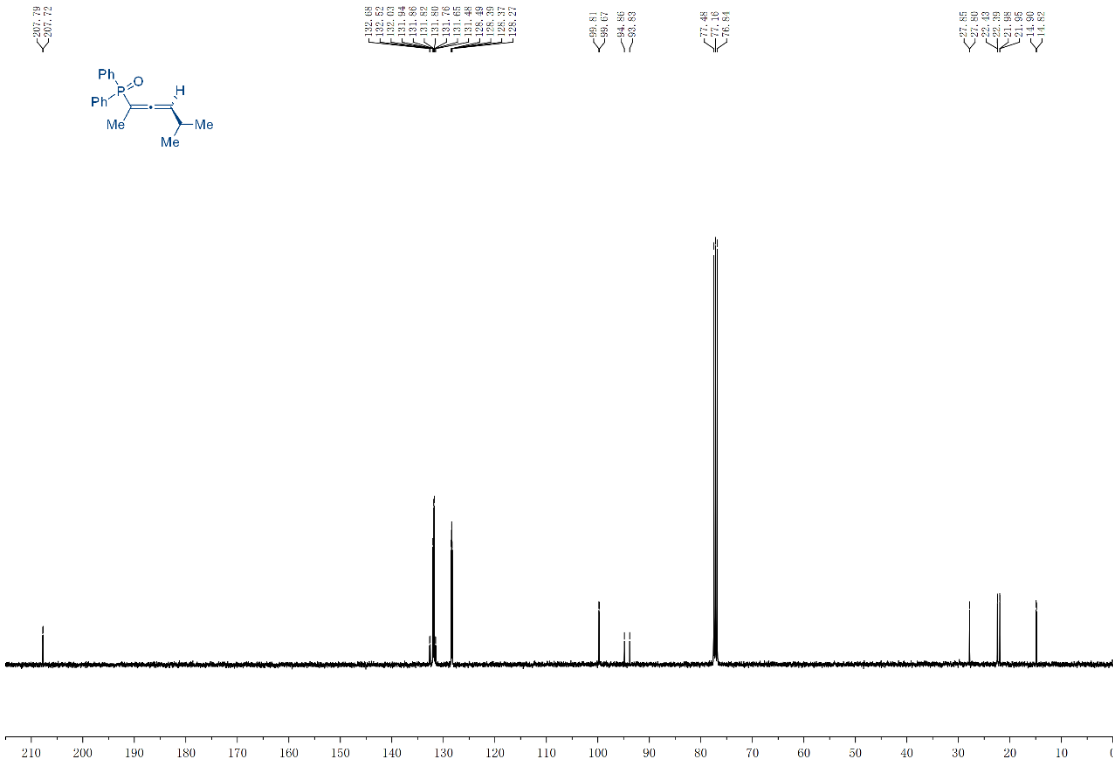

^{31}P NMR of 3e

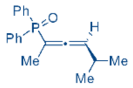

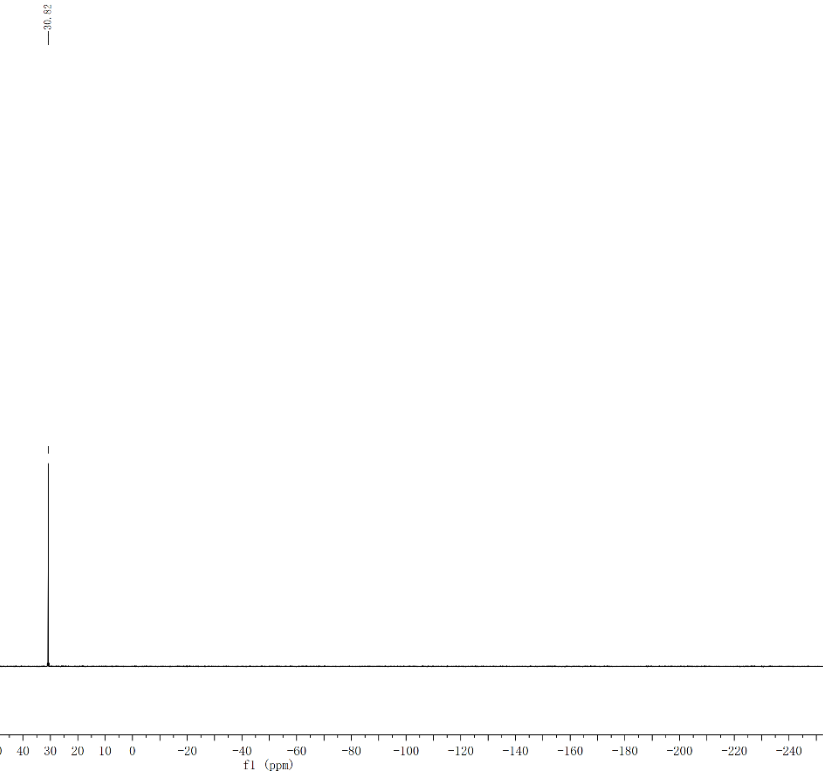

Supplementary Figure 16. ^{1}H NMR, ^{13}C NMR, and ^{31}P NMR spectra of compound **3e**

^{1}H NMR of 3f

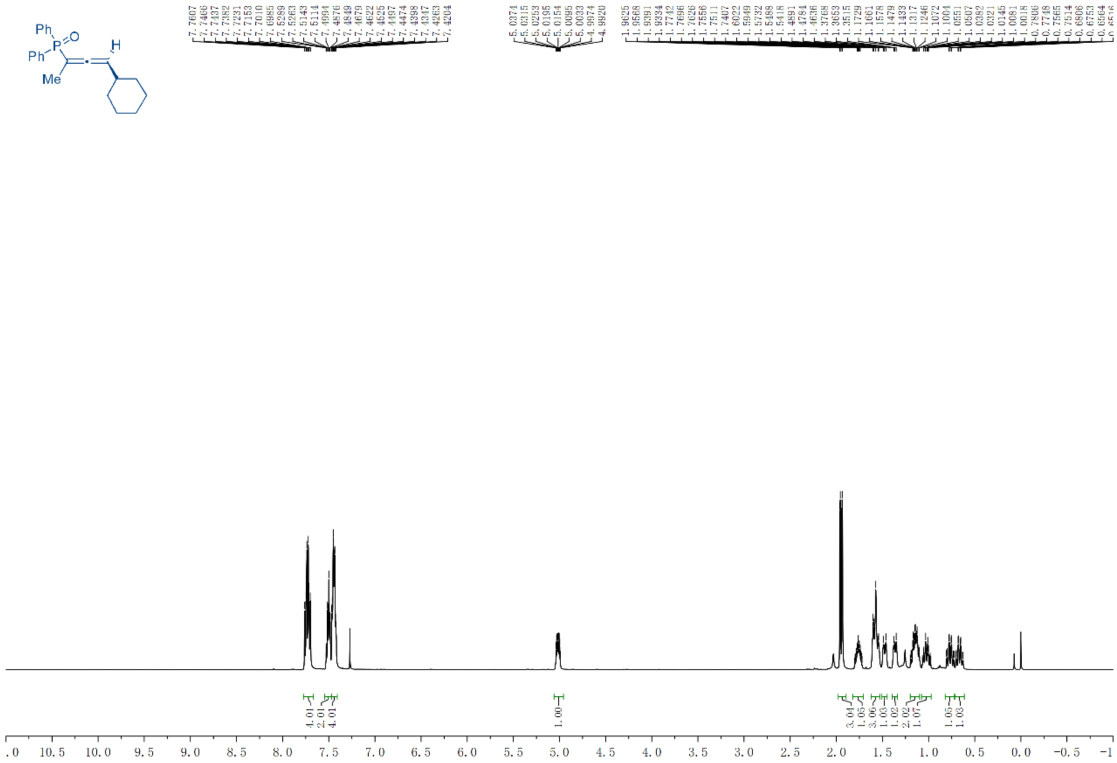

^{13}C NMR of 3f

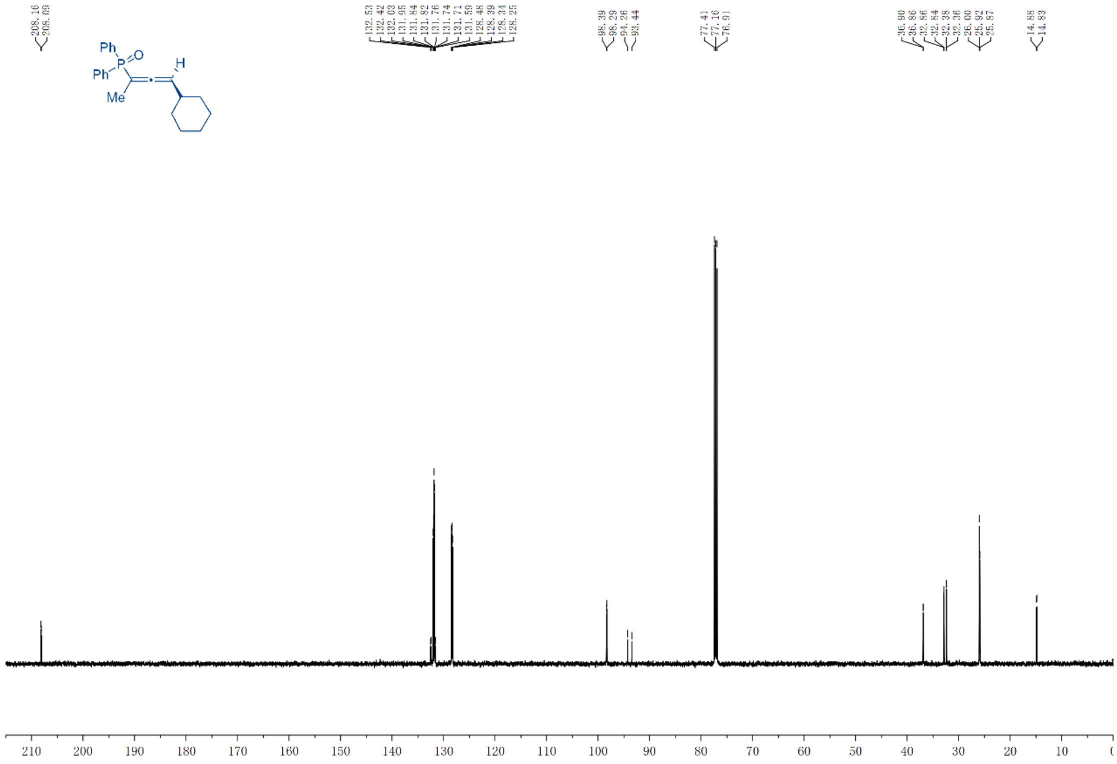

^{31}P NMR of 3f

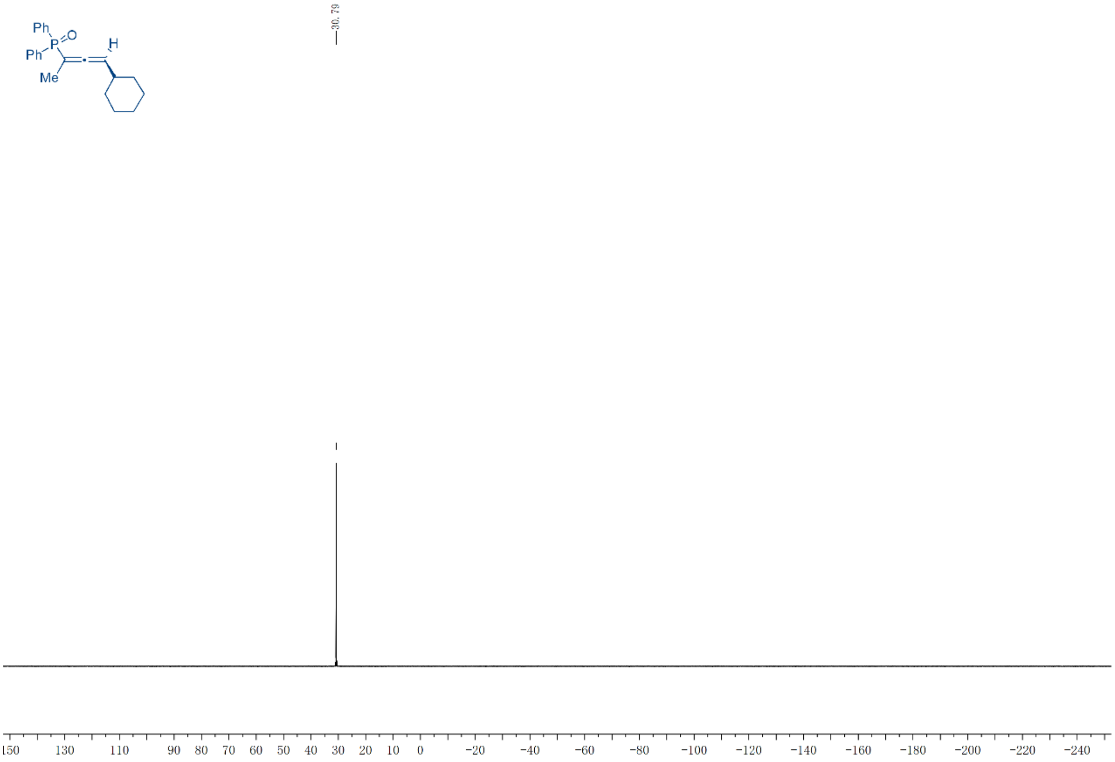

Supplementary Figure 17. ^{1}H NMR, ^{13}C NMR, and ^{31}P NMR spectra of compound **3f**

^{1}H NMR of 3g

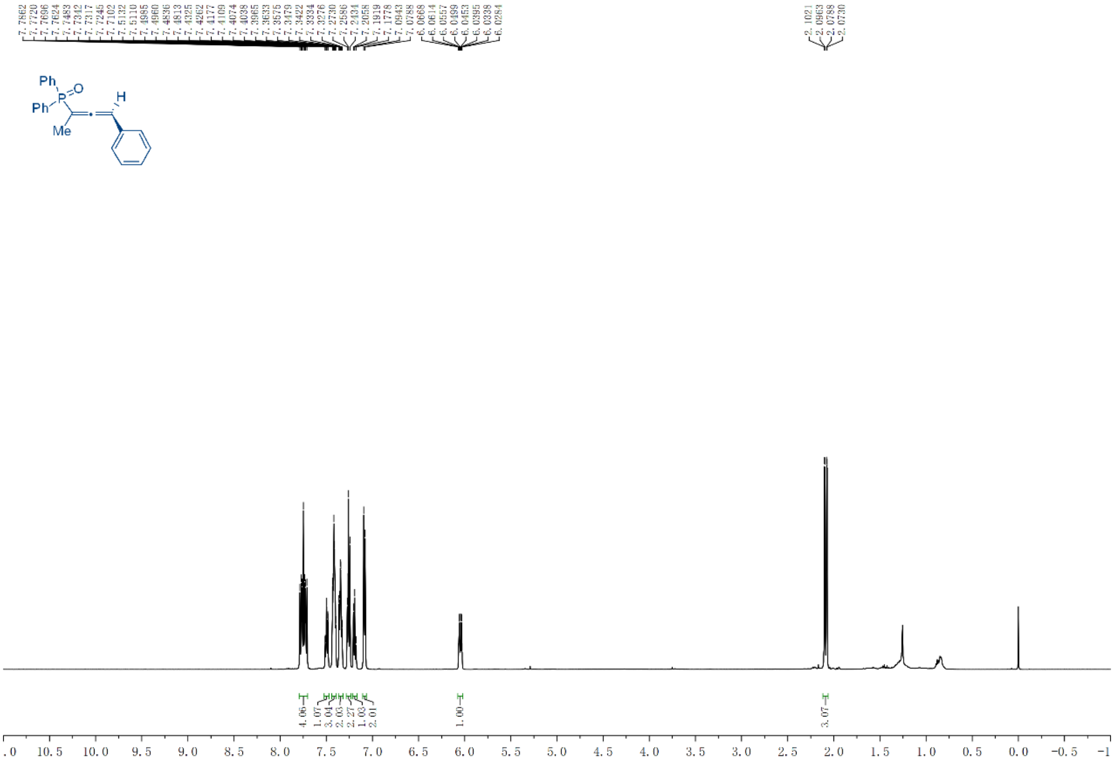

^{13}C NMR of 3g

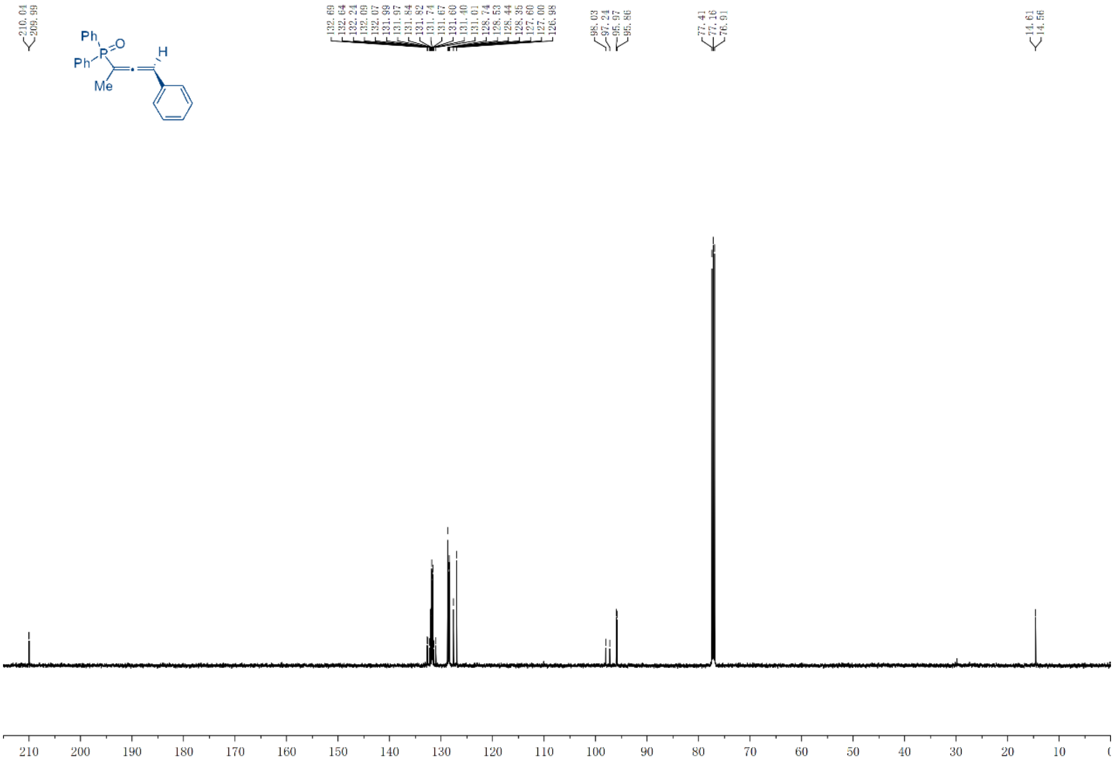

^{31}P NMR of 3g

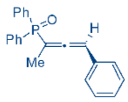

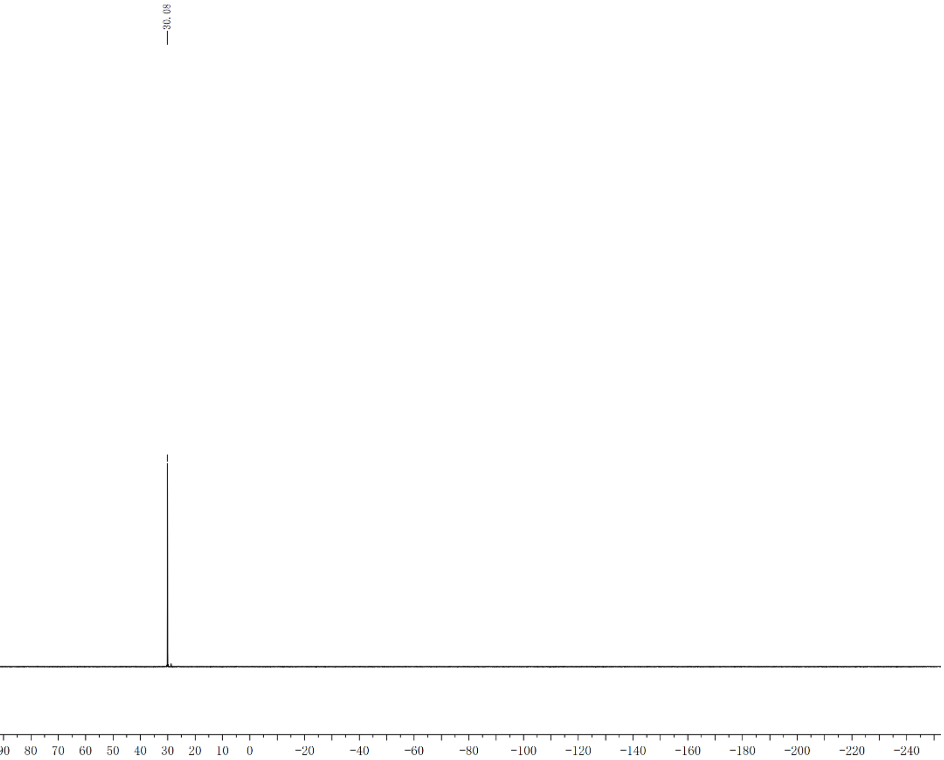

Supplementary Figure 18. ^{1}H NMR, ^{13}C NMR, and ^{31}P NMR spectra of compound **3g**

^{1}H NMR of 3h

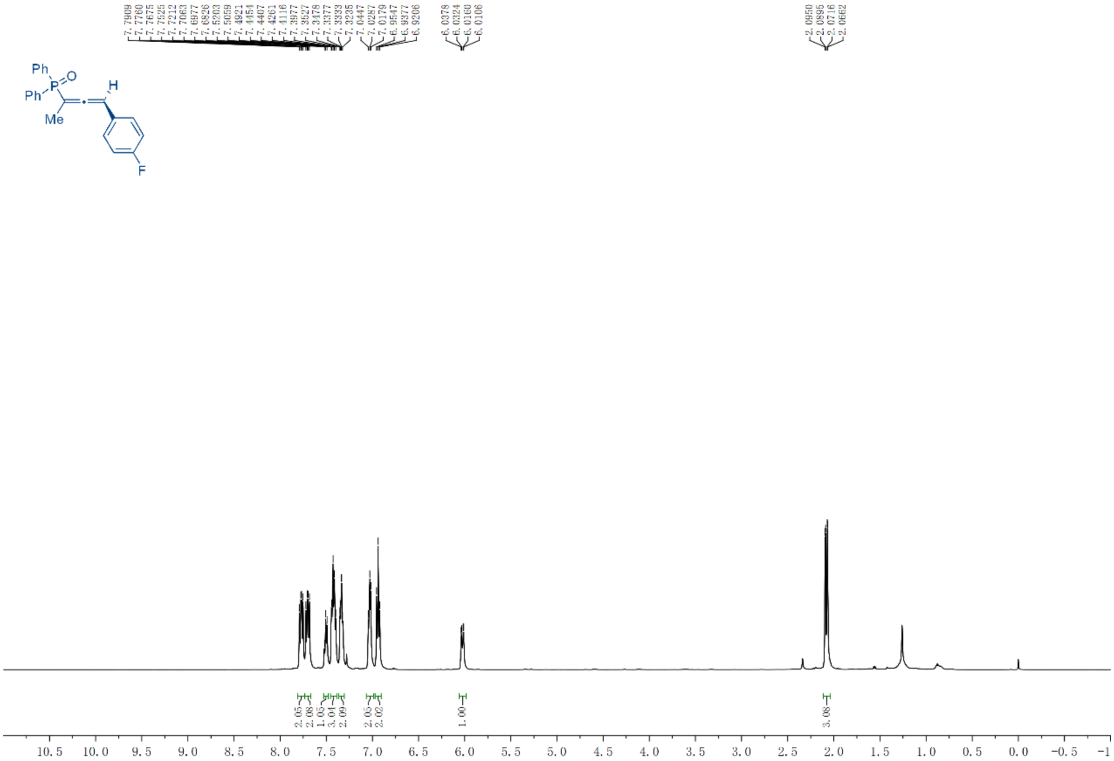

^{13}C NMR of 3h

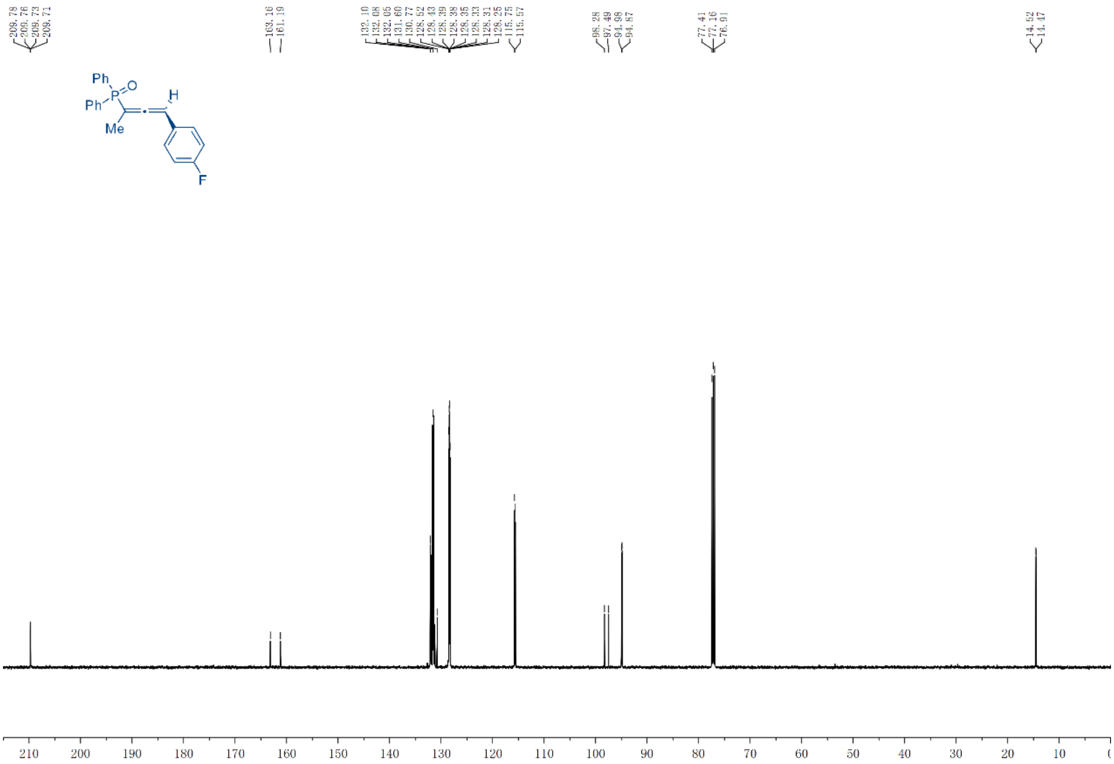

^{31}P NMR of 3h

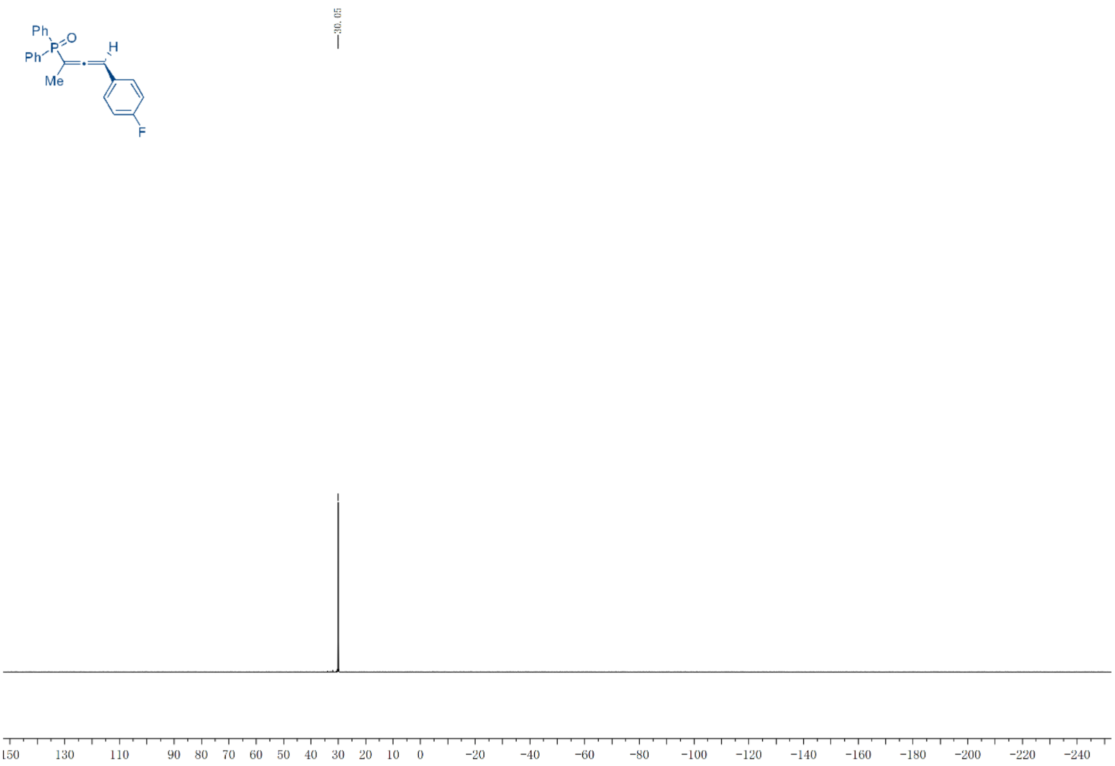

^{19}F NMR of 3h

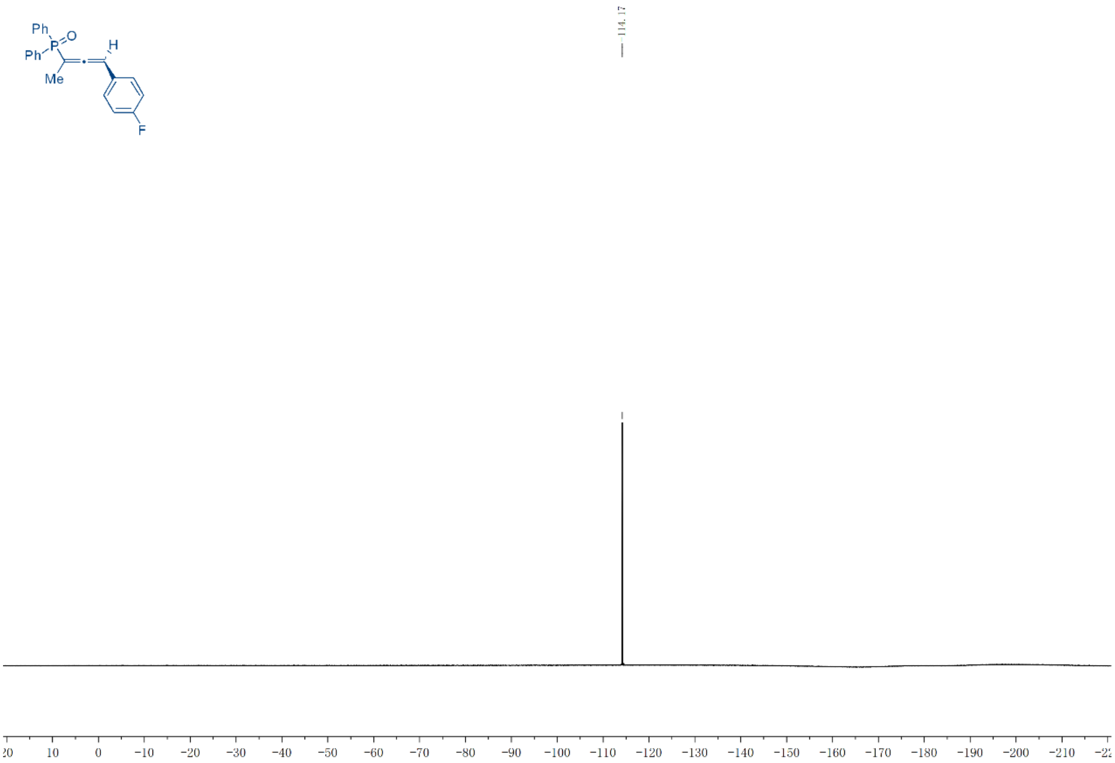

Supplementary Figure 19. ^{1}H NMR, ^{13}C NMR, ^{31}P NMR, and ^{19}F NMR spectra of compound **3h**

^{1}H NMR of 3i

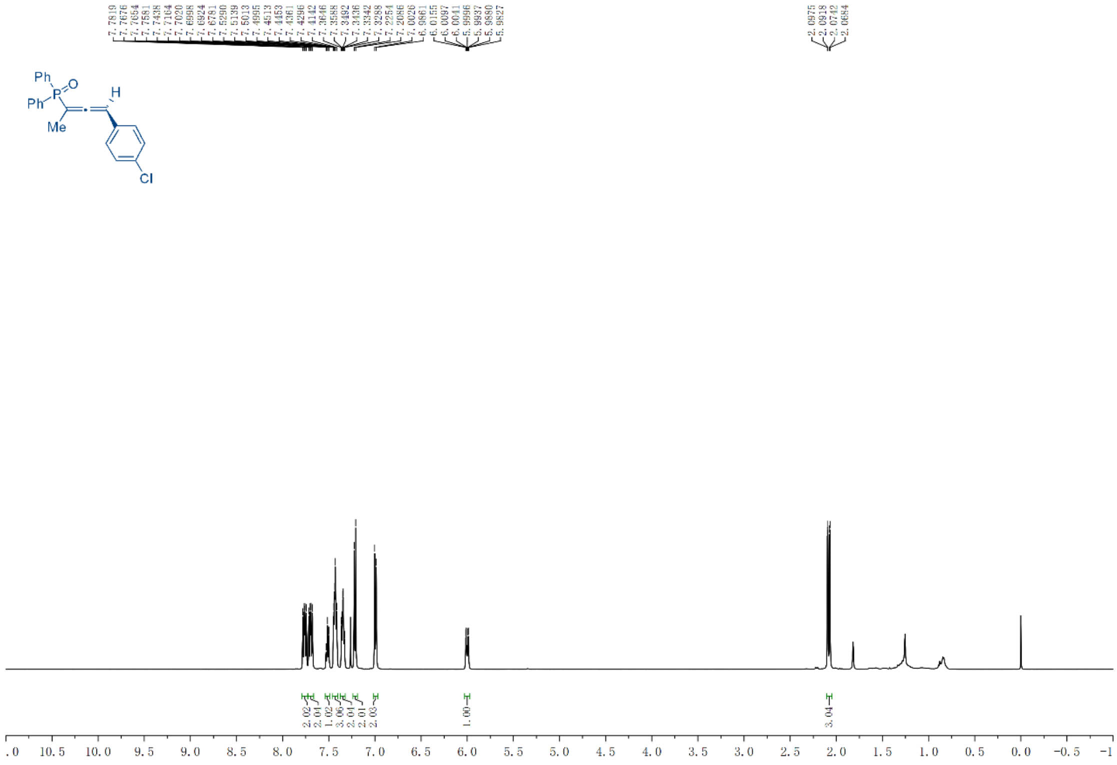

^{13}C NMR of 3i

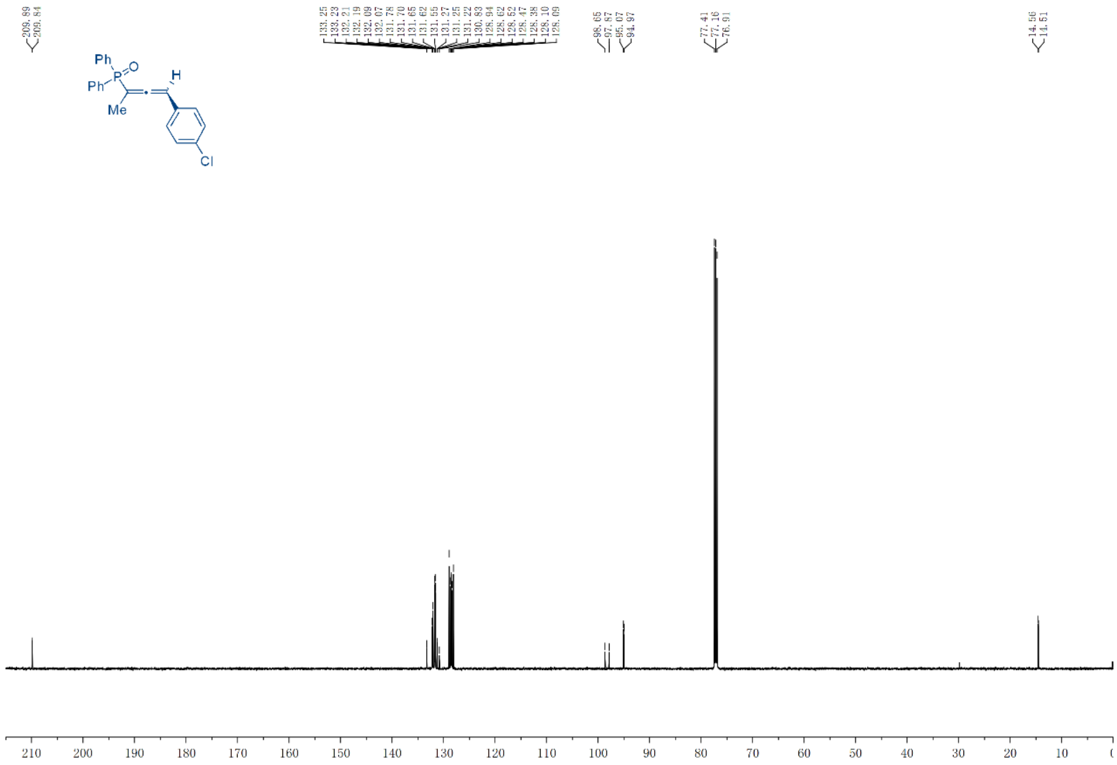

^{31}P NMR of 3i

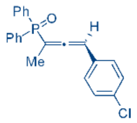

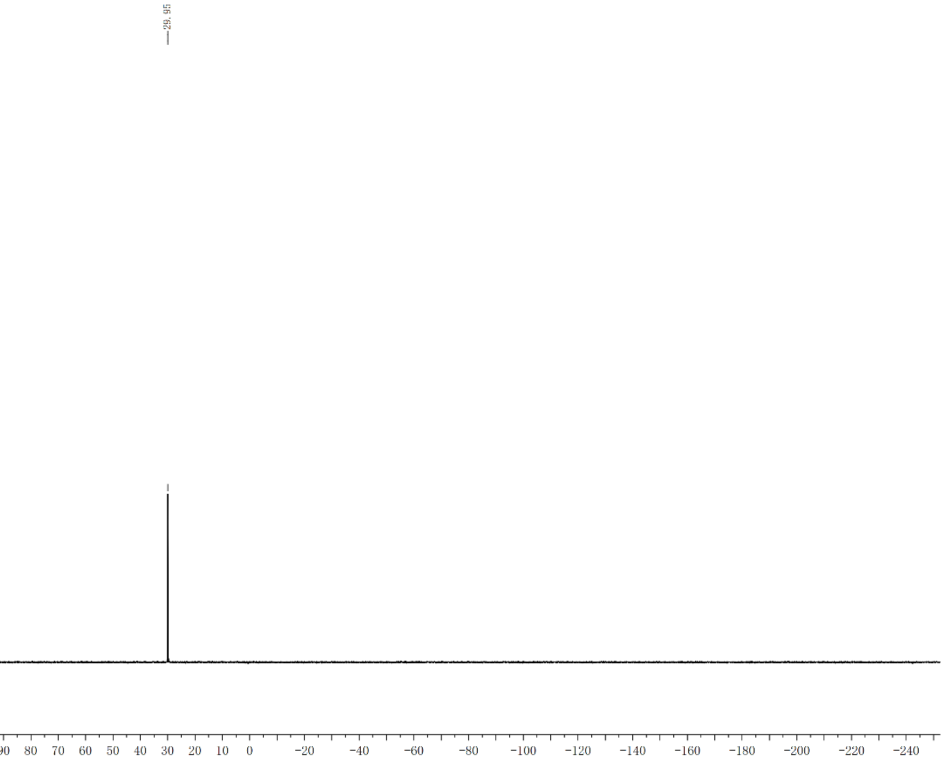

Supplementary Figure 20. ^{1}H NMR, ^{13}C NMR, and ^{31}P NMR spectra of compound **3i**

^{1}H NMR of 3j

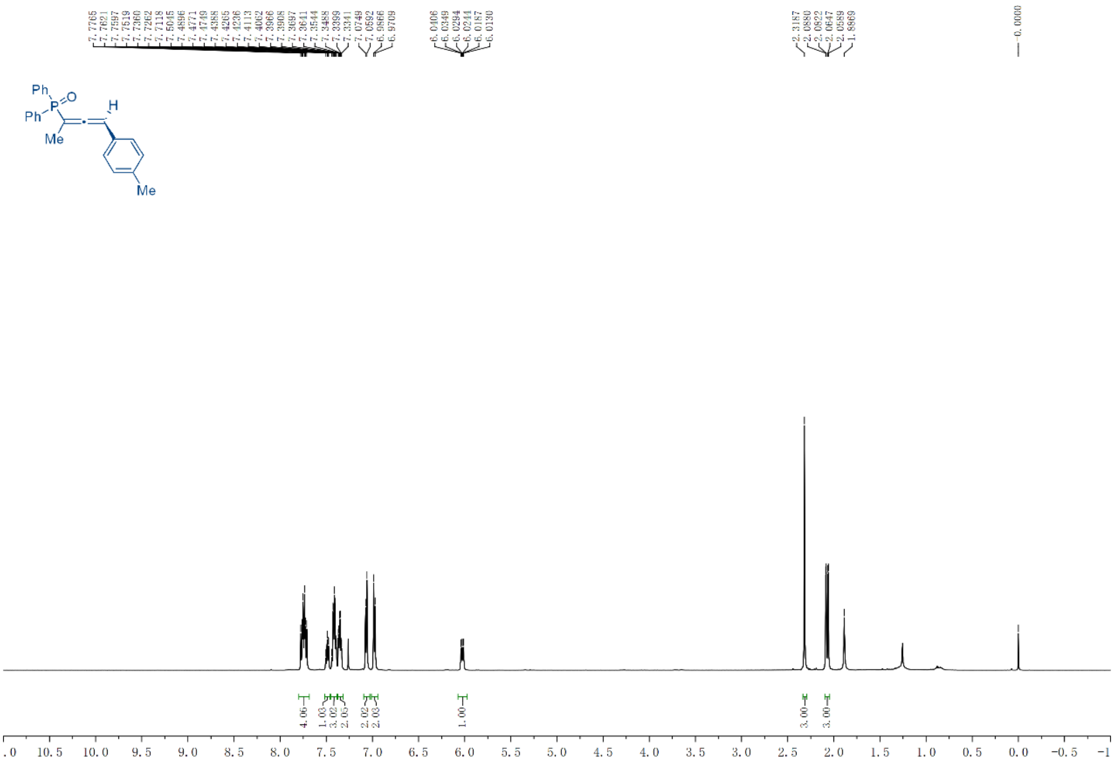

^{13}C NMR of 3j

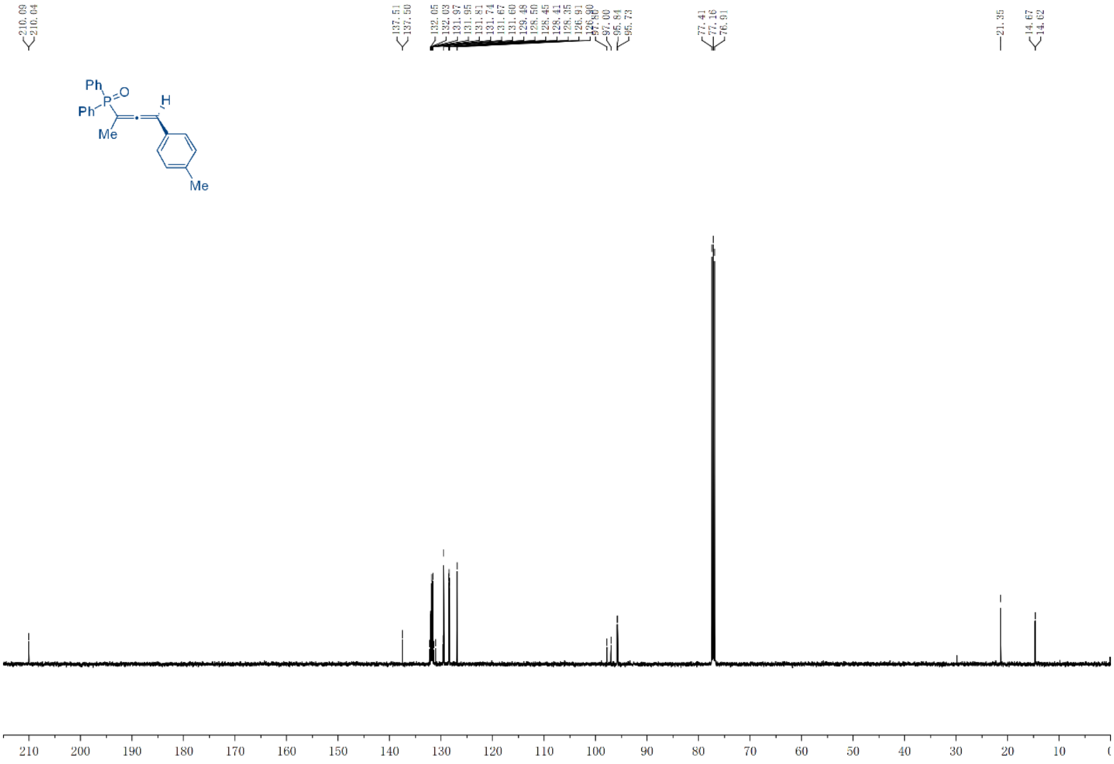

^{31}P NMR of 3j

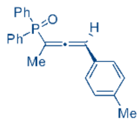

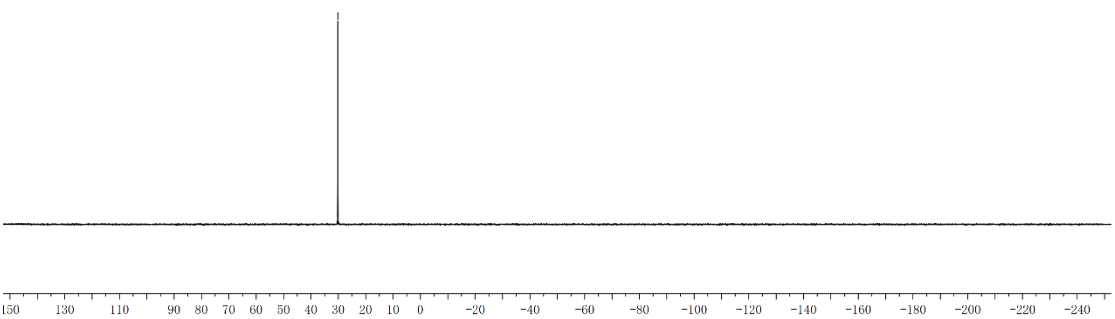

Supplementary Figure 21. ^{1}H NMR, ^{13}C NMR, and ^{31}P NMR spectra of compound **3j**

^{1}H NMR of 3k

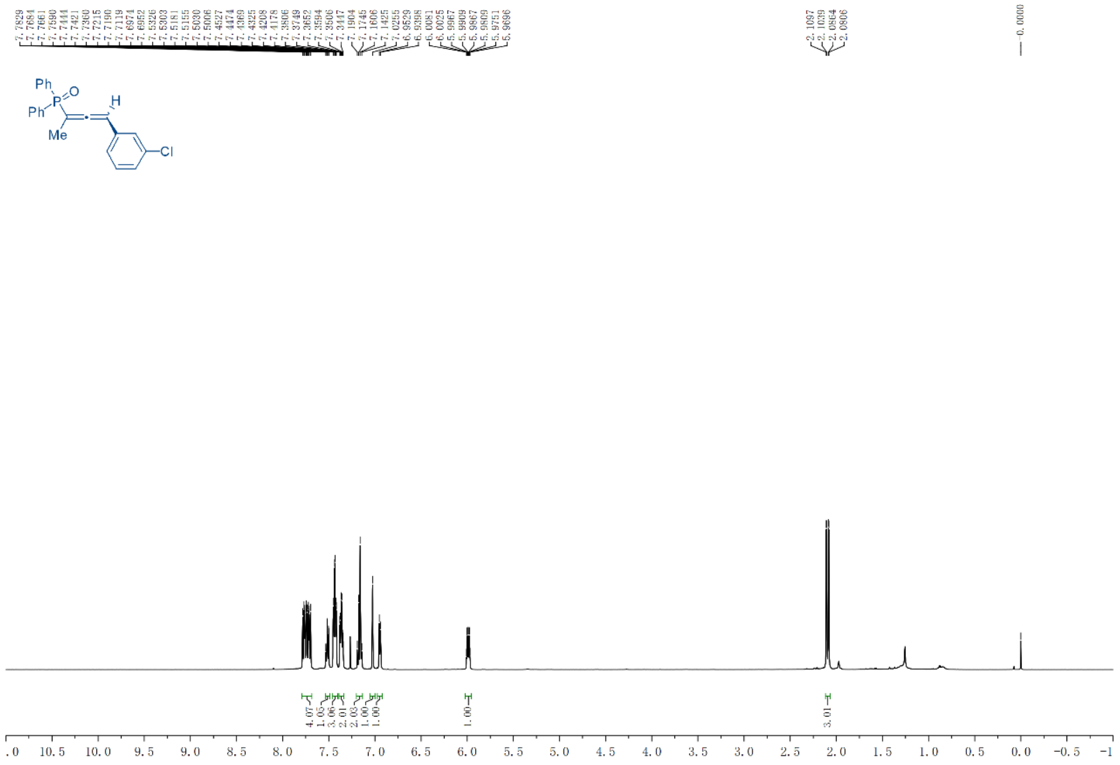

^{13}C NMR of 3k

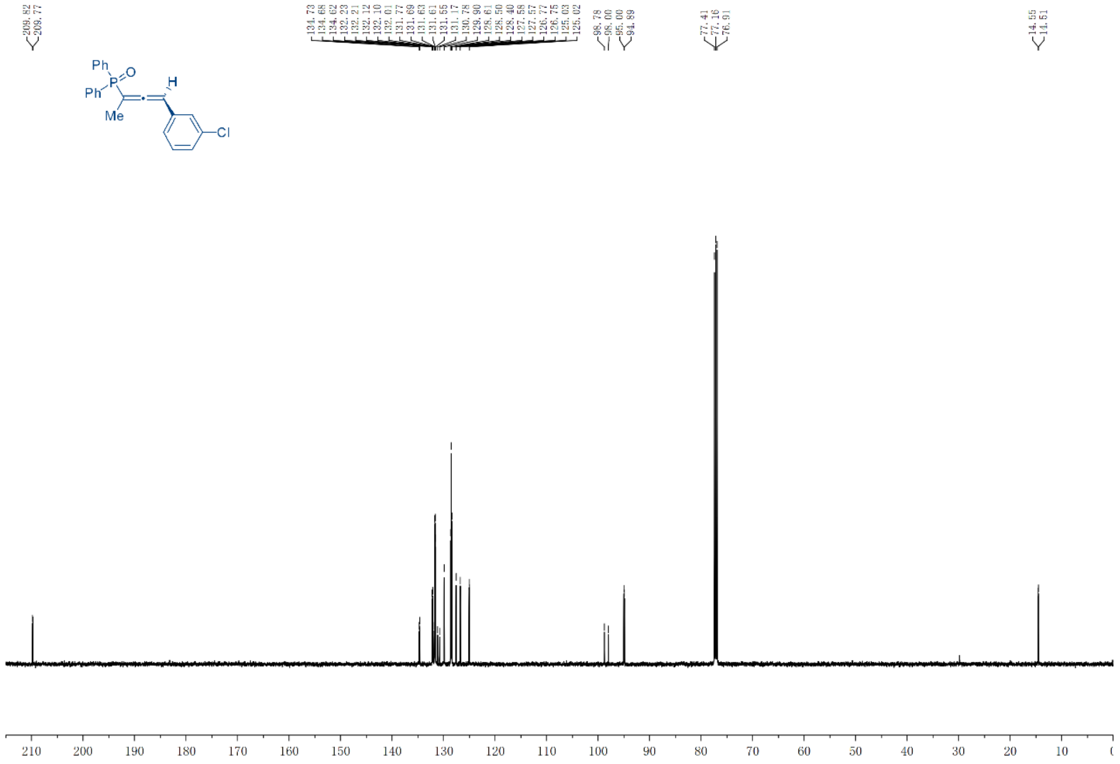

^{31}P NMR of 3k

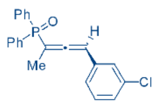

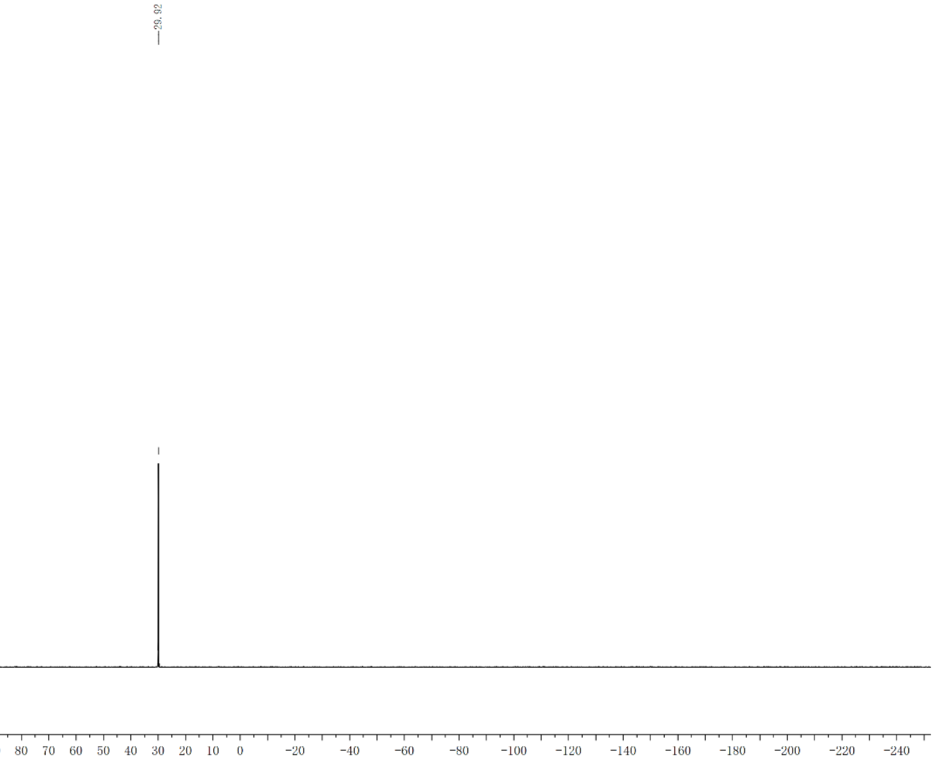

Supplementary Figure 22. ^{1}H NMR, ^{13}C NMR, and ^{31}P NMR spectra of compound **3k**

1H NMR of 3l

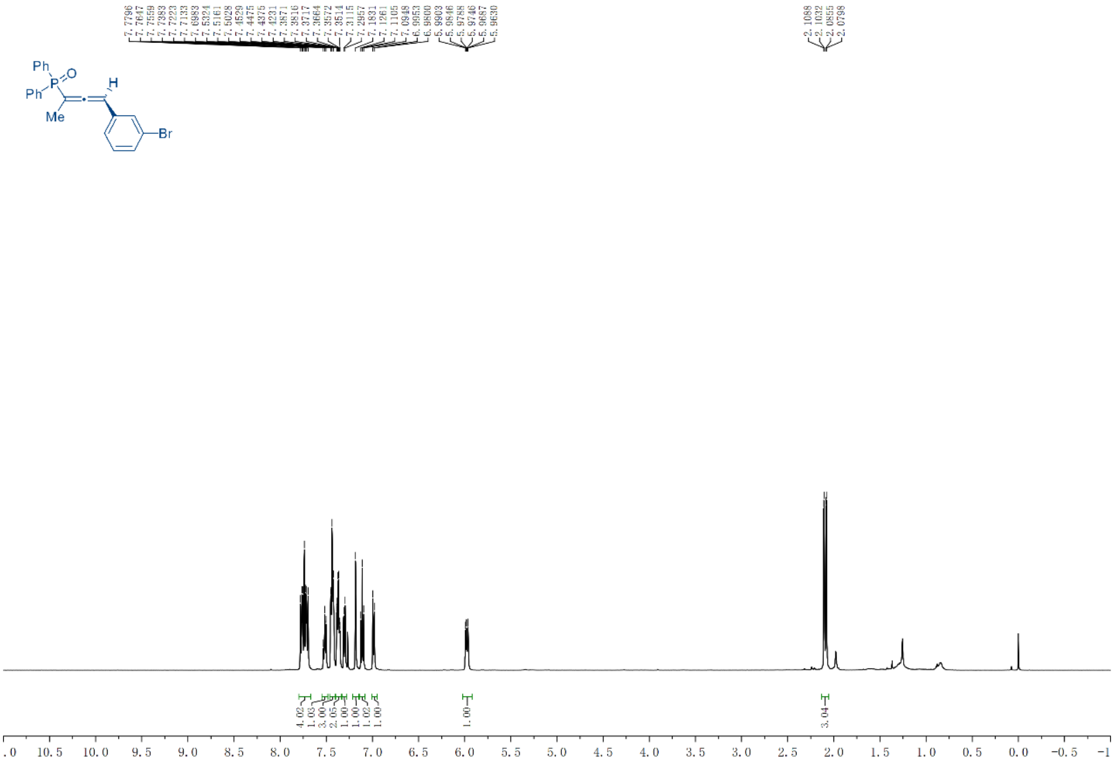

13C NMR of 3l

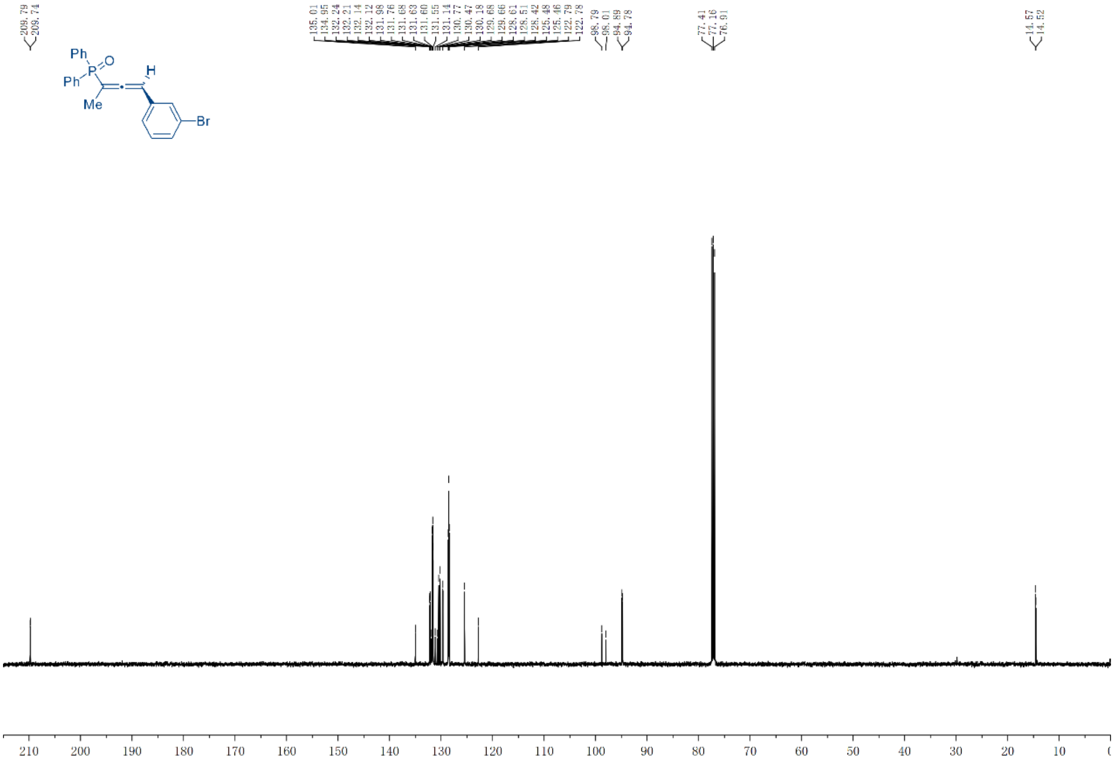

^{31}P NMR of 3l

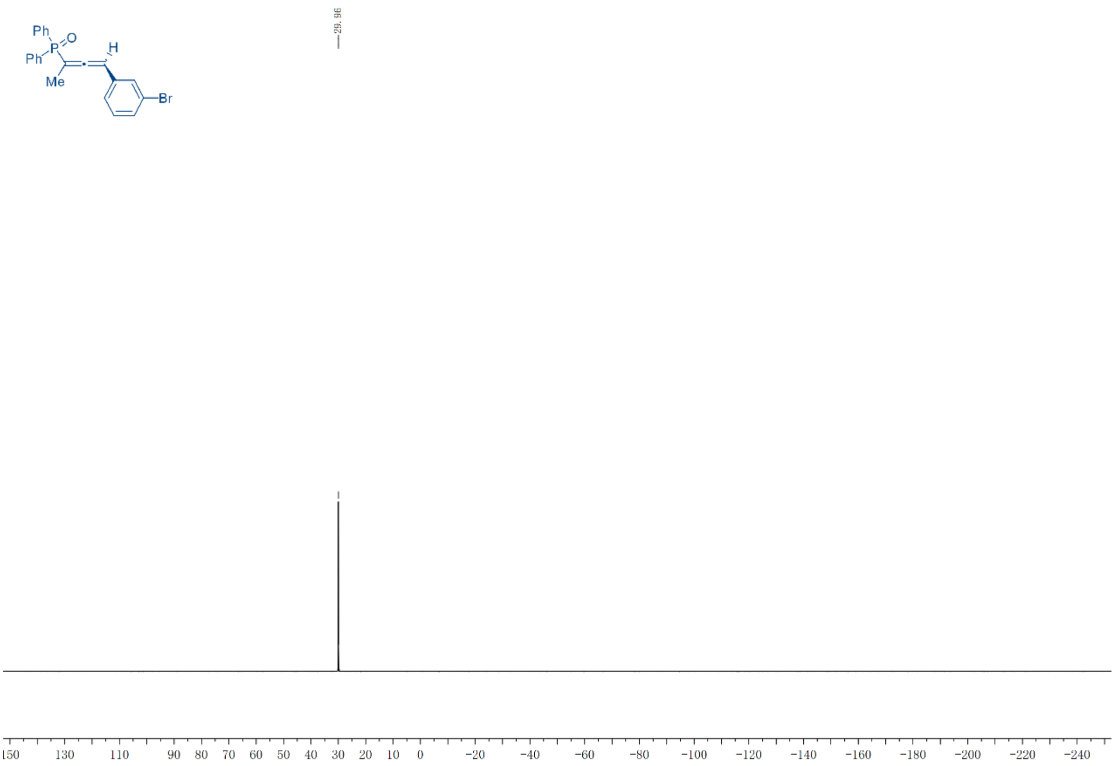

Supplementary Figure 23. ^{1}H NMR, ^{13}C NMR, and ^{31}P NMR spectra of compound **3l**

^{1}H NMR of 3m

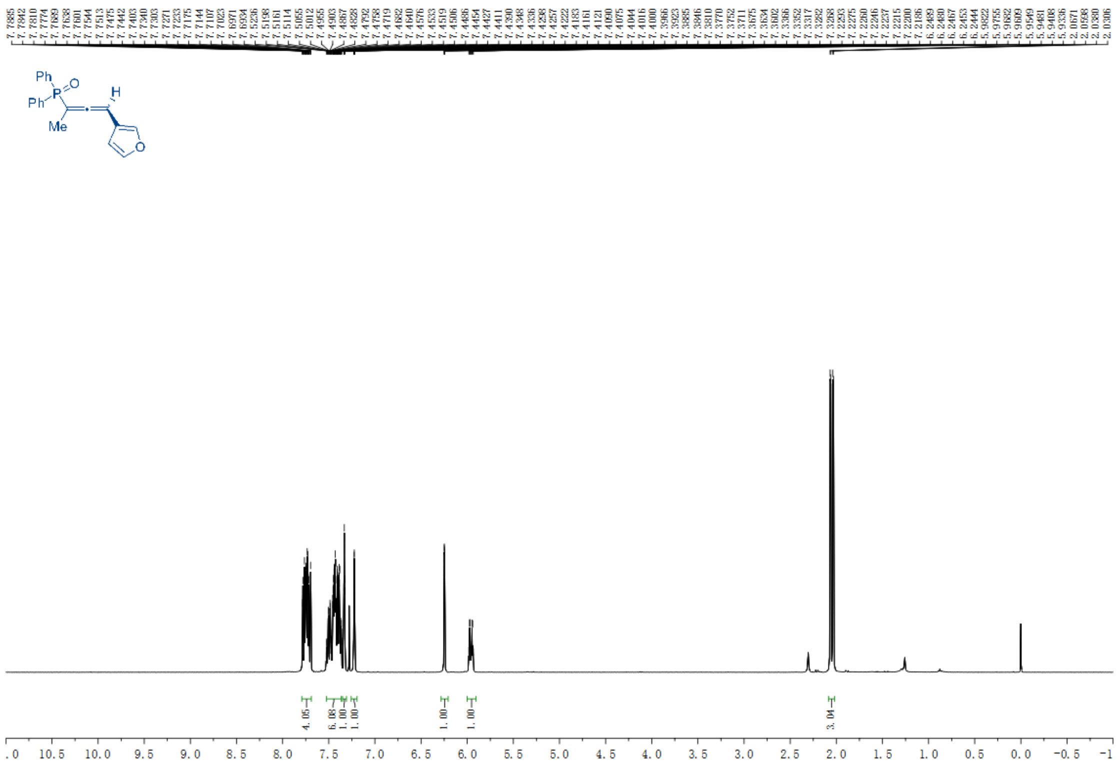

^{13}C NMR of 3m

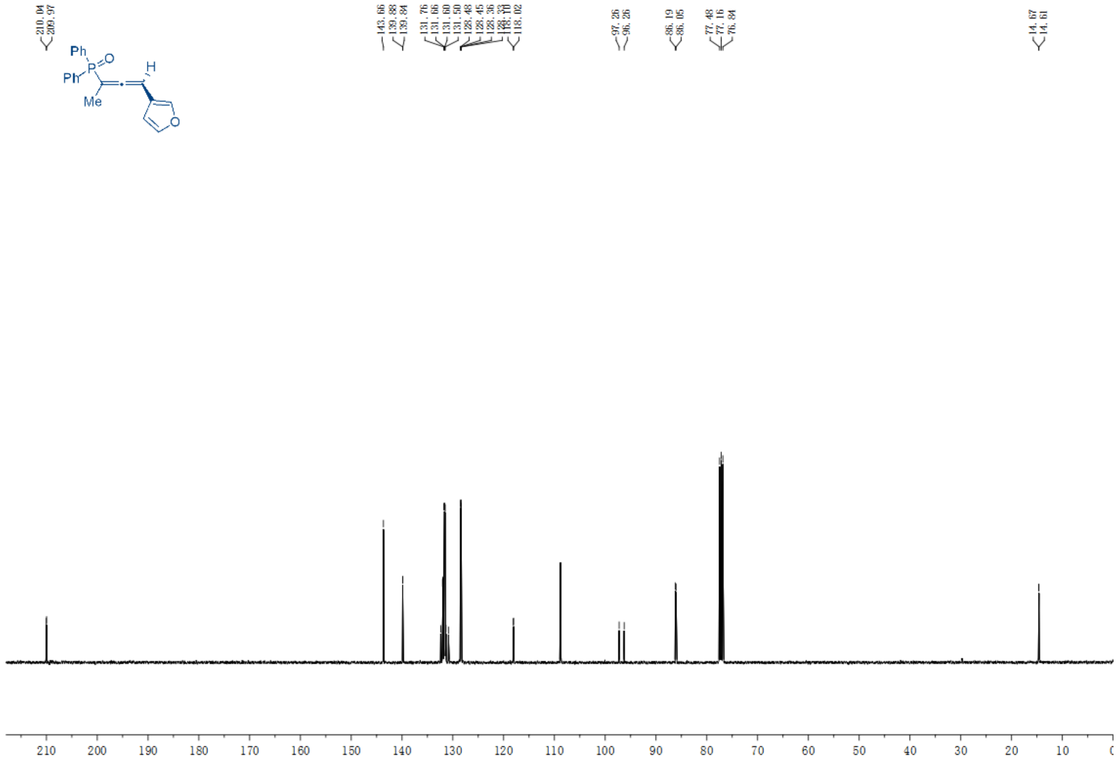

^{31}P NMR of 3m

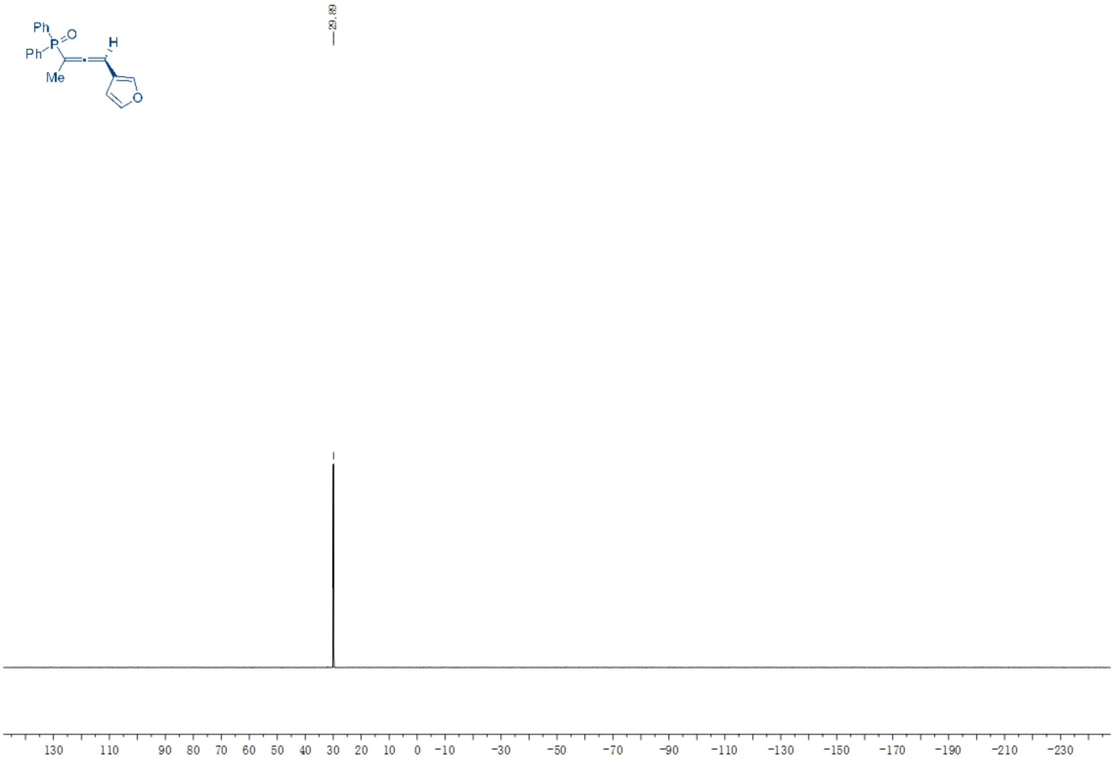

Supplementary Figure 24. ^{1}H NMR, ^{13}C NMR, and ^{31}P NMR spectra of compound **3m**

^{1}H NMR of 3n

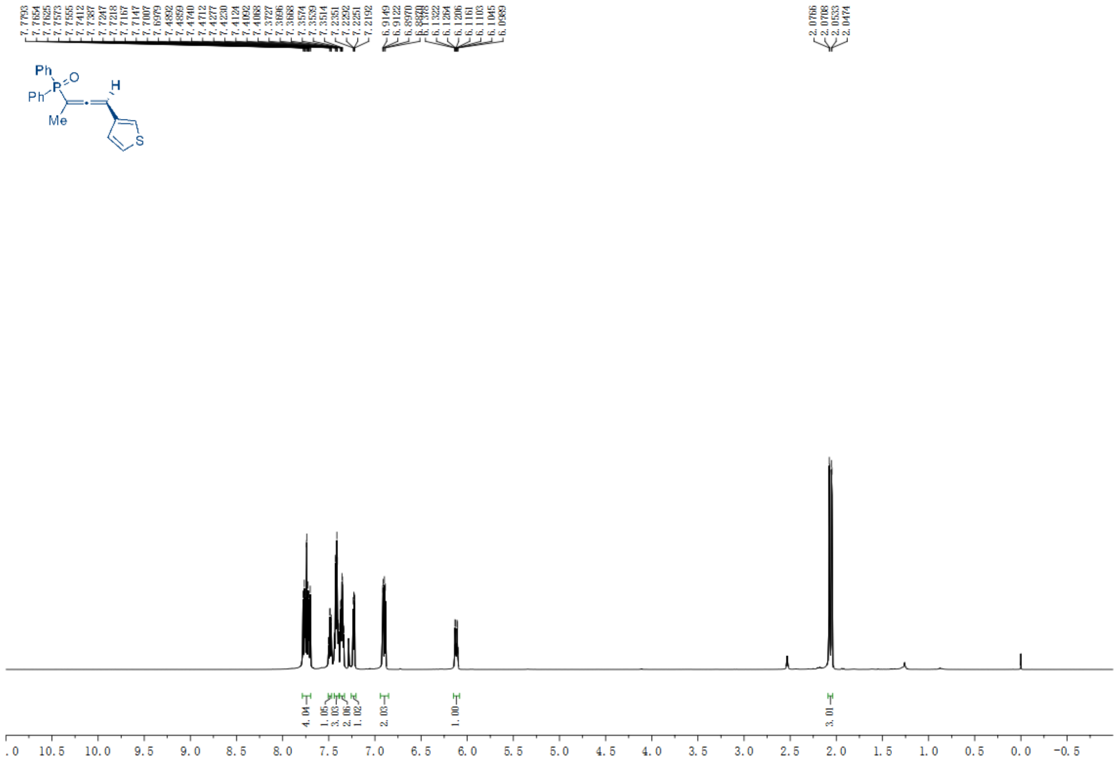

^{13}C NMR of 3n

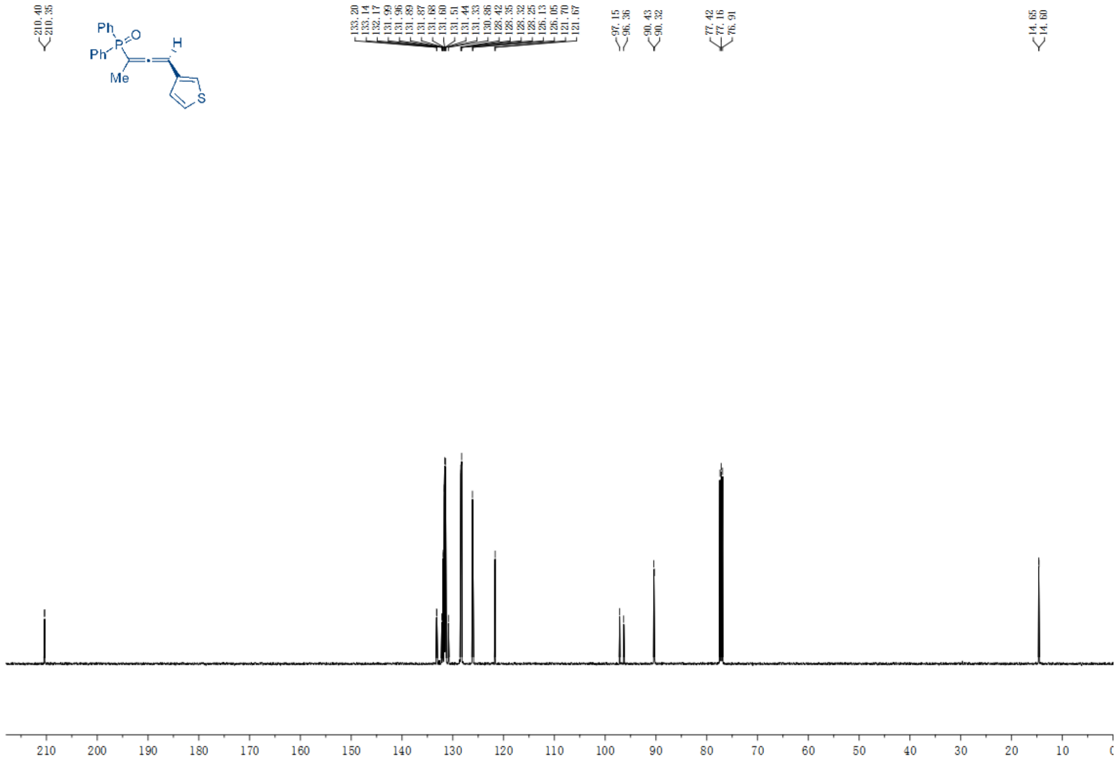

^{31}P NMR of 3n

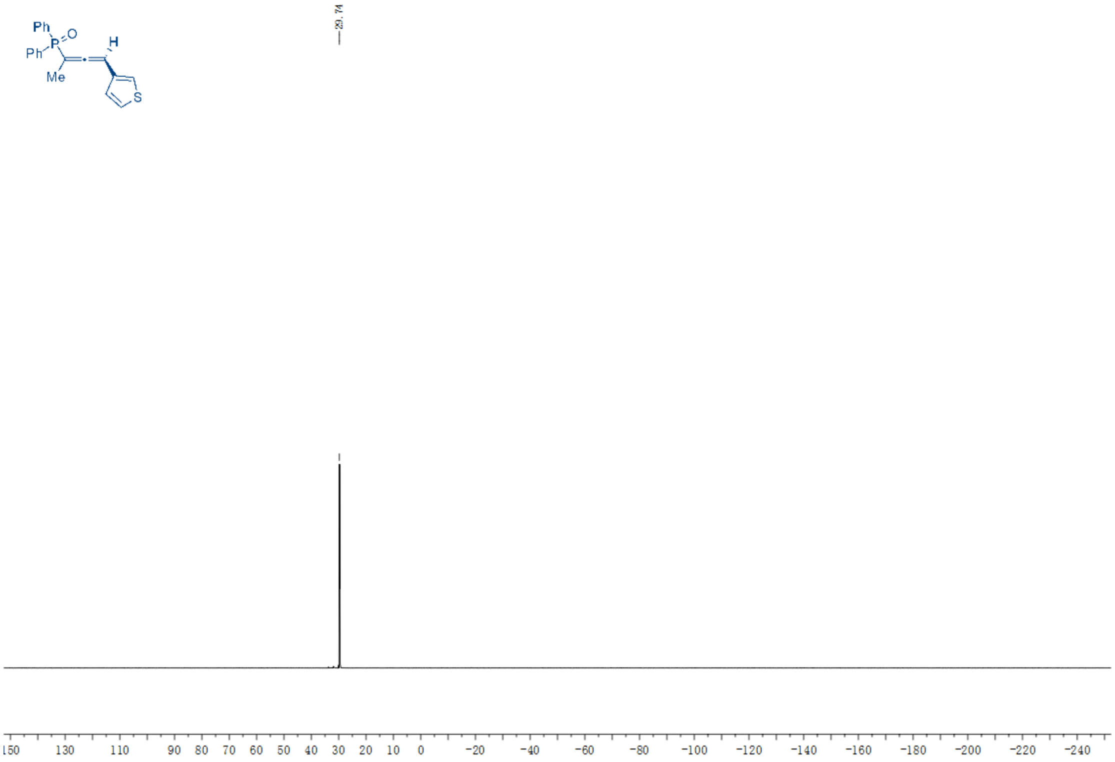

Supplementary Figure 25. ^{1}H NMR, ^{13}C NMR, and ^{31}P NMR spectra of compound **3n**

^{1}H NMR of 3o

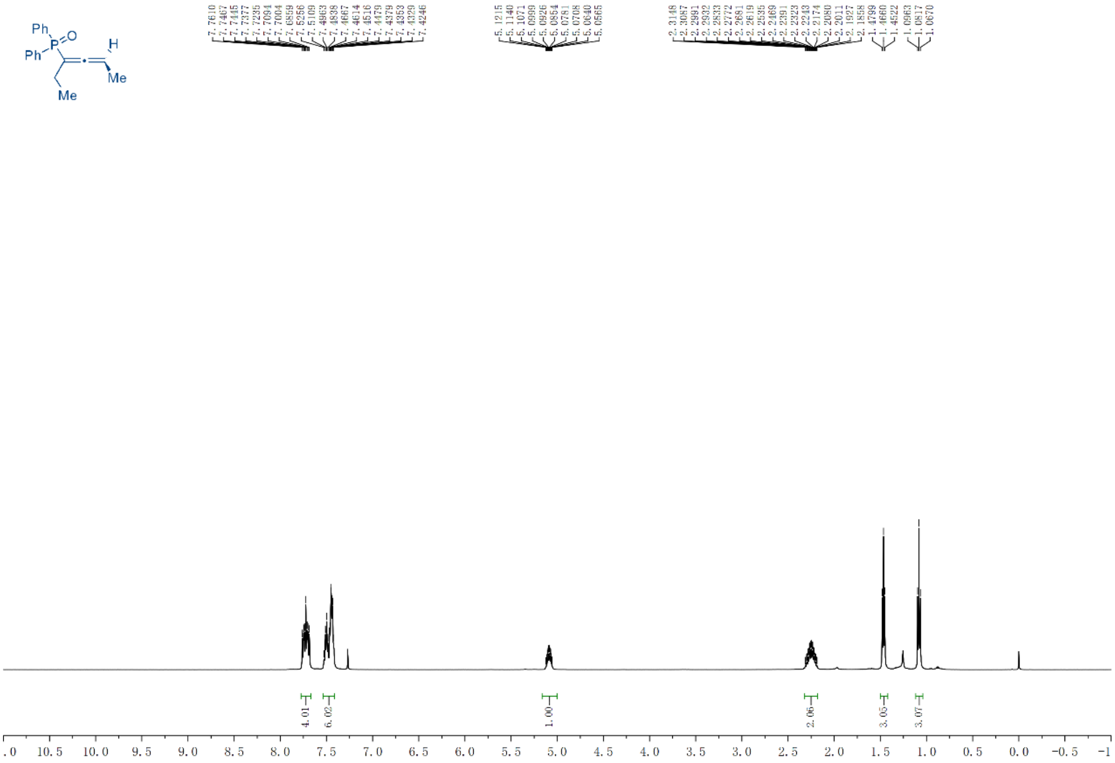

^{13}C NMR of 3o

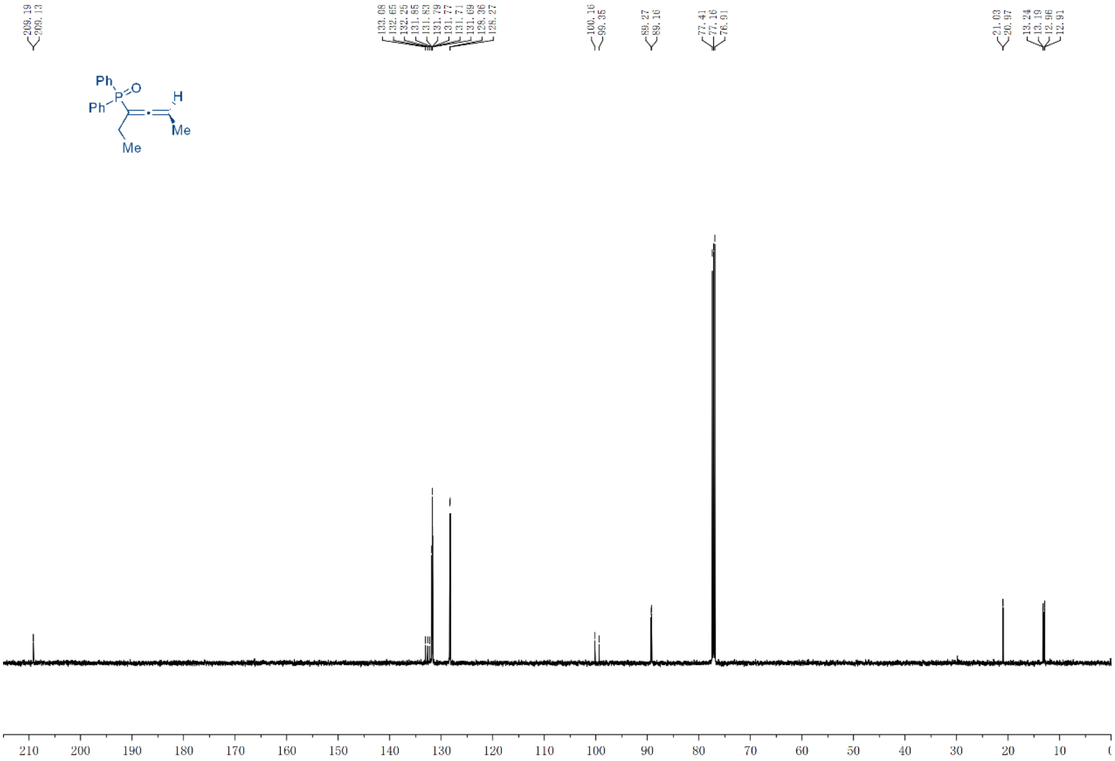

^{31}P NMR of 3o

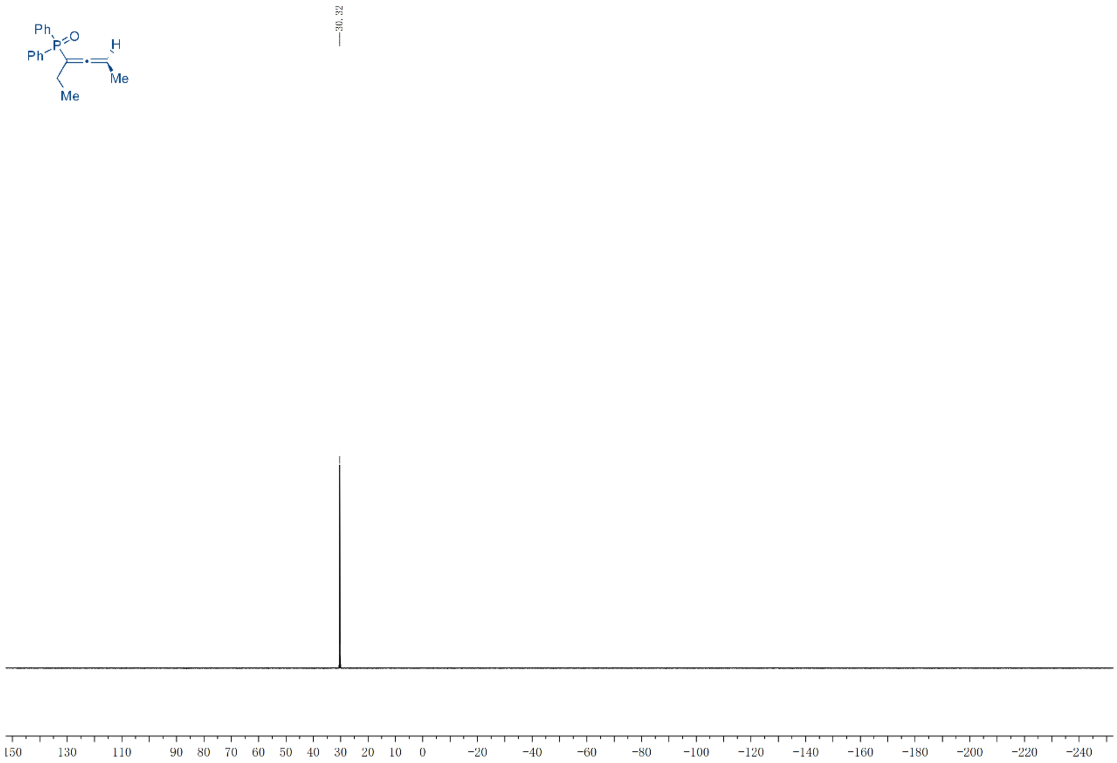

Supplementary Figure 26. ^{1}H NMR, ^{13}C NMR, and ^{31}P NMR spectra of compound **3o**

^{1}H NMR of 3p

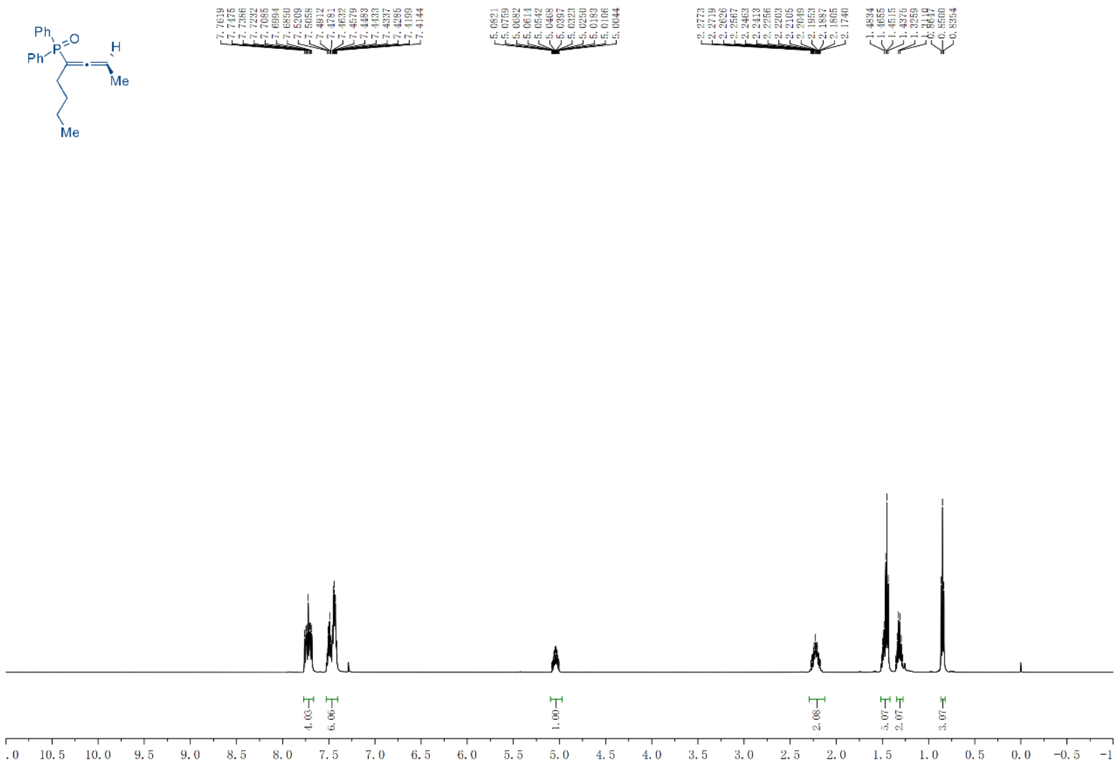

^{13}C NMR of 3p

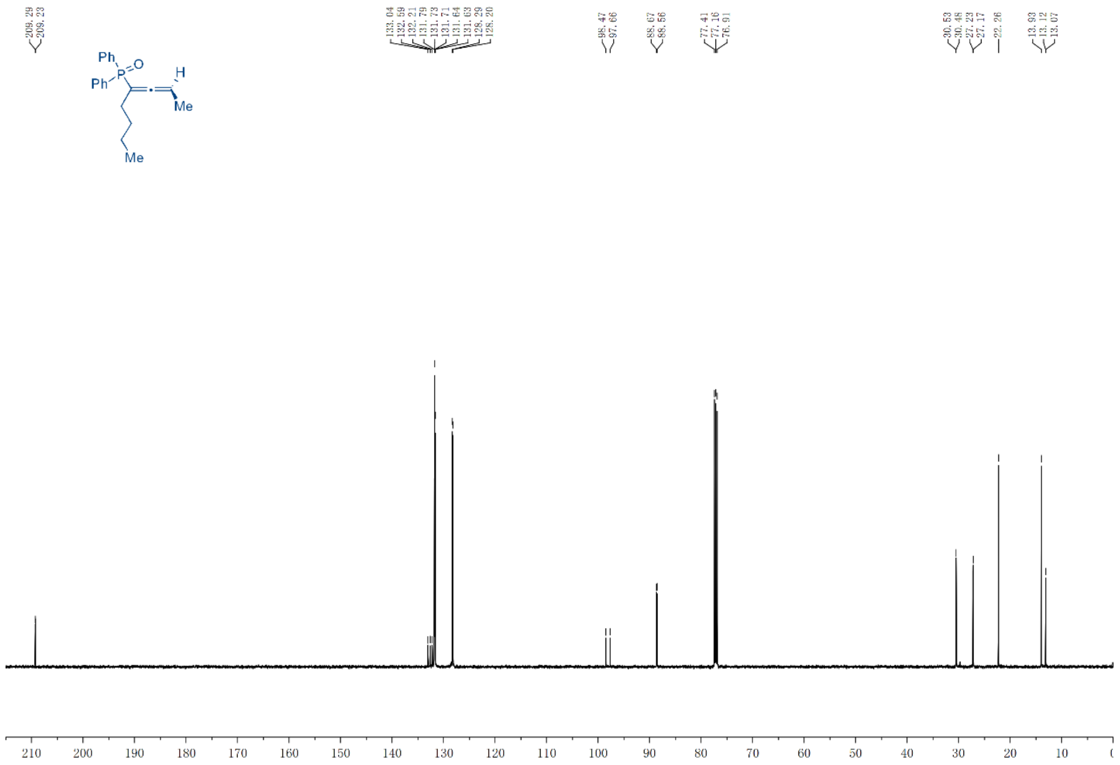

^{31}P NMR of 3p

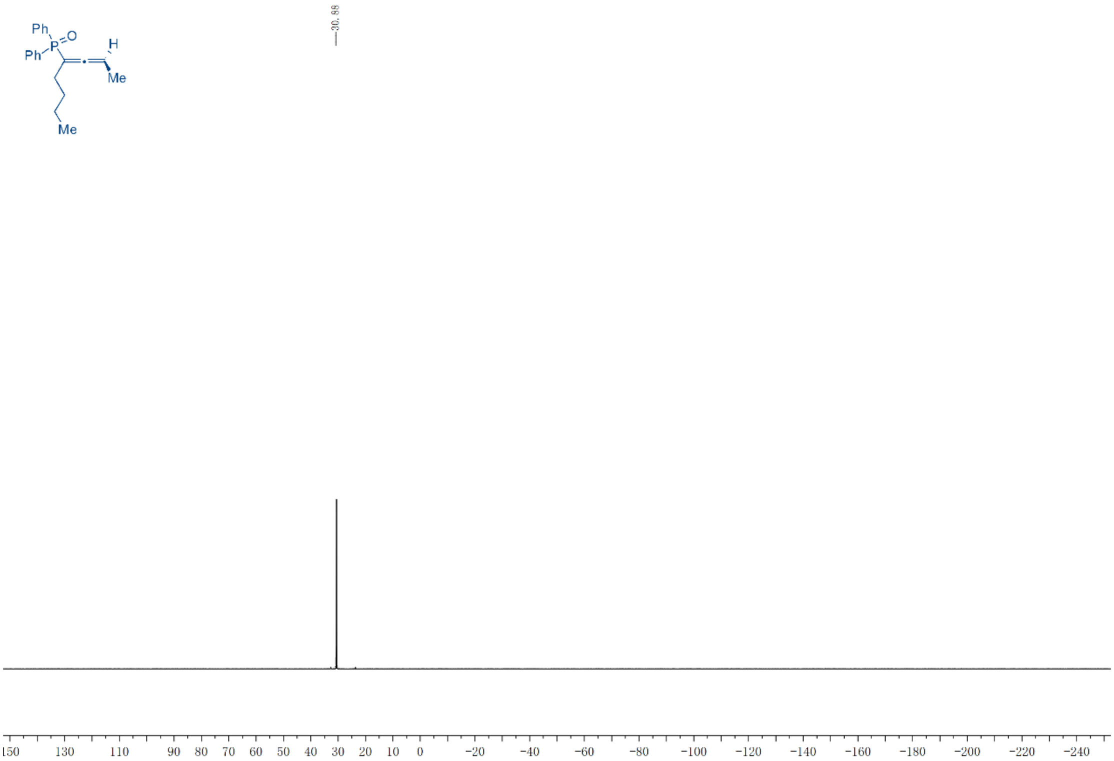

Supplementary Figure 27. ^{1}H NMR, ^{13}C NMR, and ^{31}P NMR spectra of compound **3p**

^{1}H NMR of 3q

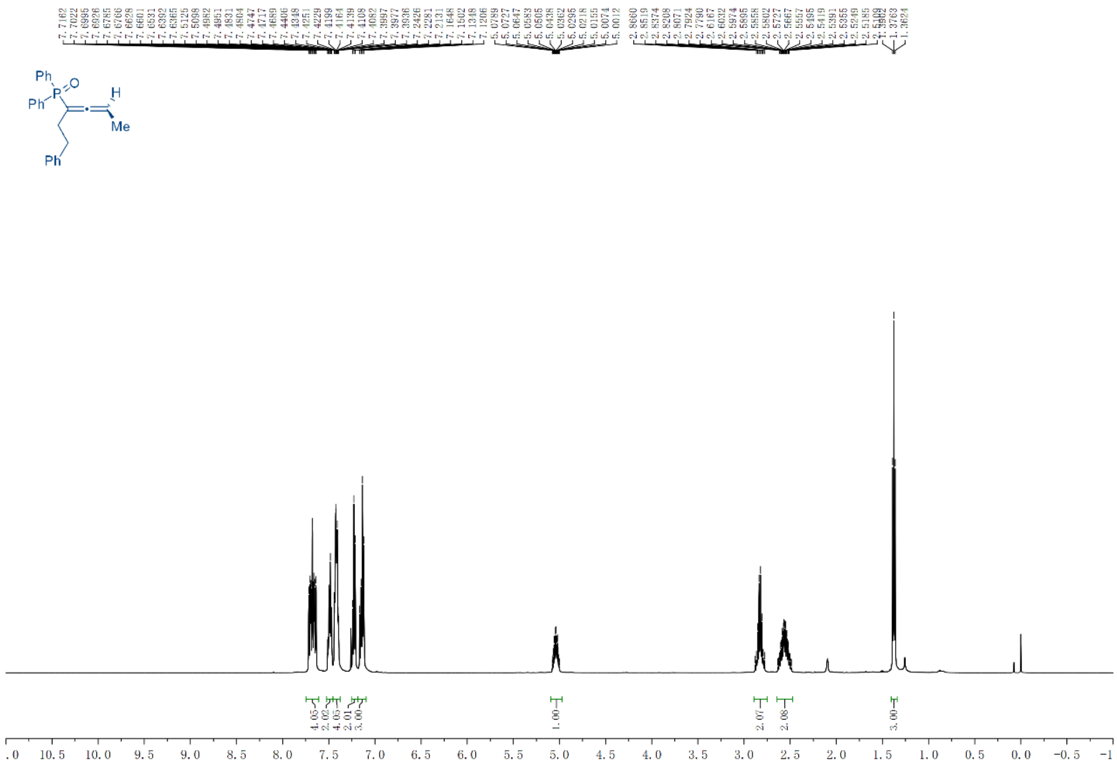

^{13}C NMR of 3q

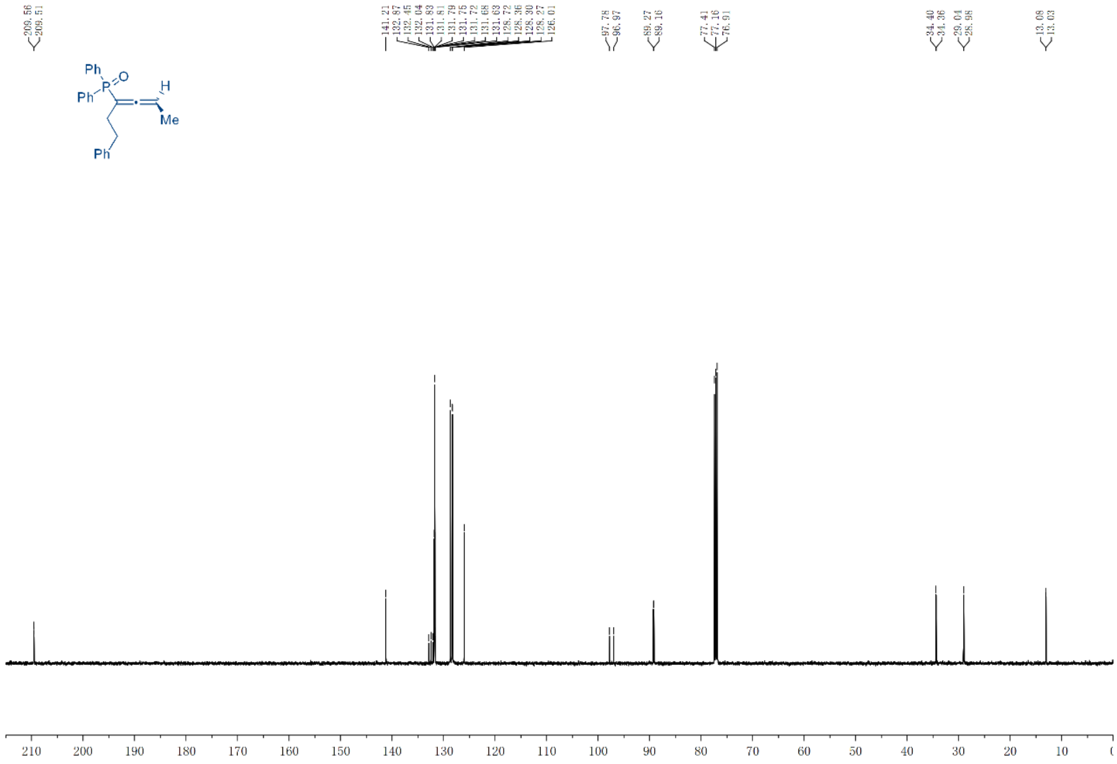

^{31}P NMR of 3q

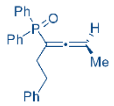

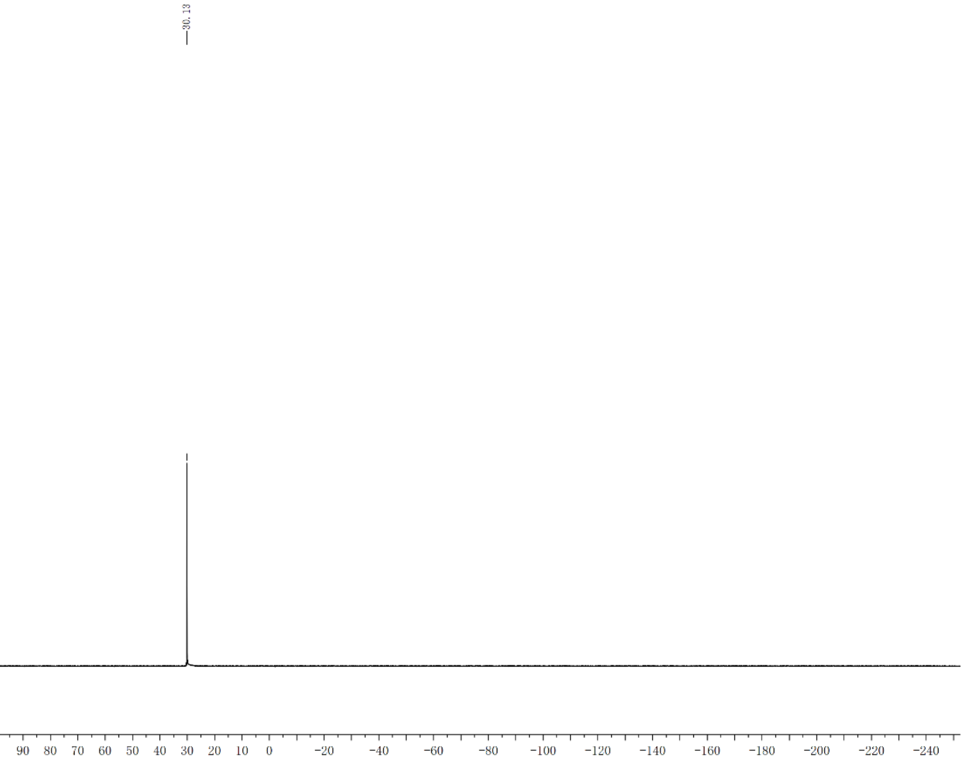

Supplementary Figure 28. ^{1}H NMR, ^{13}C NMR, and ^{31}P NMR spectra of compound **3q**

^{1}H NMR of 3r

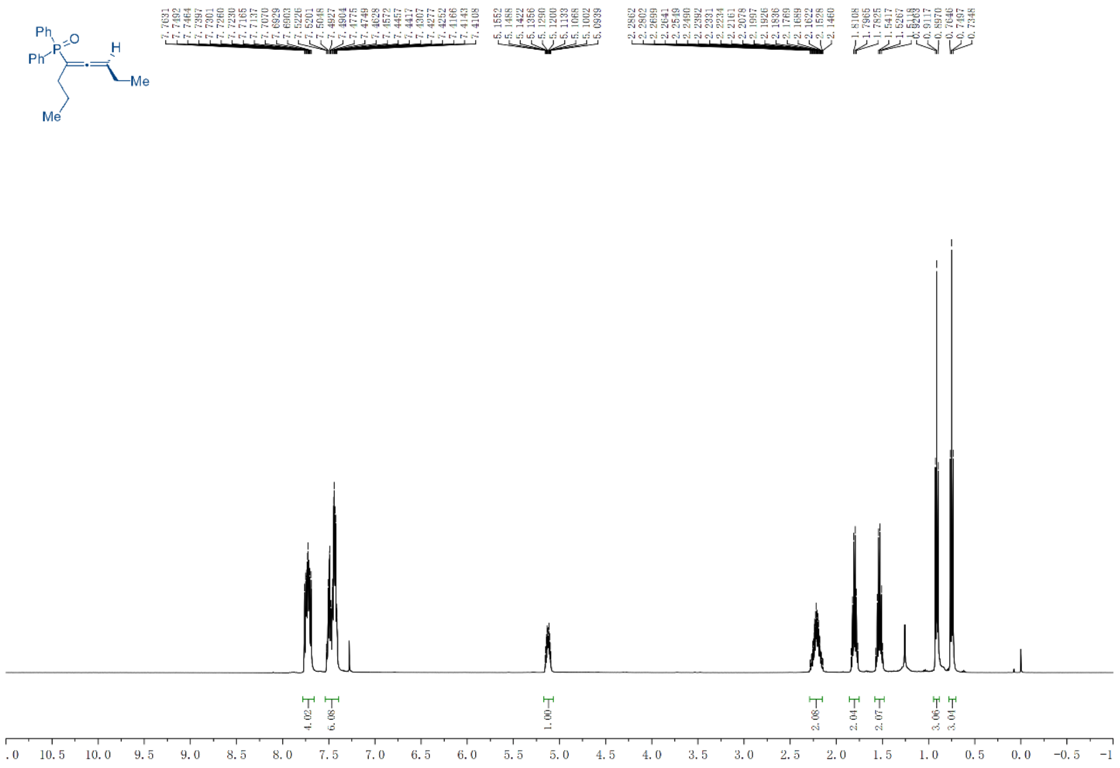

^{13}C NMR of 3r

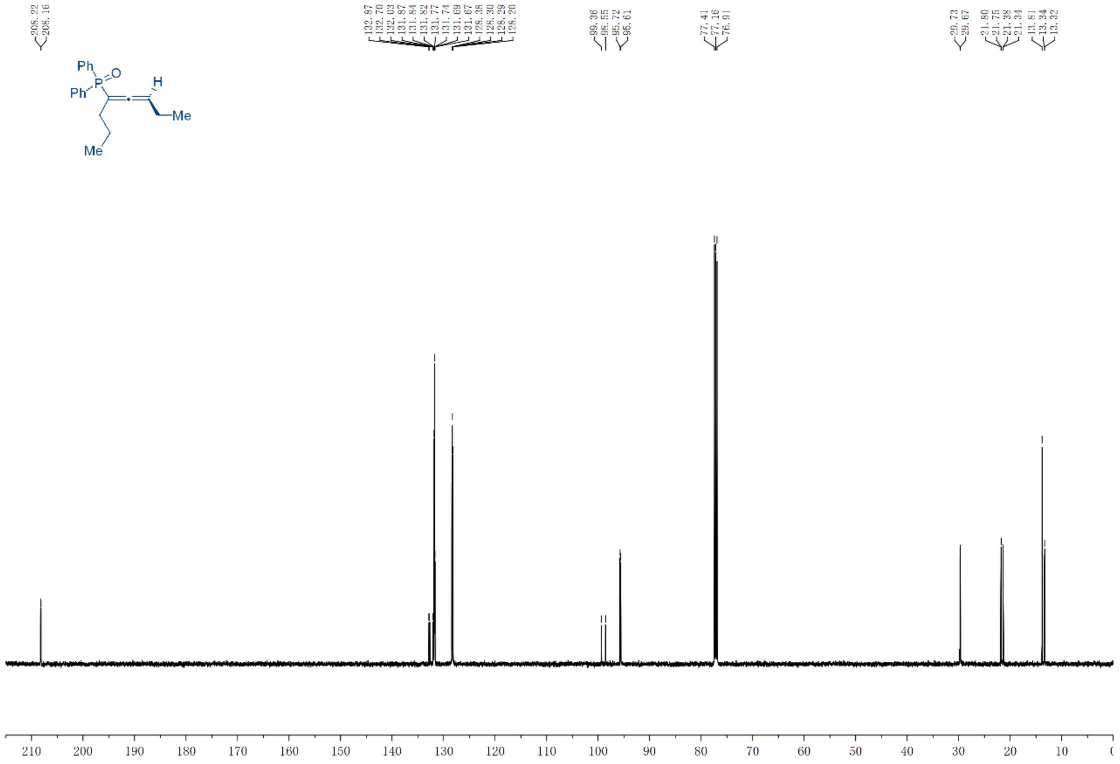

^{31}P NMR of 3r

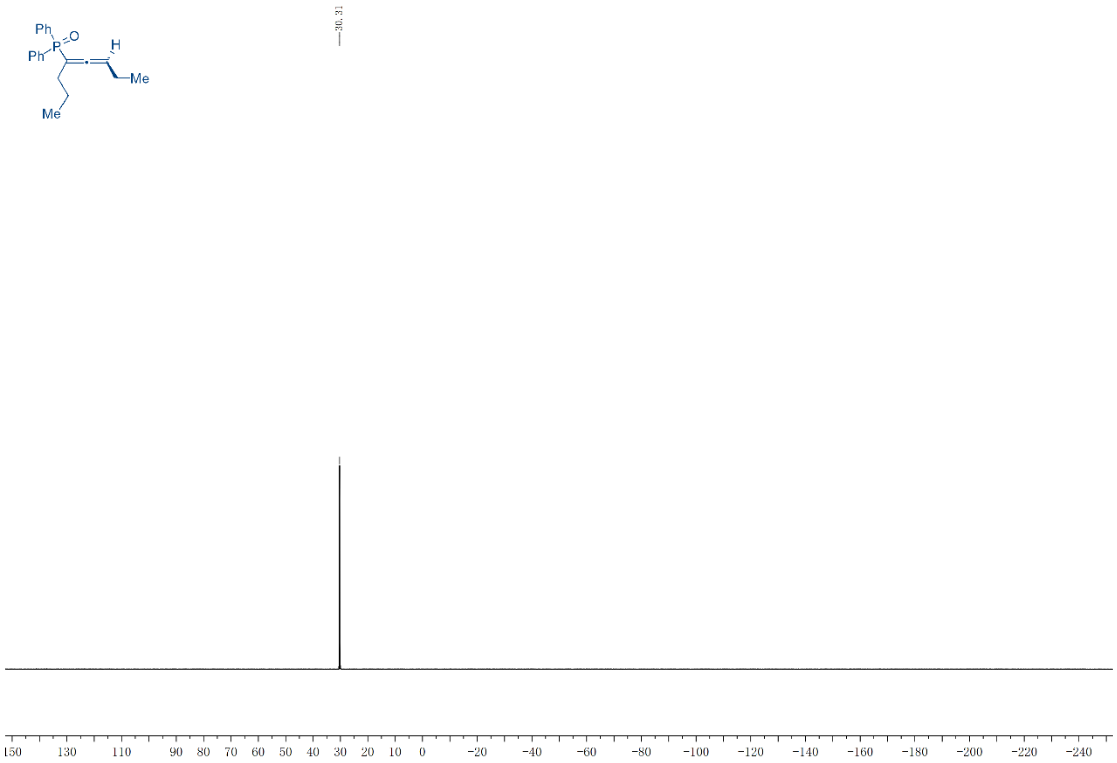

Supplementary Figure 29. ^{1}H NMR, ^{13}C NMR, and ^{31}P NMR spectra of compound **3r**

^{1}H NMR of 3s

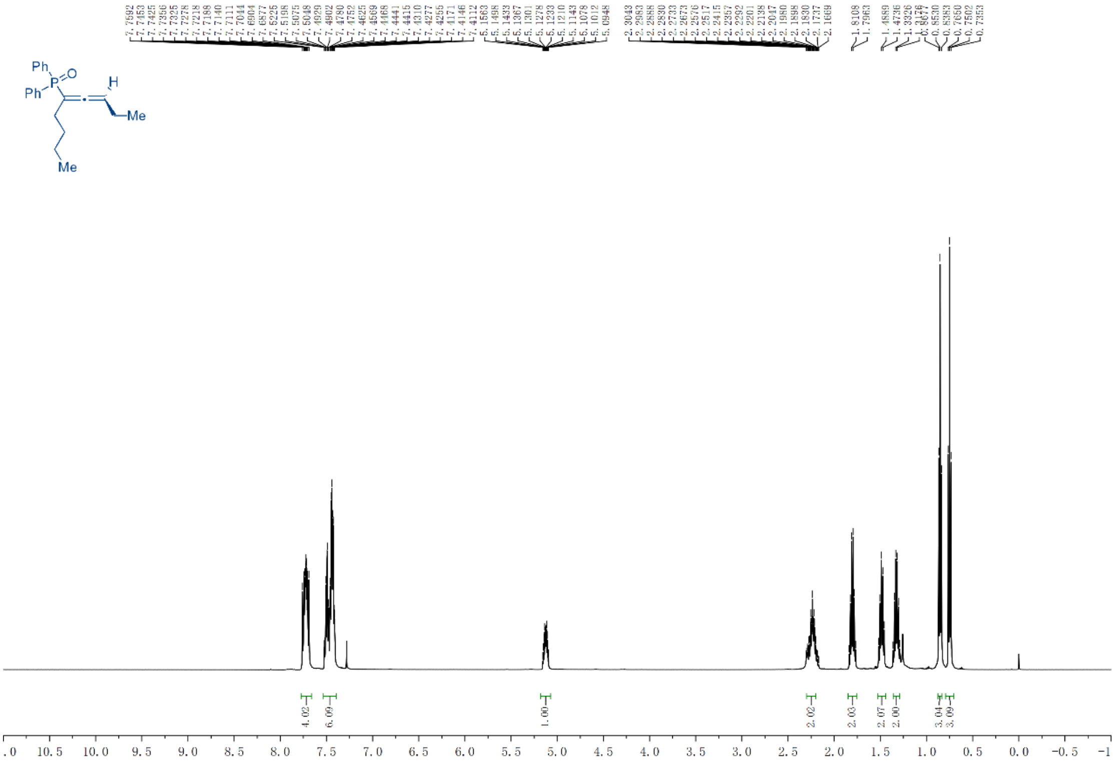

^{13}C NMR of 3s

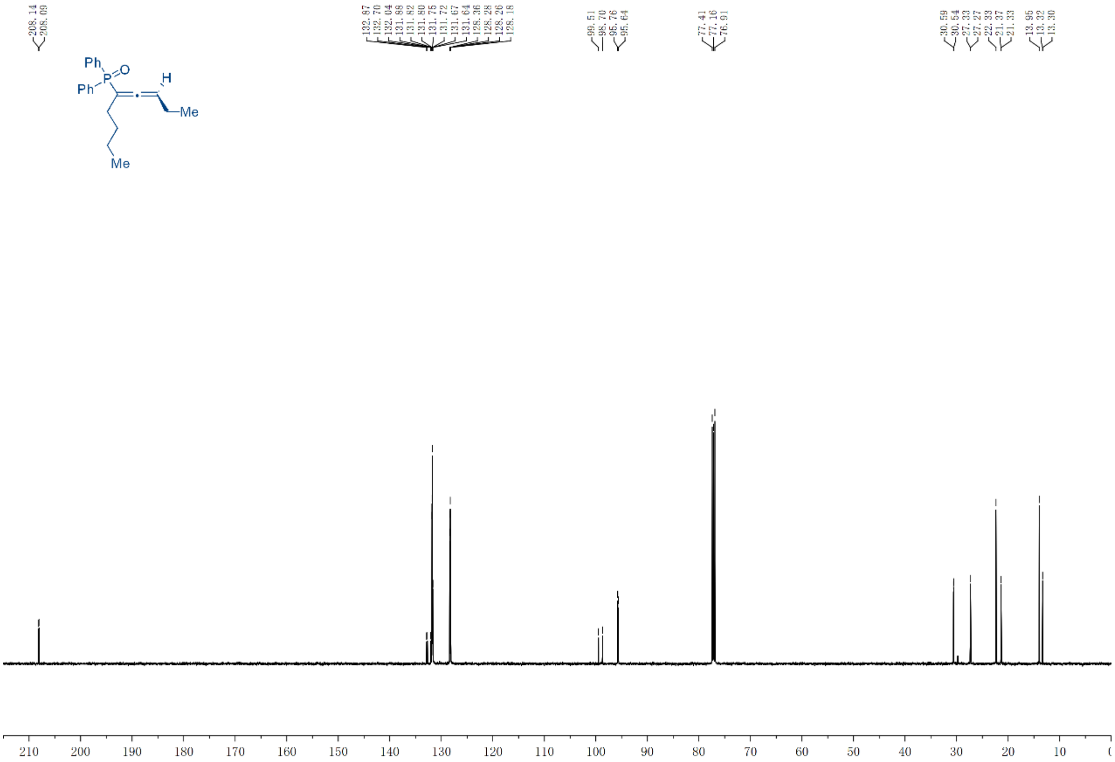

^{31}P NMR of 3s

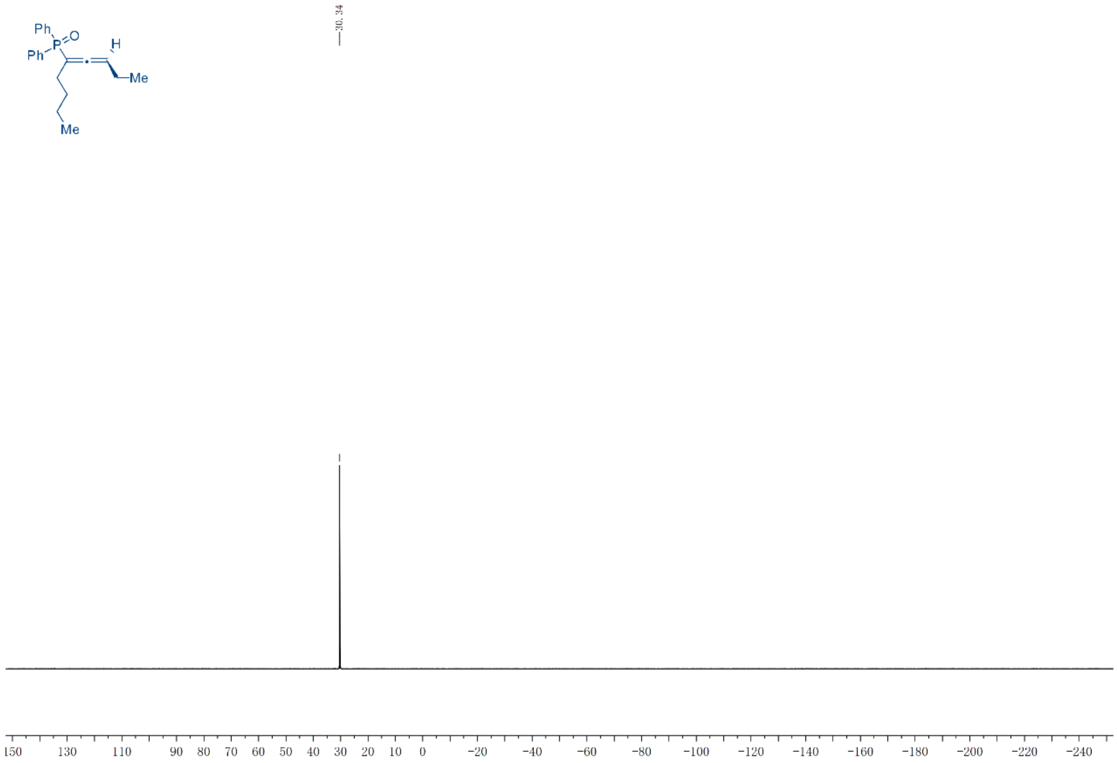

Supplementary Figure 30. ^{1}H NMR, ^{13}C NMR, and ^{31}P NMR spectra of compound **3s**

^{1}H NMR of 3t

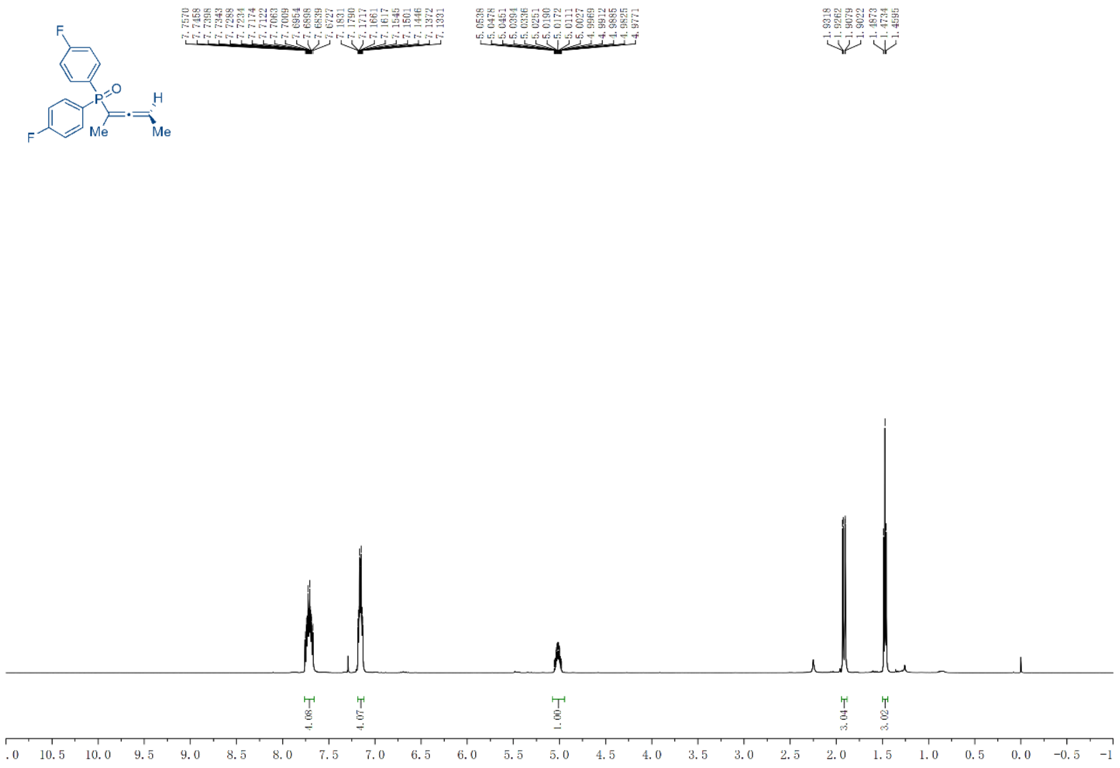

^{13}C NMR of 3t

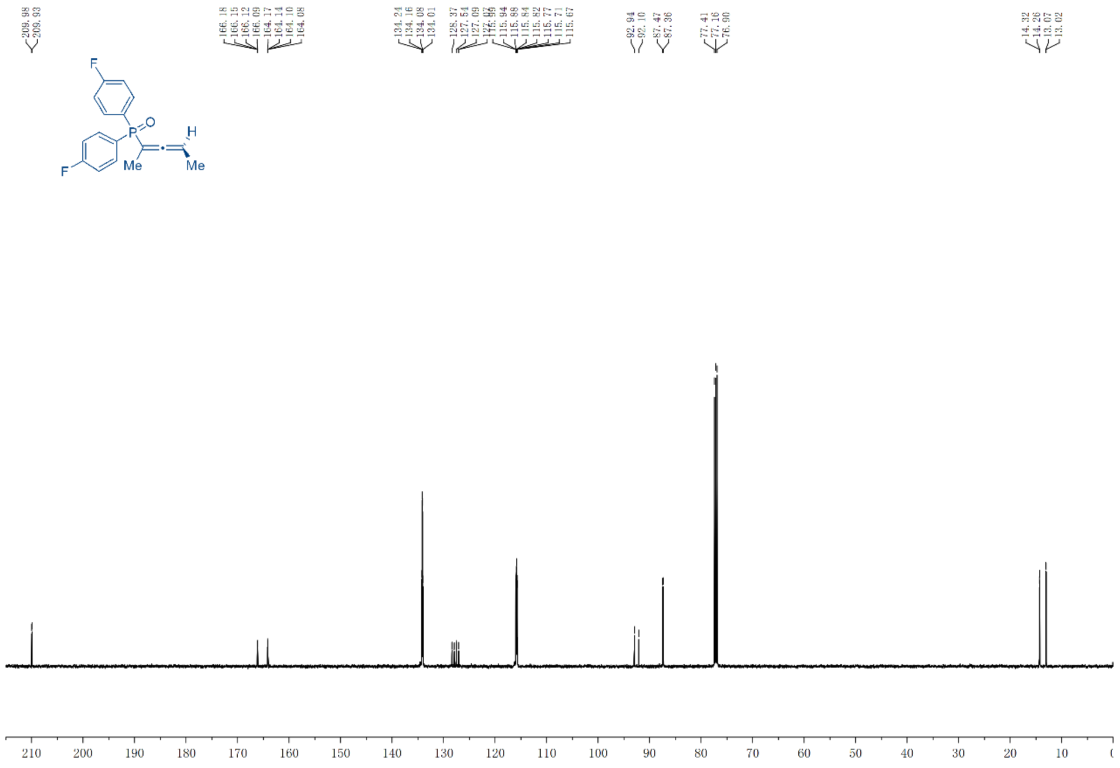

^{31}P NMR of 3t

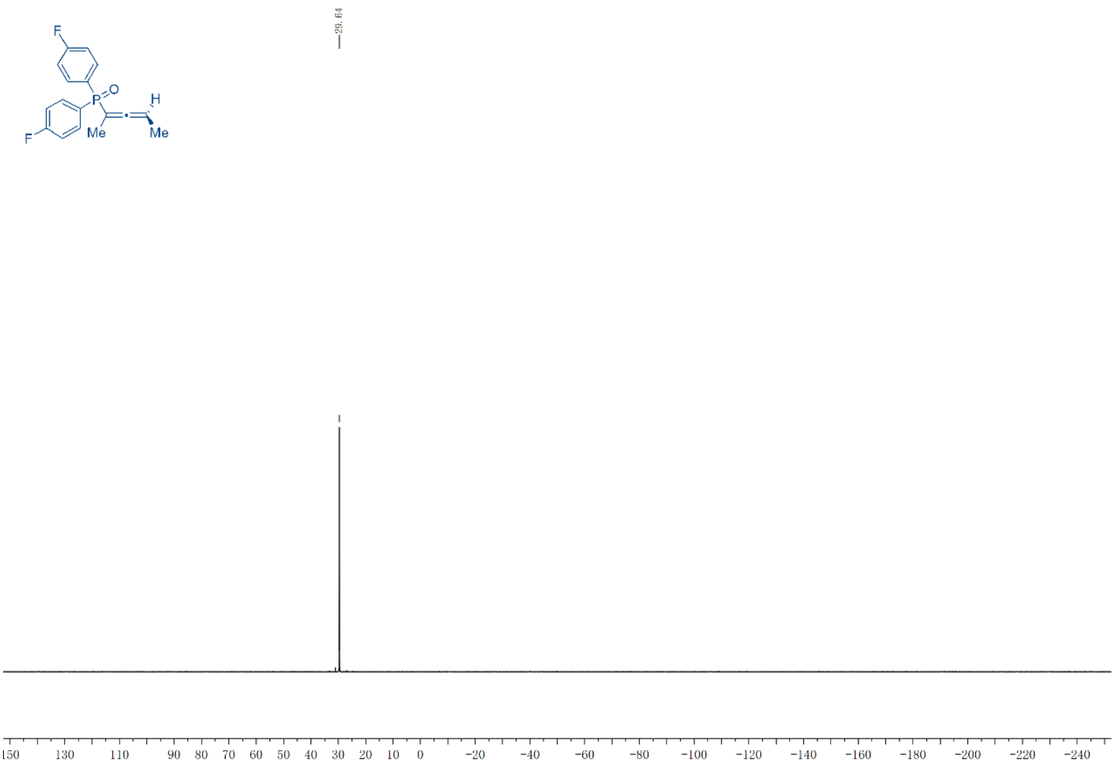

^{19}F NMR of 3t

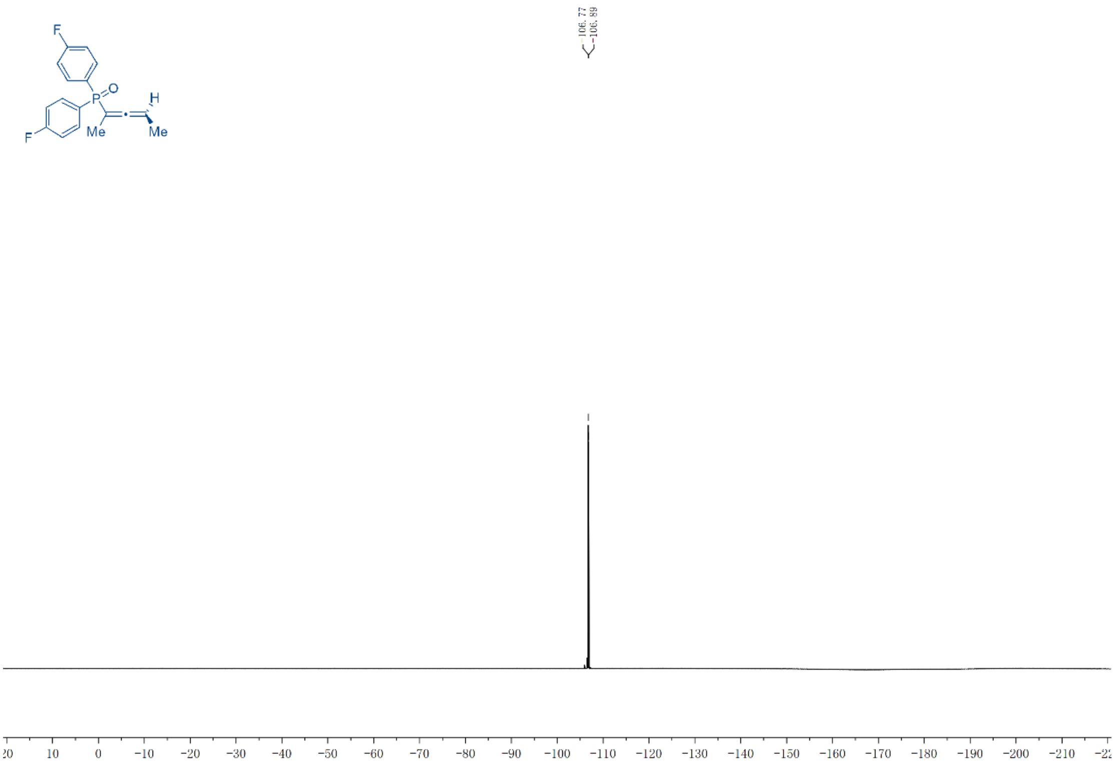

Supplementary Figure 31. ^{1}H NMR, ^{13}C NMR, ^{31}P NMR, and ^{19}F NMR spectra of compound **3t**

^{1}H NMR of 3u

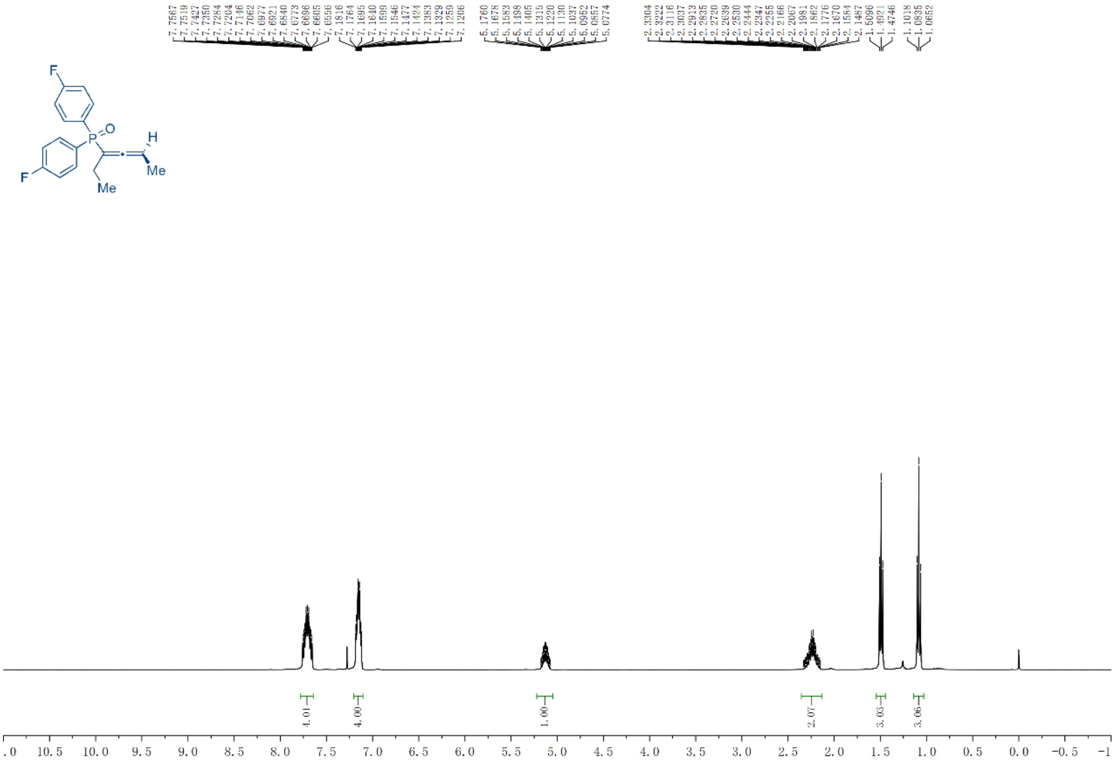

^{13}C NMR of 3u

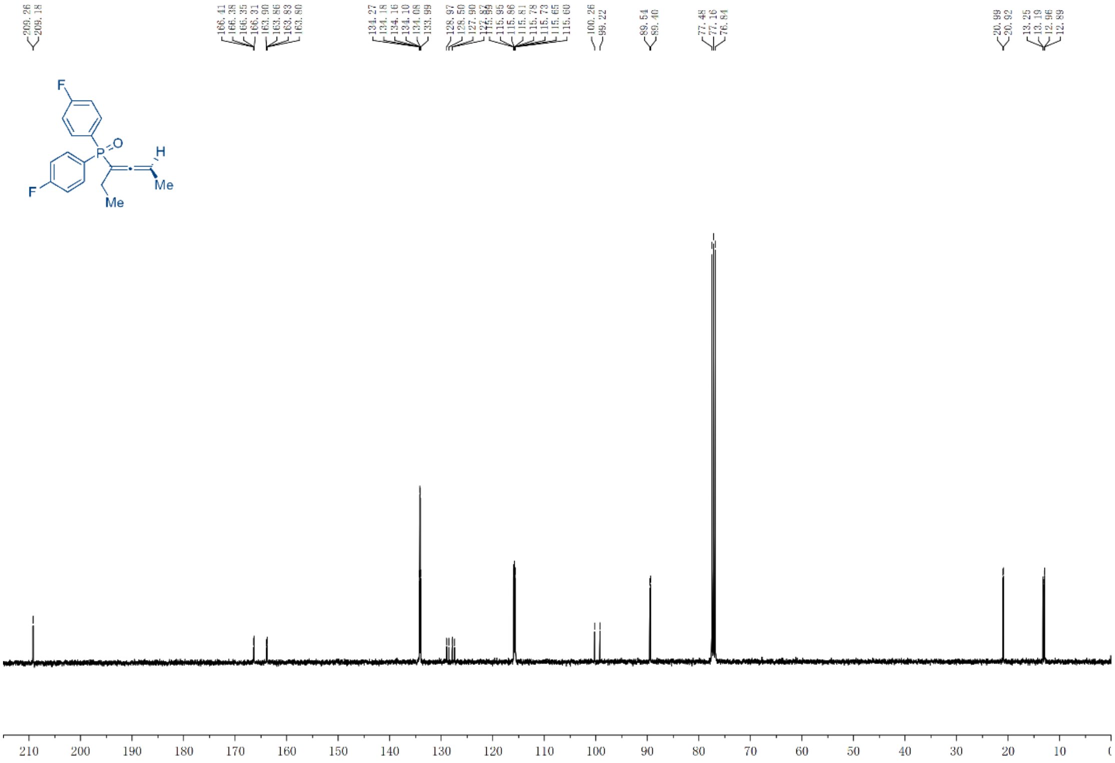

^{31}P NMR of 3u

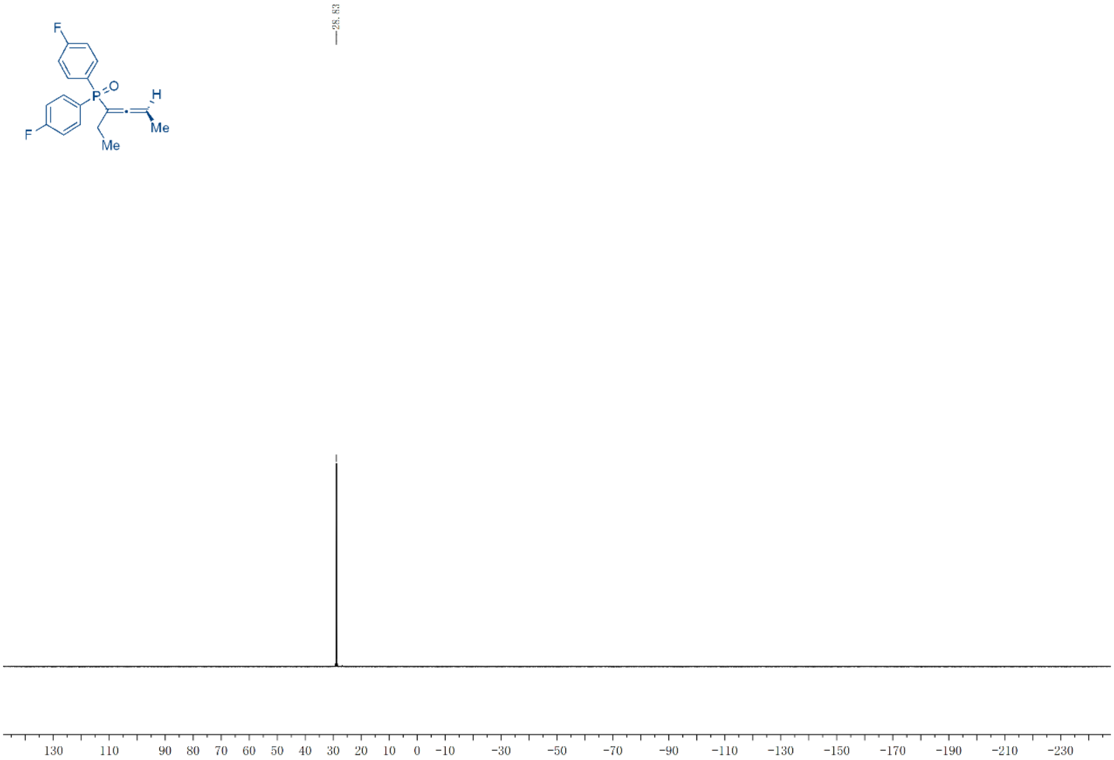

^{19}F NMR of 3u

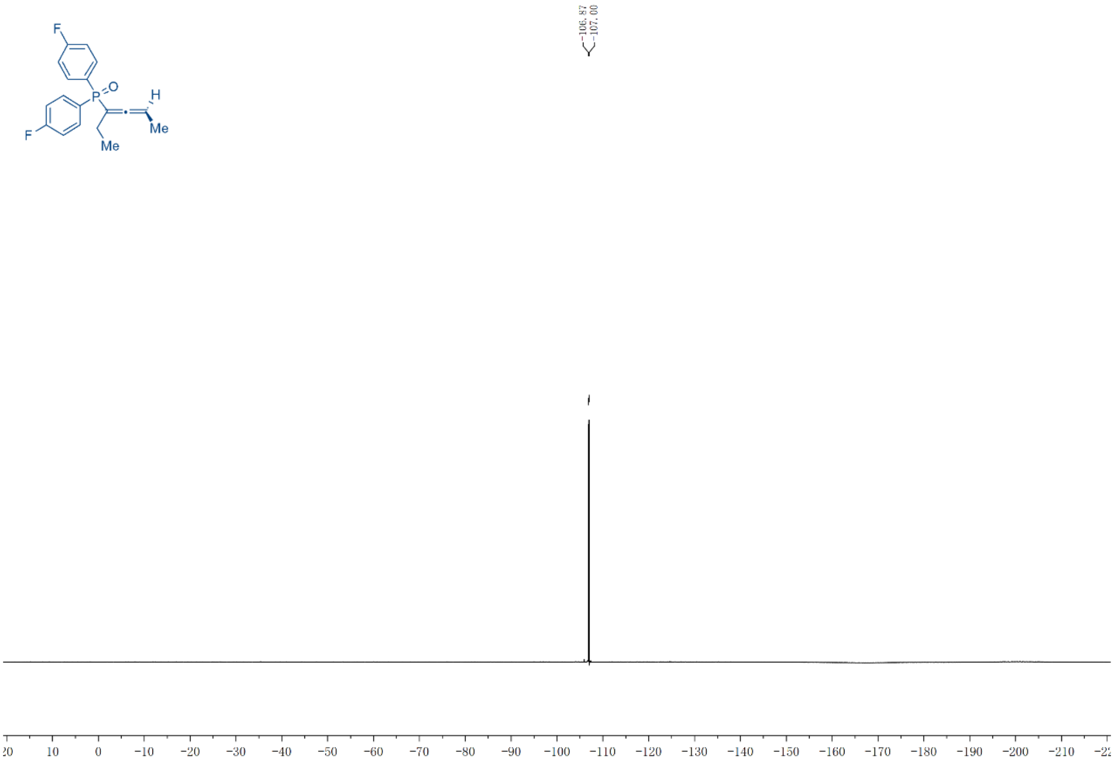

Supplementary Figure 32. ^{1}H NMR, ^{13}C NMR, ^{31}P NMR, and ^{19}F NMR spectra of compound **3u**

^{1}H NMR of 3v

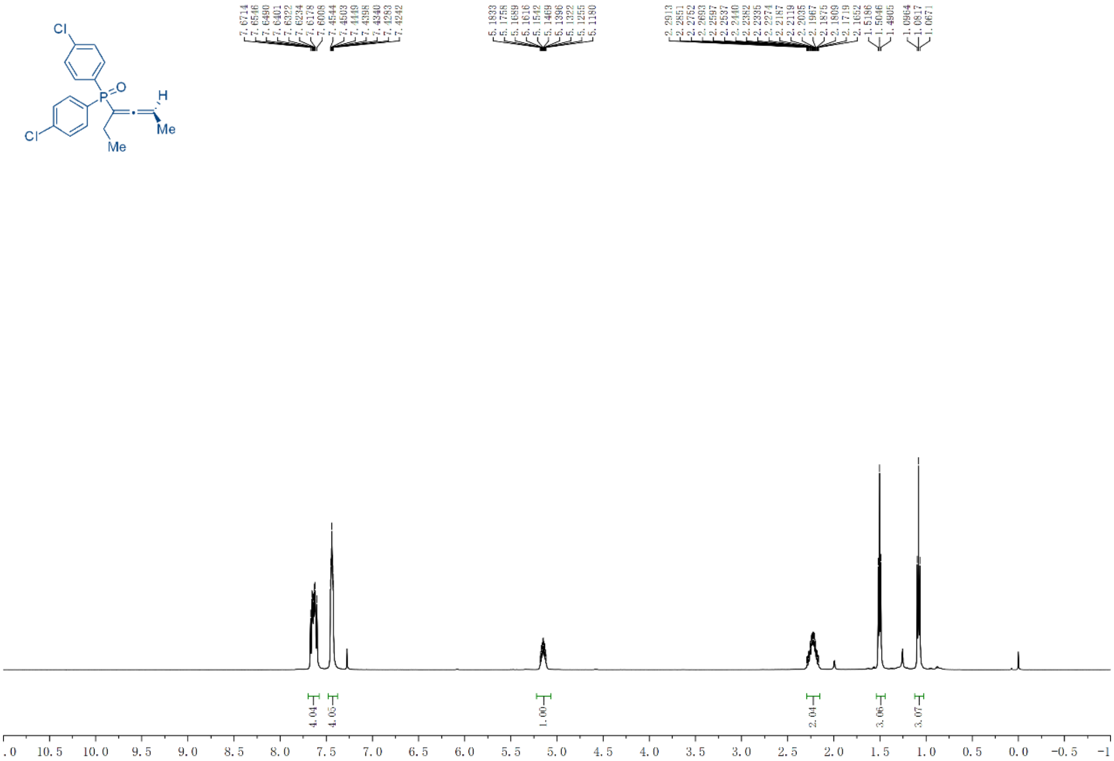

^{13}C NMR of 3v

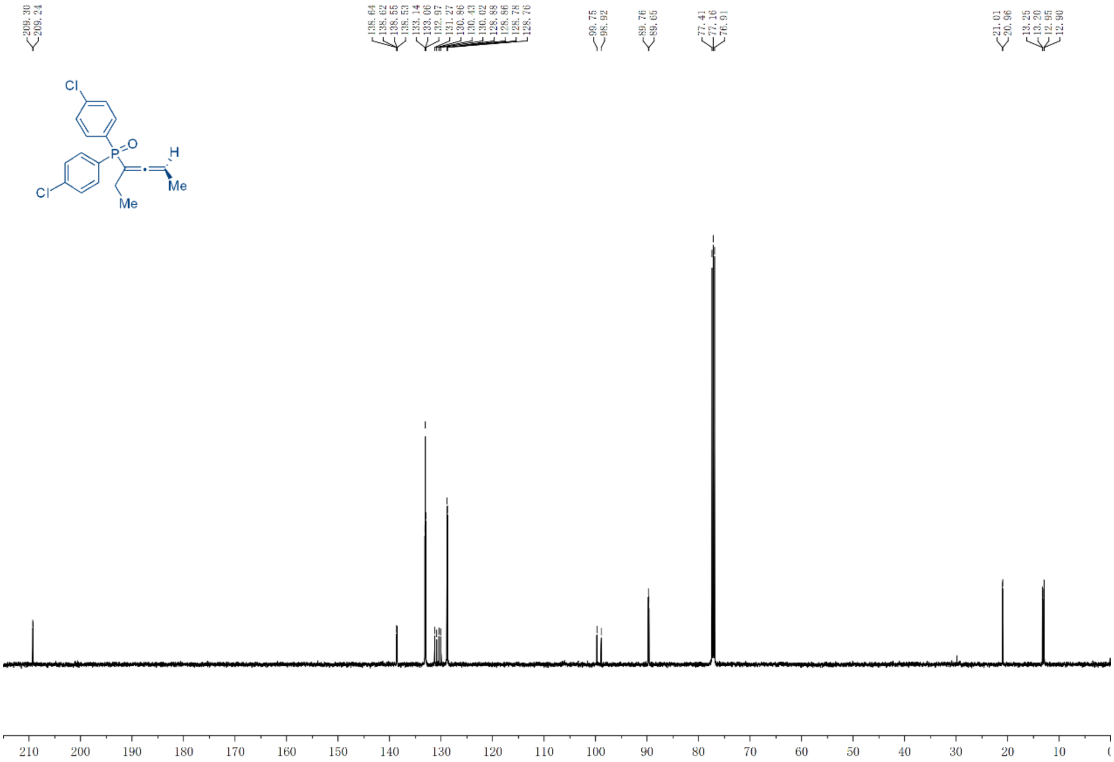

^{31}P NMR of 3v

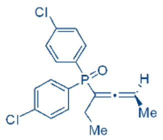

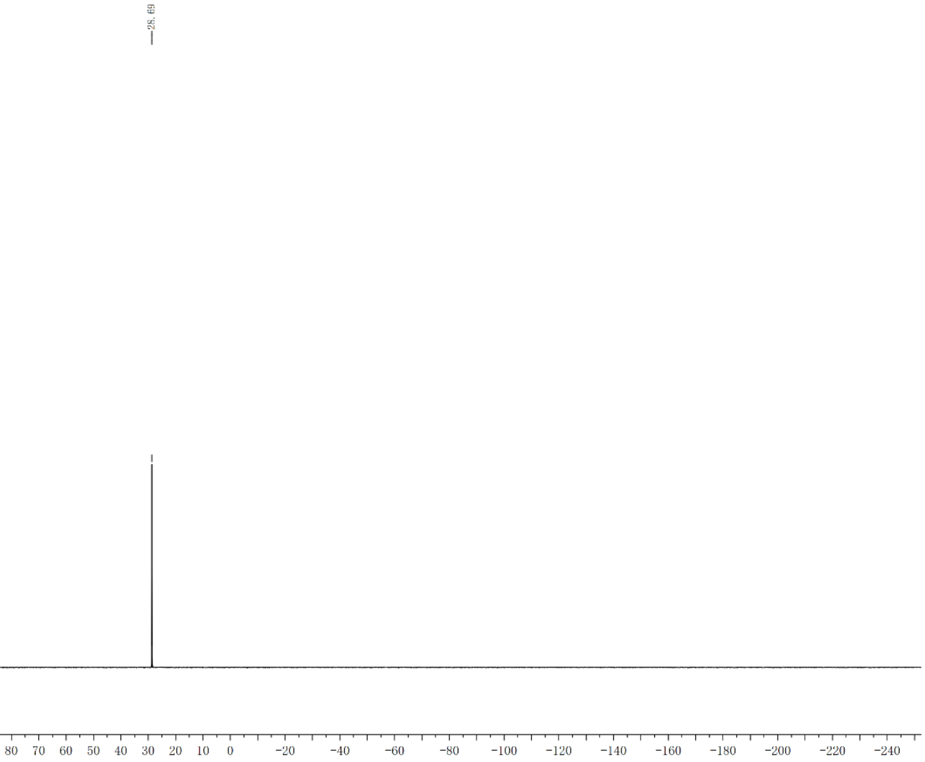

Supplementary Figure 33. ^{1}H NMR, ^{13}C NMR, and ^{31}P NMR spectra of compound **3v**

^{1}H NMR of 3w

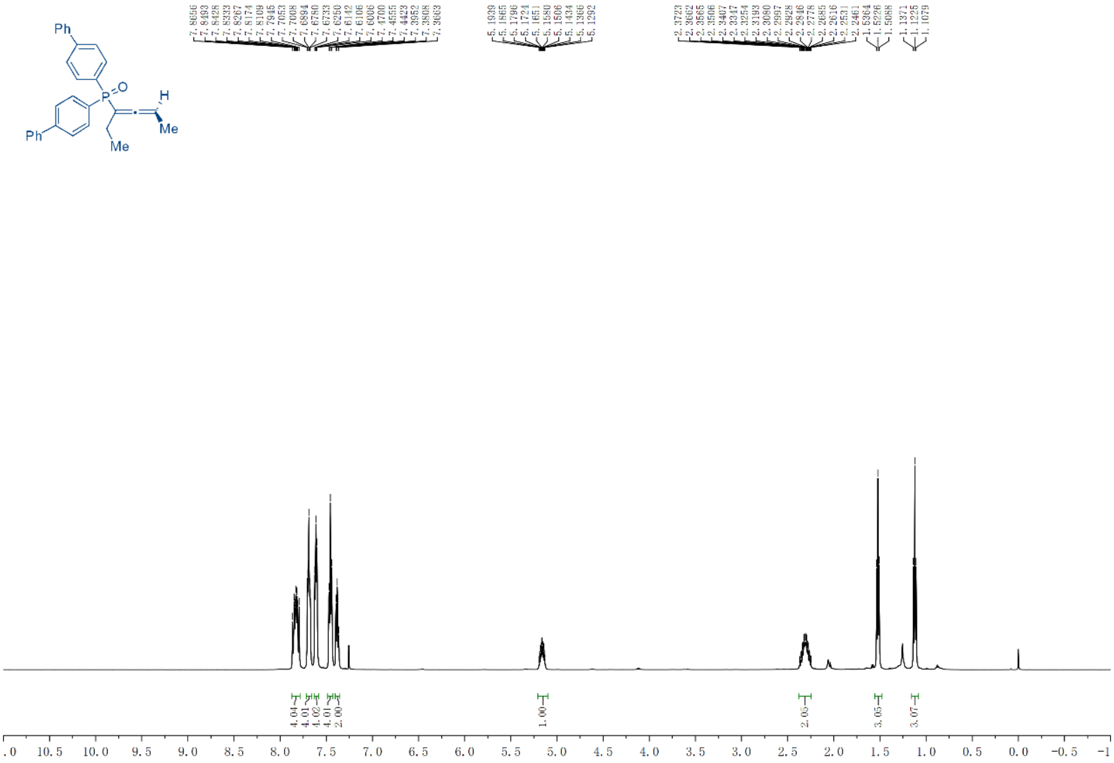

^{13}C NMR of 3w

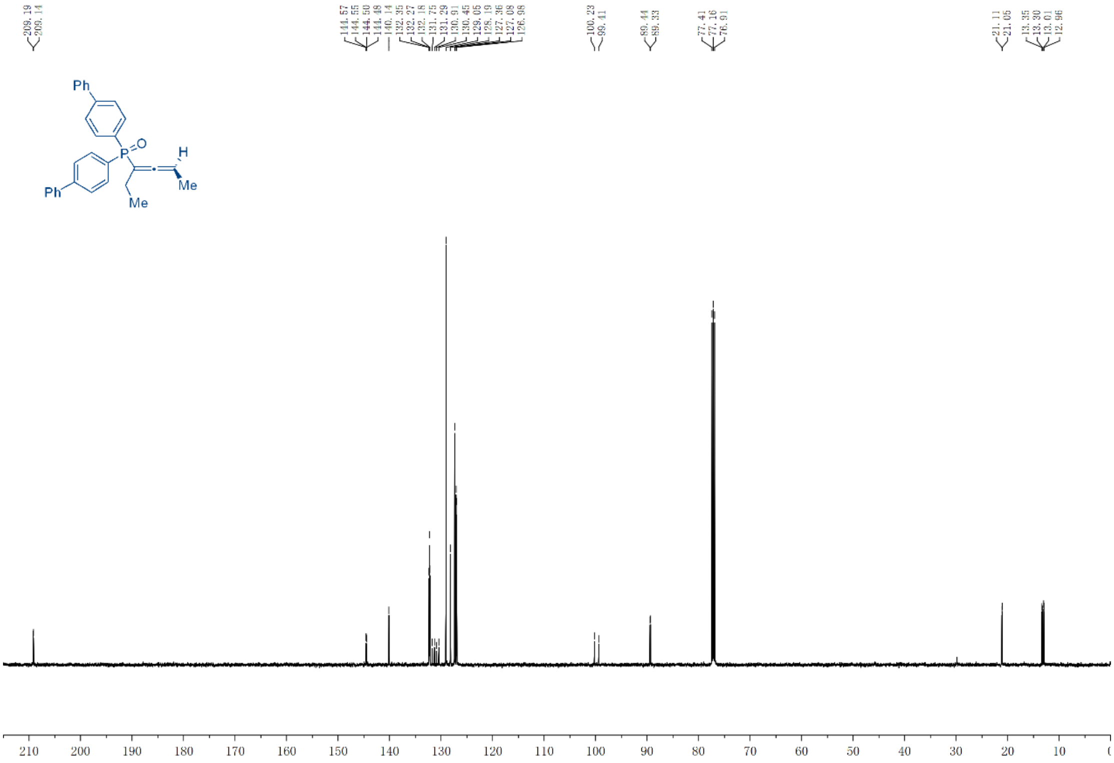

^{31}P NMR of 3w

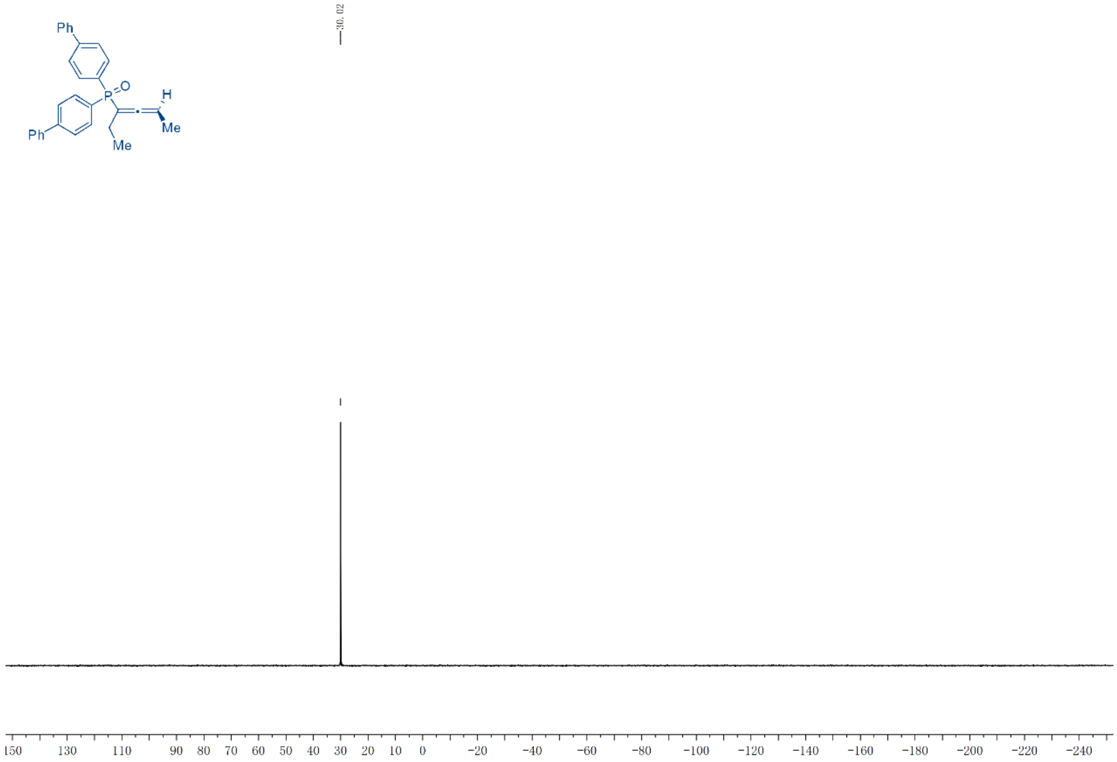

Supplementary Figure 34. ^{1}H NMR, ^{13}C NMR, and ^{31}P NMR spectra of compound **3w**

^{1}H NMR of 3x

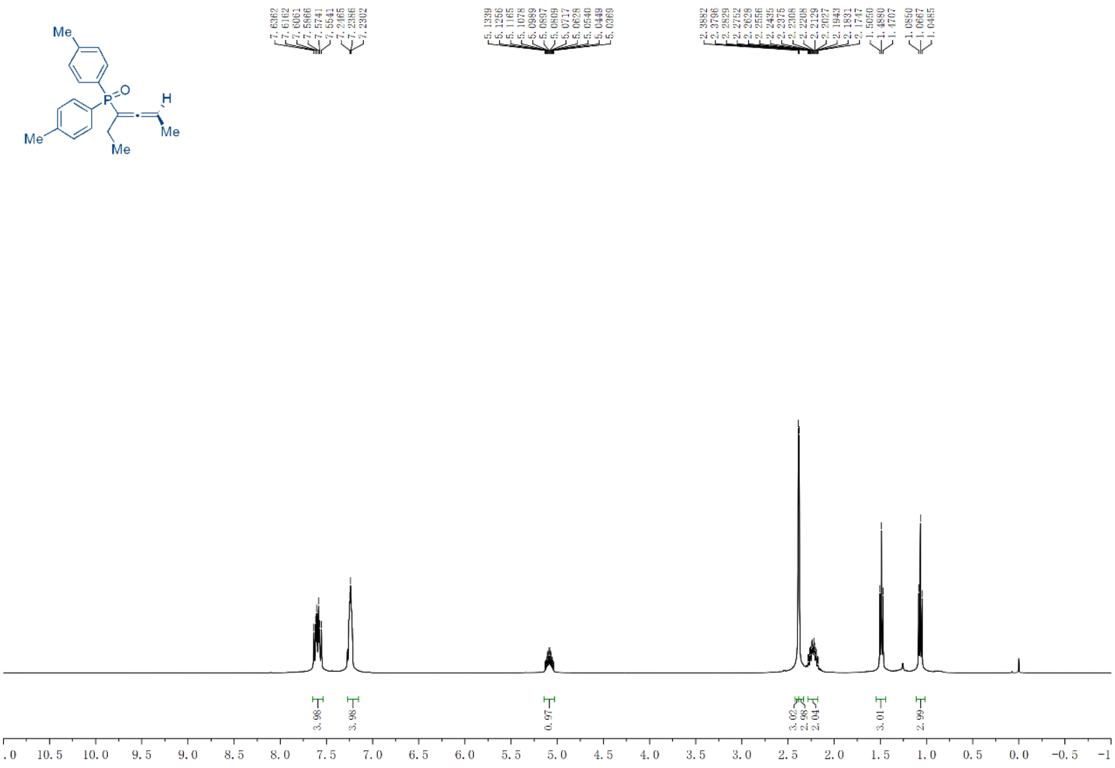

^{13}C NMR of 3x

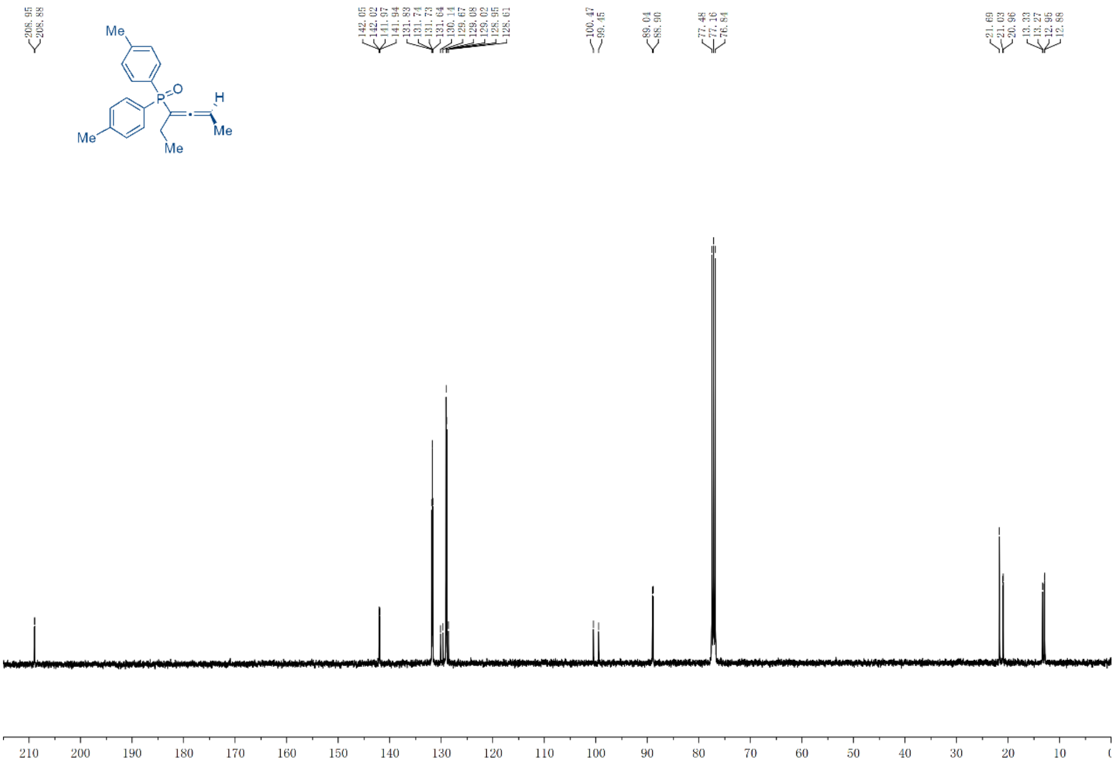

^{31}P NMR of 3x

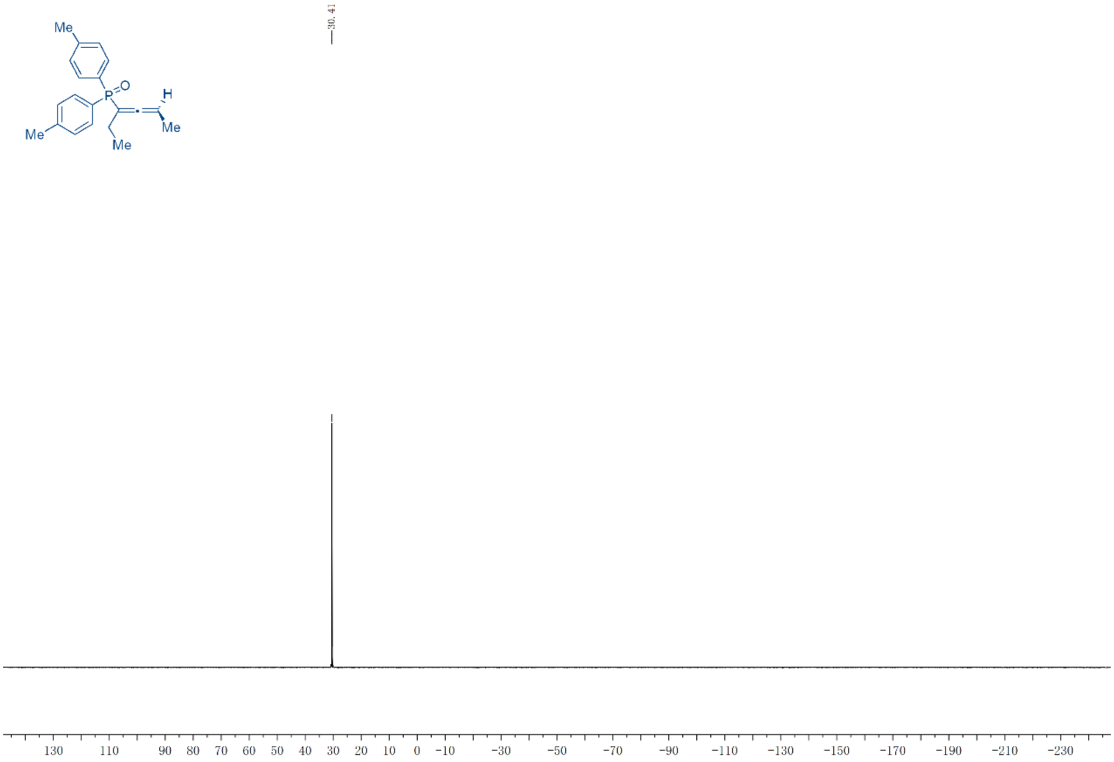

Supplementary Figure 35. ^{1}H NMR, ^{13}C NMR, and ^{31}P NMR spectra of compound **3x**

^{1}H NMR of 3y

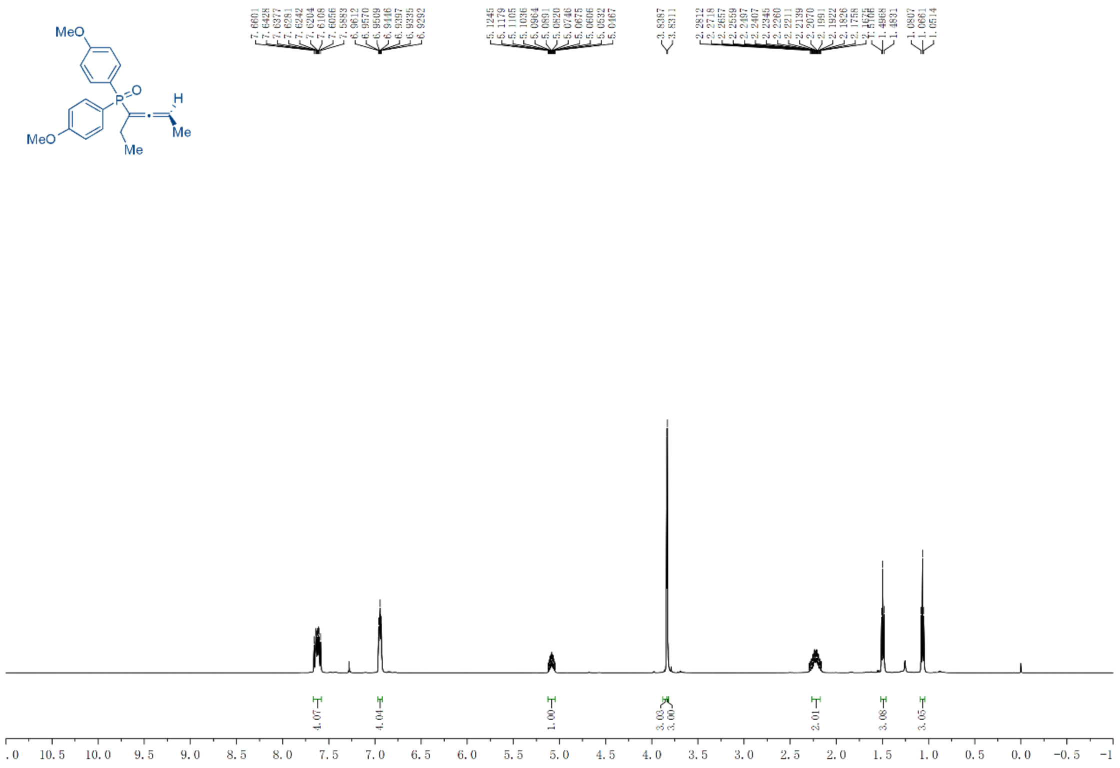

^{13}C NMR of 3y

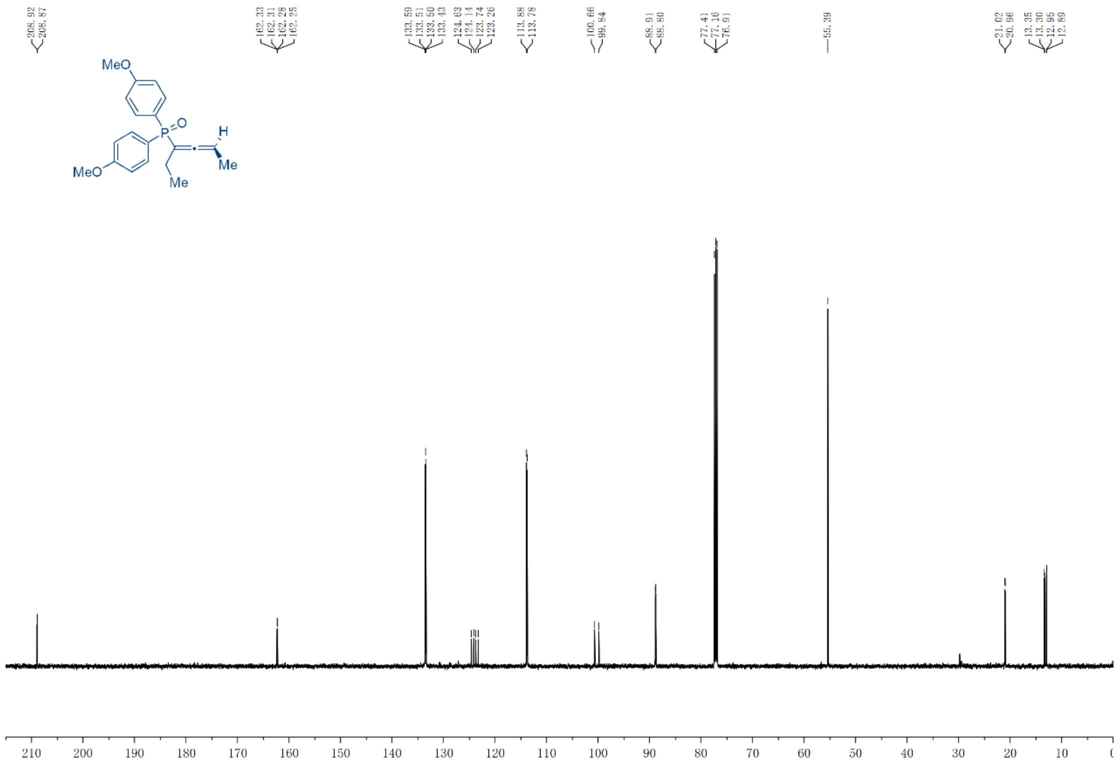

^{31}P NMR of 3y

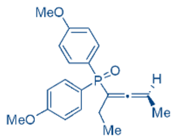

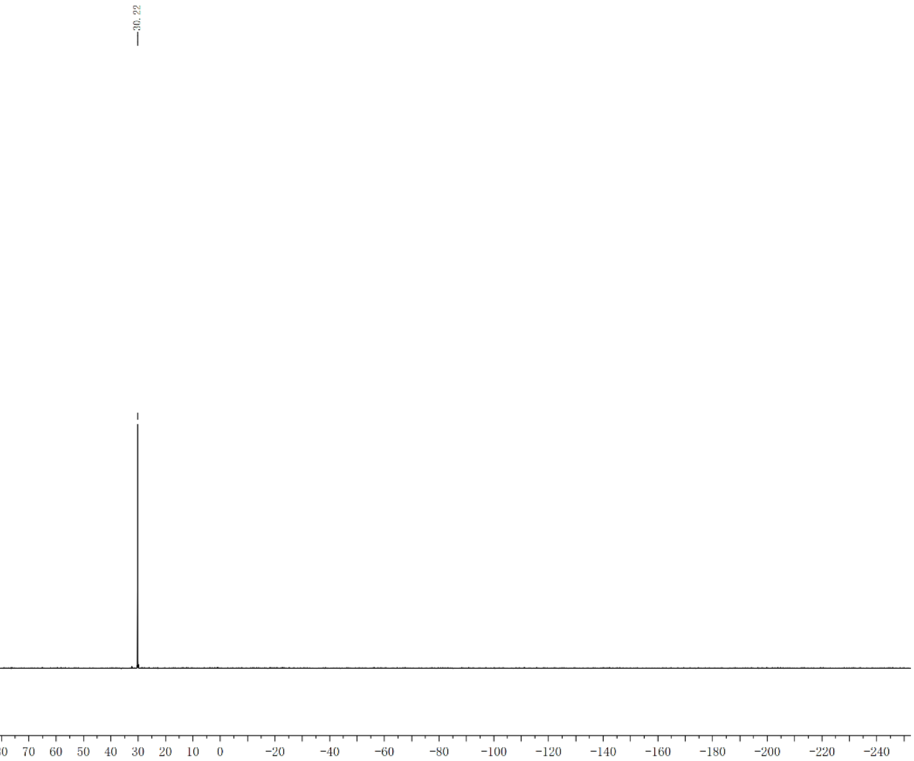

Supplementary Figure 36. ^{1}H NMR, ^{13}C NMR, and ^{31}P NMR spectra of compound **3y**

^{1}H NMR of 3z

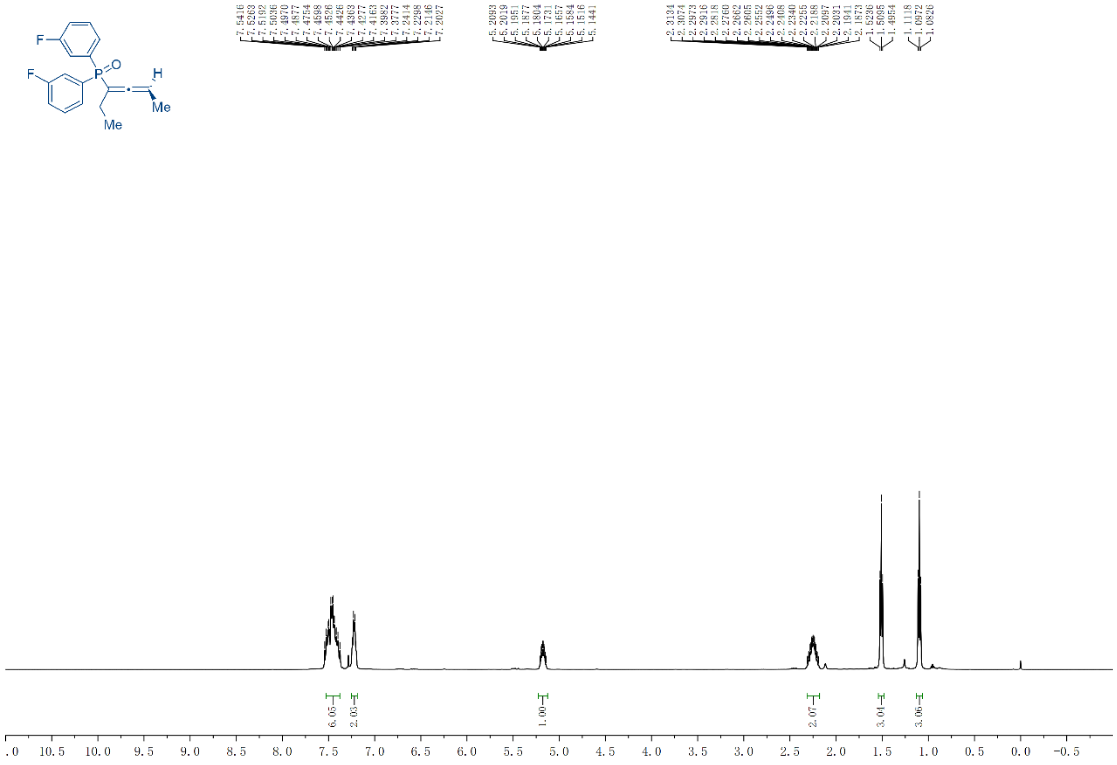

^{13}C NMR of 3z

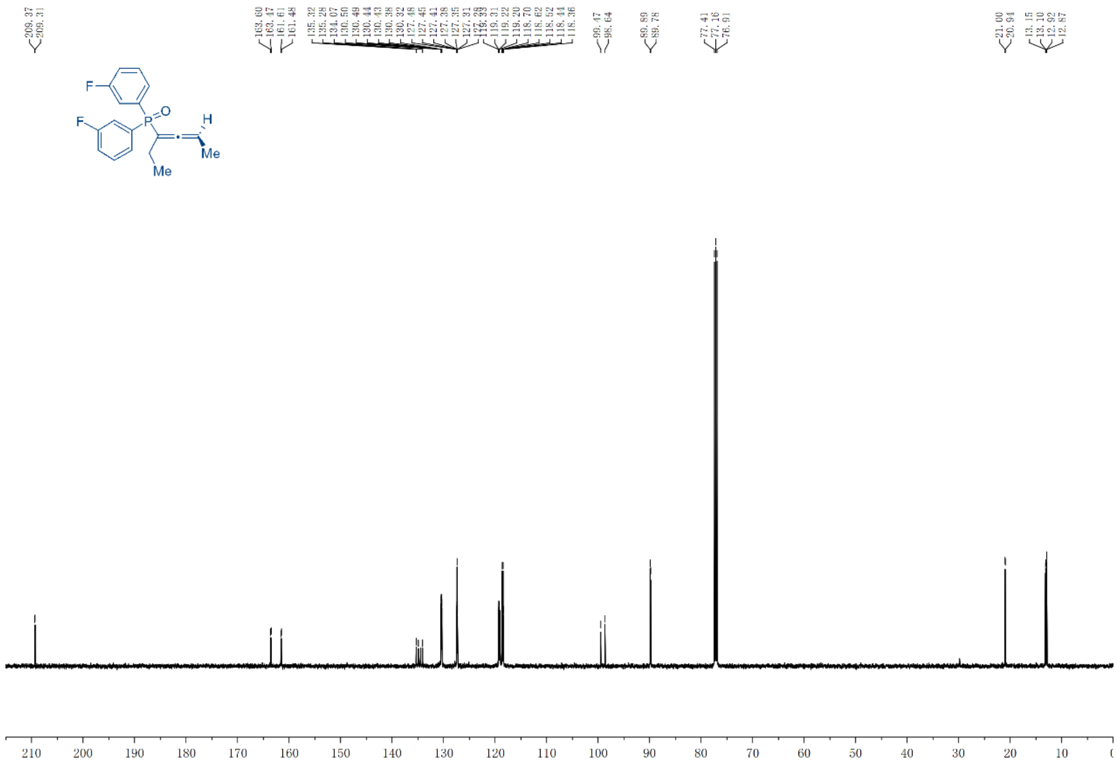

^{31}P NMR of 3z

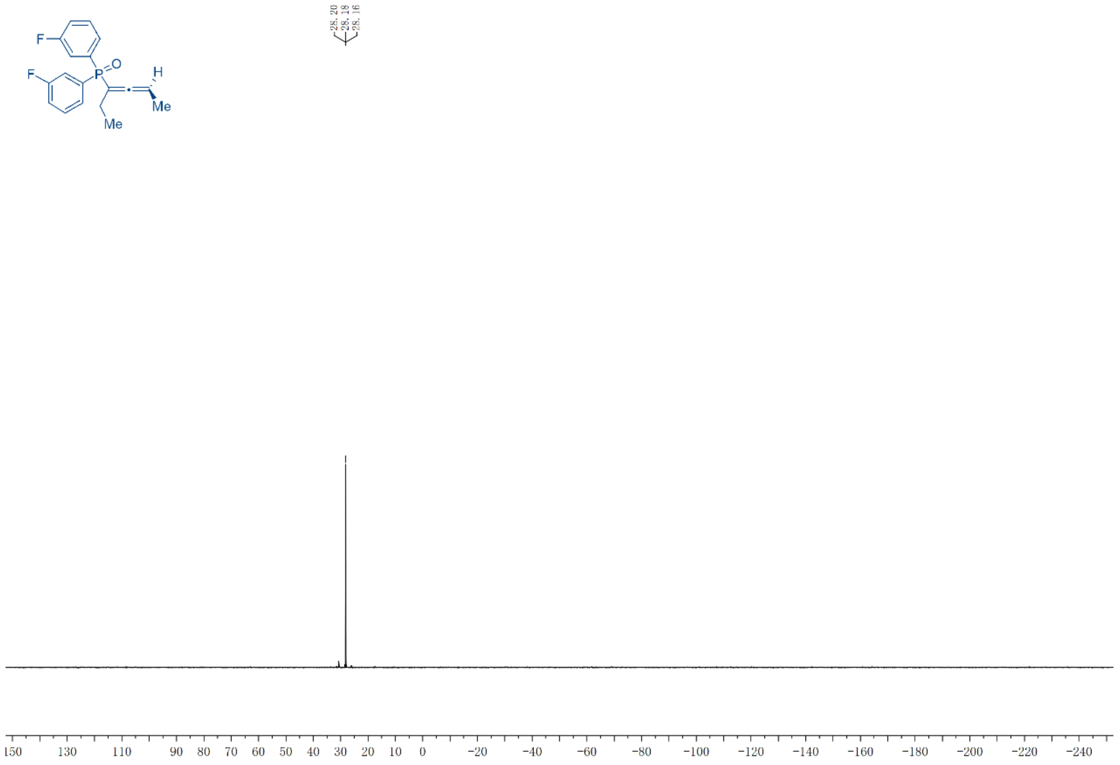

^{19}F NMR of 3z

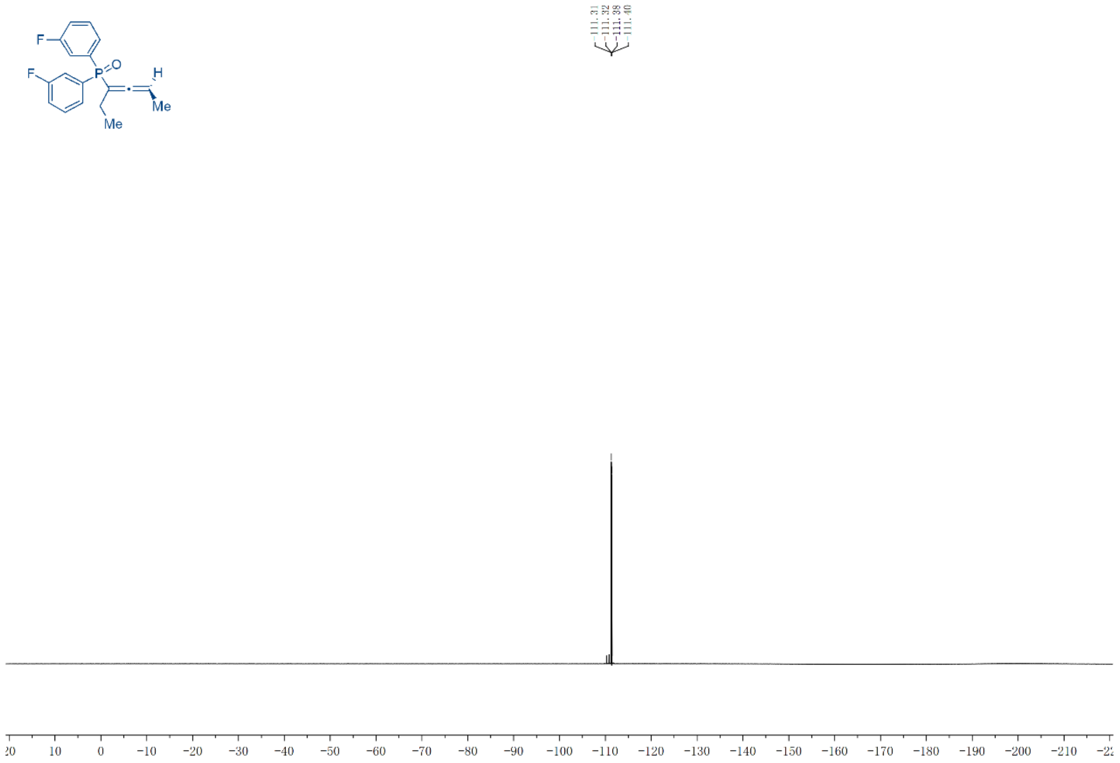

Supplementary Figure 37. ^{1}H NMR, ^{13}C NMR, ^{31}P NMR, and ^{19}F NMR spectra of compound **3z**

^{1}H NMR of 3aa

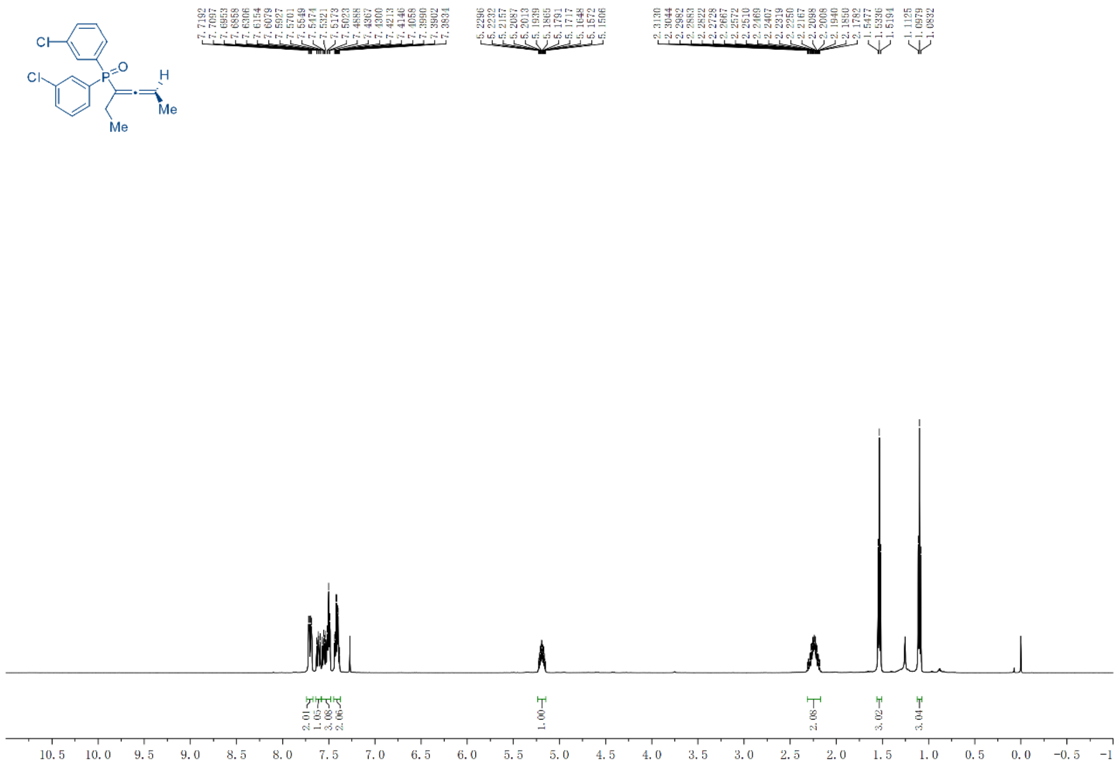

^{13}C NMR of 3aa

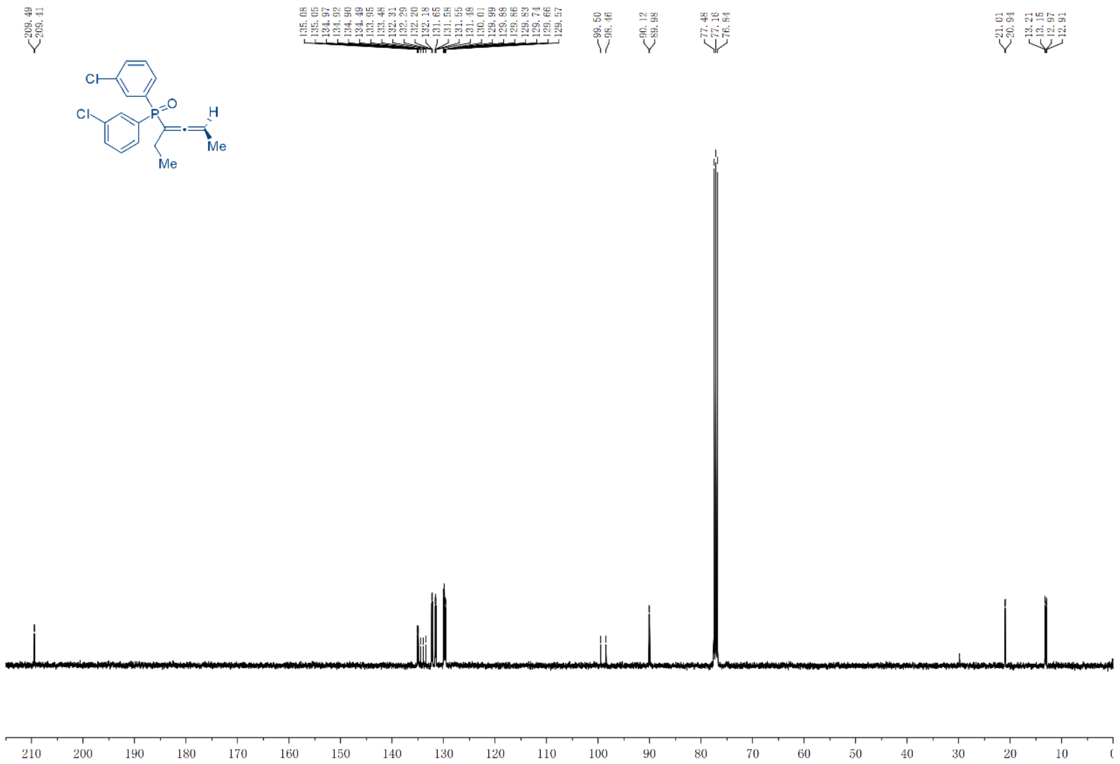

^{31}P NMR of 3aa

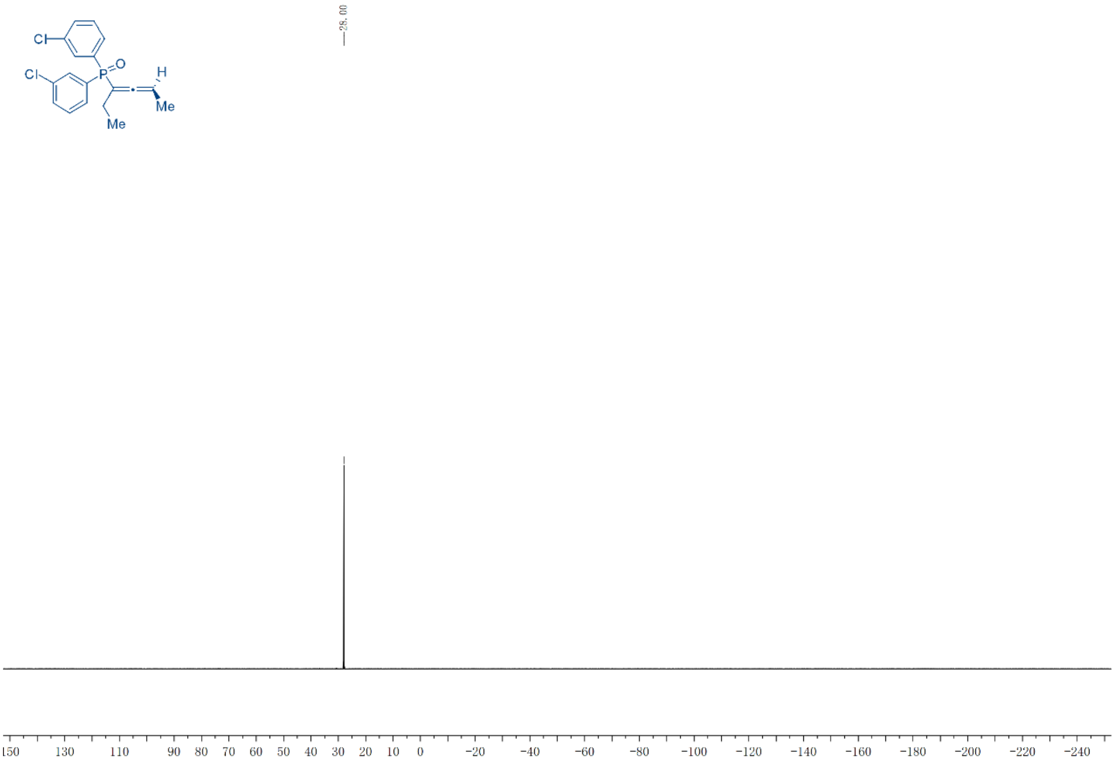

Supplementary Figure 38. ^{1}H NMR, ^{13}C NMR, and ^{31}P NMR spectra of compound **3aa**

^{1}H NMR of 3ab

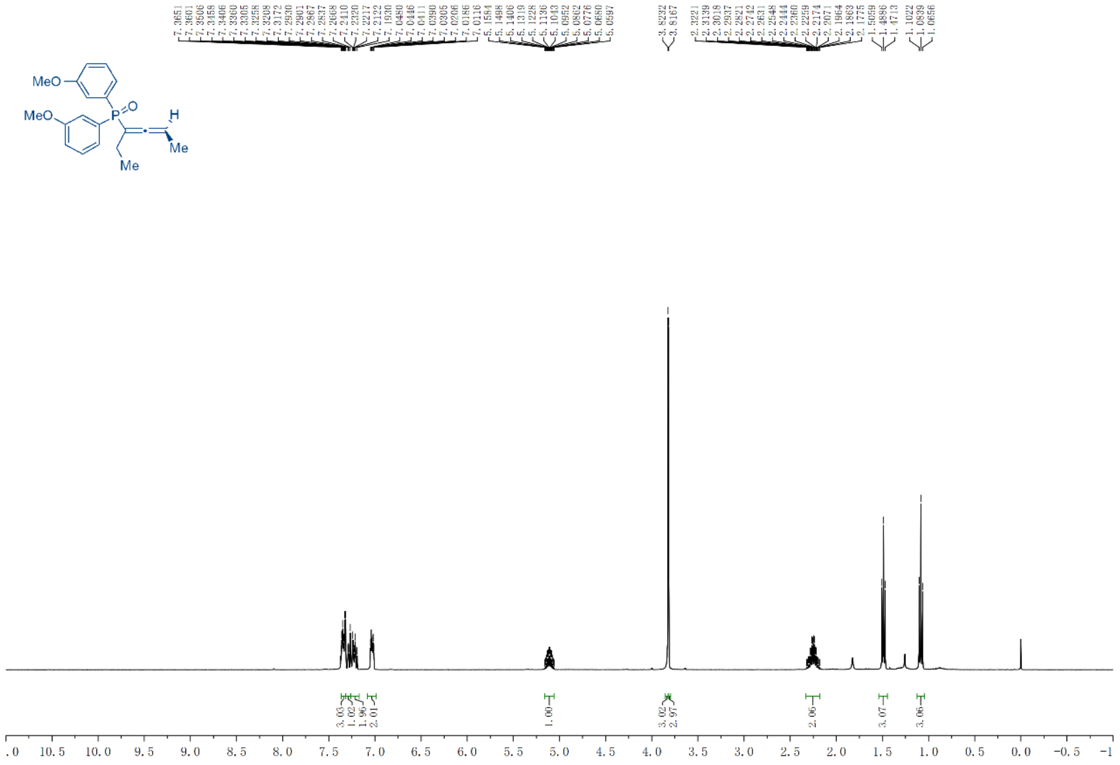

^{13}C NMR of 3ab

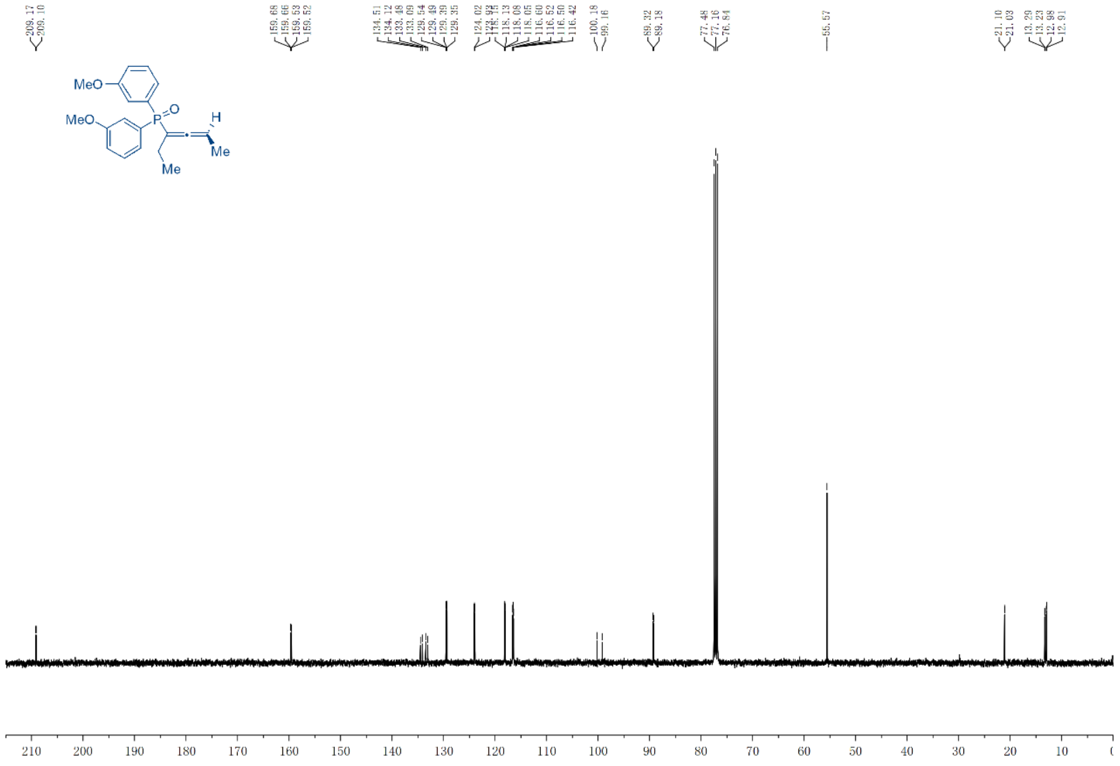

^{31}P NMR of 3ab

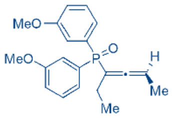

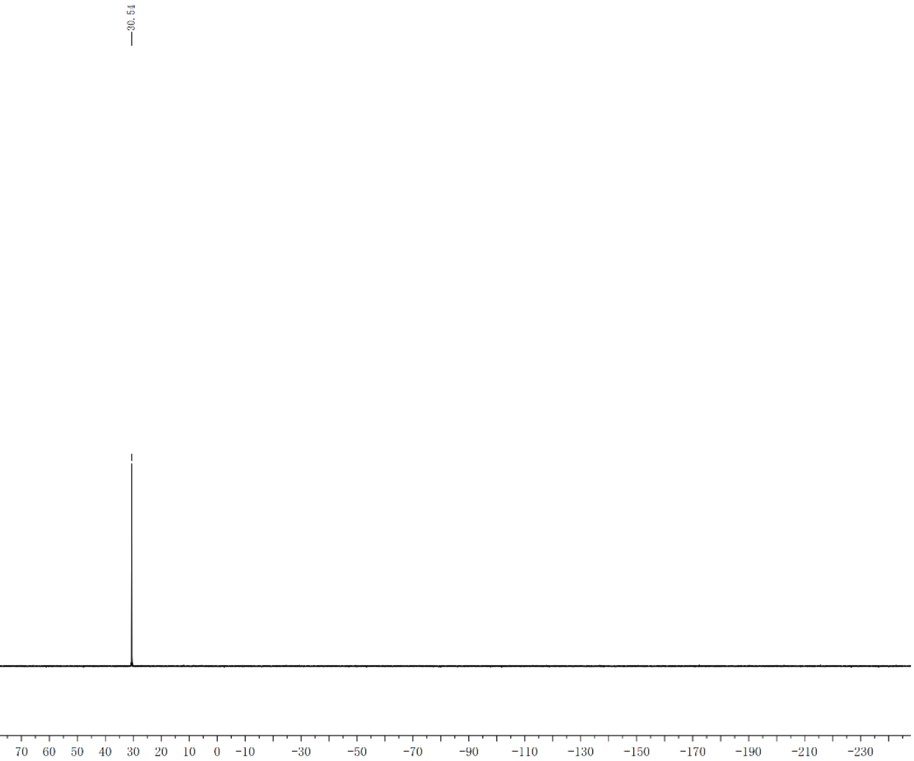

Supplementary Figure 39. ^{1}H NMR, ^{13}C NMR, and ^{31}P NMR spectra of compound **3ab**

^{1}H NMR of 3ac

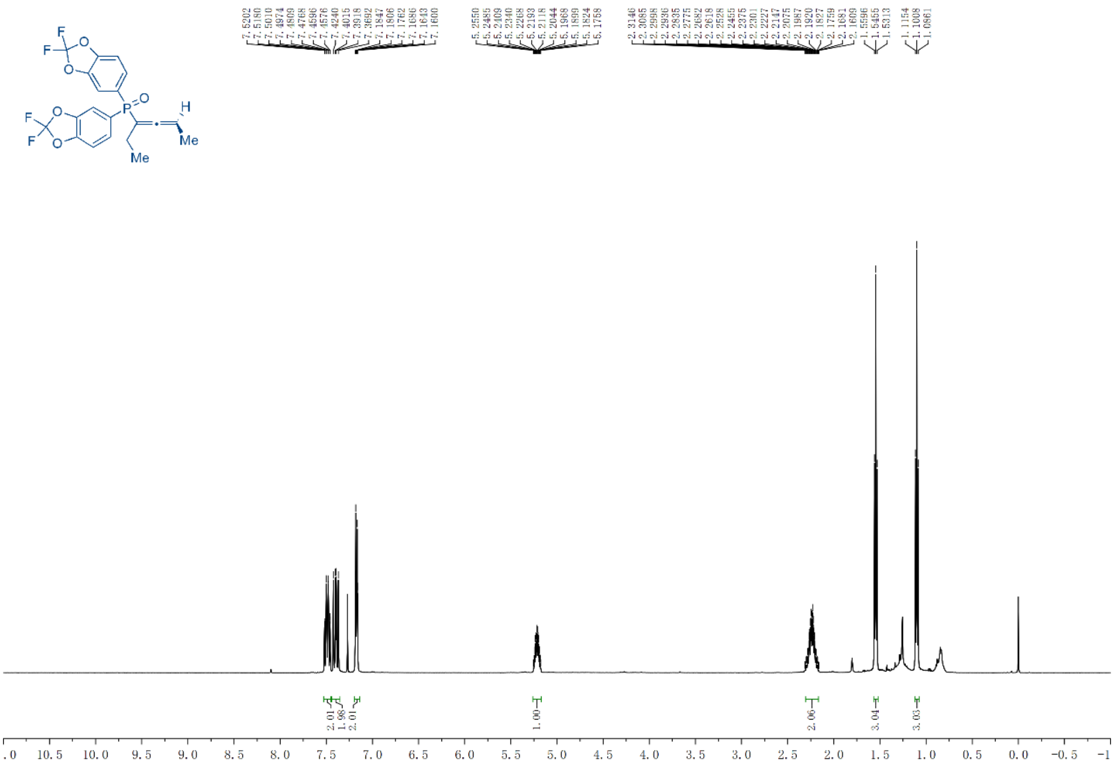

^{13}C NMR of 3ac

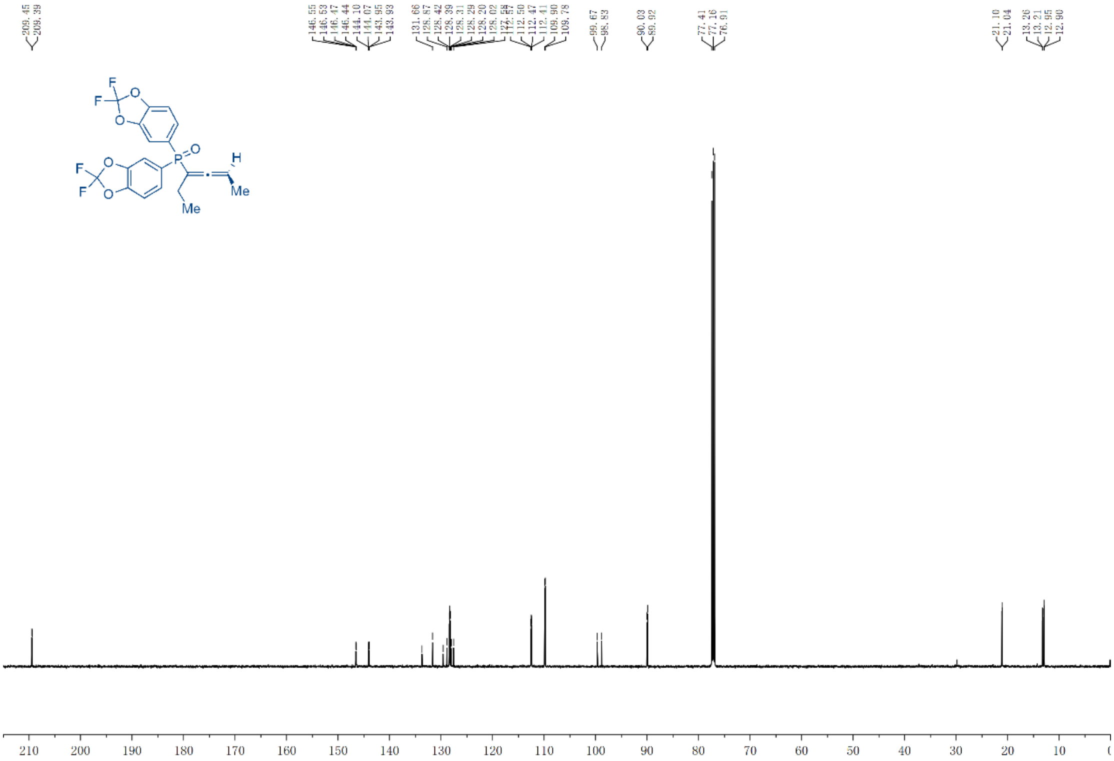

^{31}P NMR of 3ac

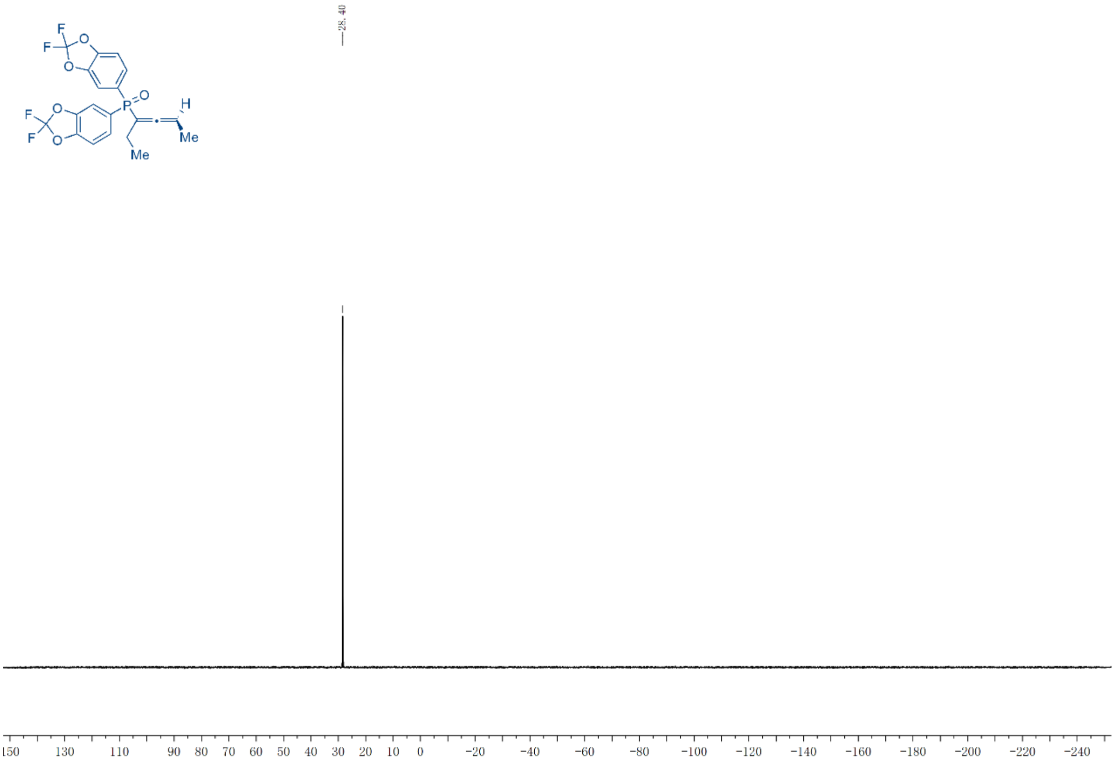

^{19}F NMR of 3ac

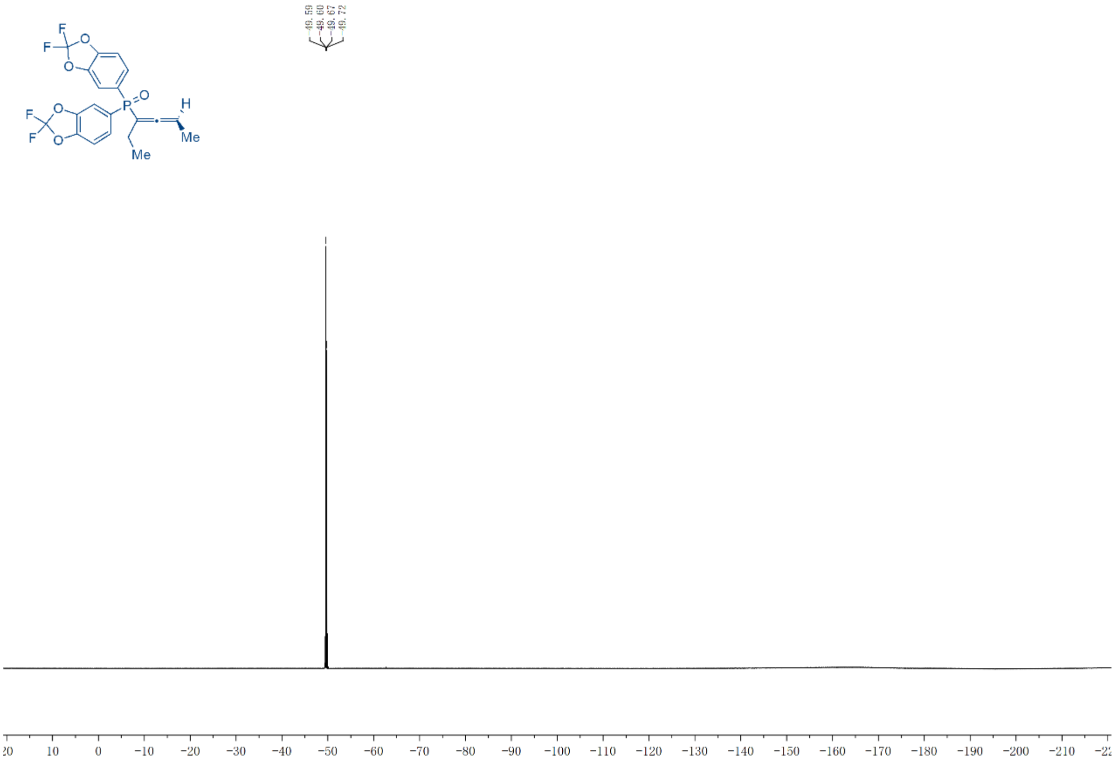

Supplementary Figure 40. ^{1}H NMR, ^{13}C NMR, ^{31}P NMR, and ^{19}F NMR spectra of compound **3ac**

^{1}H NMR of 4a

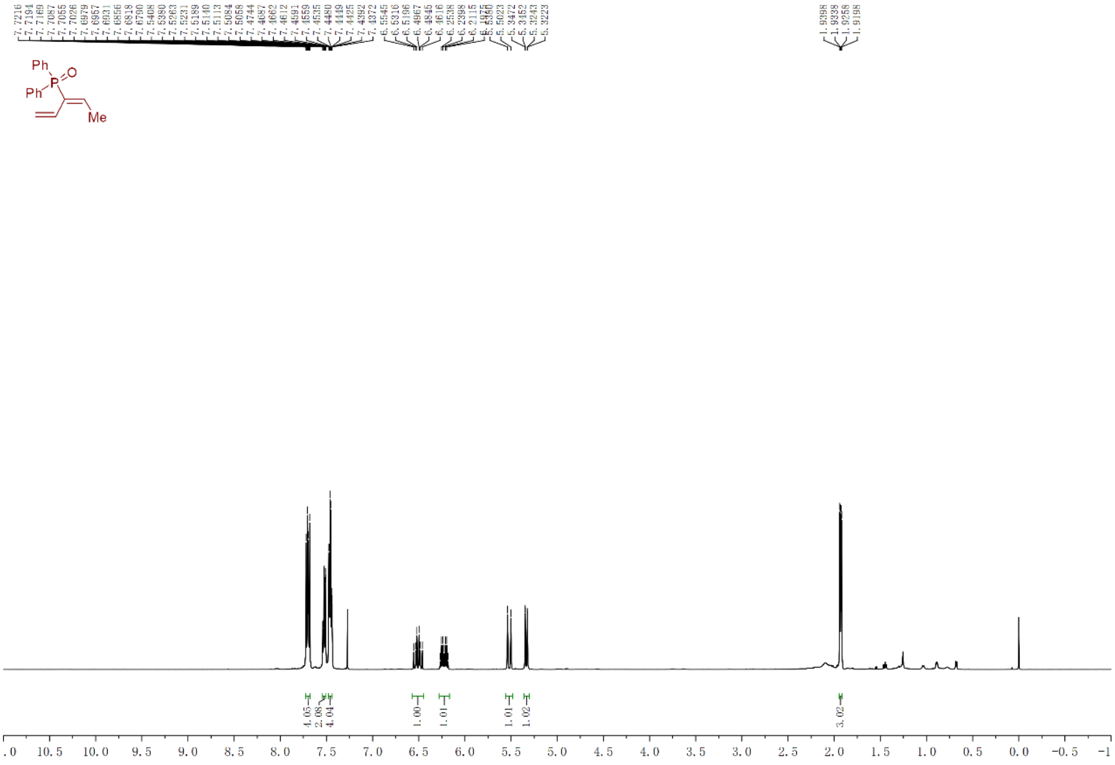

^{13}C NMR of 4a

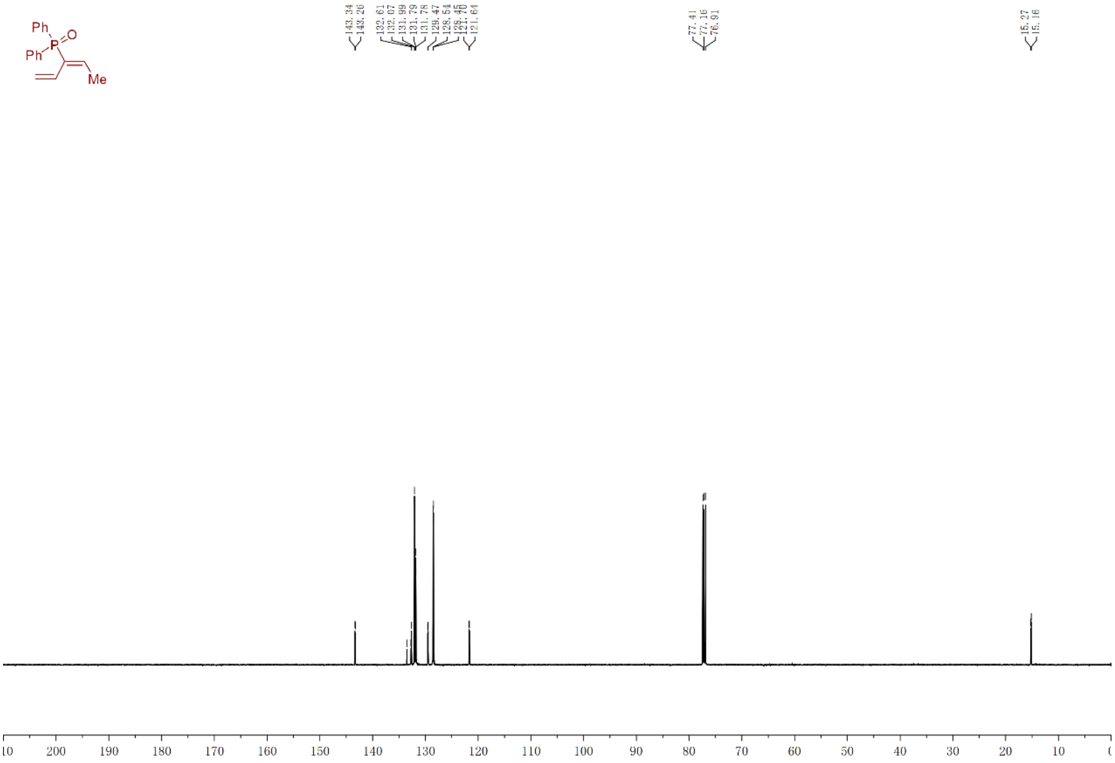

^{31}P NMR of 4a

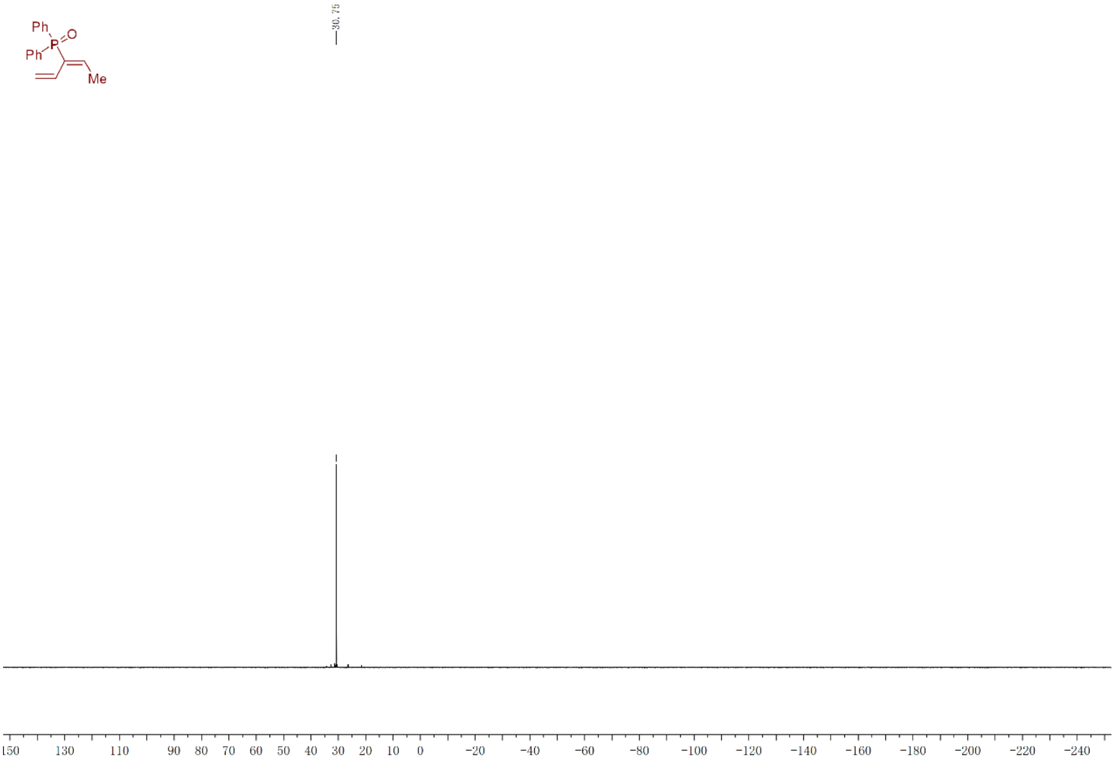

Supplementary Figure 41. ^{1}H NMR, ^{13}C NMR, and ^{31}P NMR spectra of compound **4a**

^{1}H NMR of 4b

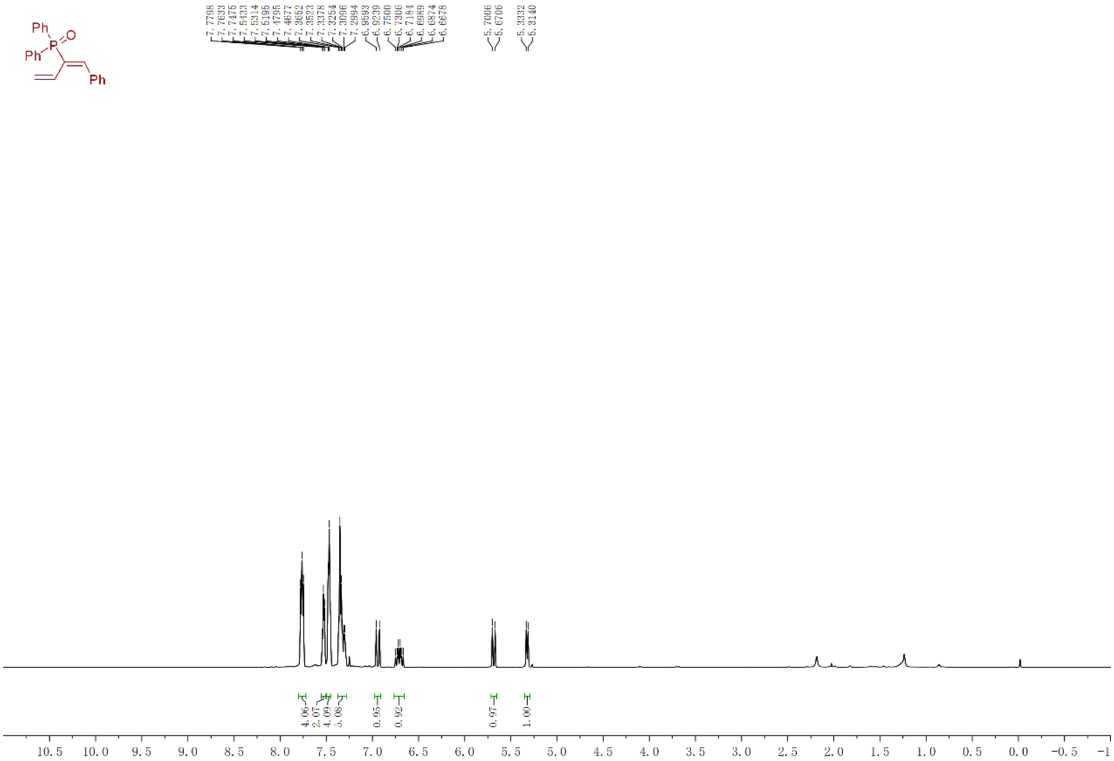

^{13}C NMR of 4b

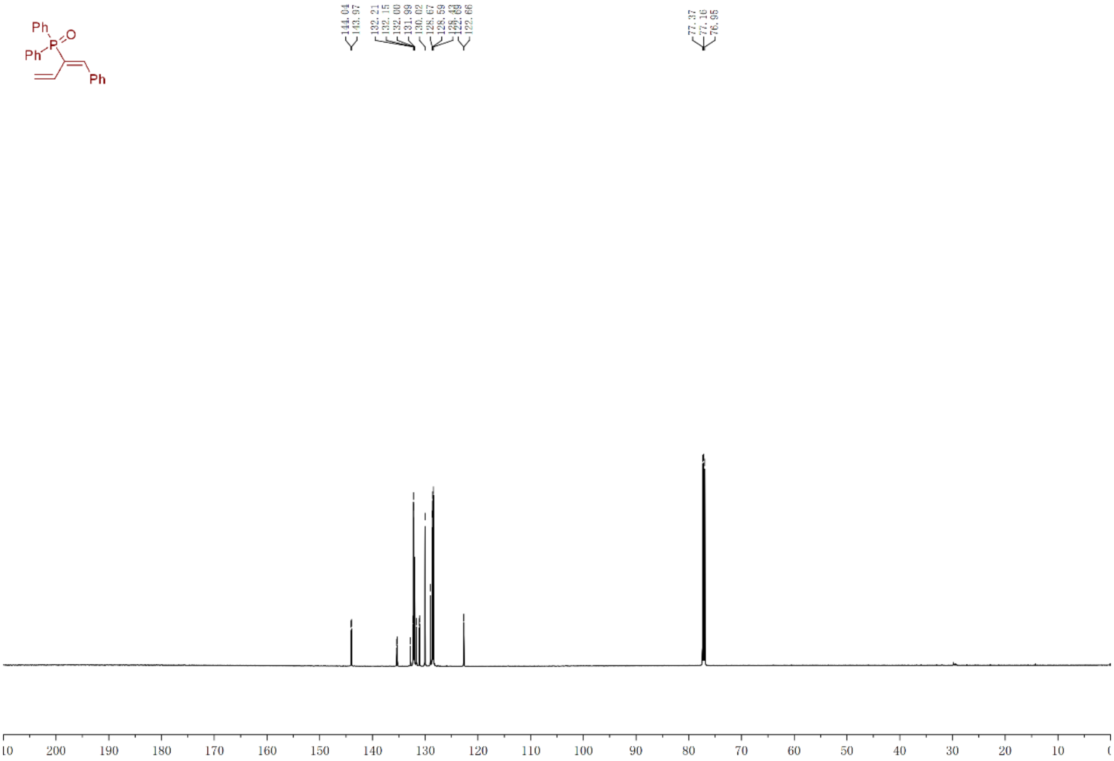

^{31}P NMR of 4b

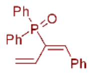

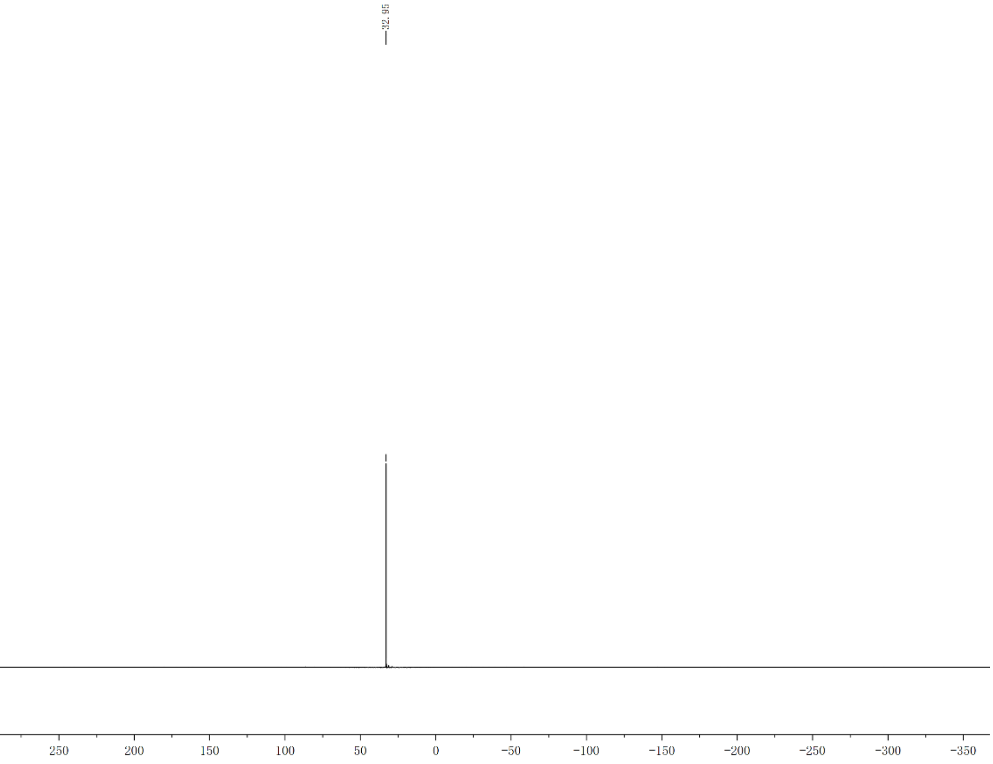

Supplementary Figure 42. ^{1}H NMR, ^{13}C NMR, and ^{31}P NMR spectra of compound **4b**

^{1}H NMR of 4c

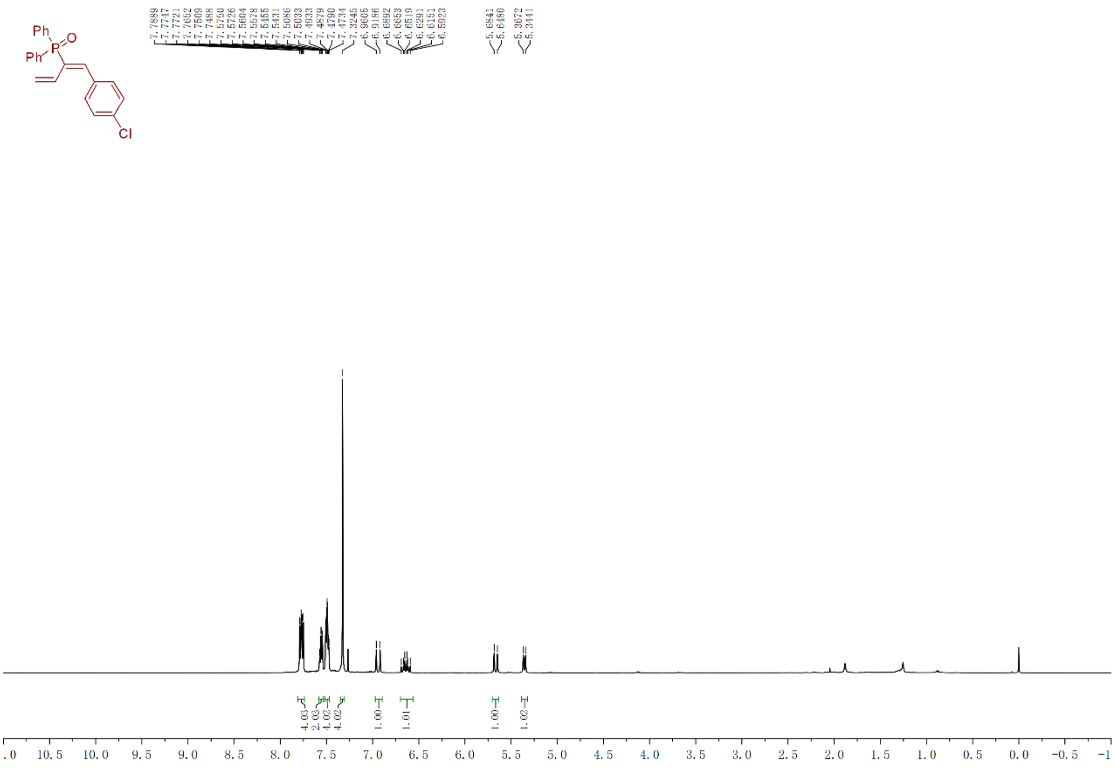

^{13}C NMR of 4c

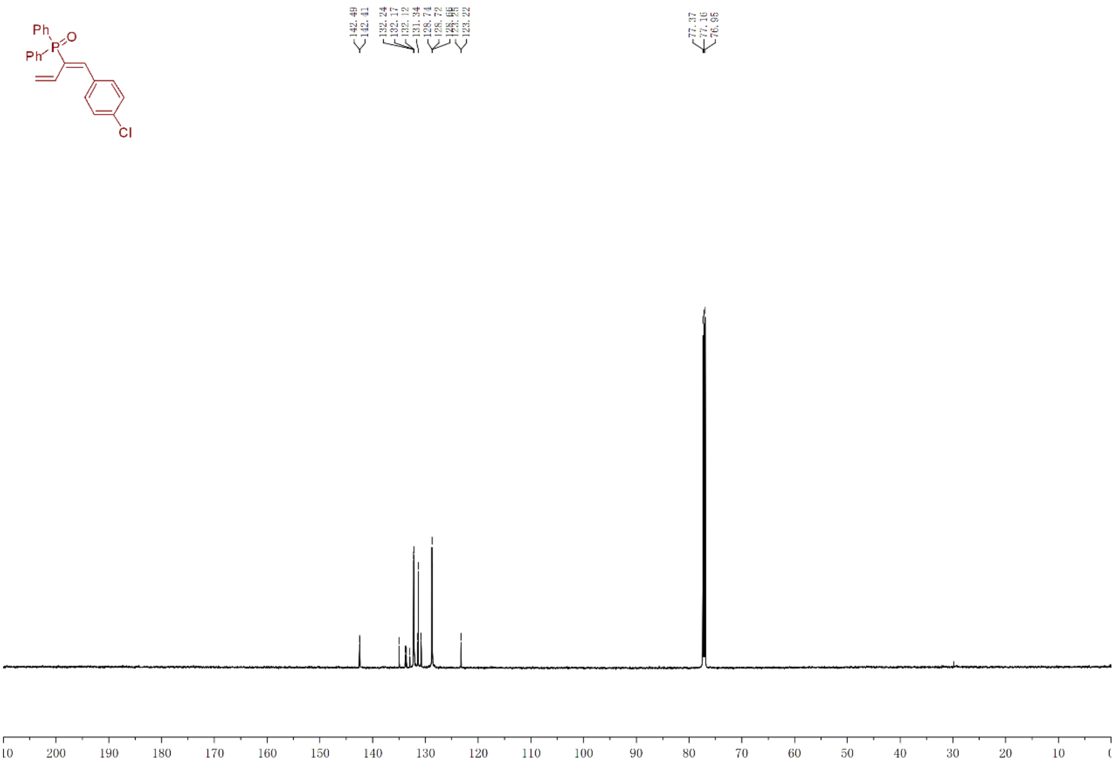

^{31}P NMR of 4c

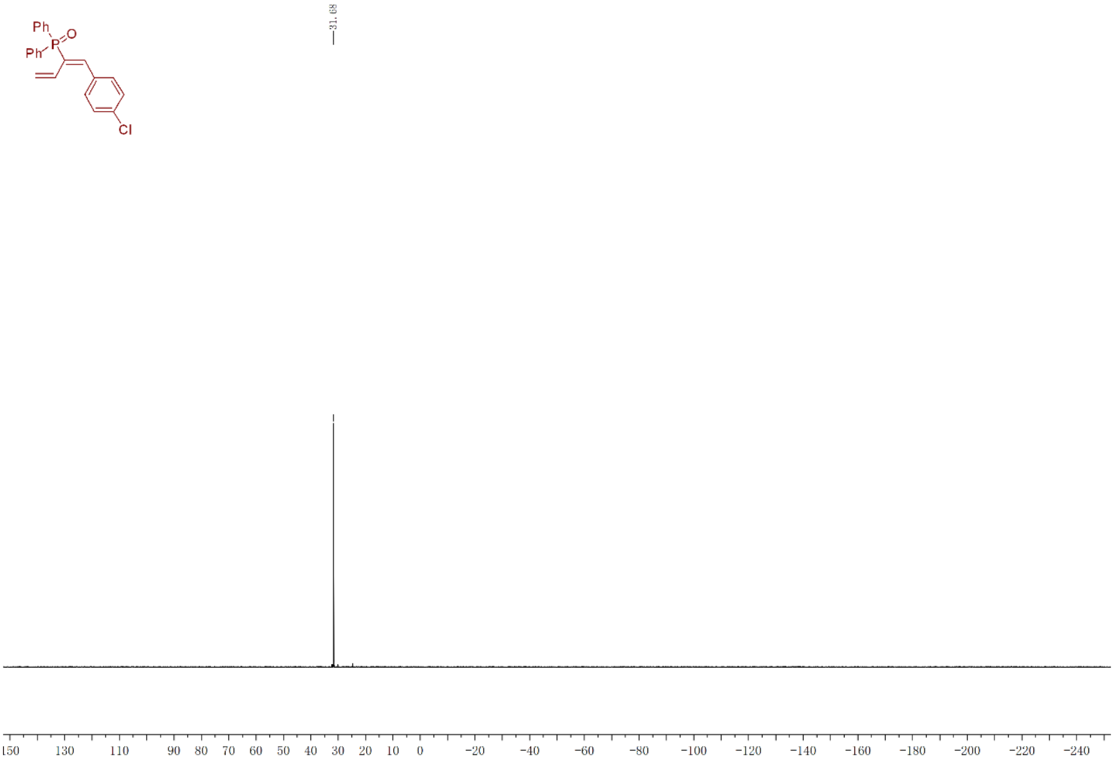

Supplementary Figure 43. ^{1}H NMR, ^{13}C NMR, and ^{31}P NMR spectra of compound **4c**

^{1}H NMR of 4d

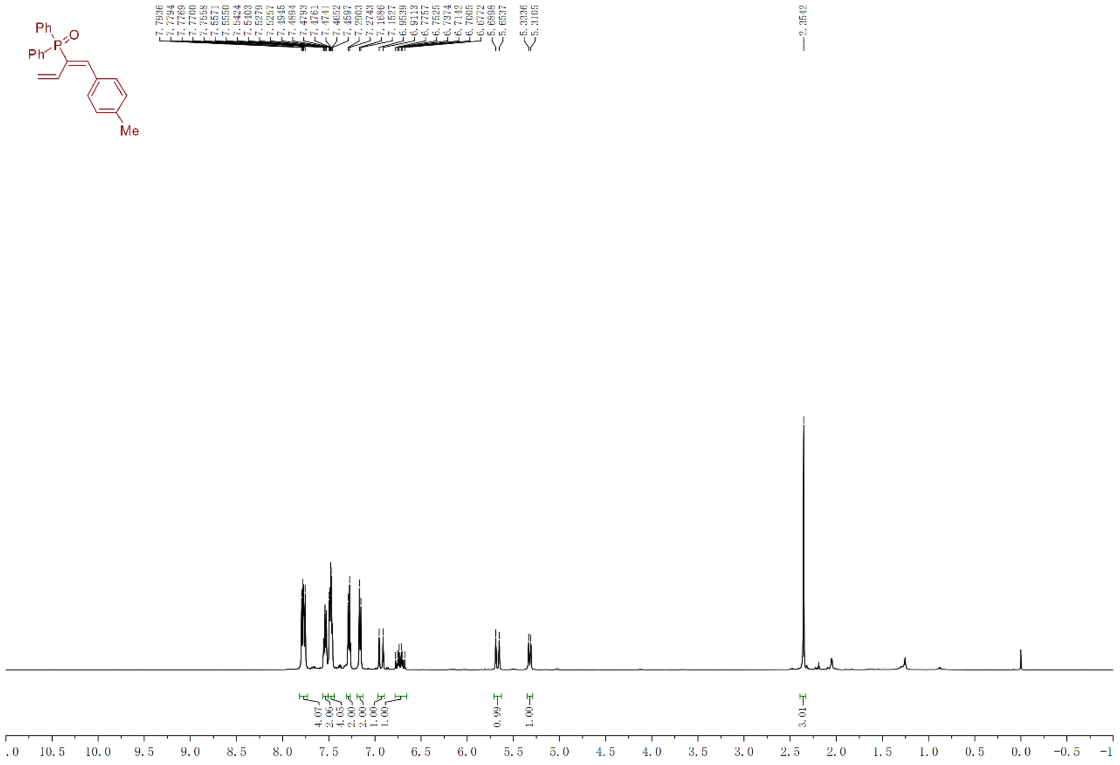

^{13}C NMR of 4d

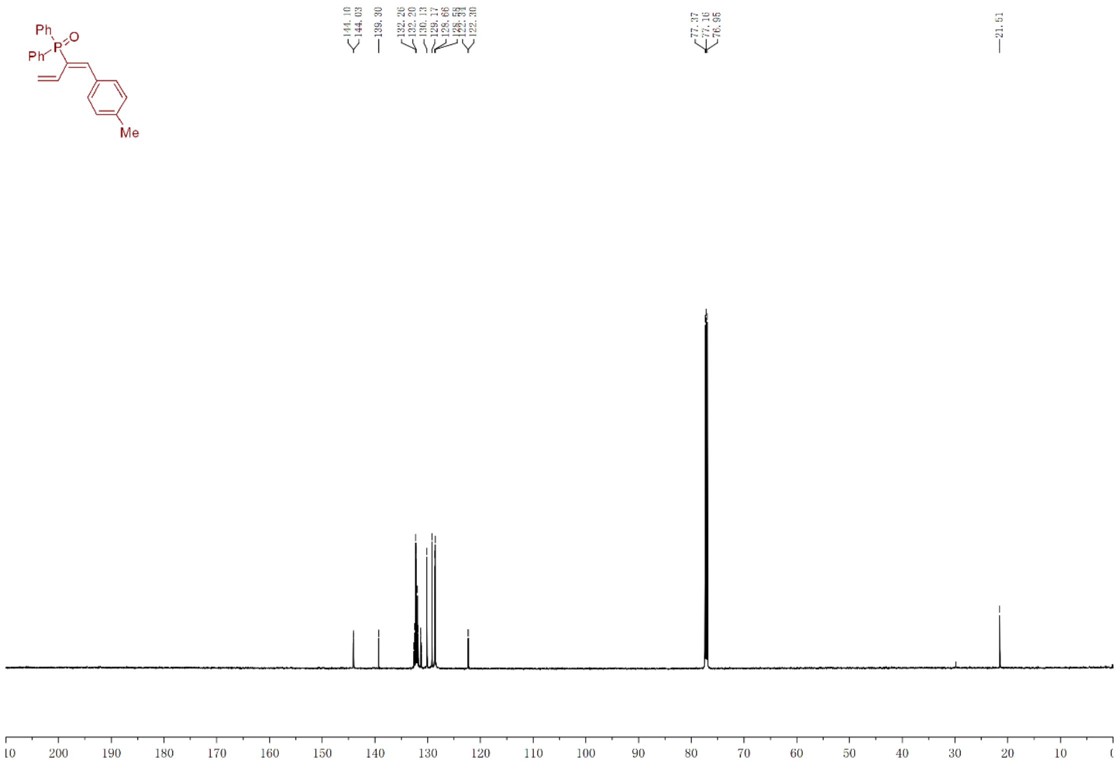

^{31}P NMR of 4d

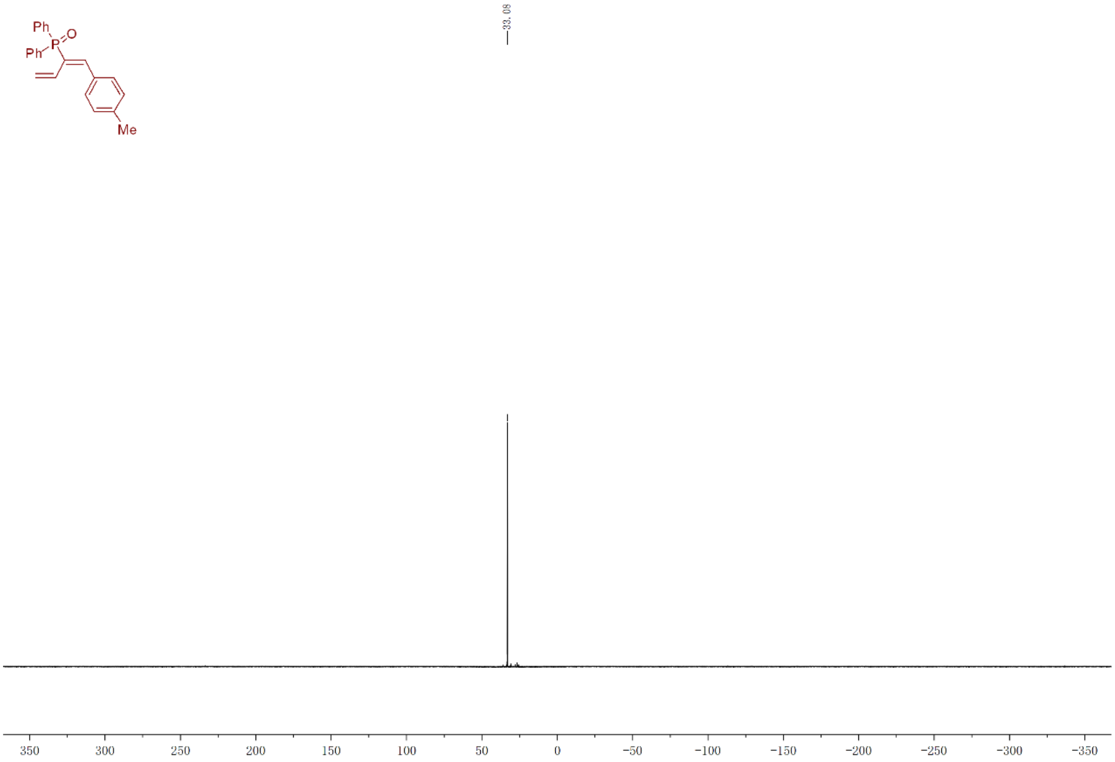

Supplementary Figure 44. ^{1}H NMR, ^{13}C NMR, and ^{31}P NMR spectra of compound **4d**

^{1}H NMR of 4e

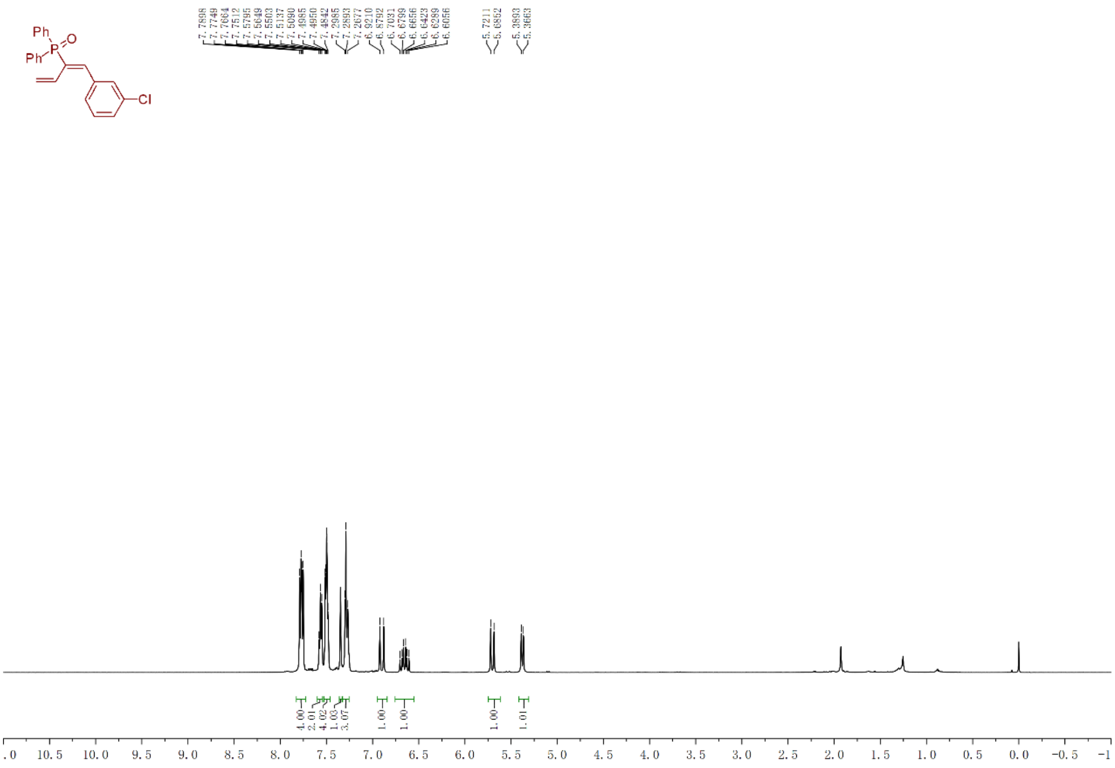

^{13}C NMR of 4e

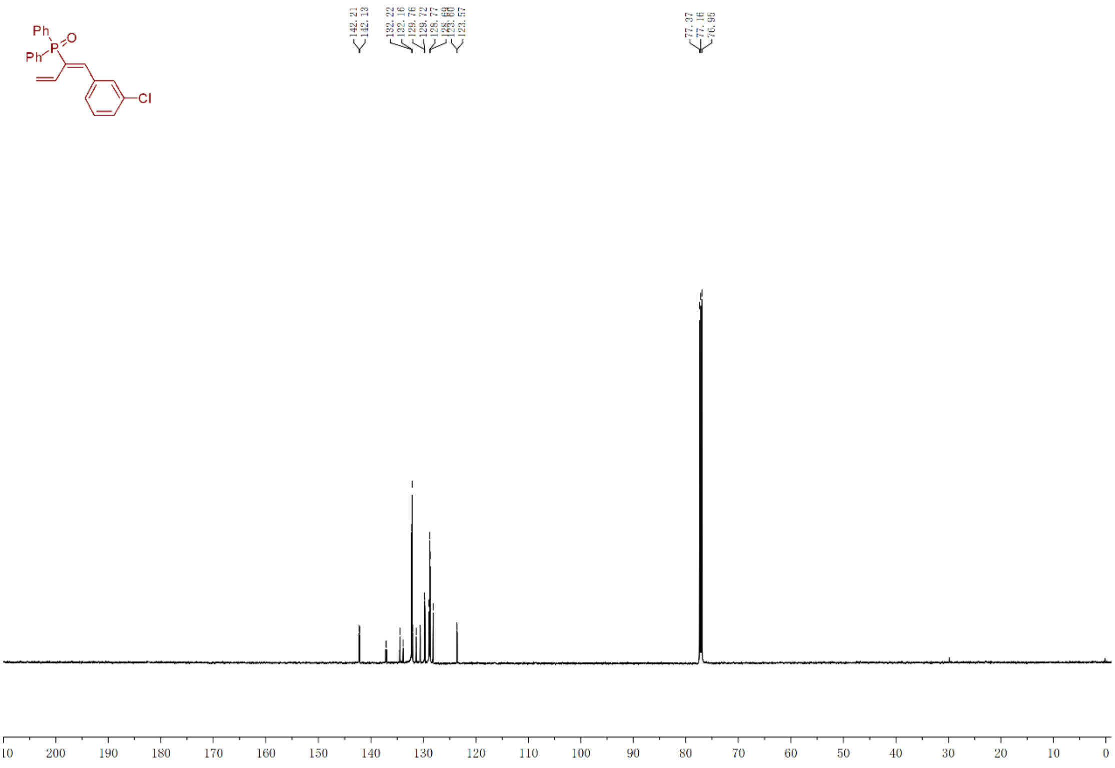

^{31}P NMR of 4e

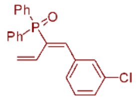

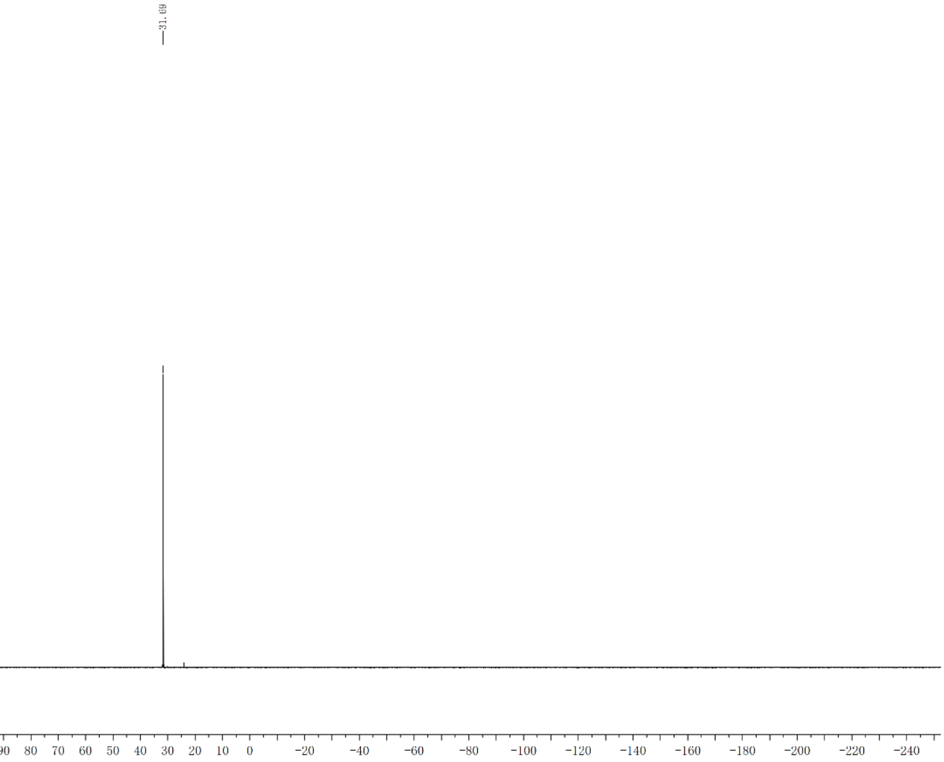

Supplementary Figure 45. ^{1}H NMR, ^{13}C NMR, and ^{31}P NMR spectra of compound **4e**

^{1}H NMR of 4f

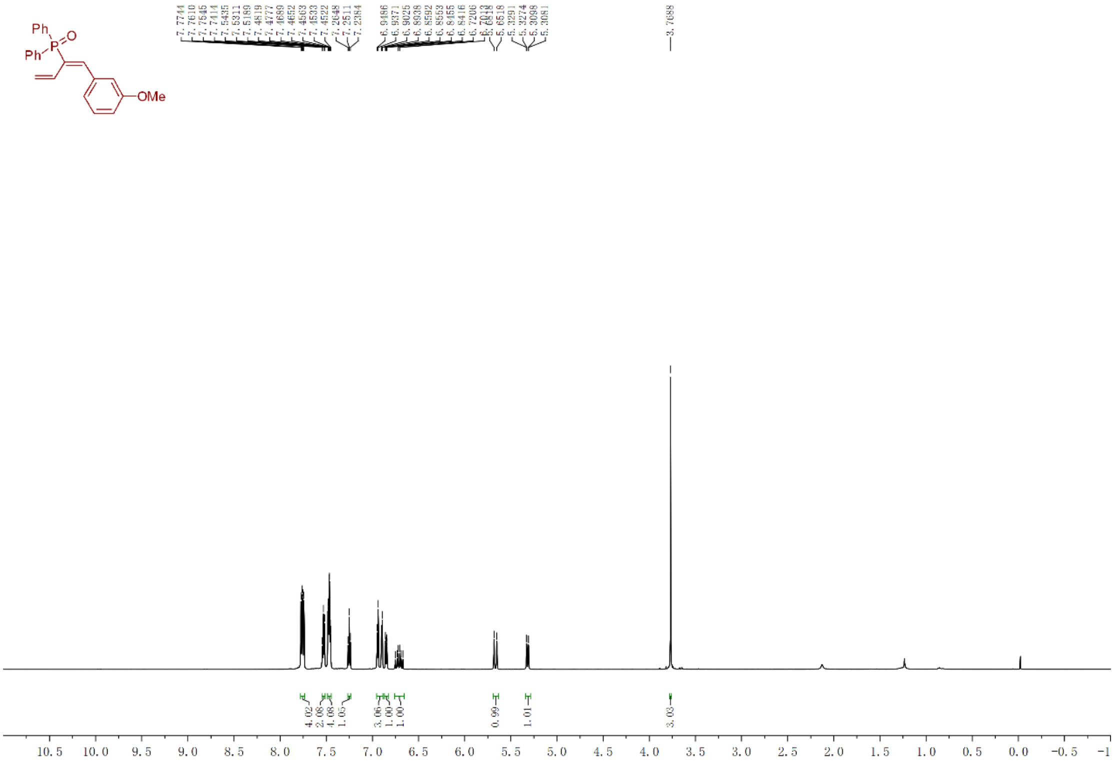

^{13}C NMR of 4f

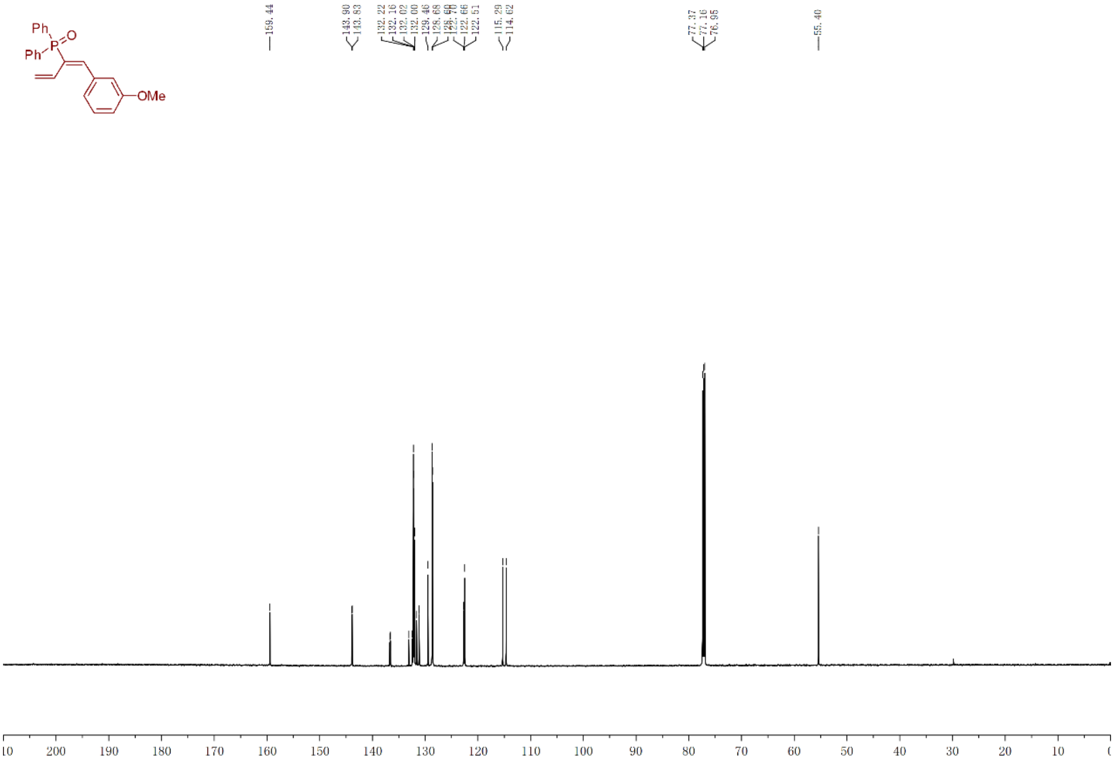

^{31}P NMR of 4f

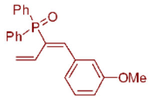

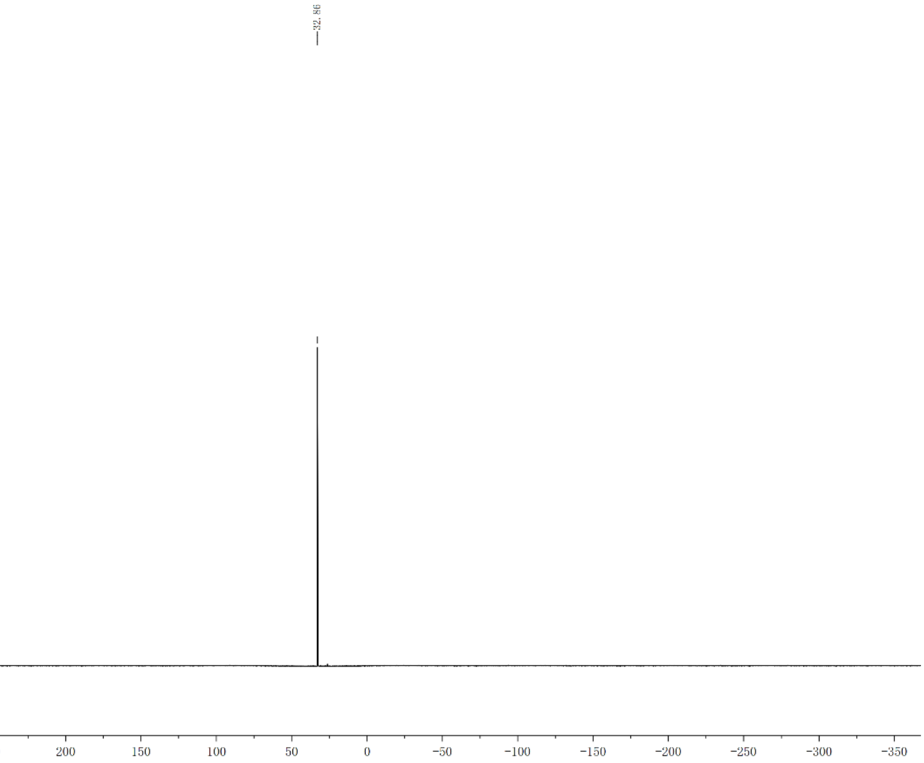

Supplementary Figure 46. ^{1}H NMR, ^{13}C NMR, and ^{31}P NMR spectra of compound **4f**

^{1}H NMR of 4g

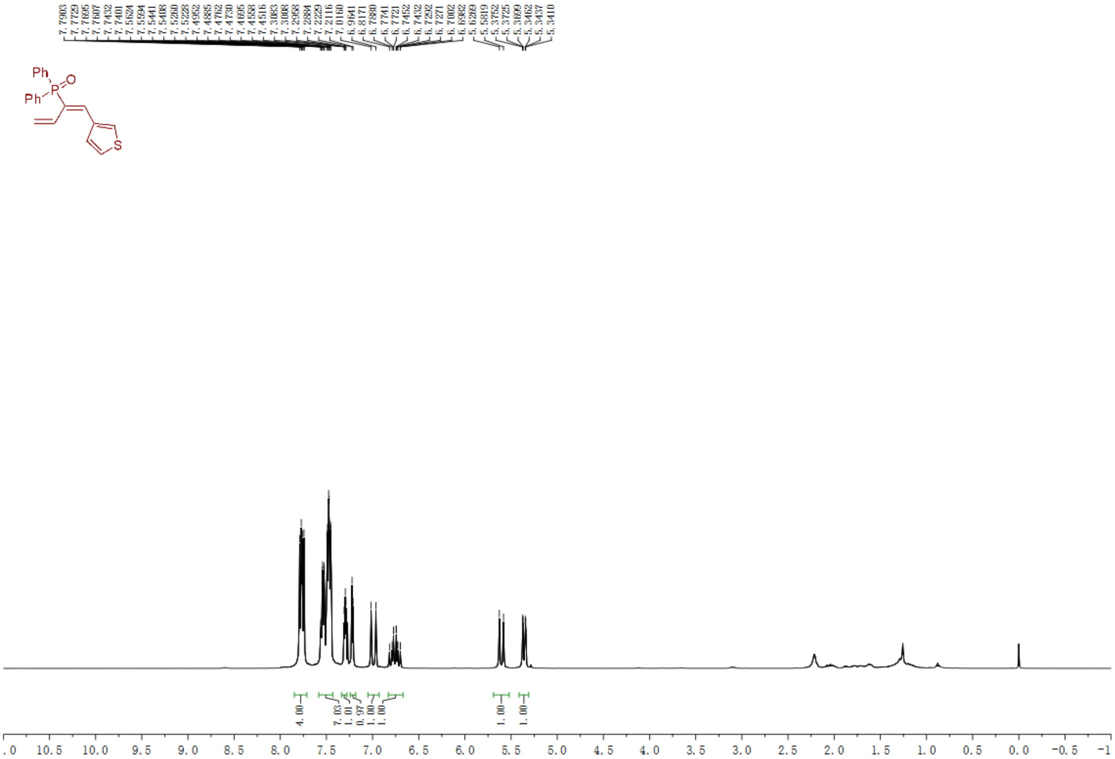

^{13}C NMR of 4g

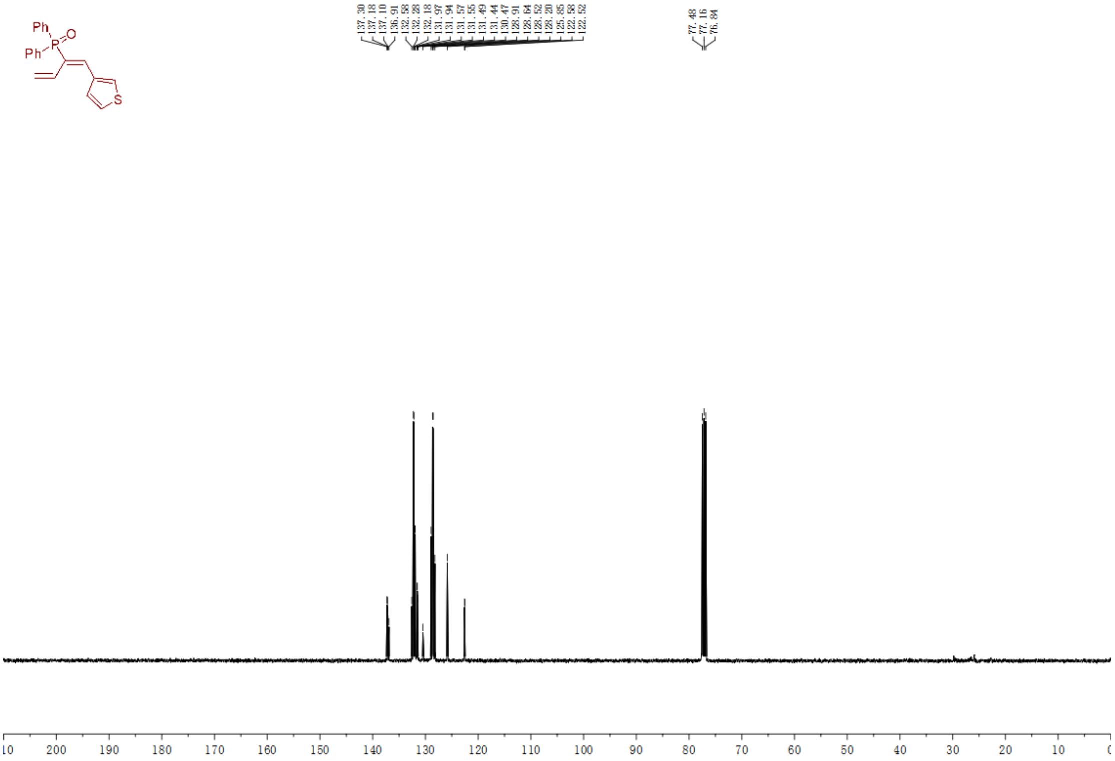

^{31}P NMR of 4g

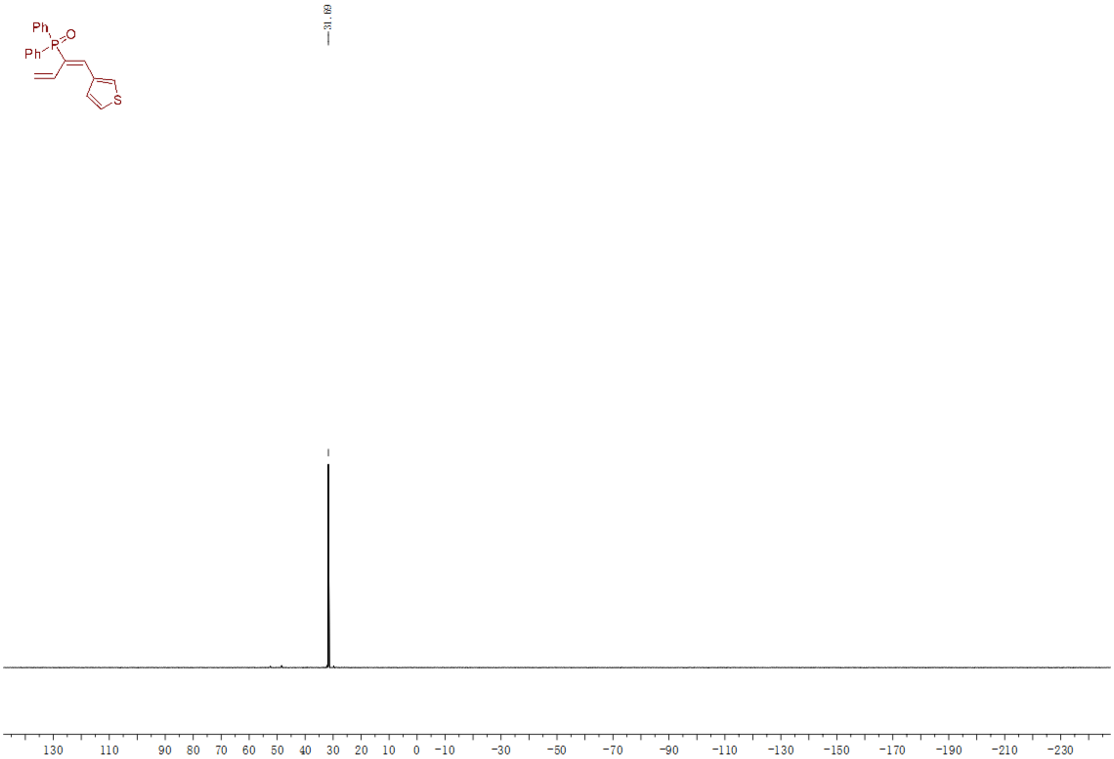

Supplementary Figure 47. ^{1}H NMR, ^{13}C NMR, and ^{31}P NMR spectra of compound **4g**

^{1}H NMR of 4h

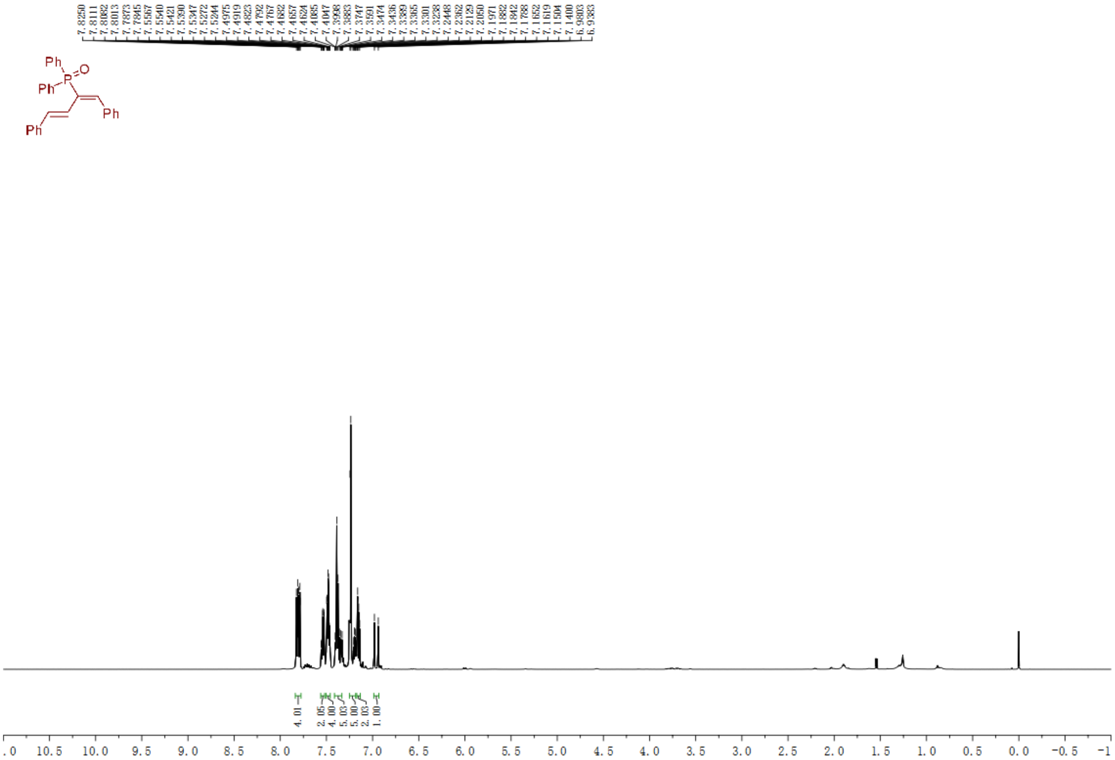

^{13}C NMR of 4h

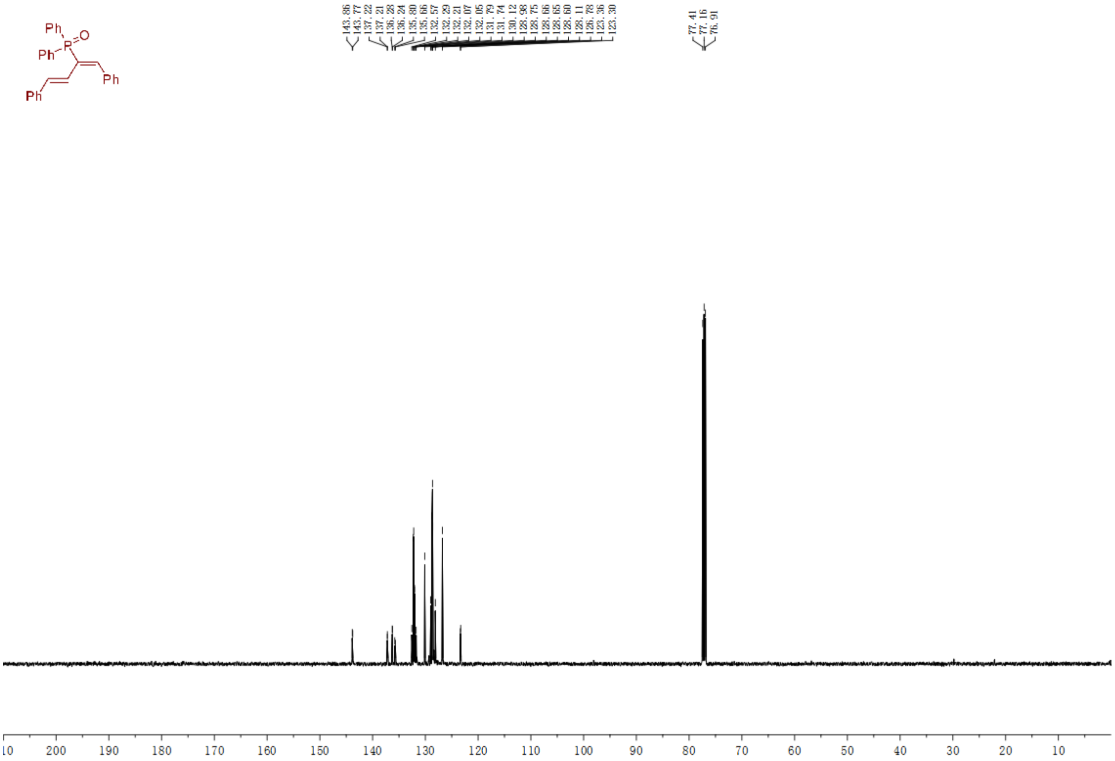

^{31}P NMR of 4h

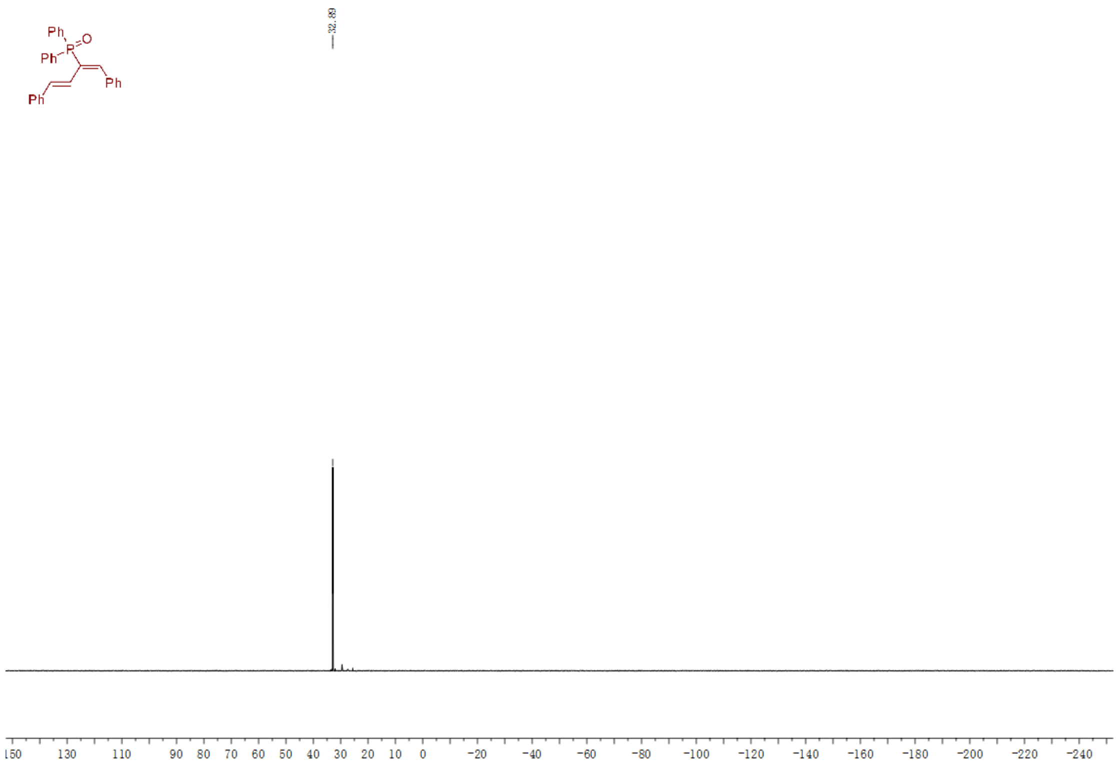

Supplementary Figure 48. ^{1}H NMR, ^{13}C NMR, and ^{31}P NMR spectra of compound **4h**

^{1}H NMR of 4i

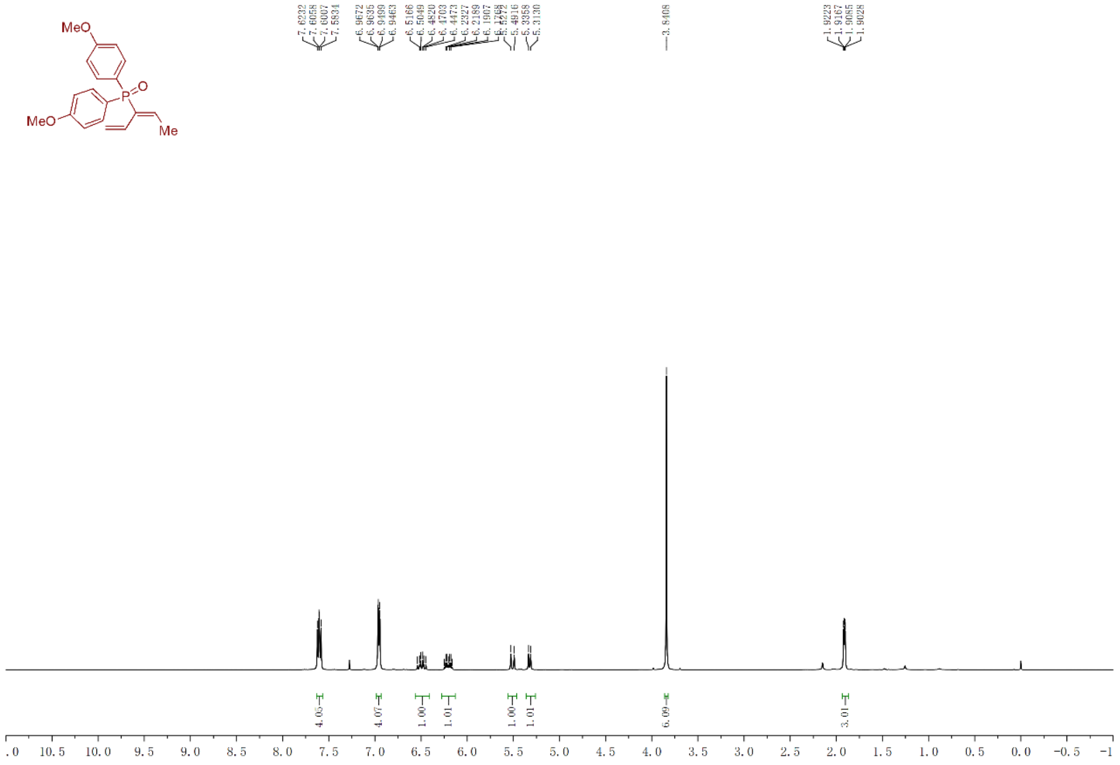

^{13}C NMR of 4i

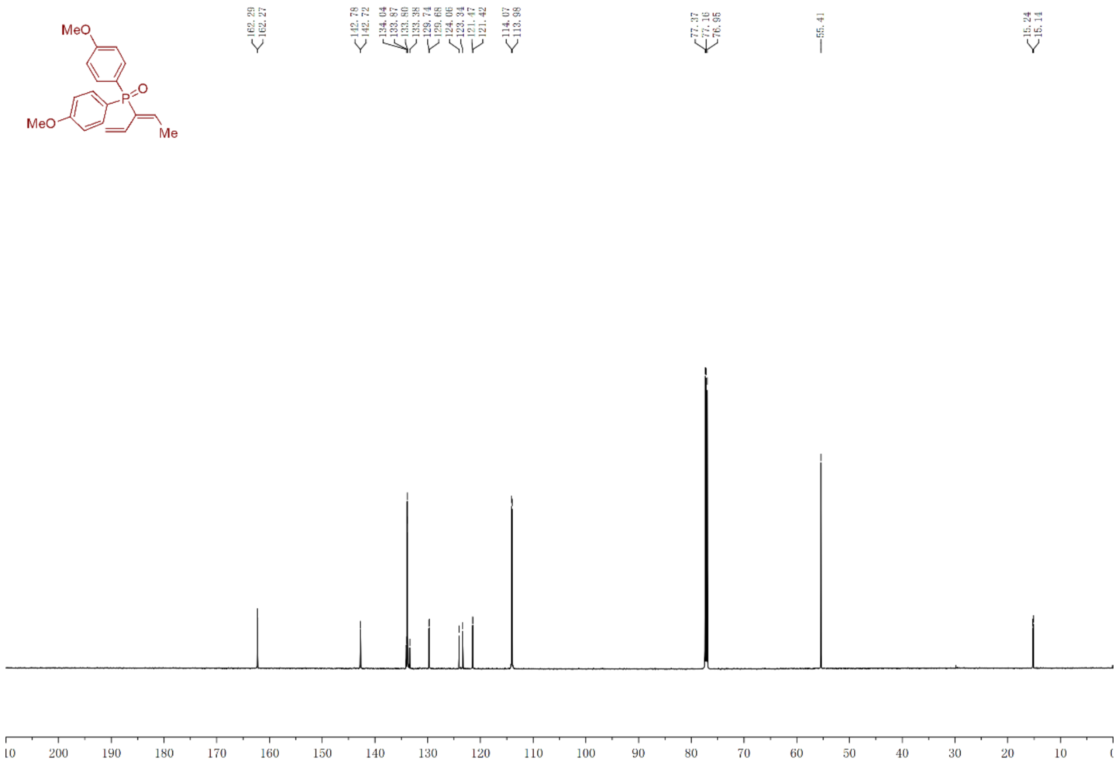

^{31}P NMR of 4i

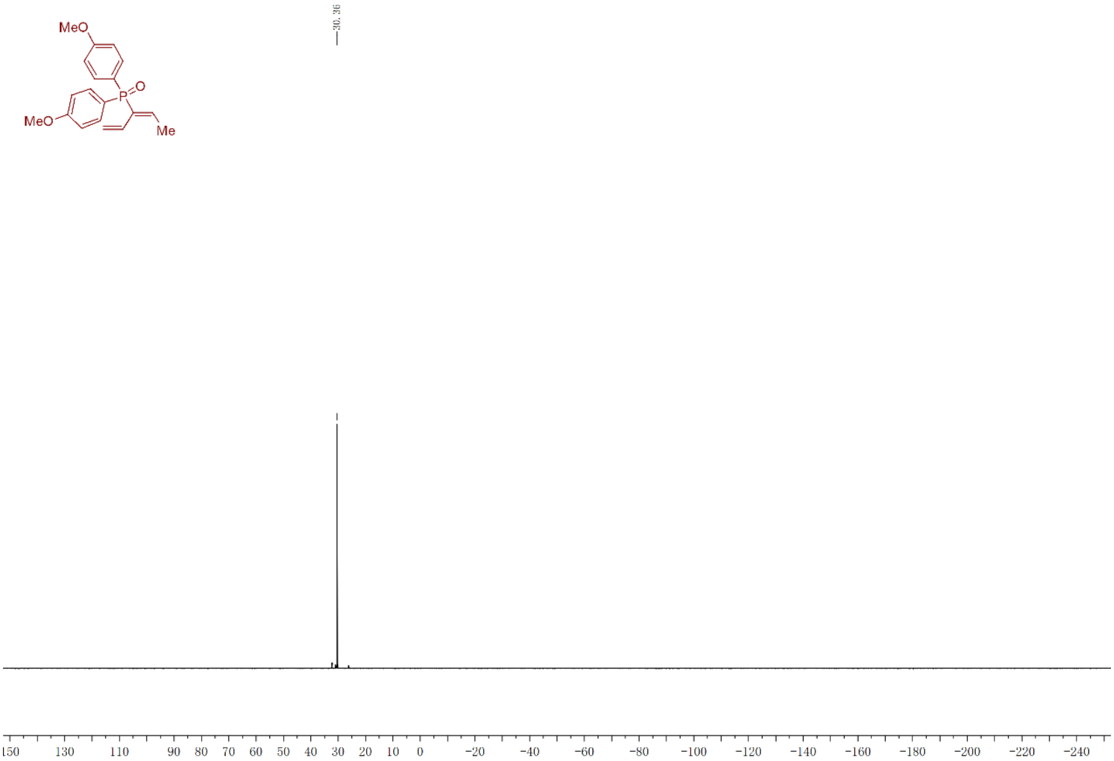

Supplementary Figure 49. ^{1}H NMR, ^{13}C NMR, and ^{31}P NMR spectra of compound **4i**

^{1}H NMR of 4j

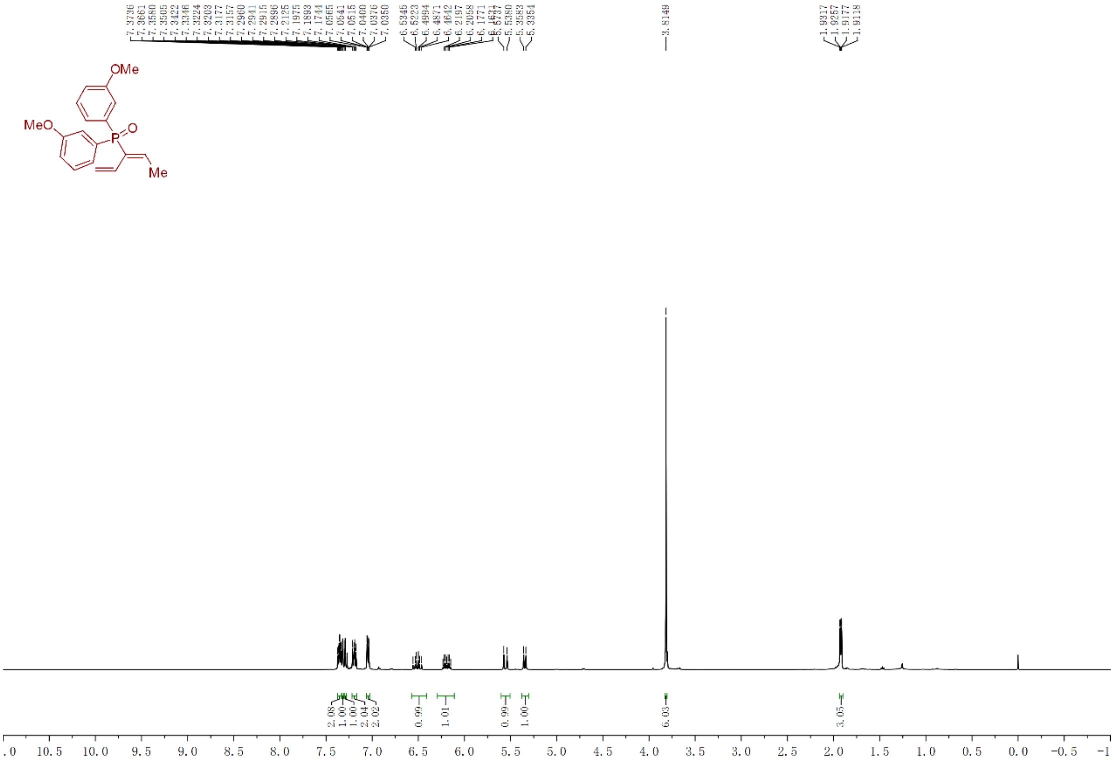

^{13}C NMR of 4j

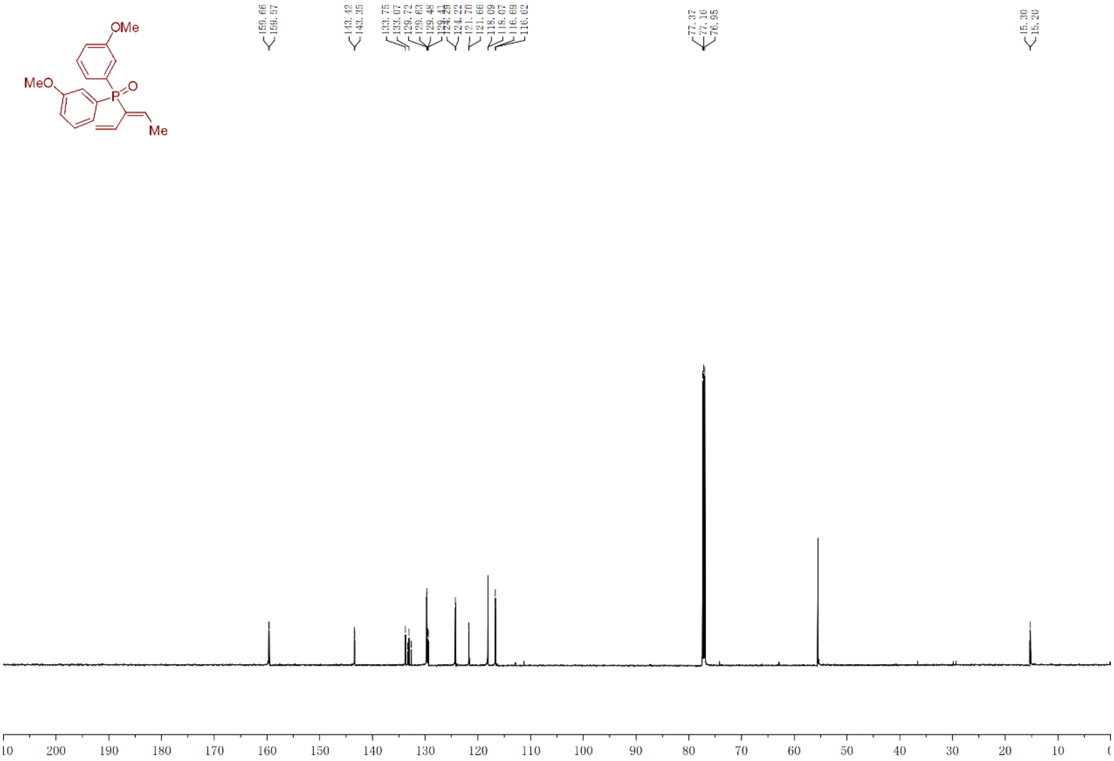

^{31}P NMR of 4j

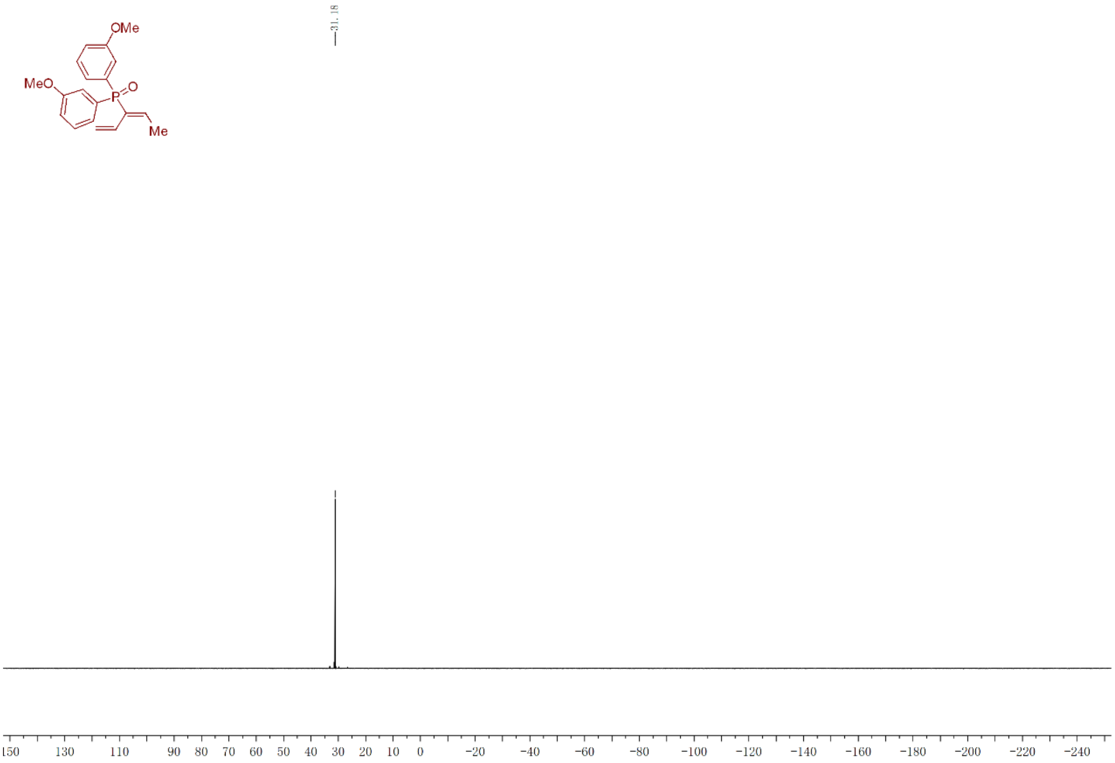

Supplementary Figure 50. ^{1}H NMR, ^{13}C NMR, and ^{31}P NMR spectra of compound **4j**

^{1}H NMR of 4k

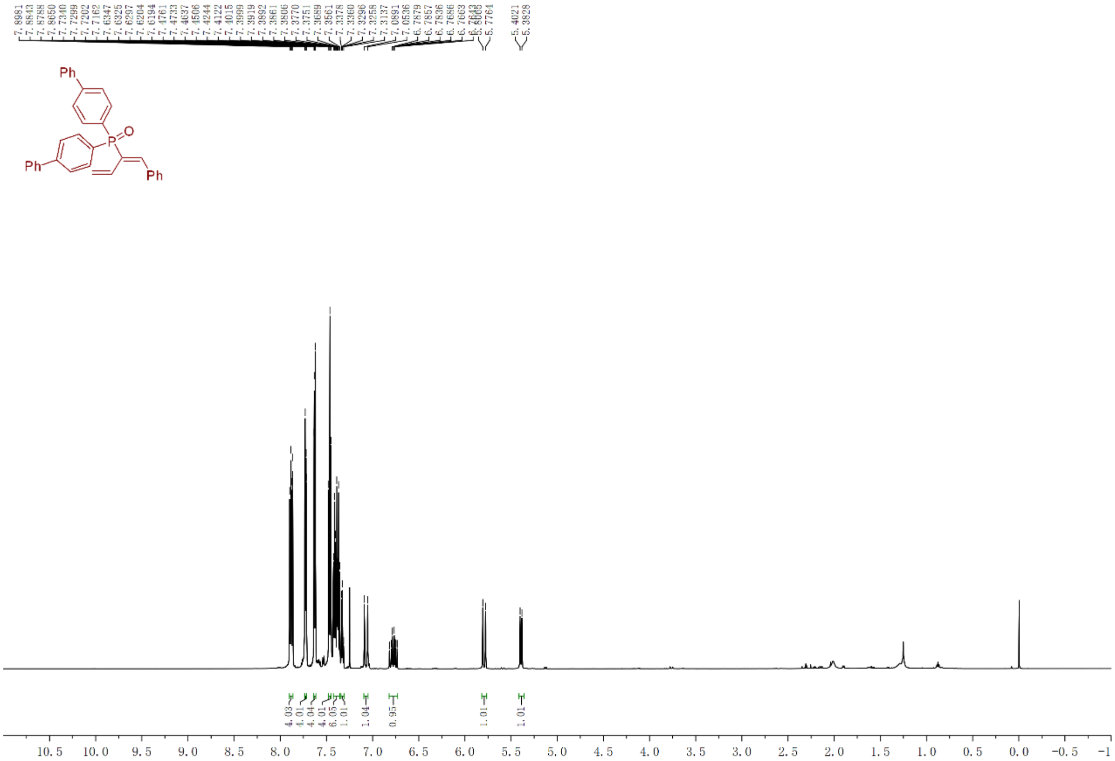

^{13}C NMR of 4k

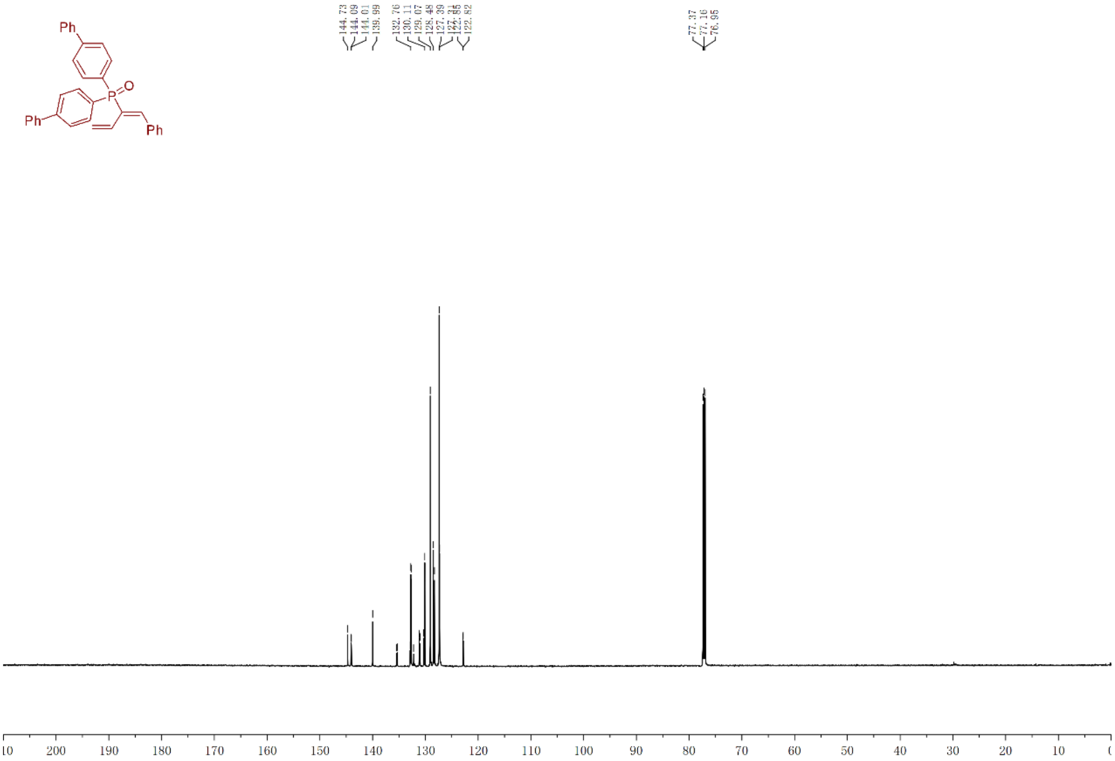

^{31}P NMR of 4k

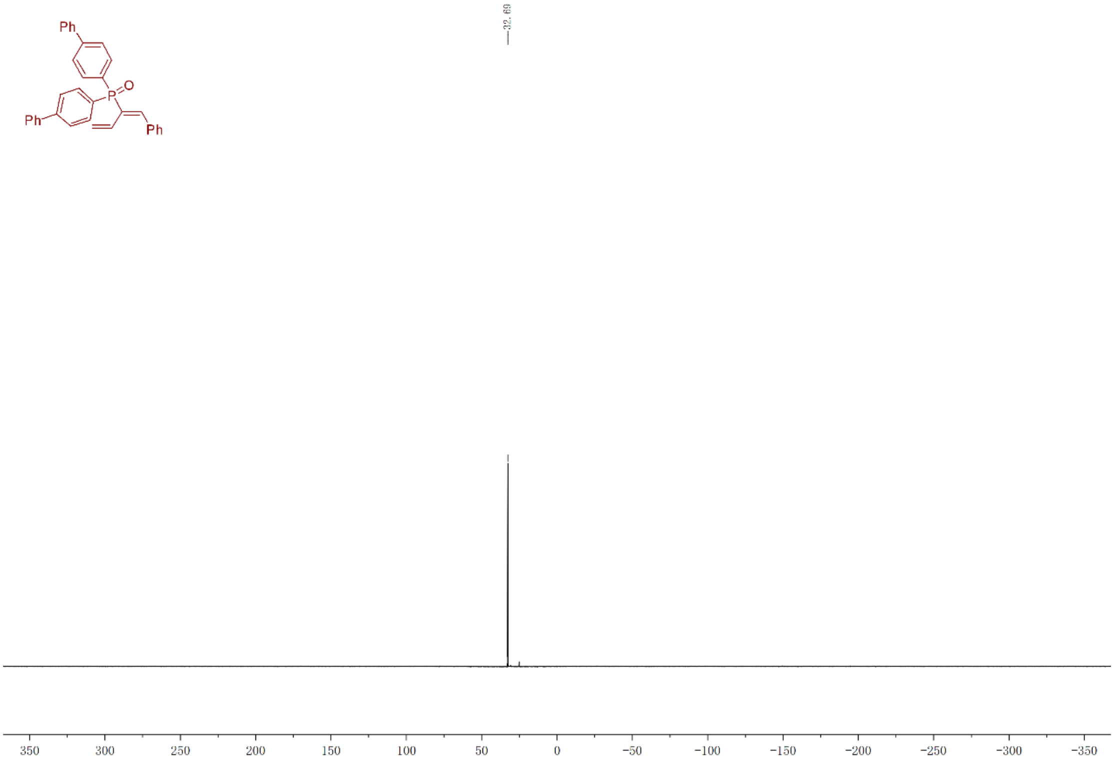

Supplementary Figure 51. ^{1}H NMR, ^{13}C NMR, and ^{31}P NMR spectra of compound **4k**

^{1}H NMR of 4l

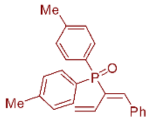

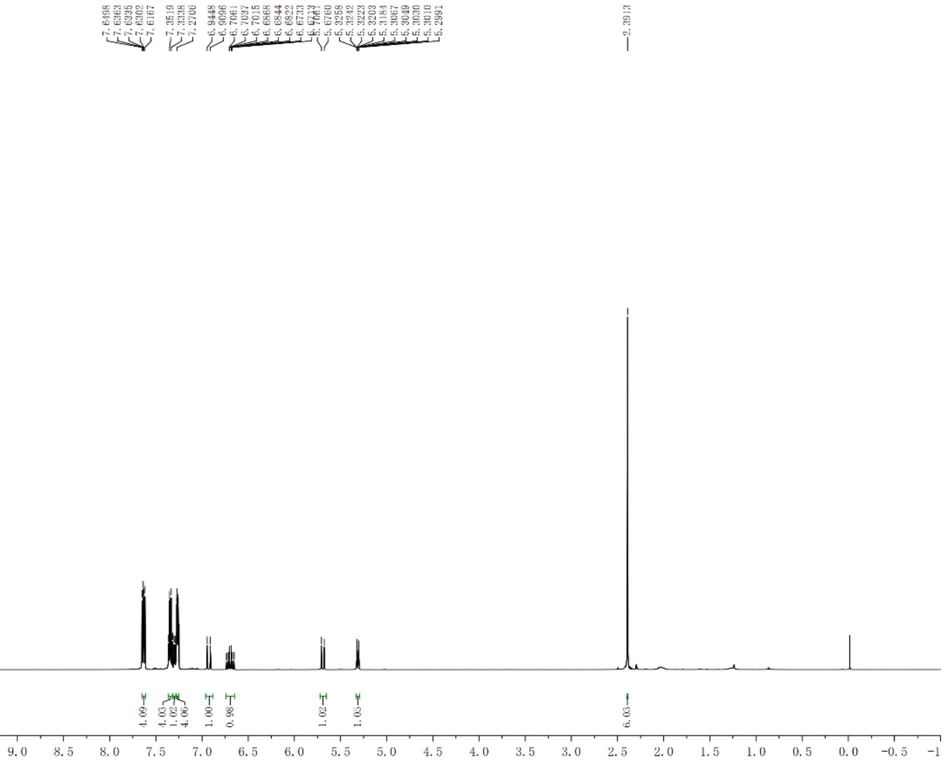

^{13}C NMR of 4l

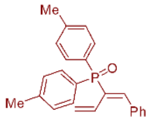

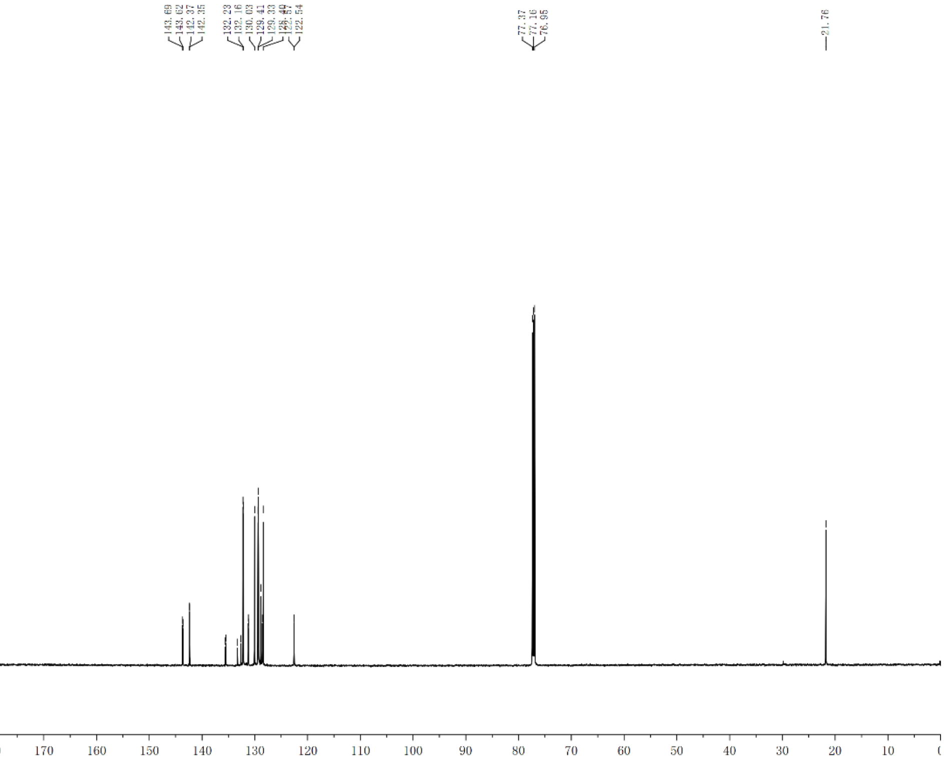

^{31}P NMR of 4l

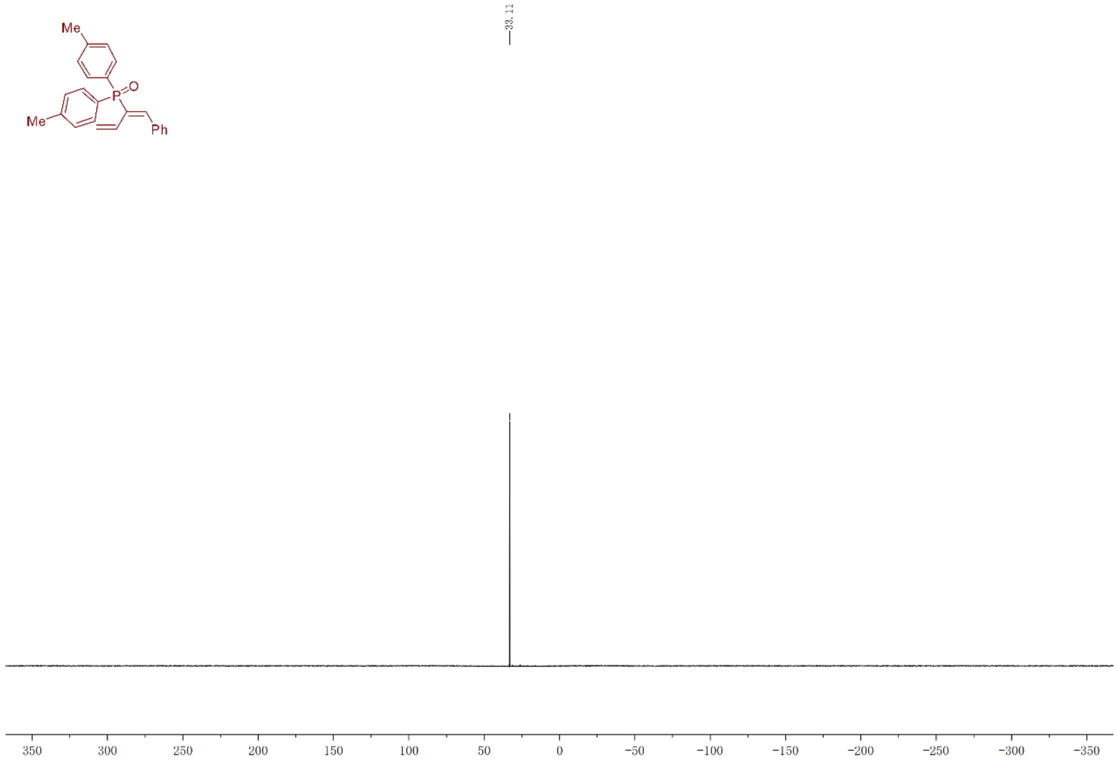

Supplementary Figure 52. ^{1}H NMR, ^{13}C NMR, and ^{31}P NMR spectra of compound **4l**

^{1}H NMR of 4m

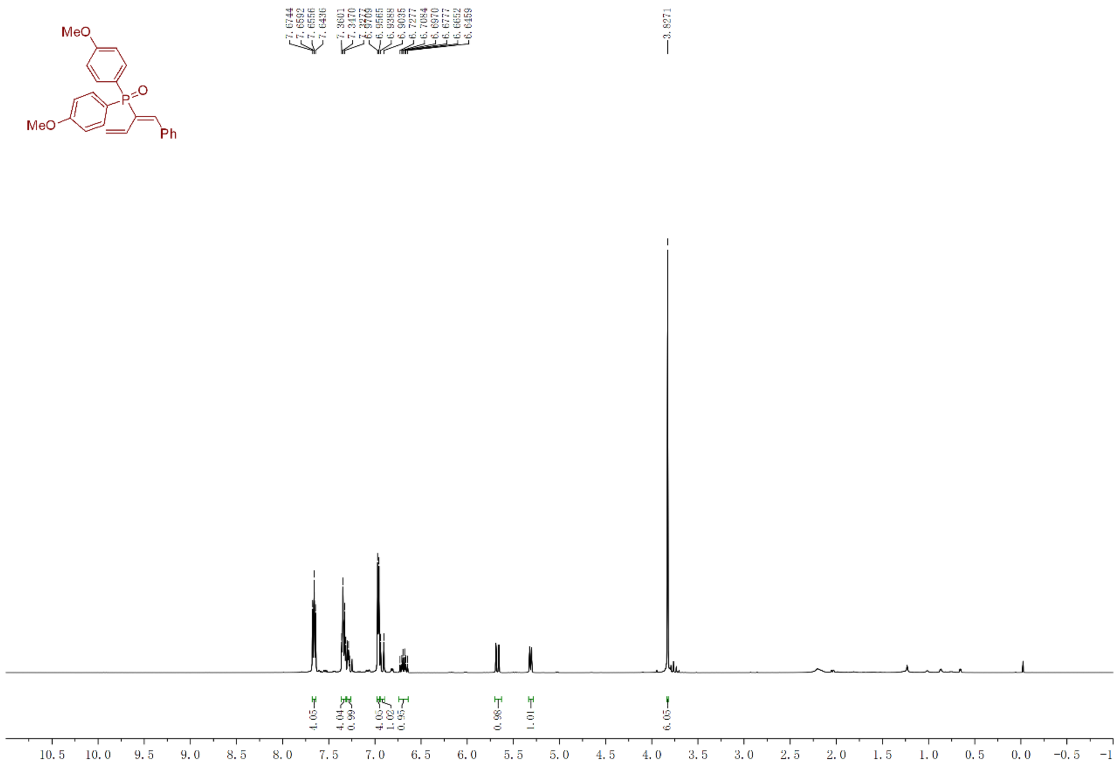

^{13}C NMR of 4m

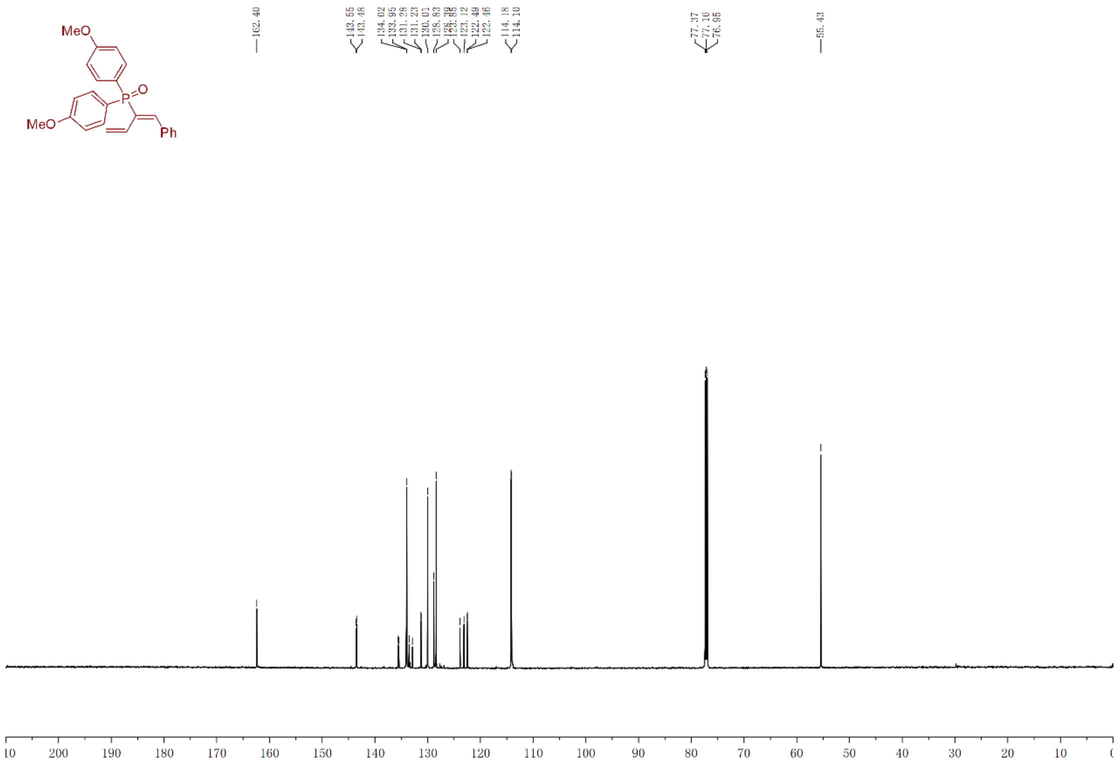

^{31}P NMR of 4m

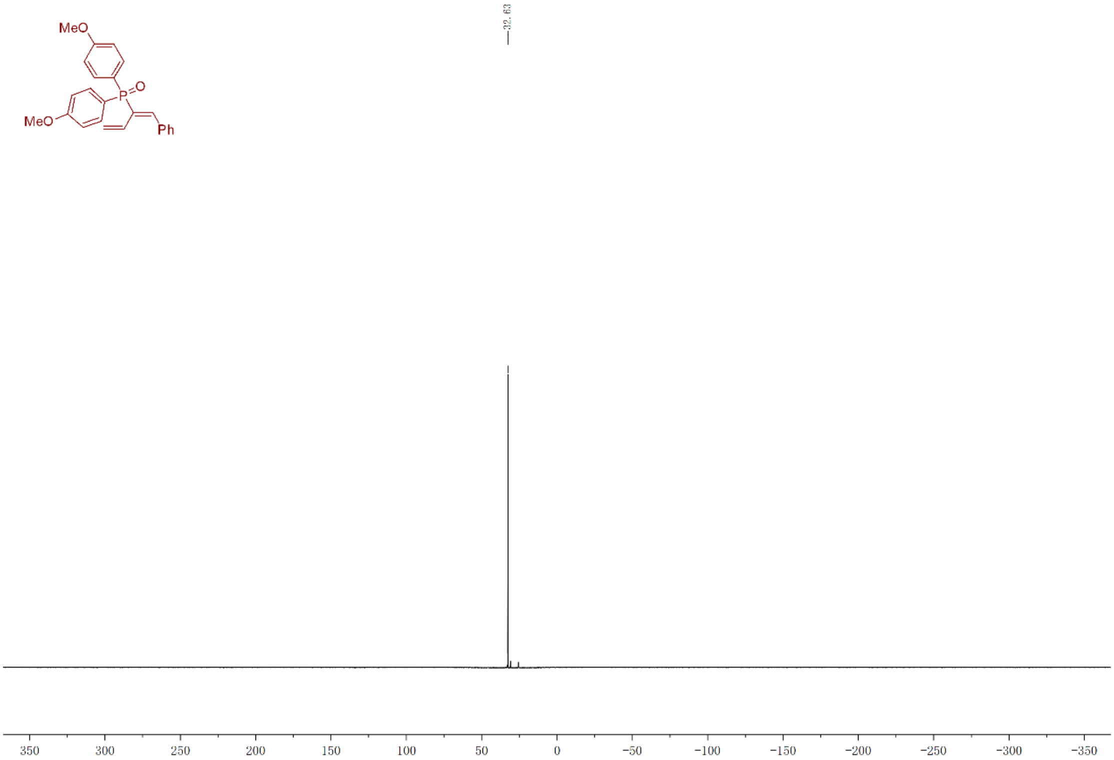

Supplementary Figure 53. ^{1}H NMR, ^{13}C NMR, and ^{31}P NMR spectra of compound **4m**

^{1}H NMR of 4n

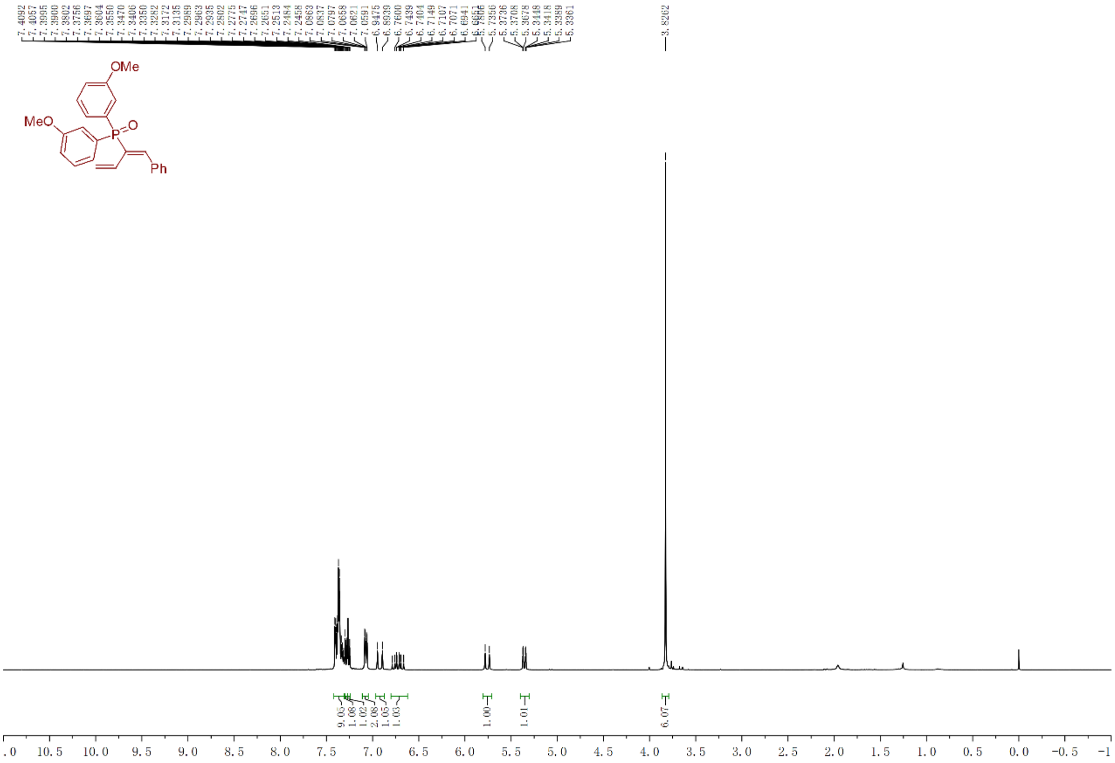

^{13}C NMR of 4n

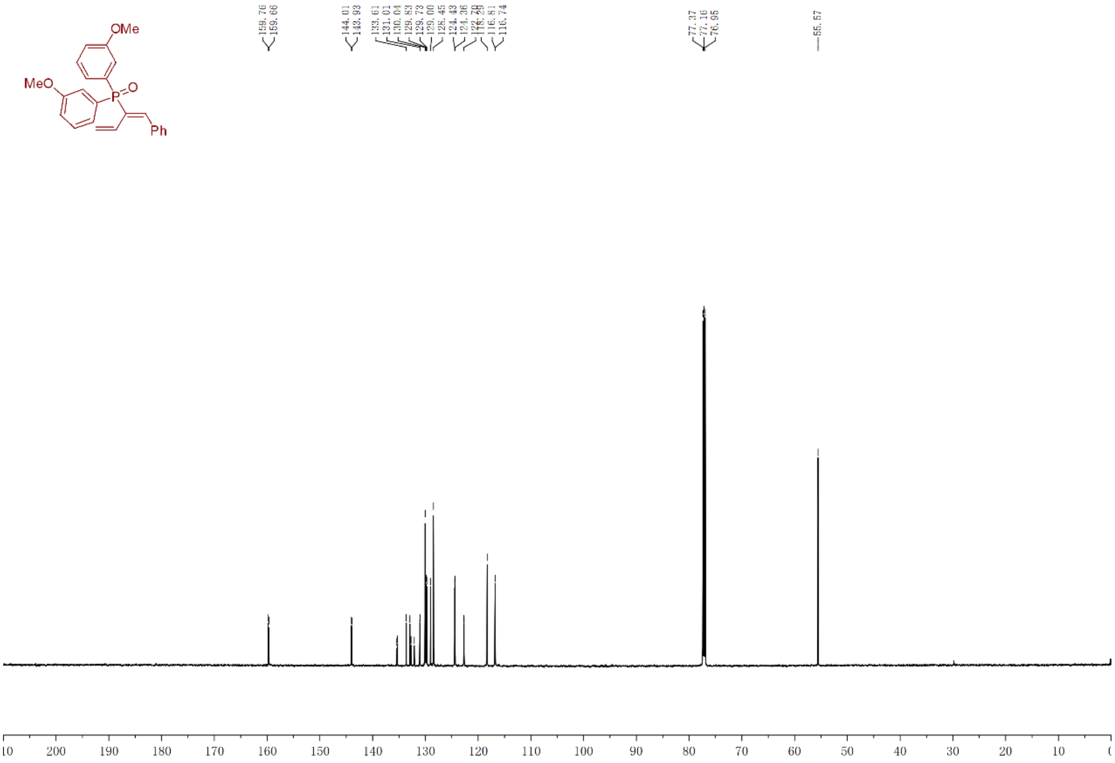

^{31}P NMR of 4n

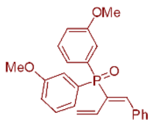

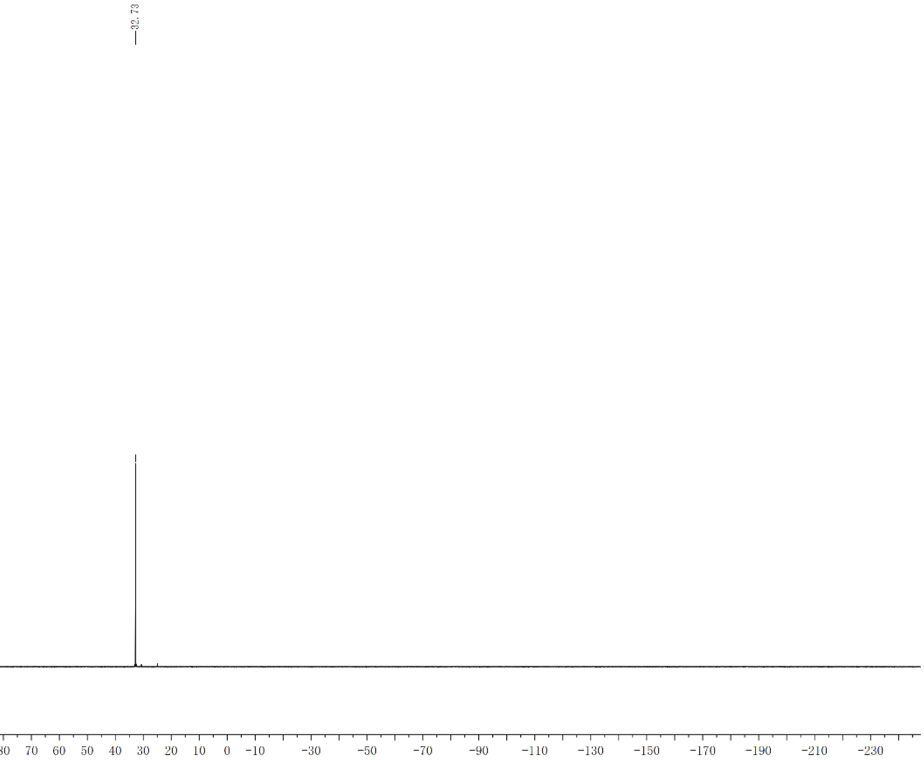

Supplementary Figure 54. ^{1}H NMR, ^{13}C NMR, and ^{31}P NMR spectra of compound **4n**

^{1}H NMR of 4o (CD_3OD-d_4)

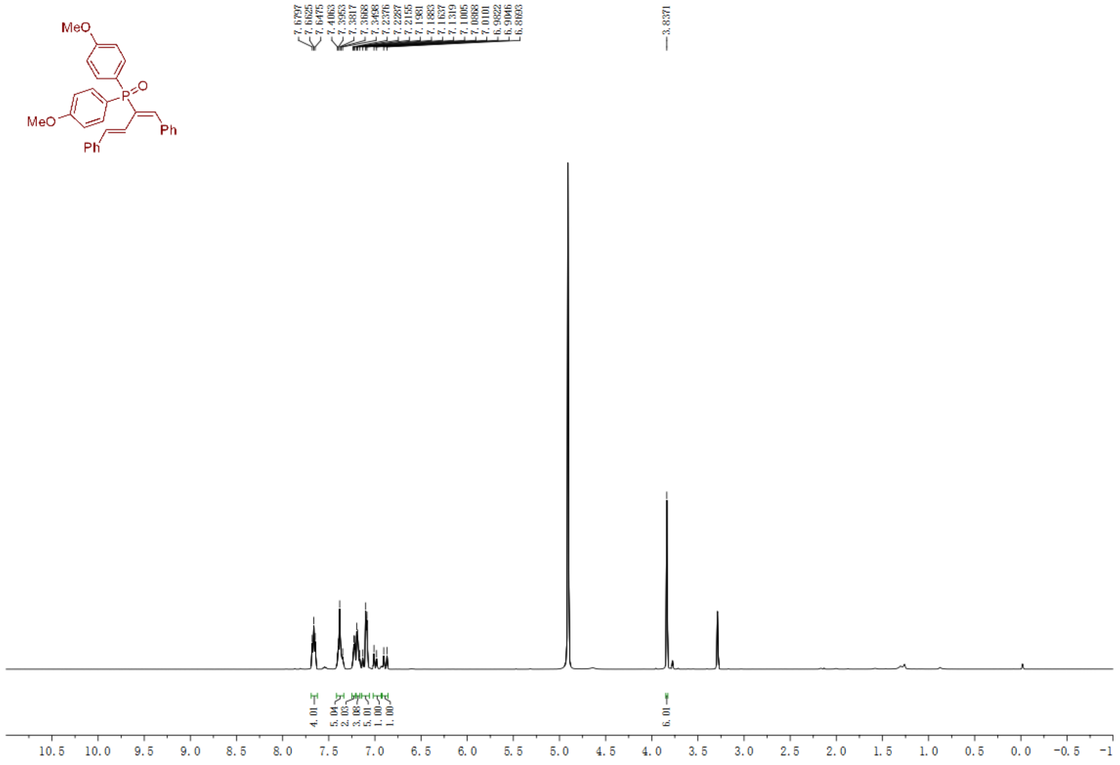

^{1}H NMR of 4o ($CDCl_3$)

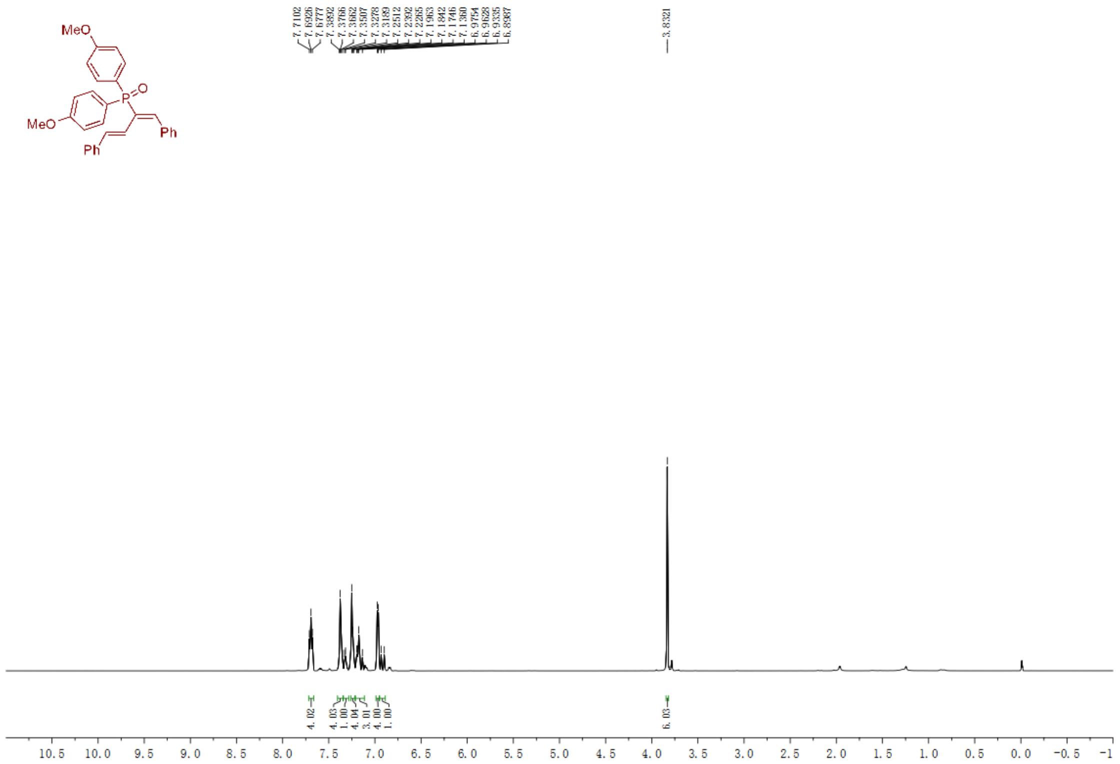

^{13}C NMR of 4o

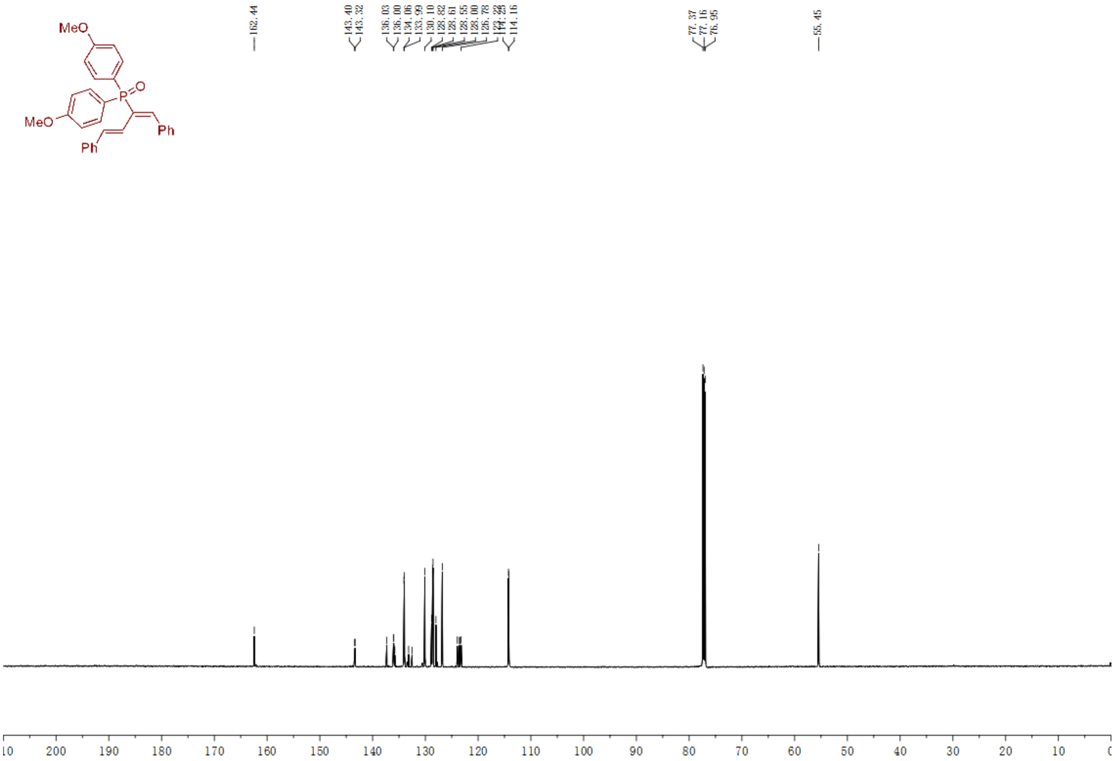

^{31}P NMR of 4o

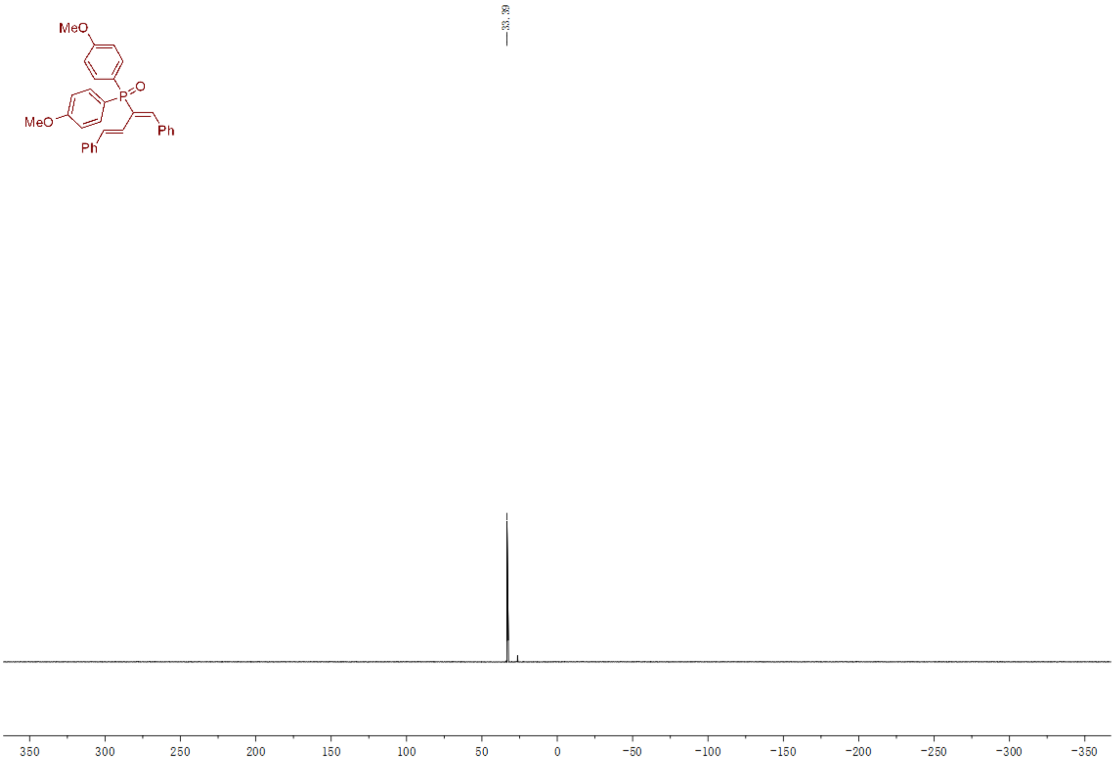

Supplementary Figure 55. ^{1}H NMR, ^{13}C NMR, and ^{31}P NMR spectra of compound **4o**

^{1}H NMR of 5

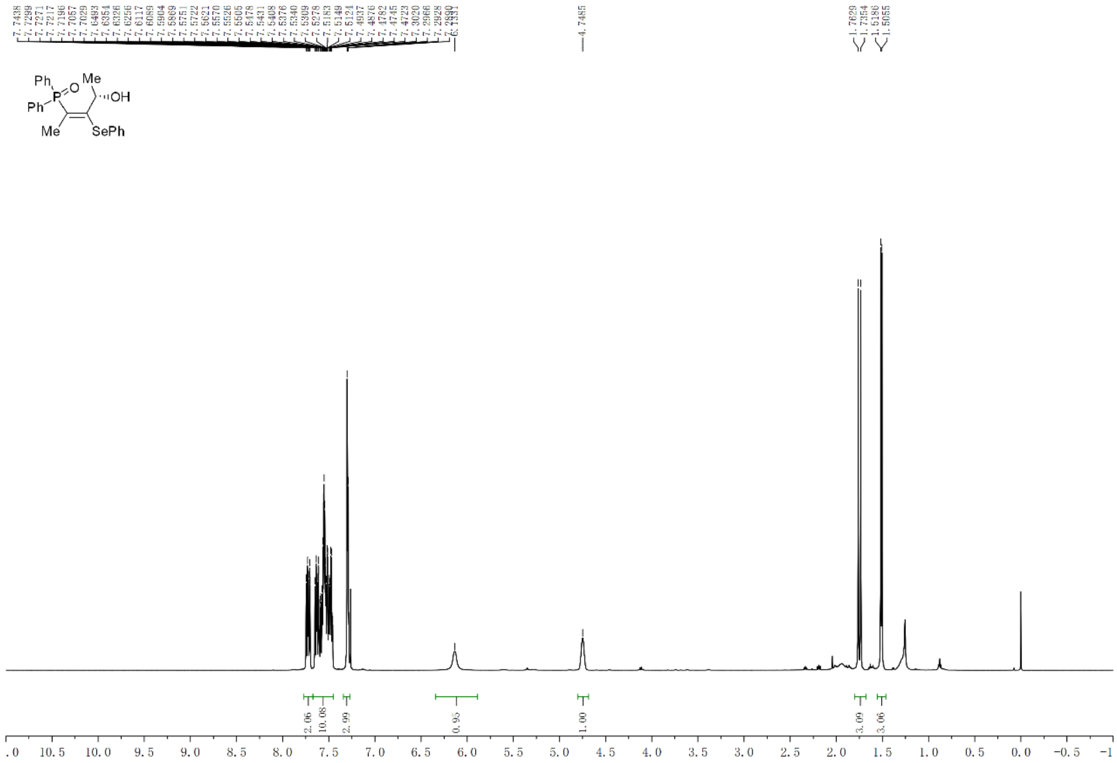

^{13}C NMR of 5

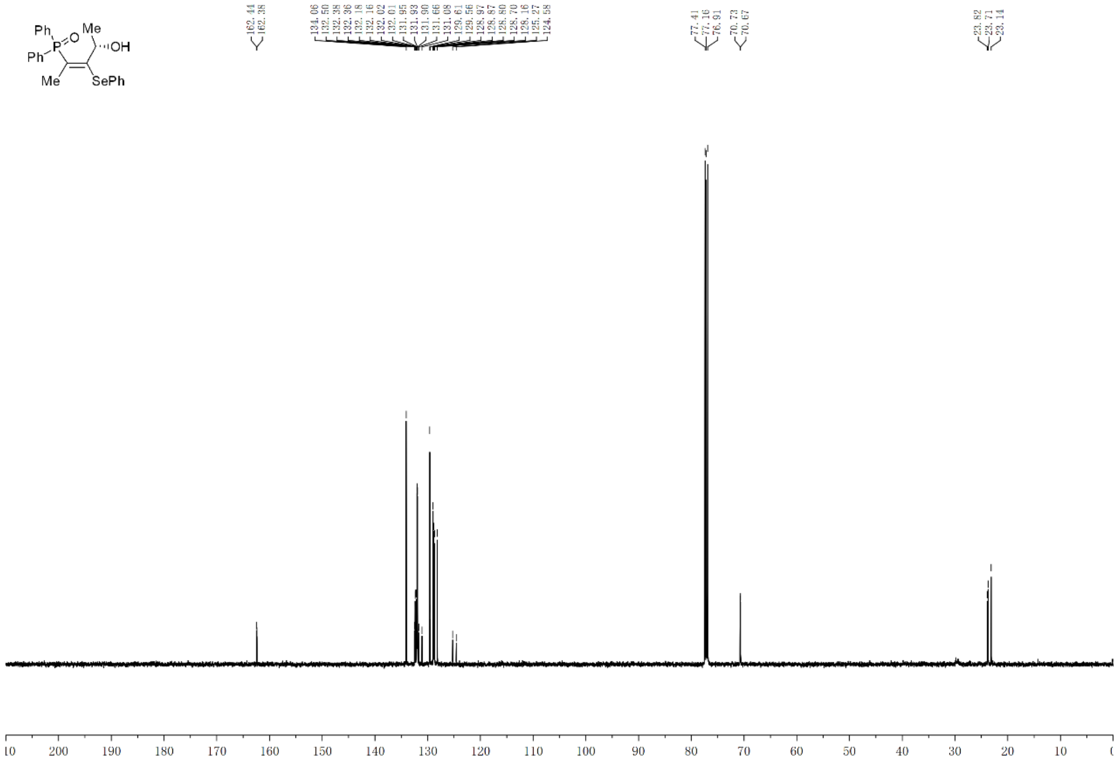

^{31}P NMR of 5

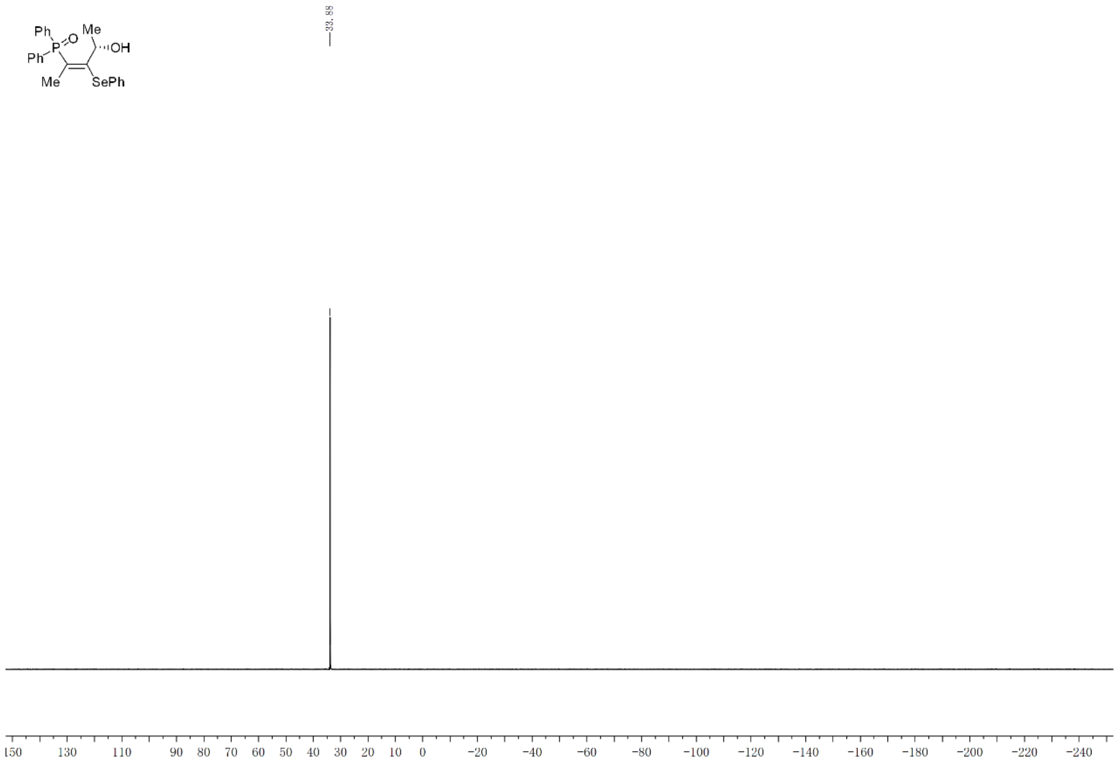

Supplementary Figure 56. ^{1}H NMR, ^{13}C NMR, and ^{31}P NMR spectra of compound **5**

^{1}H NMR of 6

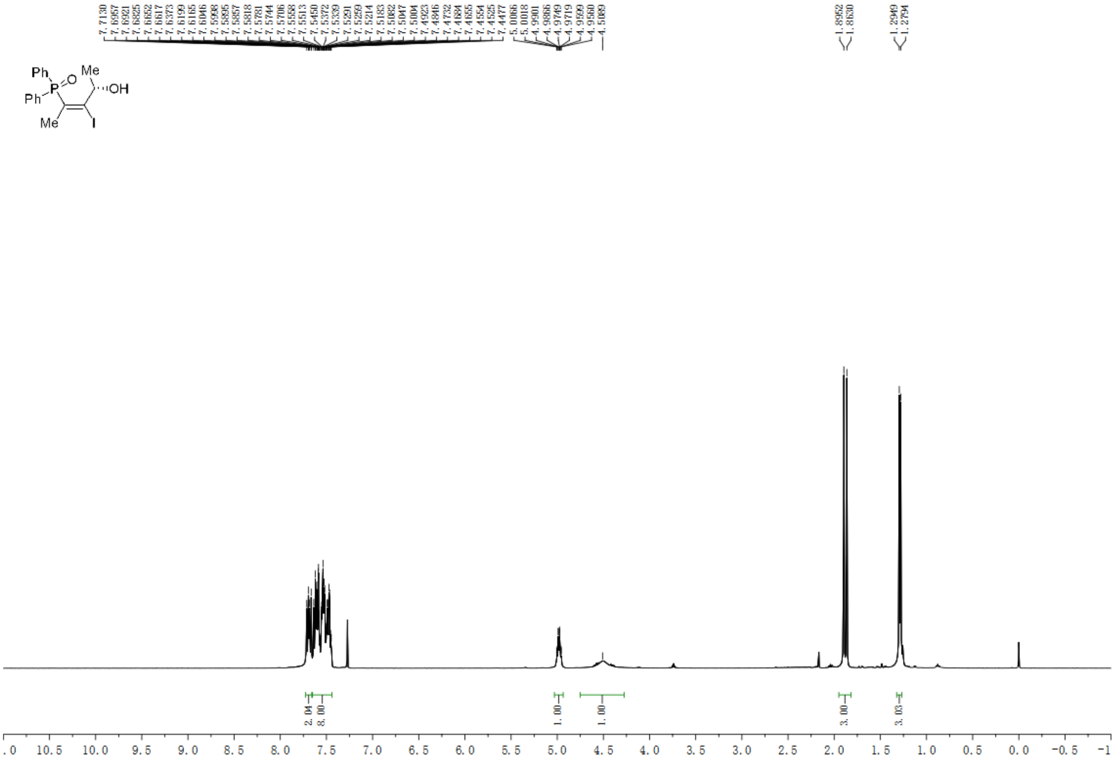

^{13}C NMR of 6

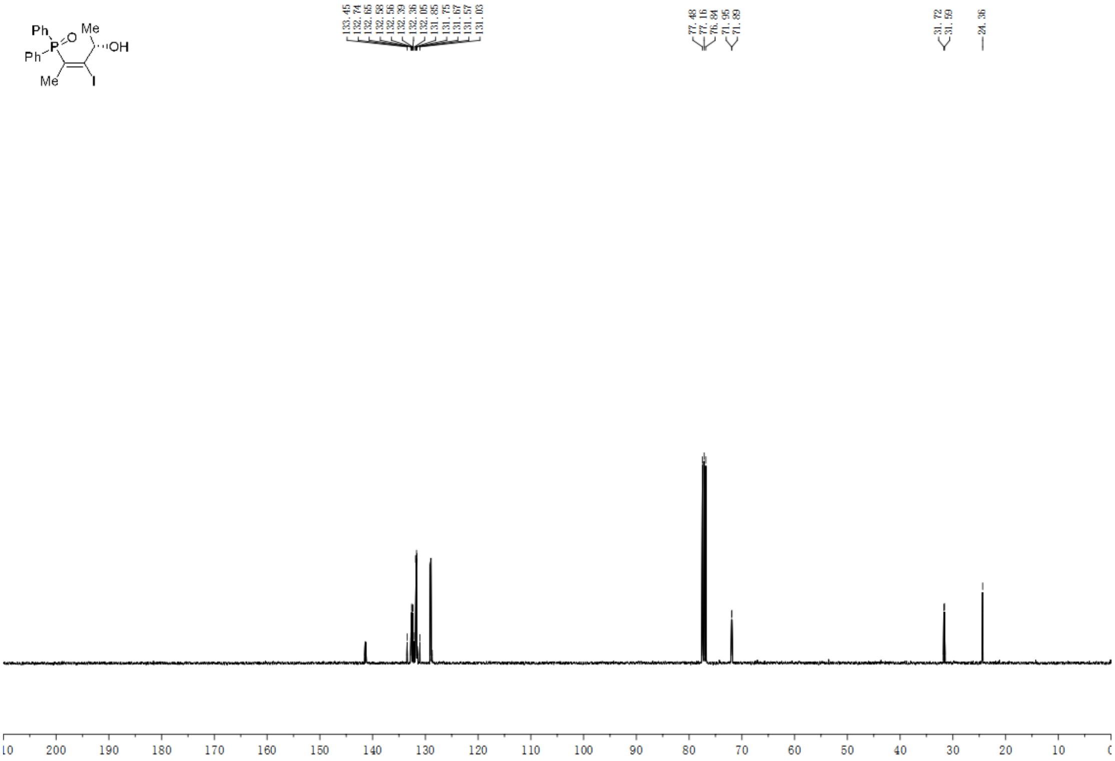

^{31}P NMR of 6

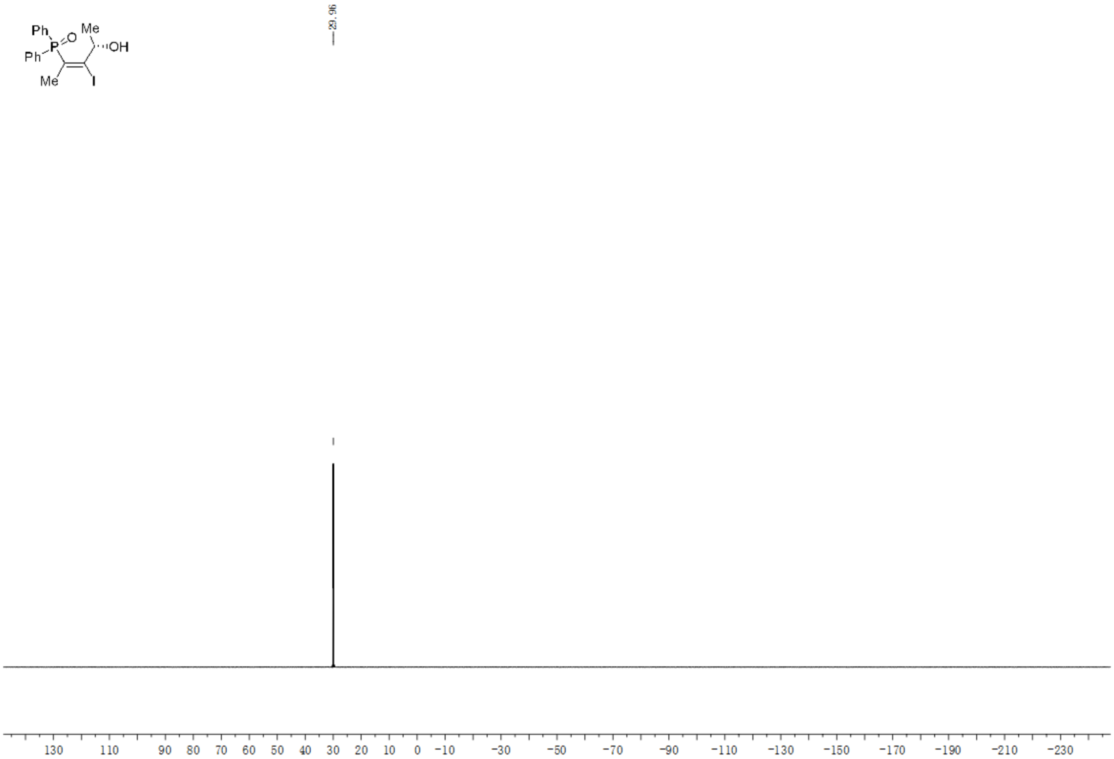

Supplementary Figure 57. ^{1}H NMR, ^{13}C NMR, and ^{31}P NMR spectra of compound **6**

^{1}H NMR of 7

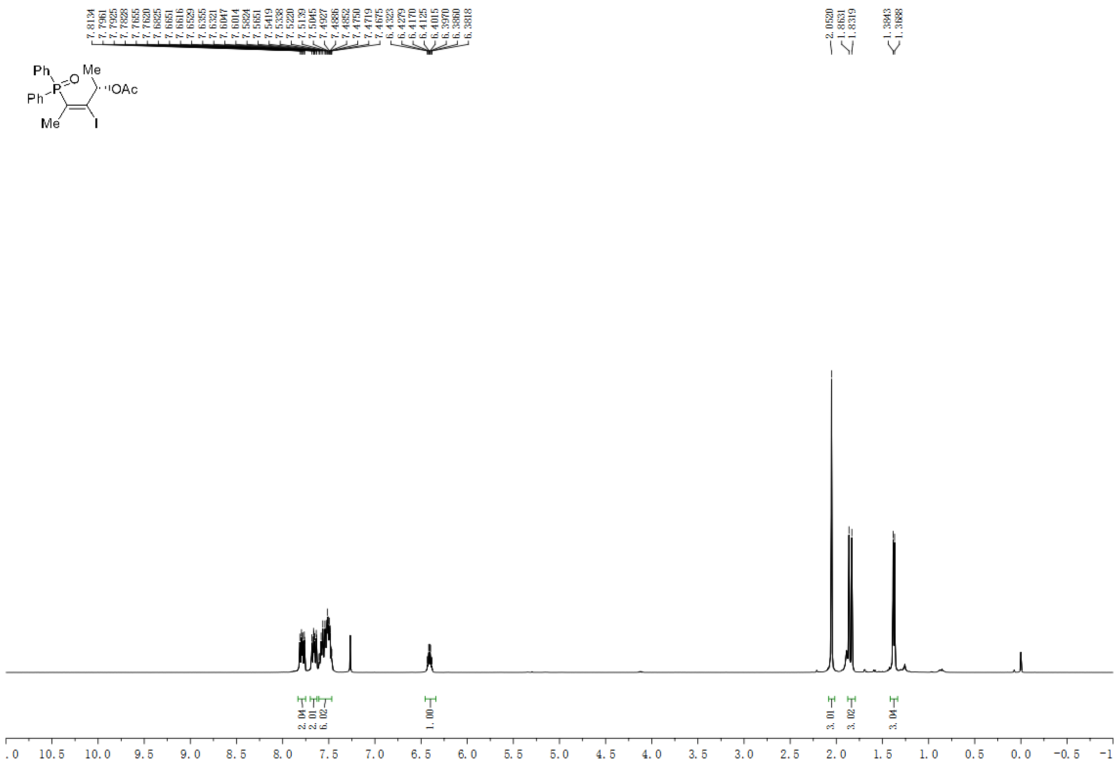

^{13}C NMR of 7

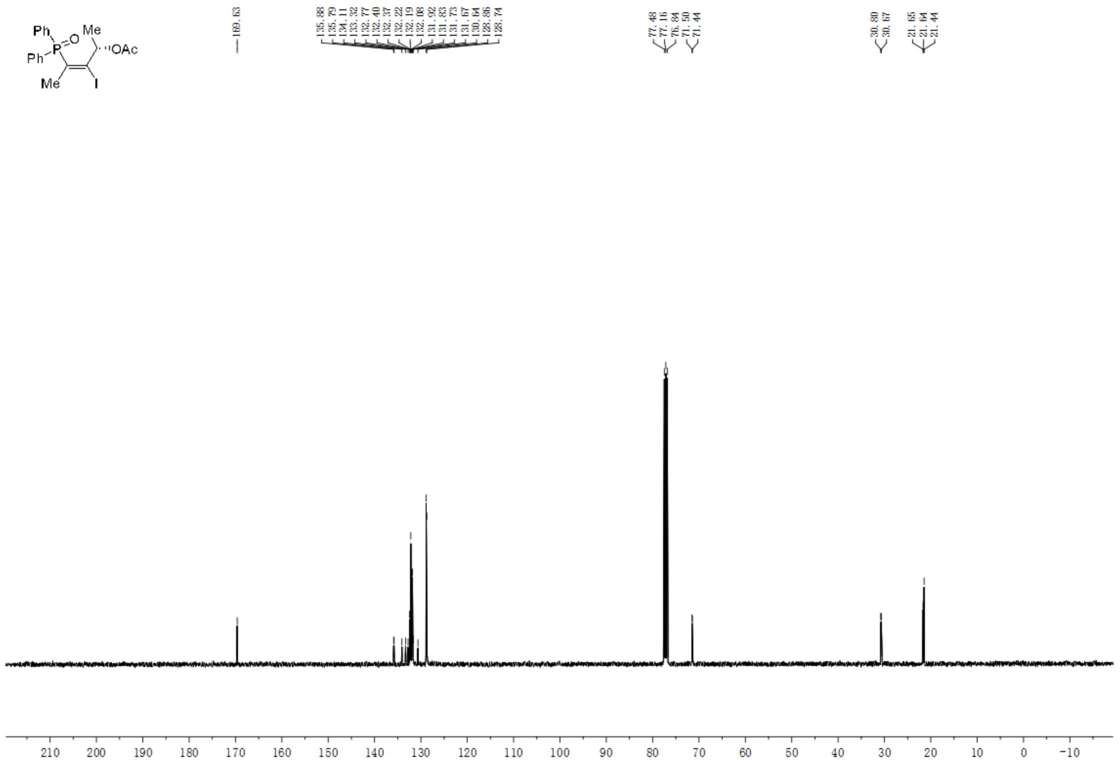

^{31}P NMR of 7

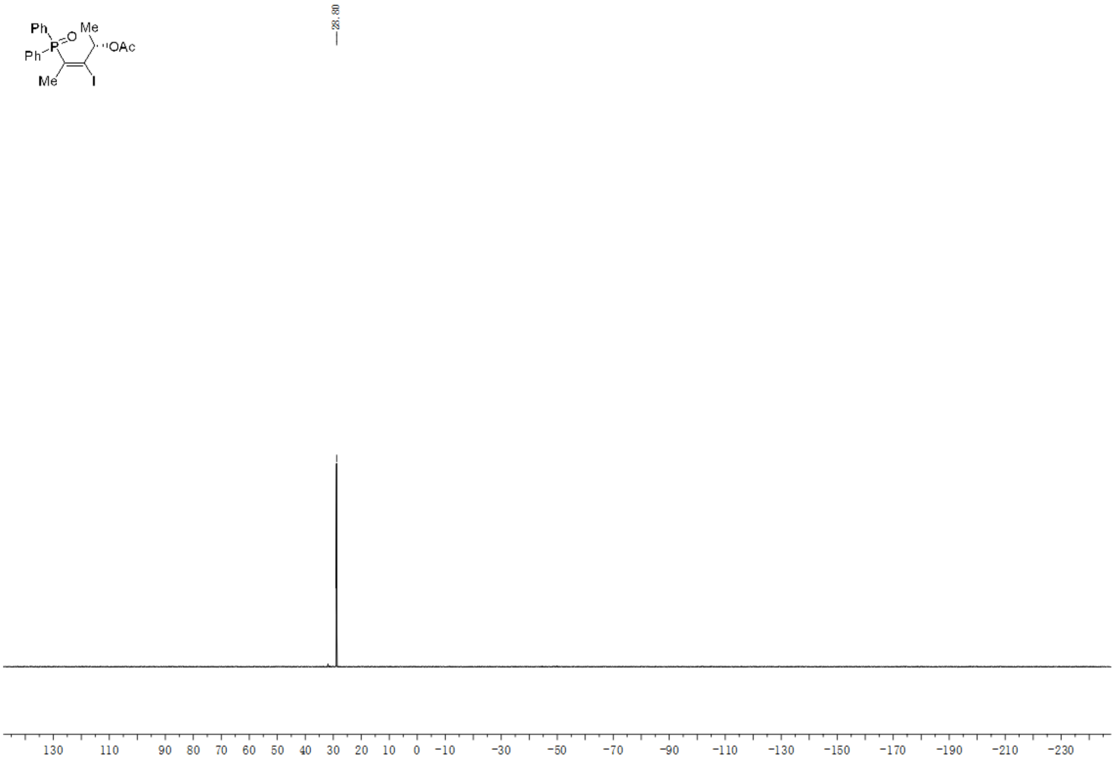

Supplementary Figure 58. ^{1}H NMR, ^{13}C NMR, and ^{31}P NMR spectra of compound **7**

^{1}H NMR of 8

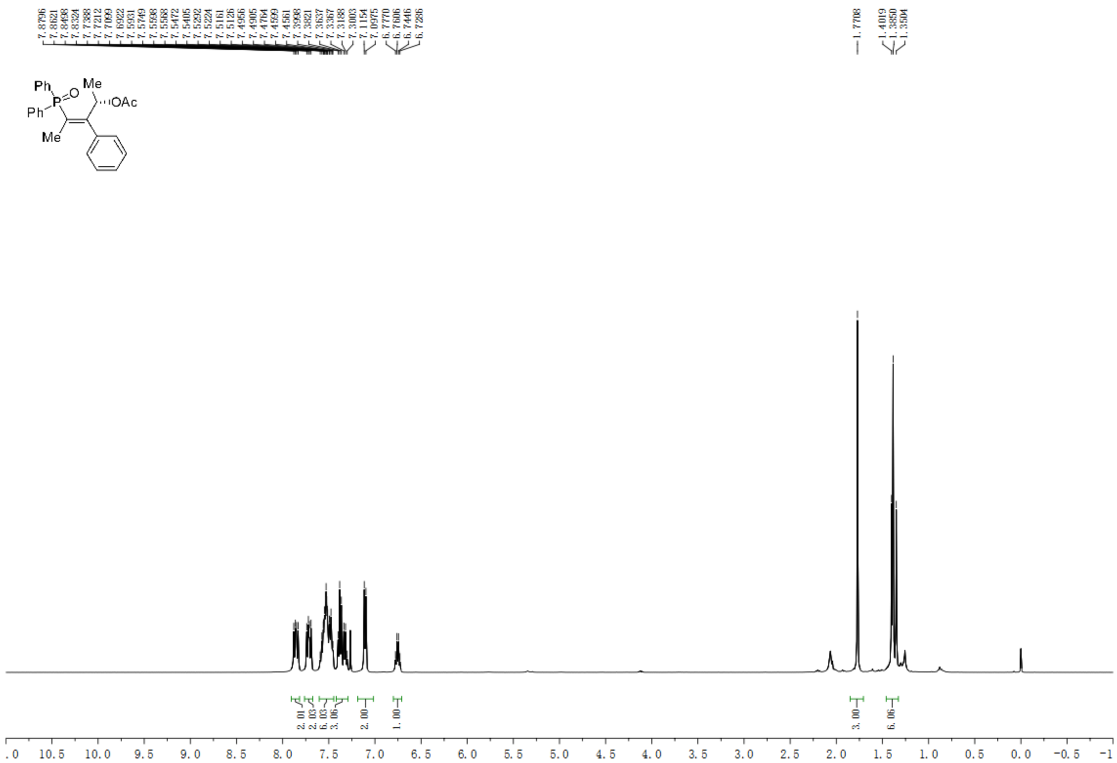

^{13}C NMR of 8

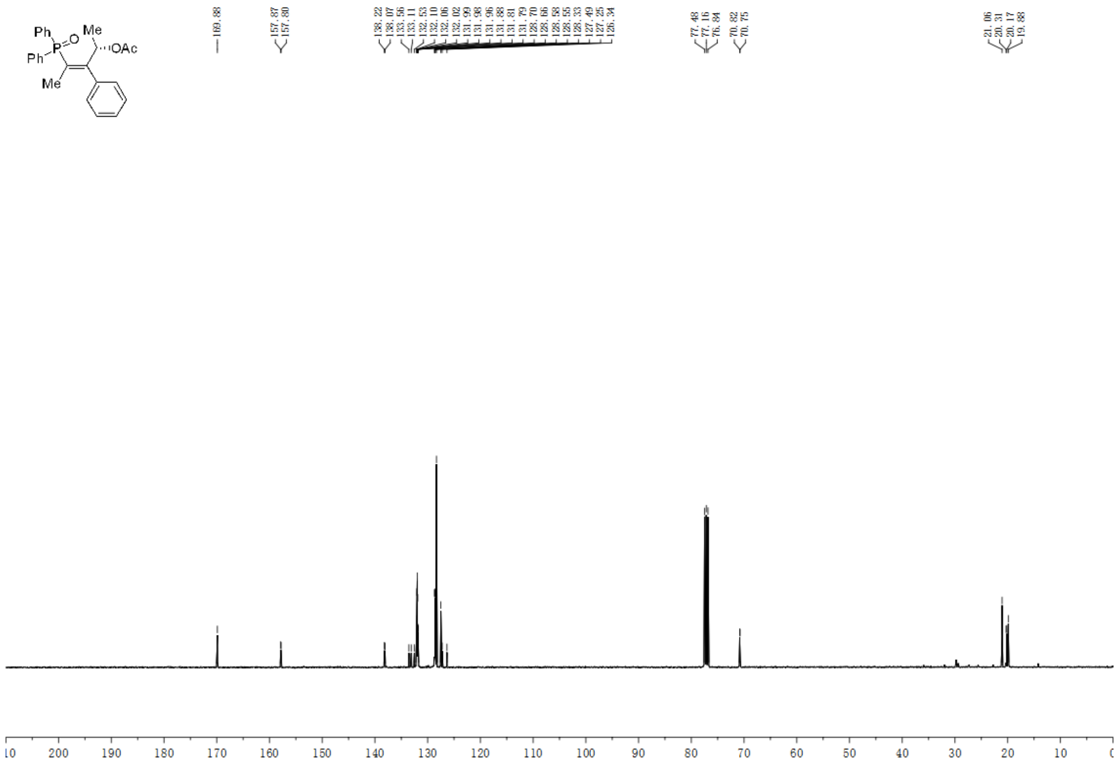

^{31}P NMR of 8

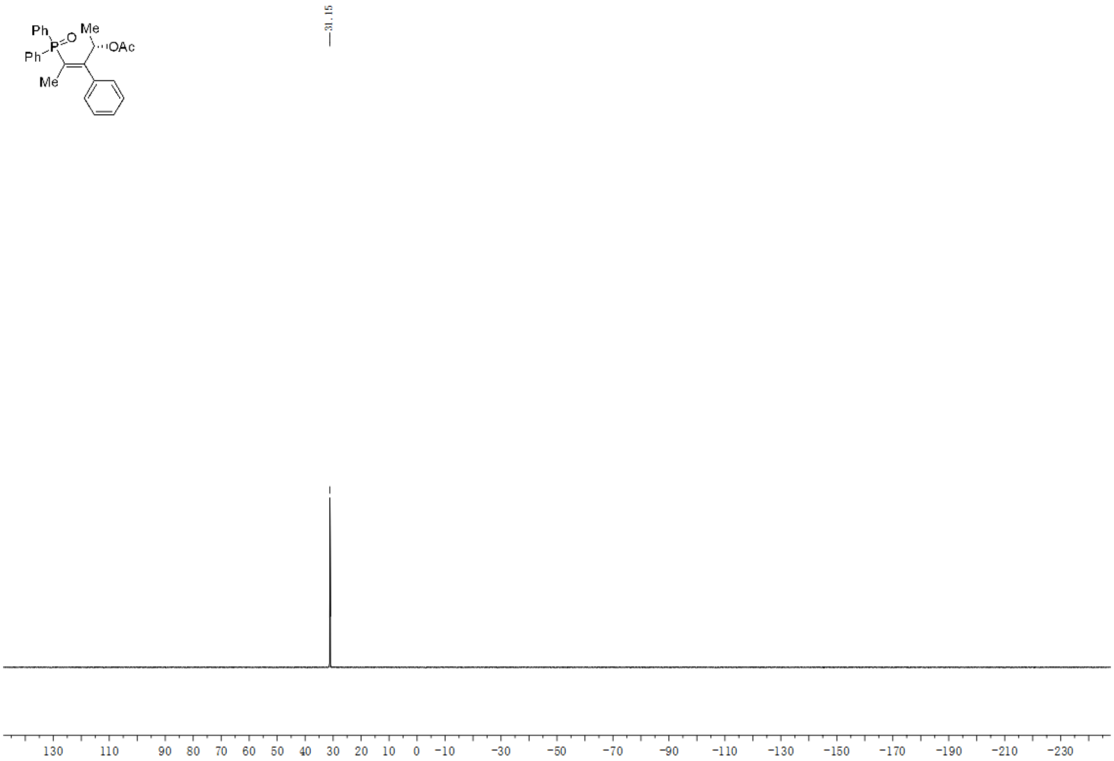

Supplementary Figure 59. ^{1}H NMR, ^{13}C NMR, and ^{31}P NMR spectra of compound **8**

^{1}H NMR of 9

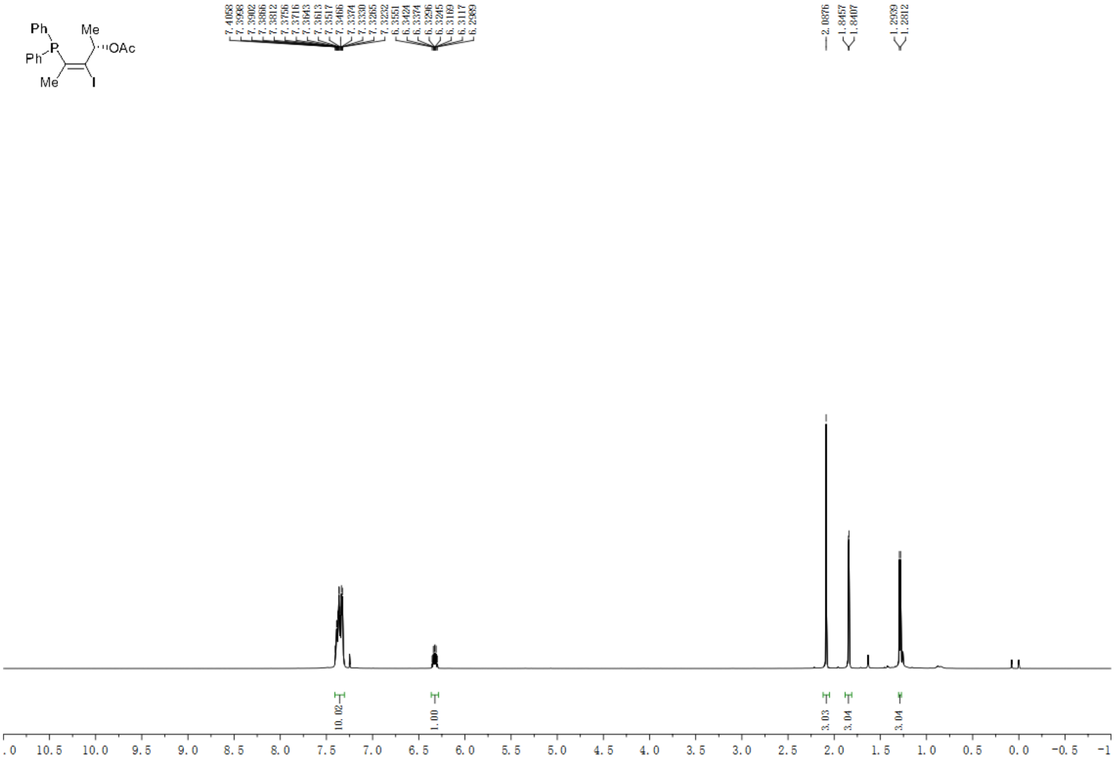

^{13}C NMR of 9

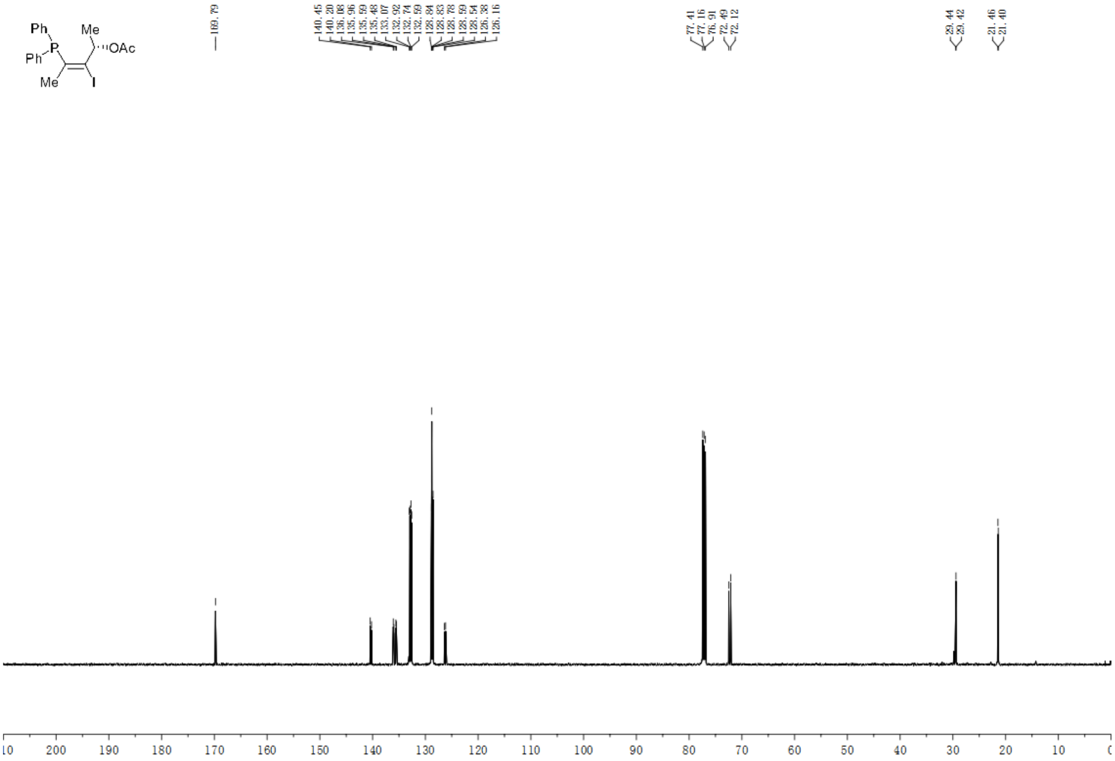

^{31}P NMR of 9

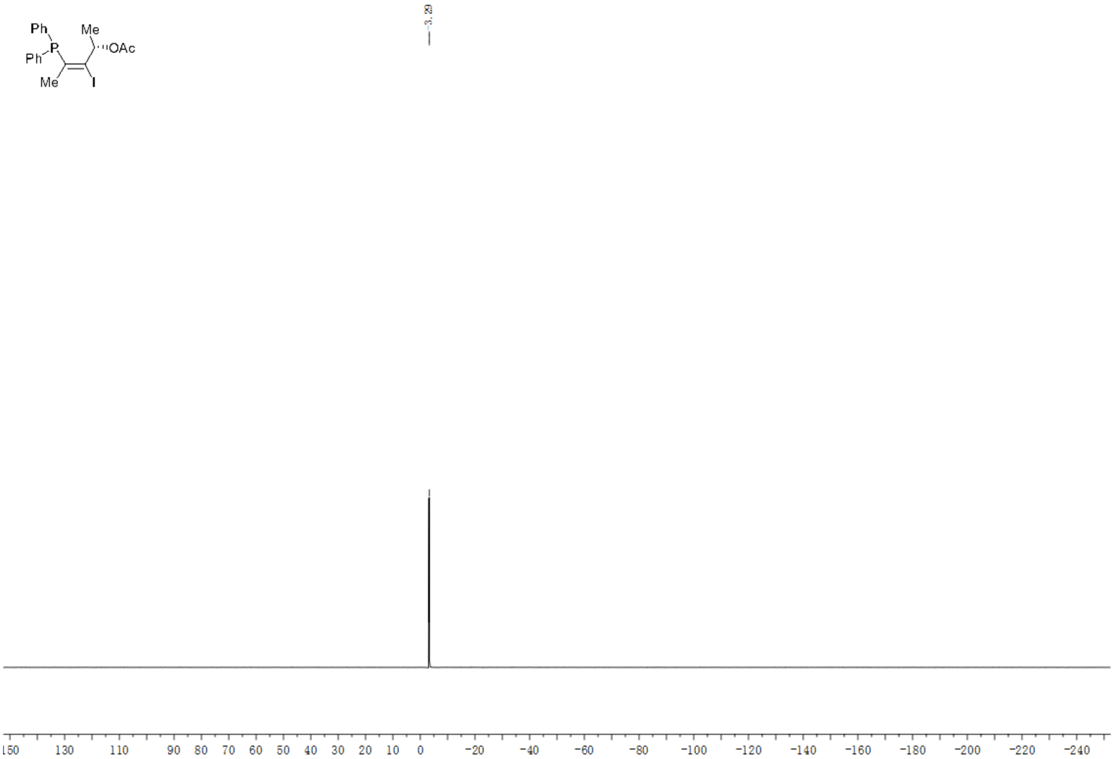

Supplementary Figure 60. ^{1}H NMR, ^{13}C NMR, and ^{31}P NMR spectra of compound **9**

^{1}H NMR of 10

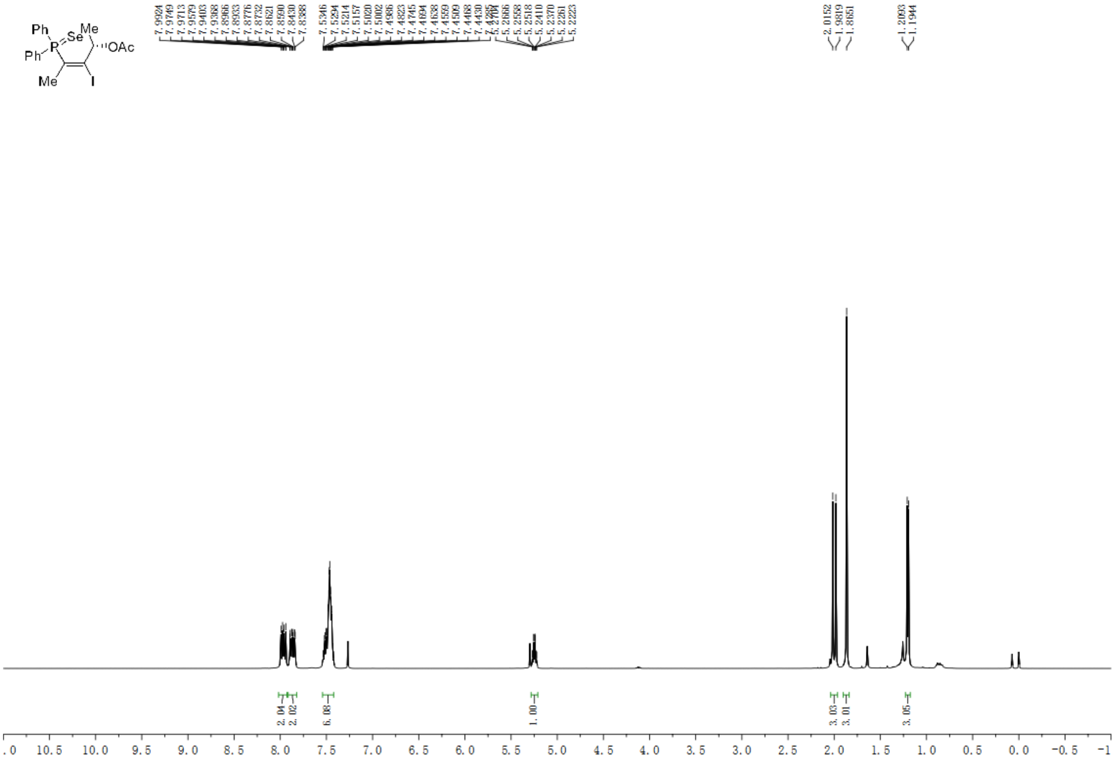

^{13}C NMR of 10

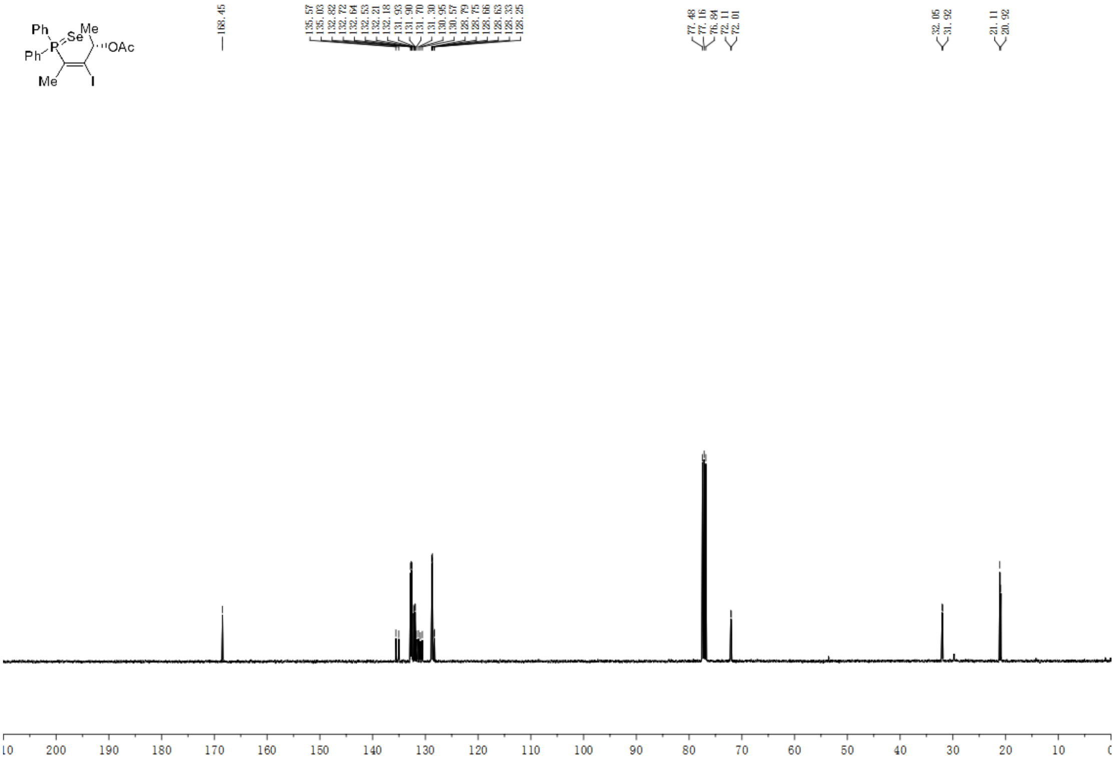

^{31}P NMR of 10

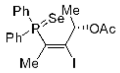

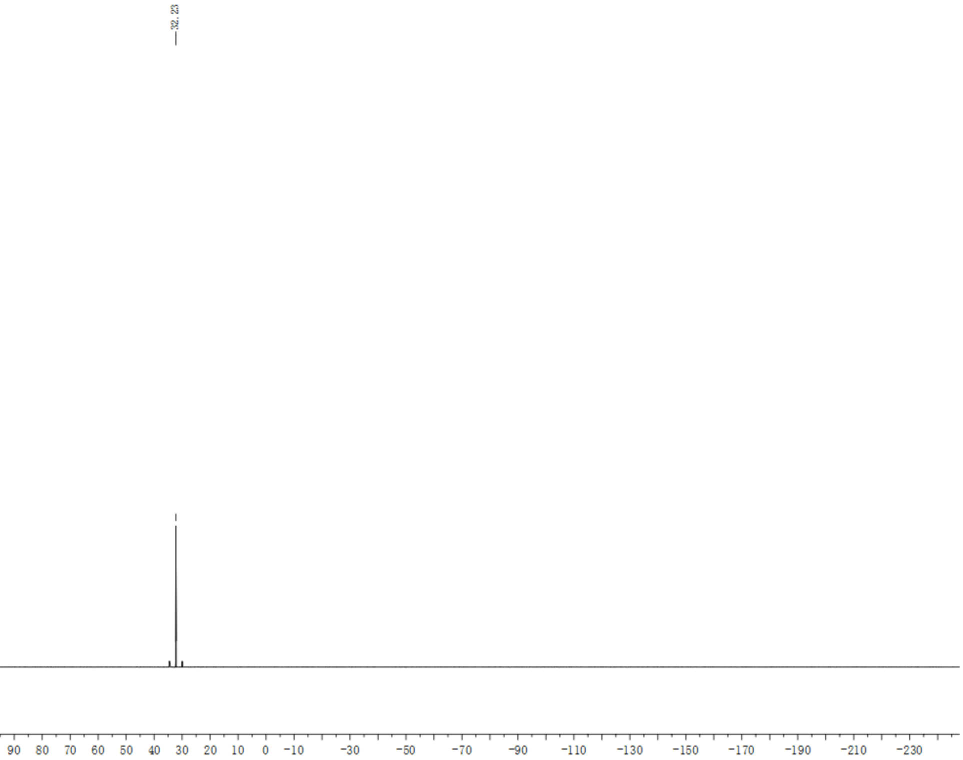

Supplementary Figure 61. ^{1}H NMR, ^{13}C NMR, and ^{31}P NMR spectra of compound **10**

^{1}H NMR of 11

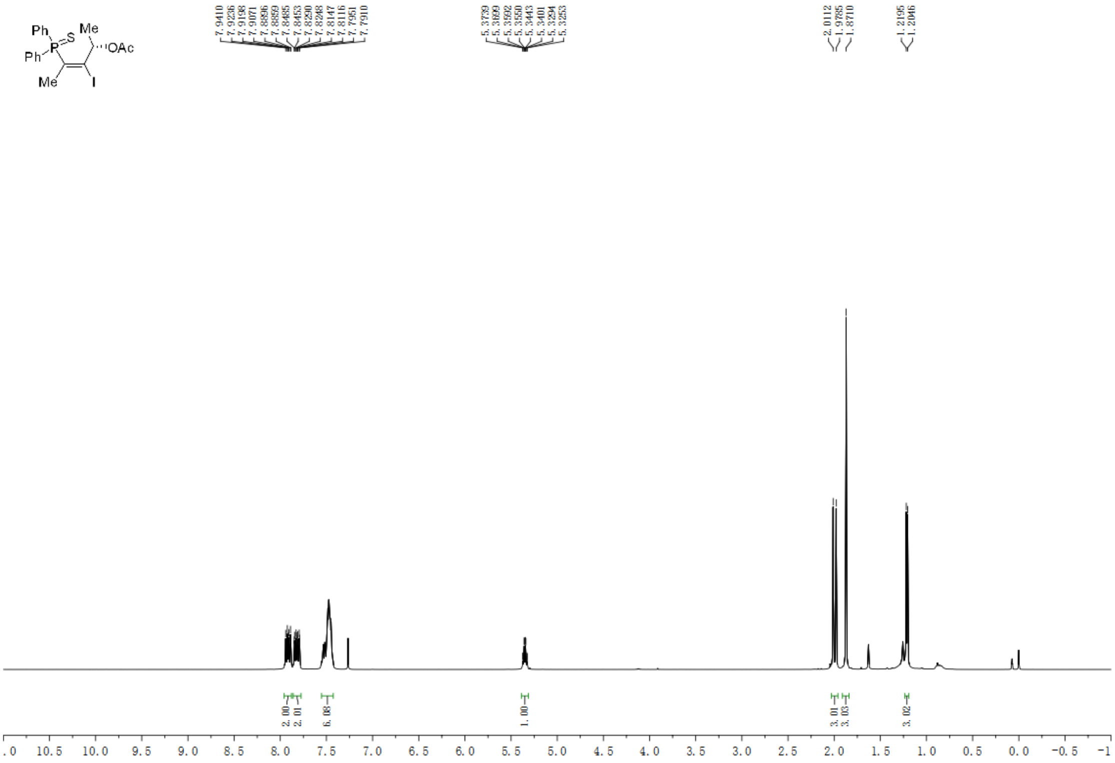

^{13}C NMR of 11

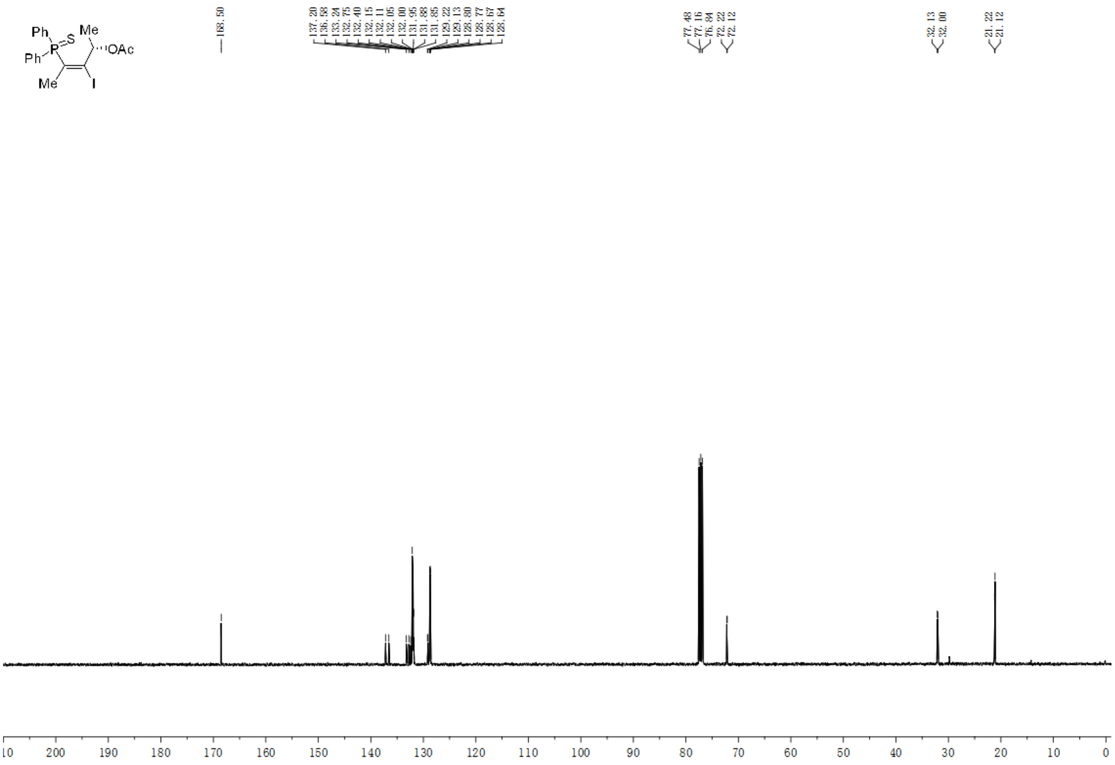

^{31}P NMR of 11

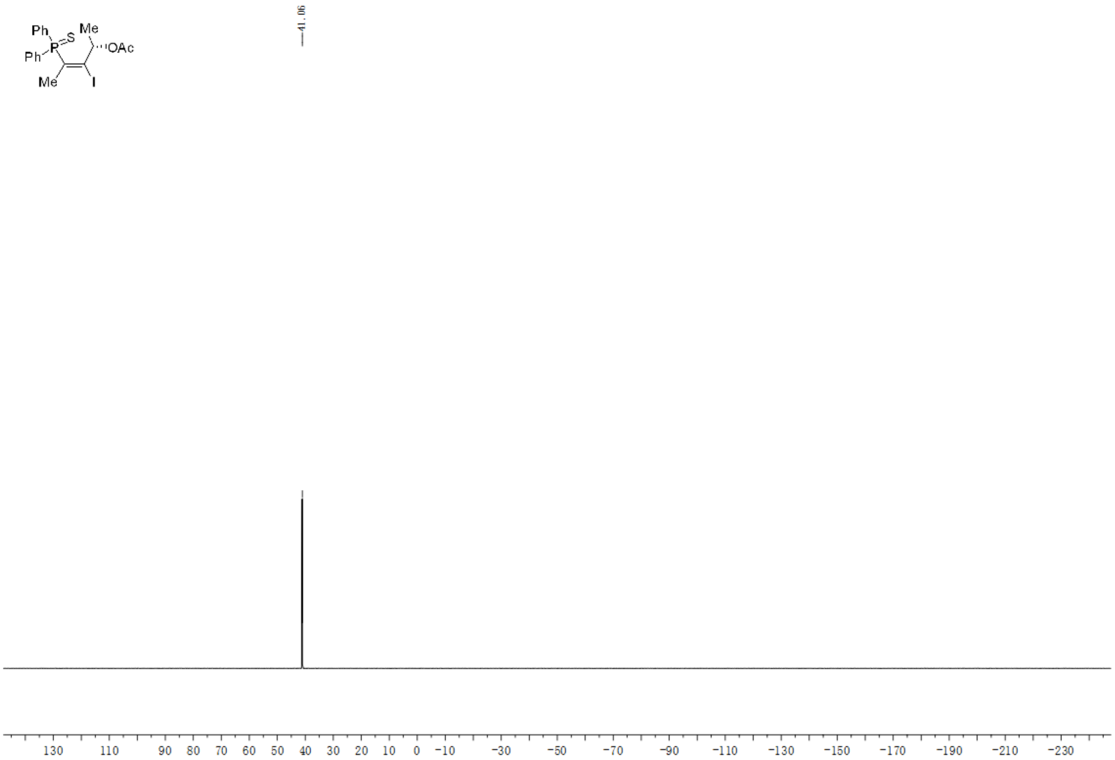

Supplementary Figure 62. ^{1}H NMR, ^{13}C NMR, and ^{31}P NMR spectra of compound **11**

^{1}H NMR of 12

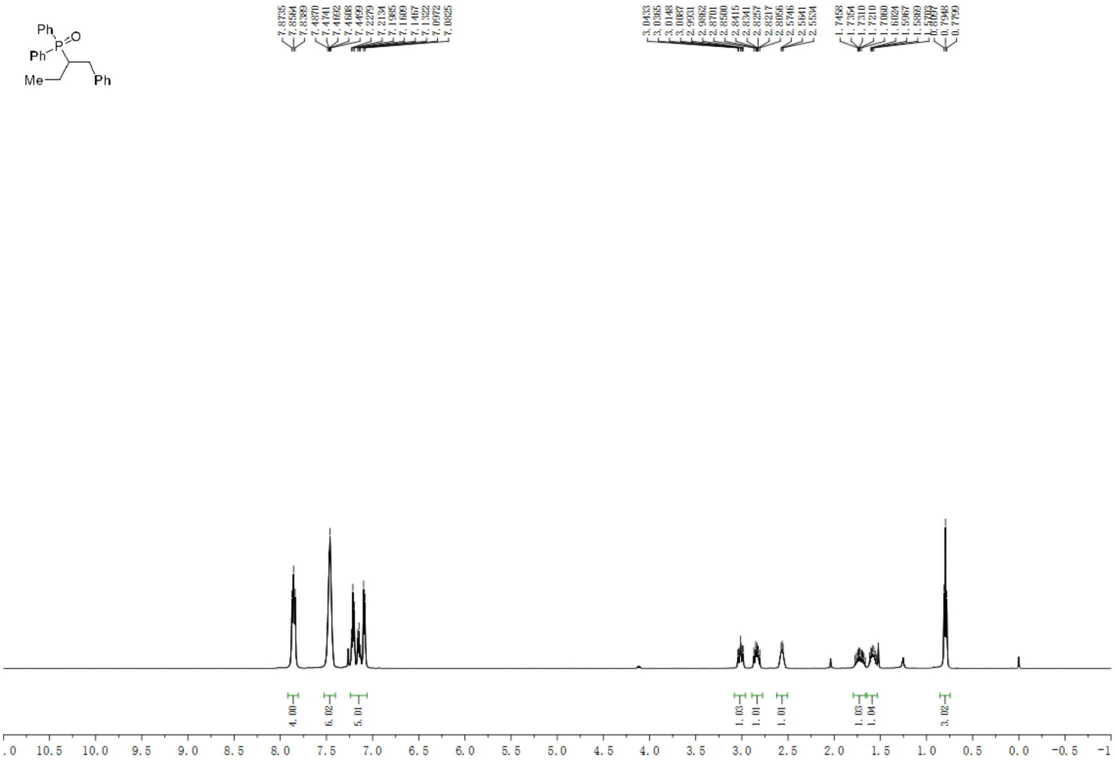

^{13}C NMR of 12

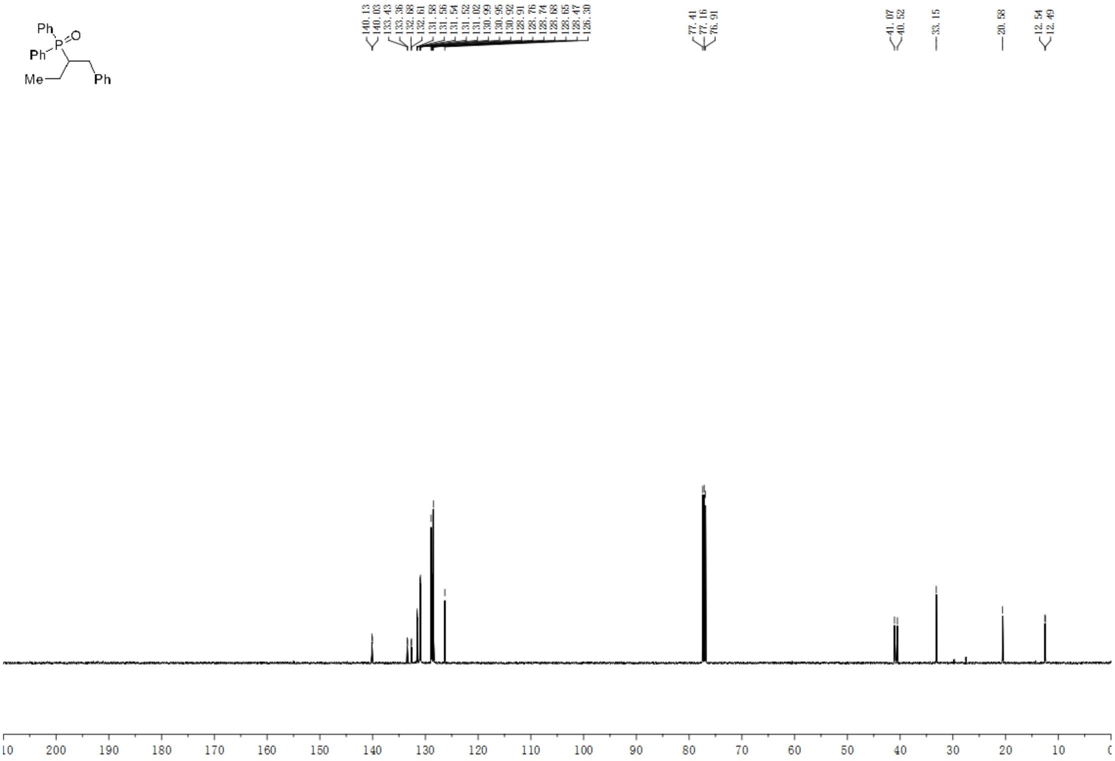

^{31}P NMR of 12

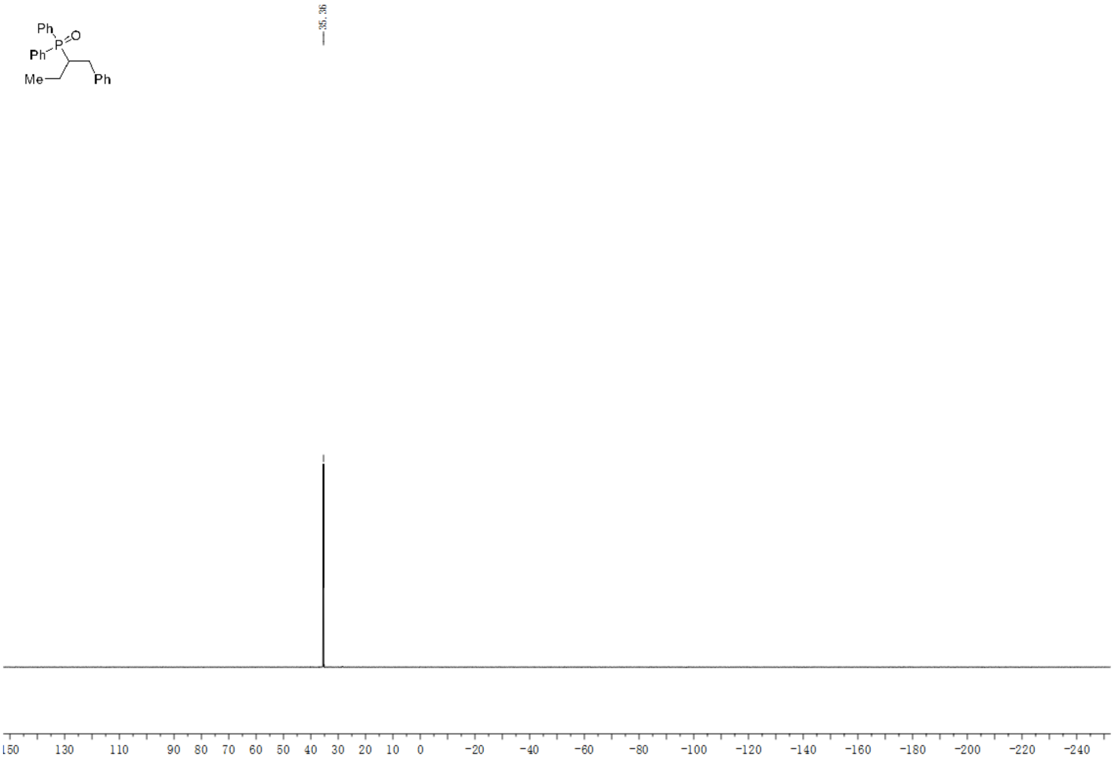

Supplementary Figure 63. ^{1}H NMR, ^{13}C NMR, and ^{31}P NMR spectra of compound **12**

^{1}H NMR of 13

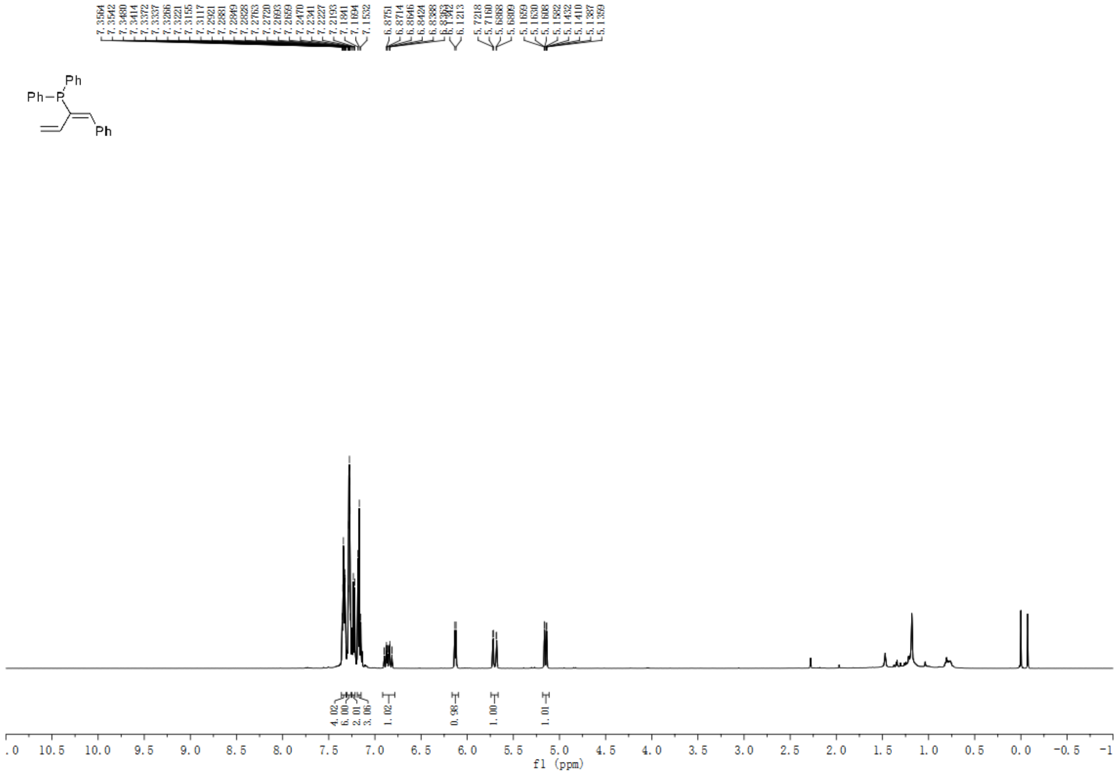

^{13}C NMR of 13

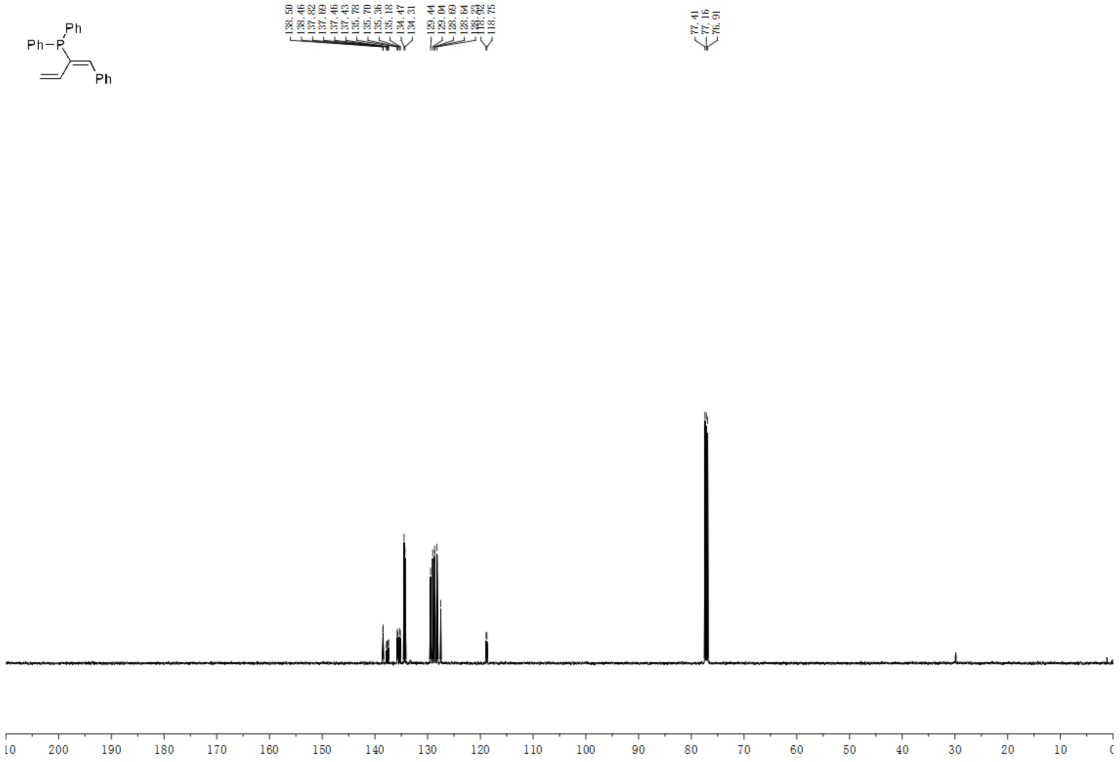

^{31}P NMR of 13

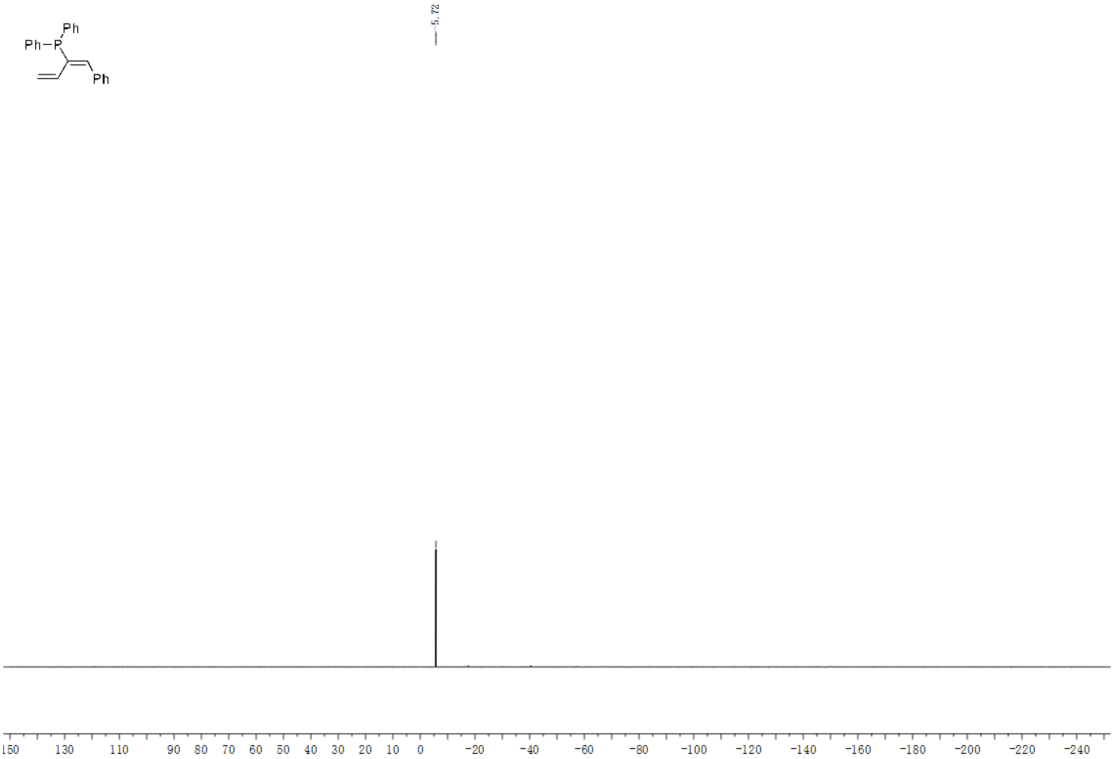

Supplementary Figure 64. ^{1}H NMR, ^{13}C NMR, and ^{31}P NMR spectra of compound **13**

^{1}H NMR of 14

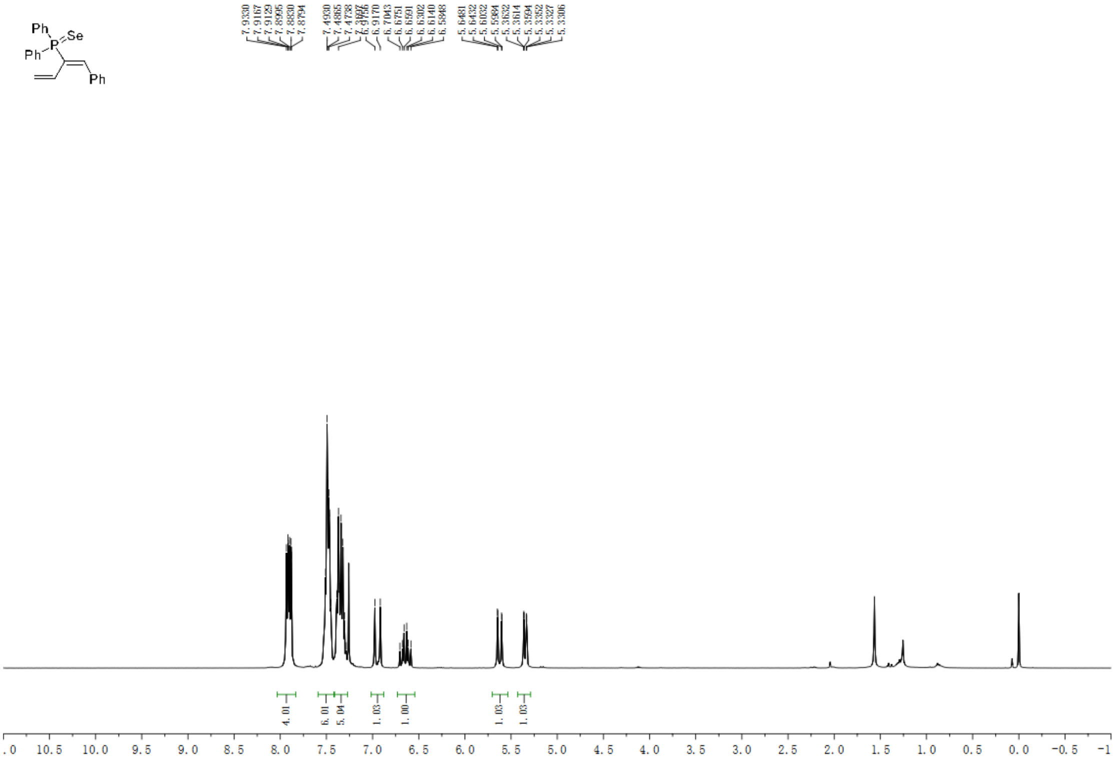

^{13}C NMR of 14

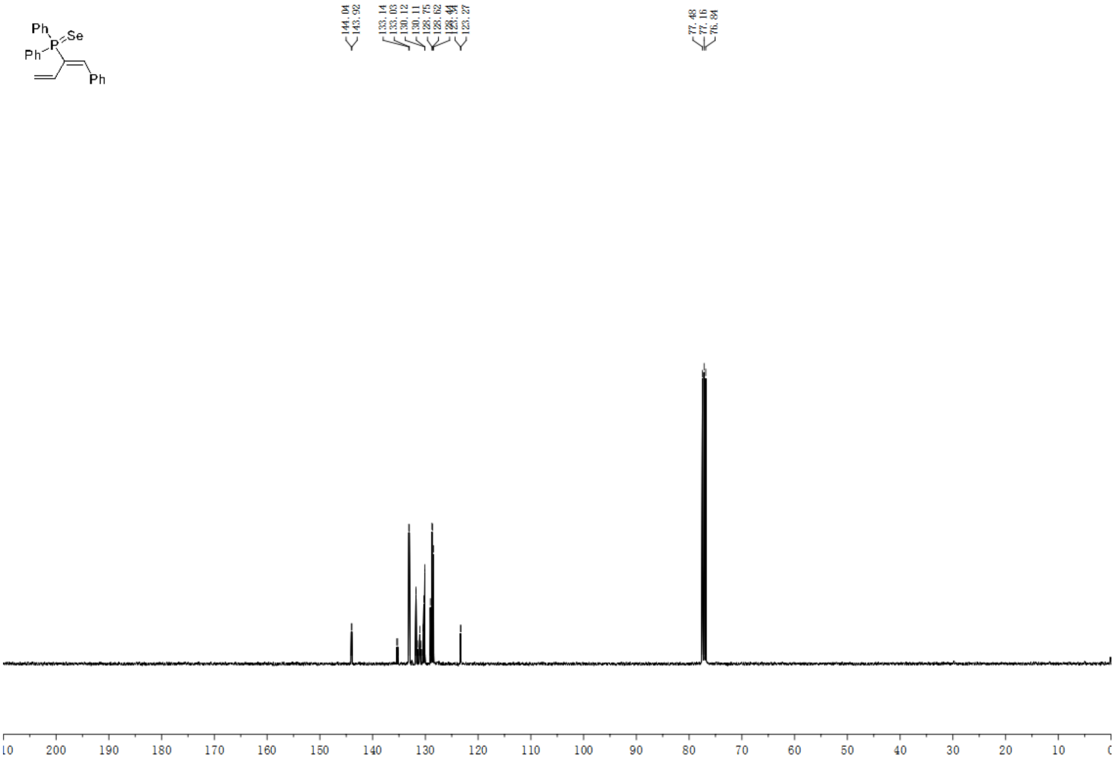

^{31}P NMR of 14

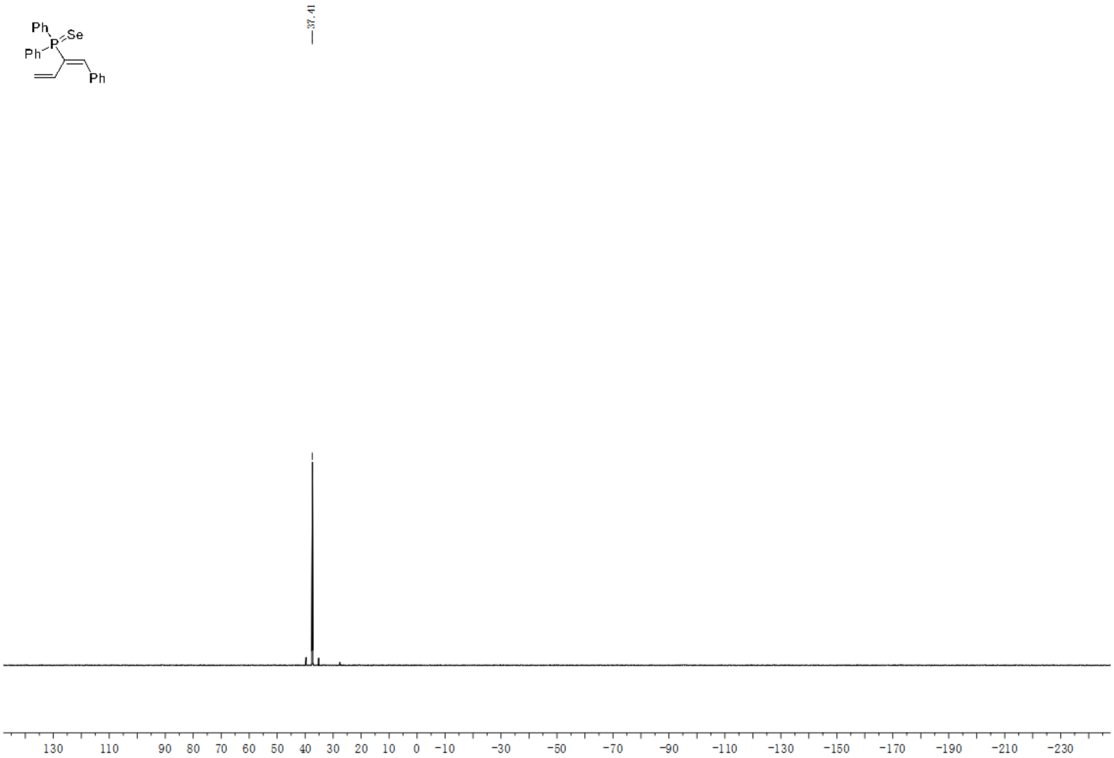

Supplementary Figure 65. ^{1}H NMR, ^{13}C NMR, and ^{31}P NMR spectra of compound **14**

^{1}H NMR of L23

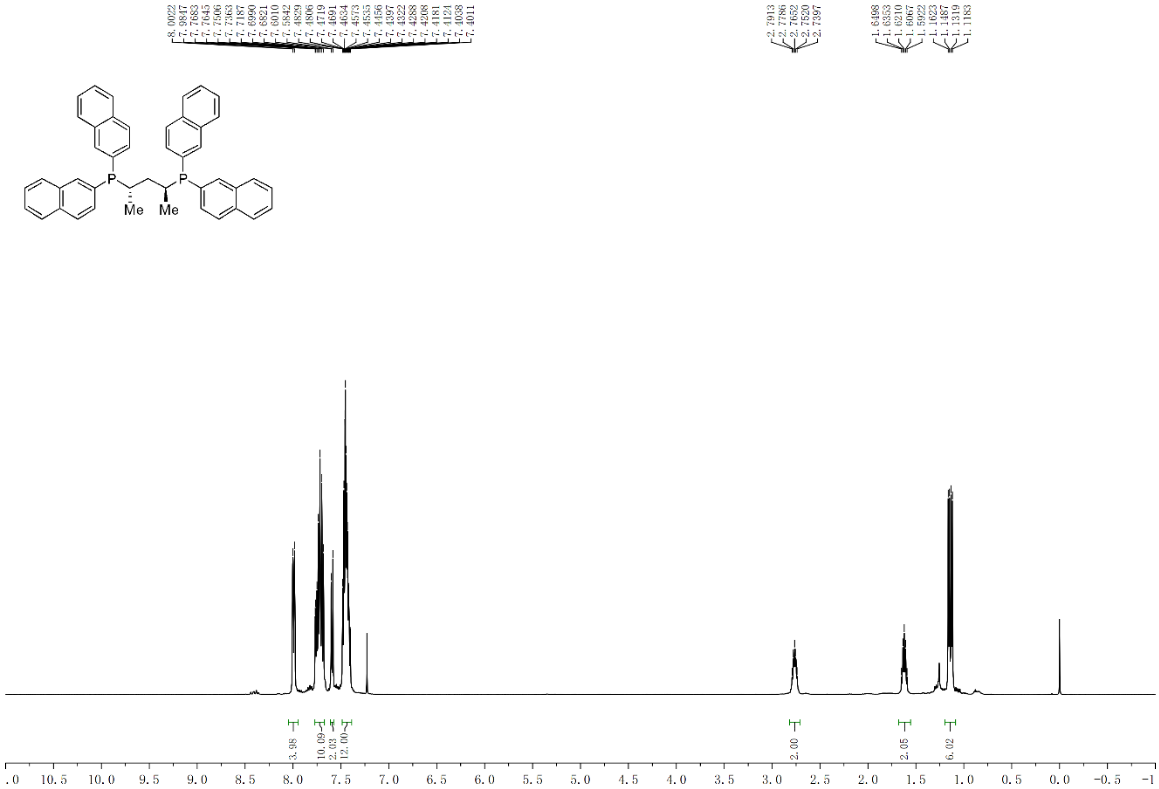

^{13}C NMR of L23

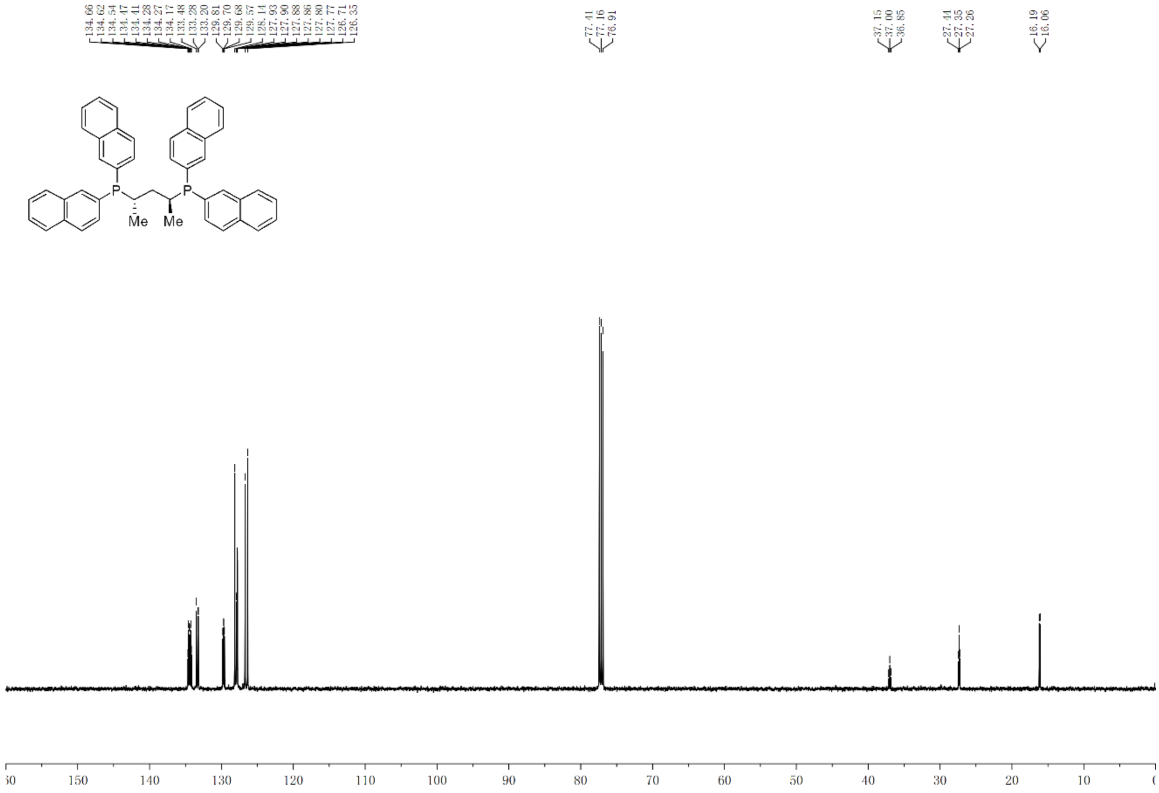

^{31}P NMR of L23

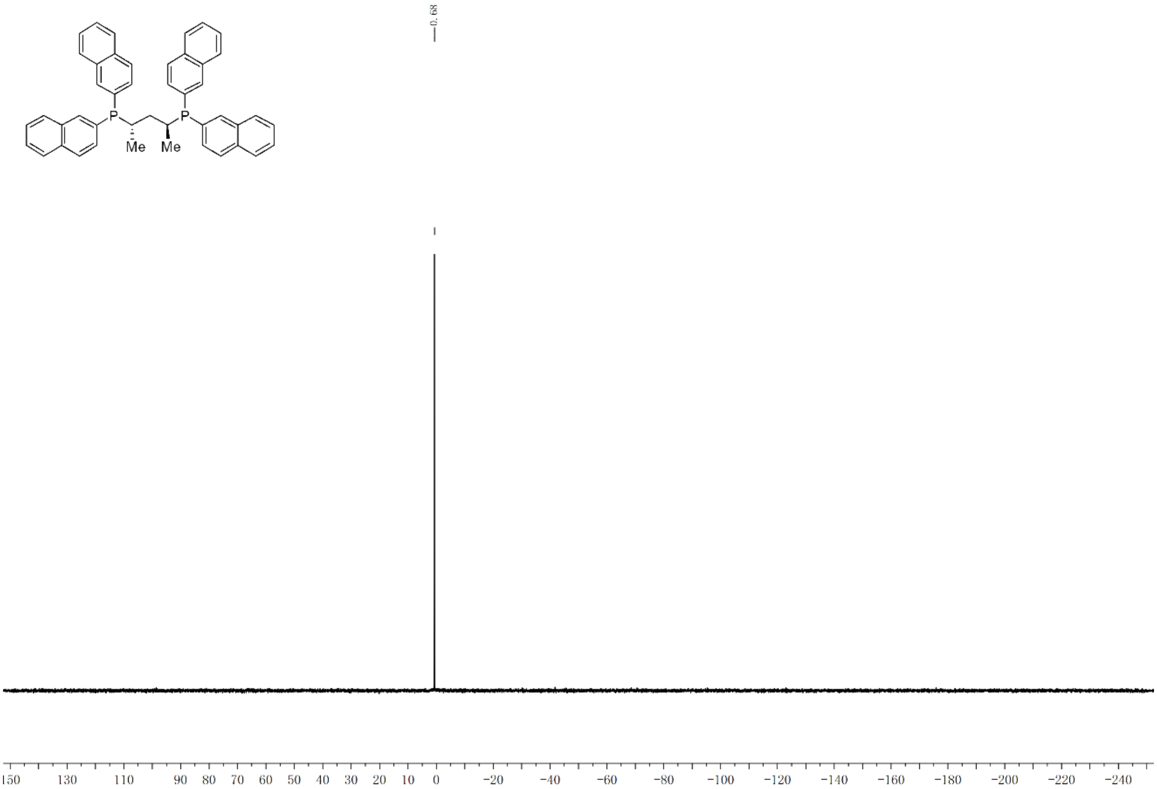

Supplementary Figure 66. ^{1}H NMR, ^{13}C NMR, and ^{31}P NMR spectra of compound **L23**

HPLC traces

Rac-**3a**

SAMPLE INFORMATION

Sample Name:	zjy-5-157-10%-AD-RAc	Acquired By:	System
Sample Type:	Unknown	Sample Set Name:	0
Vial:	53	Acq. Method Set:	10%quanbo
Injection #:	1	Processing Method:	54165463
Injection Volume:	10.00 ul	Channel Name:	220.0nm
Run Time:	18.0 Minutes	Proc. Chnl. Descr.:	2998 PDA 220.0 nm (2998
Date Acquired:	8/29/2021 11:48:57 PM CST		
Date Processed:	8/30/2021 9:43:29 PM CST		

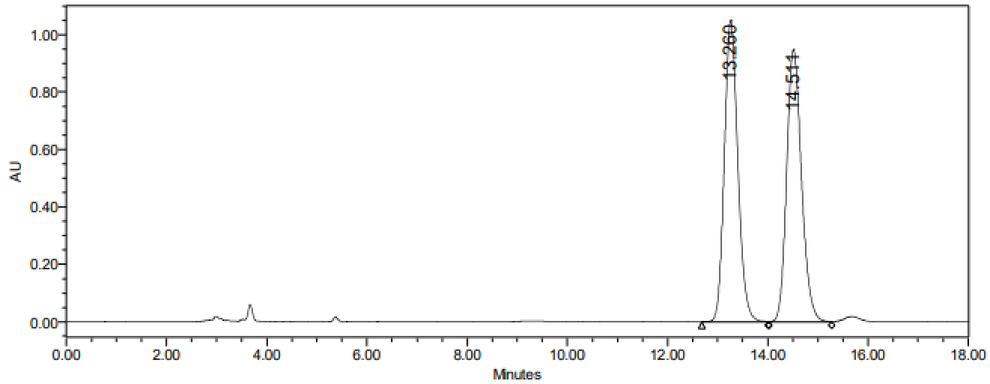

	RT	Area	% Area	Height
1	13.260	19551170	50.12	1049523
2	14.511	19458556	49.88	948084

Asy-**3a**

SAMPLE INFORMATION

Sample Name:	zjy-5-157-10%-AD-asy	Acquired By:	System
Sample Type:	Unknown	Sample Set Name:	0
Vial:	54	Acq. Method Set:	10%quanbo
Injection #:	1	Processing Method:	54165463
Injection Volume:	10.00 ul	Channel Name:	220.0nm
Run Time:	18.0 Minutes	Proc. Chnl. Descr.:	2998 PDA 220.0 nm (2998
Date Acquired:	8/30/2021 12:07:37 AM CST		
Date Processed:	8/30/2021 9:44:40 PM CST		

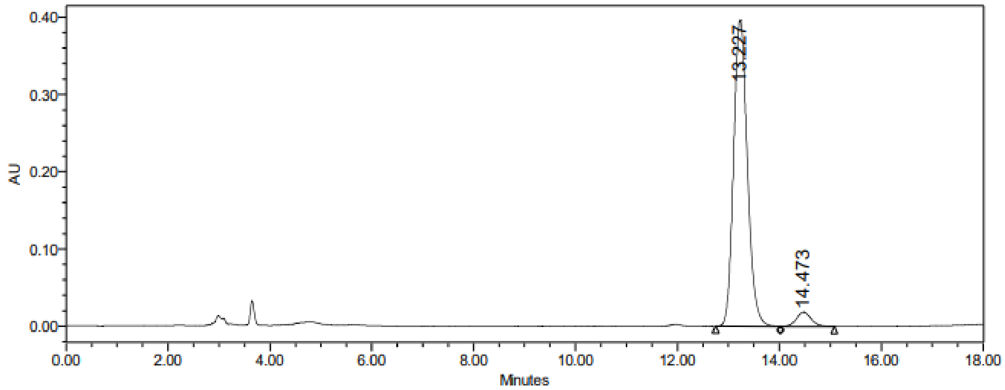

	RT	Area	% Area	Height
1	13.227	7308644	95.05	395890
2	14.473	380915	4.95	18865

Supplementary Figure 67. HPLC spectra of compound **3a**

Rac-**3b**

SAMPLE INFORMATION

Sample Name:	zjy-5-169-20%-IC-RAC	Acquired By:	System
Sample Type:	Unknown	Sample Set Name:	0
Vial:	49	Acq. Method Set:	20% quanbo
Injection #:	1	Processing Method	zjy 5 169 RAC
Injection Volume:	10.00 ul	Channel Name:	220.0nm
Run Time:	25.0 Minutes	Proc. Chnl. Descr.:	2998 PDA 220.0 nm (2998
Date Acquired:	8/29/2021 8:57:10 PM CST		
Date Processed:	8/30/2021 9:23:36 PM CST		

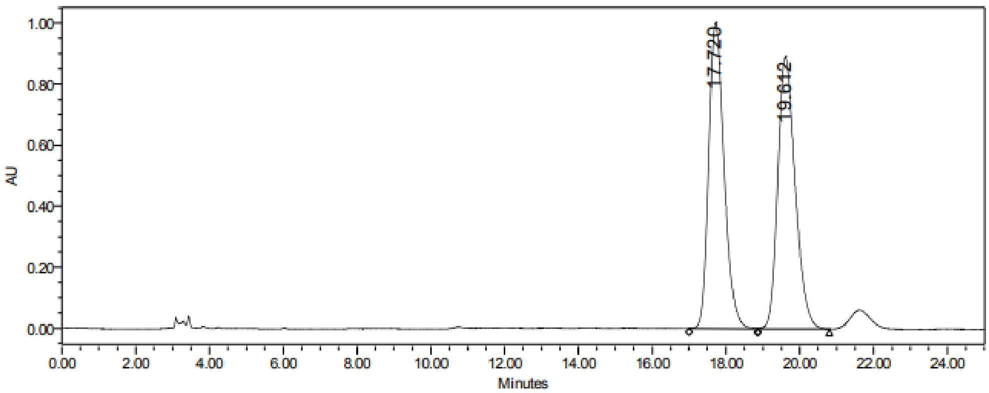

	RT	Area	% Area	Height
1	17.720	29083452	50.01	1001871
2	19.612	29075269	49.99	892503

Asy-**3b**

SAMPLE INFORMATION

Sample Name:	zjy-5-169-20%-IC-asy	Acquired By:	System
Sample Type:	Unknown	Sample Set Name:	0
Vial:	50	Acq. Method Set:	20% quanbo
Injection #:	1	Processing Method	zjy 5 169 asy
Injection Volume:	10.00 ul	Channel Name:	220.0nm
Run Time:	25.0 Minutes	Proc. Chnl. Descr.:	2998 PDA 220.0 nm (2998
Date Acquired:	8/29/2021 9:22:52 PM CST		
Date Processed:	8/30/2021 9:26:41 PM CST		

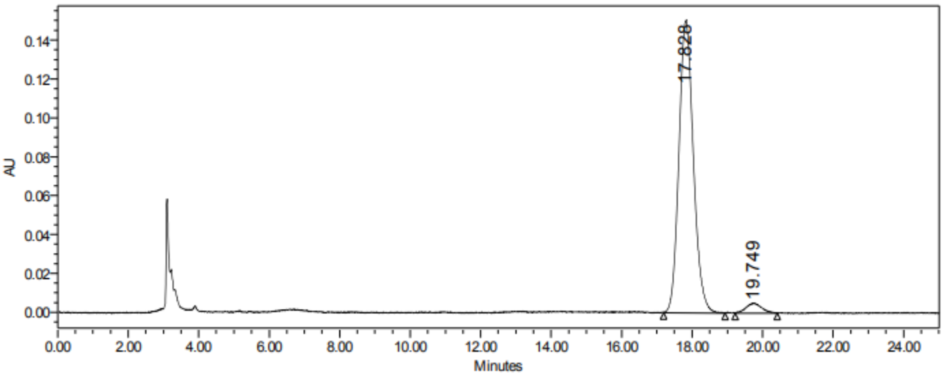

	RT	Area	% Area	Height
1	17.828	4312354	96.40	150381
2	19.749	161042	3.60	5180

Supplementary Figure 68. HPLC spectra of compound **3b**

Rac-**3c**

SAMPLE INFORMATION

Sample Name:	zjy-5-170-20%-IC-RAC	Acquired By:	System
Sample Type:	Unknown	Sample Set Name:	0
Vial:	51	Acq. Method Set:	20% quanbo
Injection #:	1	Processing Method	41654
Injection Volume:	10.00 ul	Channel Name:	220.0nm
Run Time:	20.0 Minutes	Proc. Chnl. Descr.:	2998 PDA 220.0 nm (2998
Date Acquired:	8/29/2021 9:48:35 PM CST		
Date Processed:	8/30/2021 9:28:28 PM CST		

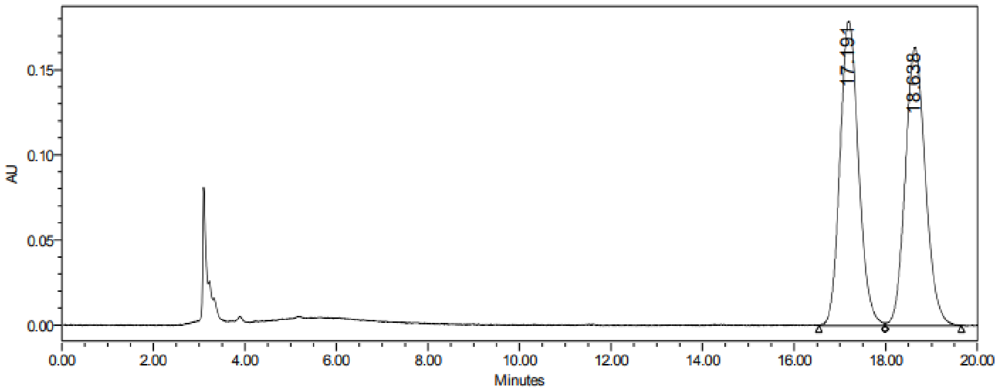

	RT	Area	% Area	Height
1	17.191	5010699	50.03	178788
2	18.638	5004583	49.97	163498

Asy-**3c**

SAMPLE INFORMATION

Sample Name:	zjy-5-170-20%-IC-asy	Acquired By:	System
Sample Type:	Unknown	Sample Set Name:	0
Vial:	52	Acq. Method Set:	20% quanbo
Injection #:	1	Processing Method	44564
Injection Volume:	10.00 ul	Channel Name:	220.0nm
Run Time:	20.0 Minutes	Proc. Chnl. Descr.:	2998 PDA 220.0 nm (2998
Date Acquired:	8/29/2021 10:09:17 PM CST		
Date Processed:	8/30/2021 9:30:19 PM CST		

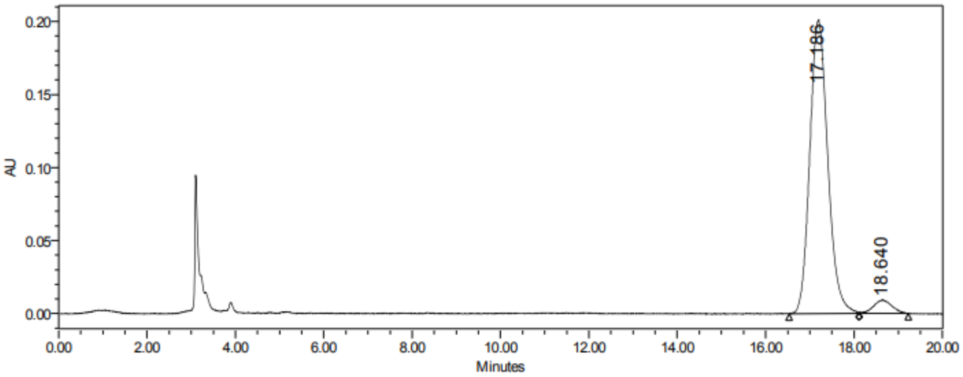

	RT	Area	% Area	Height
1	17.186	5645673	95.47	200835
2	18.640	267795	4.53	9252

Supplementary Figure 69. HPLC spectra of compound **3c**

Rac-**3d**

SAMPLE INFORMATION

Sample Name:	zjy-5-172-20%-IC-RAC	Acquired By:	System
Sample Type:	Unknown	Sample Set Name:	0
Vial:	54	Acq. Method Set:	20%quanbo
Injection #:	1	Processing Method:	ZJY 5 172
Injection Volume:	10.00 ul	Channel Name:	220.0nm
Run Time:	20.0 Minutes	Proc. Chnl. Descr.:	2998 PDA 220.0 nm (2998
Date Acquired:	7/21/2021 3:18:54 PM CST		
Date Processed:	7/21/2021 4:24:35 PM CST		

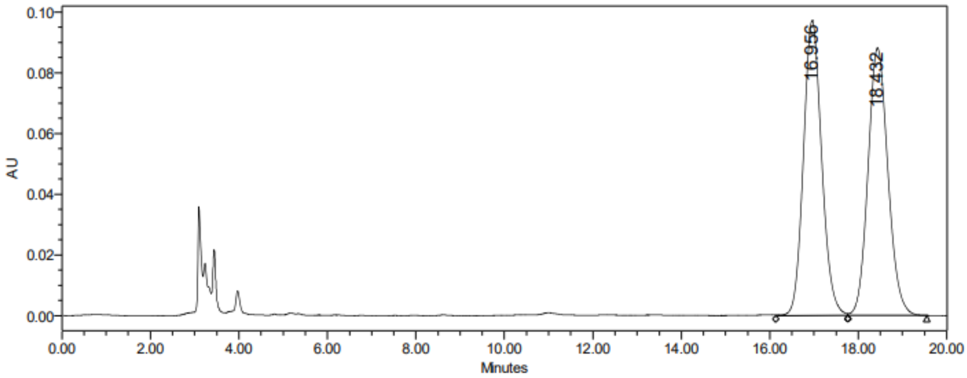

	RT	Area	% Area	Height
1	16.956	2799450	50.23	97212
2	18.432	2773913	49.77	88183

Asy-**3d**

SAMPLE INFORMATION

Sample Name:	zjy-5-172-20%-IC-asy	Acquired By:	System
Sample Type:	Unknown	Sample Set Name:	0
Vial:	52	Acq. Method Set:	20%quanbo
Injection #:	1	Processing Method:	ZJY 5 172
Injection Volume:	10.00 ul	Channel Name:	220.0nm
Run Time:	20.0 Minutes	Proc. Chnl. Descr.:	2998 PDA 220.0 nm (2998
Date Acquired:	7/21/2021 2:37:16 PM CST		
Date Processed:	7/21/2021 4:23:25 PM CST		

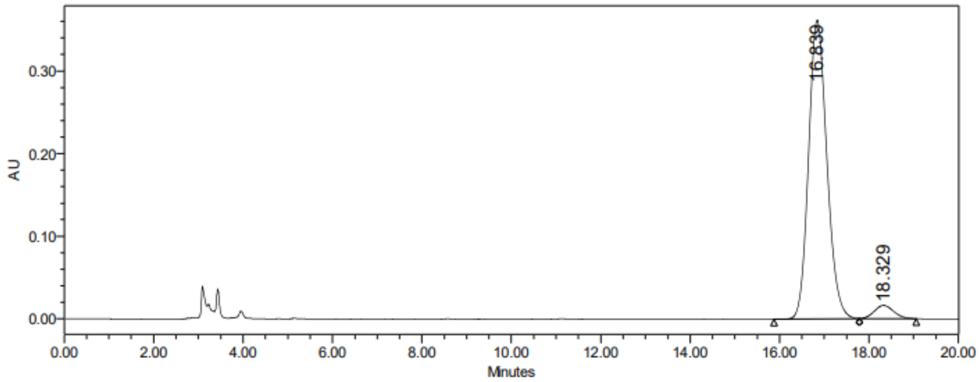

	RT	Area	% Area	Height
1	16.839	10338389	95.38	361308
2	18.329	500410	4.62	16243

Supplementary Figure 70. HPLC spectra of compound **3d**

Rac-**3e**

SAMPLE INFORMATION

Sample Name:	zjy-5-181-20%-IC-rac	Acquired By:	System
Sample Type:	Unknown	Sample Set Name:	0
Vial:	69	Acq. Method Set:	20% quanbo
Injection #:	1	Processing Method	zjy 5 181 rac
Injection Volume:	10.00 ul	Channel Name:	220.0nm
Run Time:	23.0 Minutes	Proc. Chnl. Descr.:	2998 PDA 220.0 nm (2998
Date Acquired:	7/24/2021 1:03:04 PM CST		
Date Processed:	8/29/2021 4:08:21 PM CST		

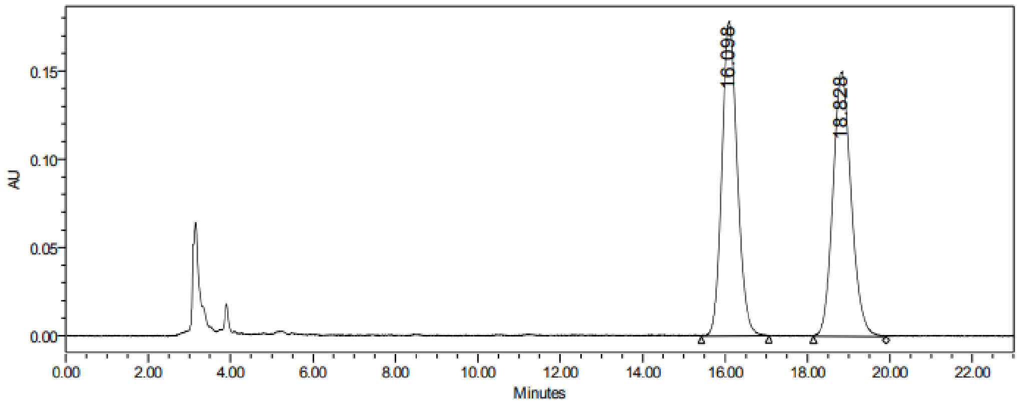

	RT	Area	% Area	Height
1	16.098	4673429	50.08	177825
2	18.828	4658646	49.92	149724

Asy-**3e**

SAMPLE INFORMATION

Sample Name:	zjy-5-181-20%-IC-asy	Acquired By:	System
Sample Type:	Unknown	Sample Set Name:	0
Vial:	67	Acq. Method Set:	20% quanbo
Injection #:	1	Processing Method	zjy 5 181 asy
Injection Volume:	10.00 ul	Channel Name:	220.0nm
Run Time:	23.0 Minutes	Proc. Chnl. Descr.:	2998 PDA 220.0 nm (2998
Date Acquired:	7/24/2021 12:15:42 PM CST		
Date Processed:	8/29/2021 4:06:26 PM CST		

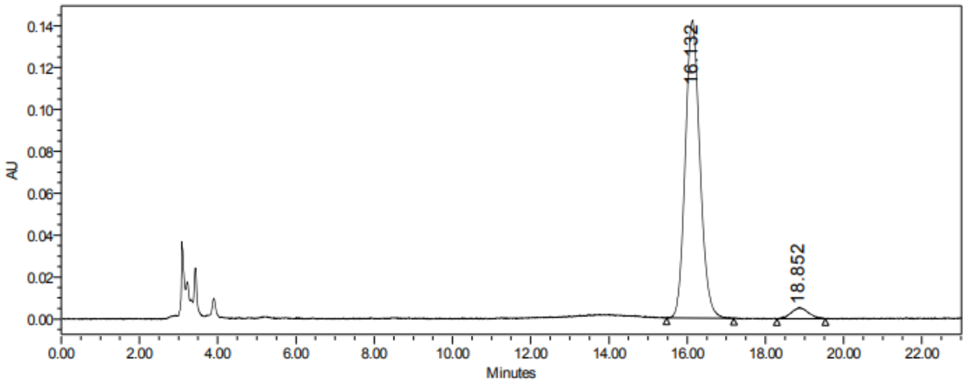

	RT	Area	% Area	Height
1	16.132	3717069	95.88	141952
2	18.852	159730	4.12	5287

Supplementary Figure 71. HPLC spectra of compound **3e**

Rac-**3f**

SAMPLE INFORMATION

Sample Name:	zjy-5-184-20%-IC-RAC	Acquired By:	System
Sample Type:	Unknown	Sample Set Name:	0
Vial:	57	Acq. Method Set:	20%quanbo
Injection #:	1	Processing Method:	ZJY 5 184 2
Injection Volume:	10.00 ul	Channel Name:	220.0nm
Run Time:	25.0 Minutes	Proc. Chnl. Descr.:	2998 PDA 220.0 nm (2998
Date Acquired:	7/30/2021 1:37:38 PM CST		
Date Processed:	8/29/2021 3:50:43 PM CST		

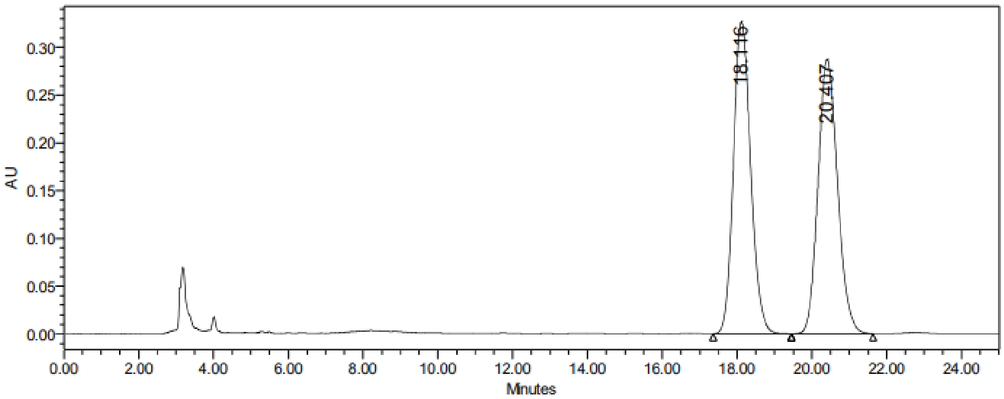

	RT	Area	% Area	Height
1	18.116	10359563	50.04	326592
2	20.407	10342733	49.96	287106

Asy-**3f**

SAMPLE INFORMATION

Sample Name:	zjy-5-184-20%-IC-asy	Acquired By:	System
Sample Type:	Unknown	Sample Set Name:	0
Vial:	56	Acq. Method Set:	20%quanbo
Injection #:	1	Processing Method:	ZJY 5 184
Injection Volume:	10.00 ul	Channel Name:	220.0nm
Run Time:	25.0 Minutes	Proc. Chnl. Descr.:	2998 PDA 220.0 nm (2998
Date Acquired:	7/30/2021 1:11:57 PM CST		
Date Processed:	8/29/2021 3:47:26 PM CST		

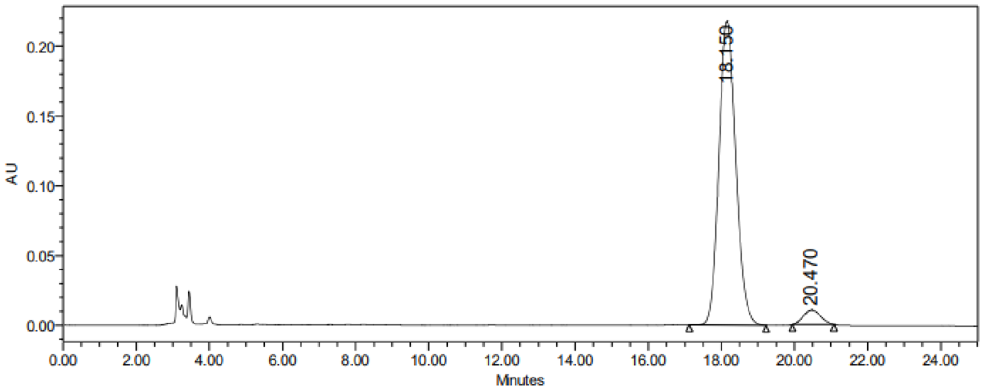

	RT	Area	% Area	Height
1	18.150	6917003	95.25	217898
2	20.470	344833	4.75	10481

Supplementary Figure 72. HPLC spectra of compound **3f**

Rac-**3g**

SAMPLE INFORMATION

Sample Name:	zjy-6-49-10%-AD-RAC	Acquired By:	System
Sample Type:	Unknown	Sample Set Name:	0
Vial:	66	Acq. Method Set:	10%quanbo
Injection #:	1	Processing Method:	54165463
Injection Volume:	10.00 ul	Channel Name:	220.0nm
Run Time:	25.0 Minutes	Proc. Chnl. Descr.:	2998 PDA 220.0 nm (2998
Date Acquired:	9/12/2021 6:13:27 PM CST		
Date Processed:	9/12/2021 8:10:16 PM CST		

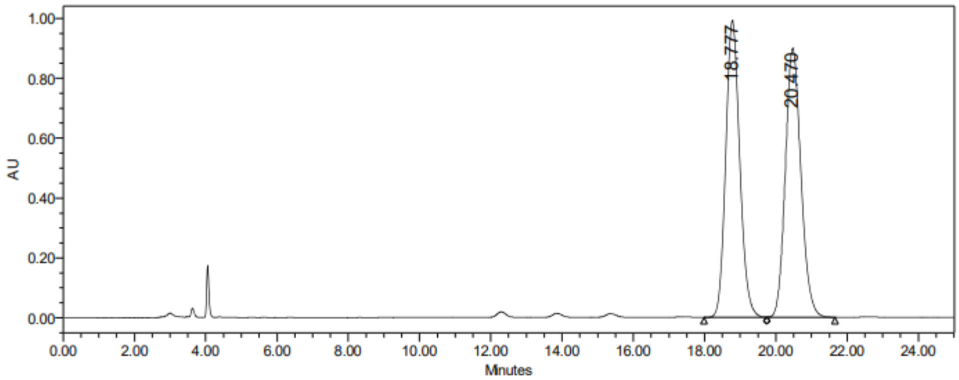

	RT	Area	% Area	Height
1	18.777	27181598	49.99	990632
2	20.470	27188548	50.01	898285

Asy-**3g**

SAMPLE INFORMATION

Sample Name:	zjy-6-49-10%-AD-asy	Acquired By:	System
Sample Type:	Unknown	Sample Set Name:	0
Vial:	67	Acq. Method Set:	10%quanbo
Injection #:	1	Processing Method:	54165463
Injection Volume:	10.00 ul	Channel Name:	254.0nm
Run Time:	25.0 Minutes	Proc. Chnl. Descr.:	2998 PDA 254.0 nm (2998
Date Acquired:	9/12/2021 6:39:08 PM CST		
Date Processed:	9/12/2021 8:09:15 PM CST		

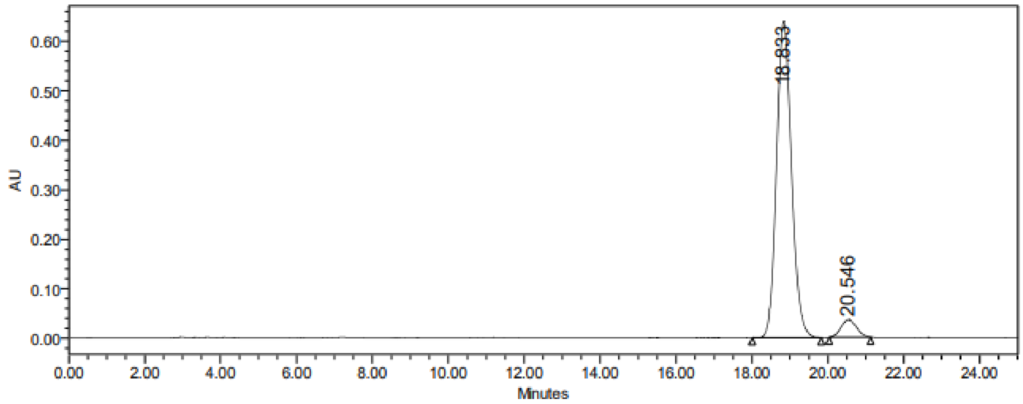

	RT	Area	% Area	Height
1	18.833	17560832	94.53	639165
2	20.546	1016260	5.47	35651

Supplementary Figure 73. HPLC spectra of compound **3g**

Rac-**3h**

SAMPLE INFORMATION

Sample Name:	zjy-5-122-10%-AD-RAC	Acquired By:	System
Sample Type:	Unknown	Sample Set Name	0
Vial:	70	Acq. Method Set:	10% quanbo
Injection #:	1	Processing Method	zjy 5 122 rac
Injection Volume:	10.00 ul	Channel Name:	254.0nm
Run Time:	40.0 Minutes	Proc. Chnl. Descr.:	2998 PDA 254.0 nm (2998
Date Acquired:	6/30/2021 5:25:17 PM CST		
Date Processed:	6/30/2021 6:35:54 PM CST		

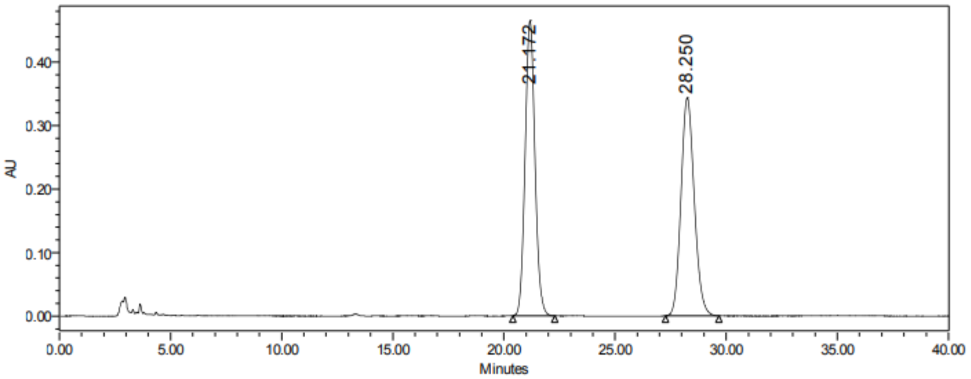

	RT	Area	% Area	Height
1	21.172	14425925	50.01	464791
2	28.250	14419743	49.99	343661

Asy-**3h**

SAMPLE INFORMATION

Sample Name:	zjy-5-122-10%-AD-asy	Acquired By:	System
Sample Type:	Unknown	Sample Set Name:	0
Vial:	68	Acq. Method Set:	10% quanbo
Injection #:	1	Processing Method	zjy 5 122 asy
Injection Volume:	10.00 ul	Channel Name:	254.0nm
Run Time:	40.0 Minutes	Proc. Chnl. Descr.:	2998 PDA 254.0 nm (2998
Date Acquired:	6/30/2021 4:23:37 PM CST		
Date Processed:	7/4/2021 4:40:14 PM CST		

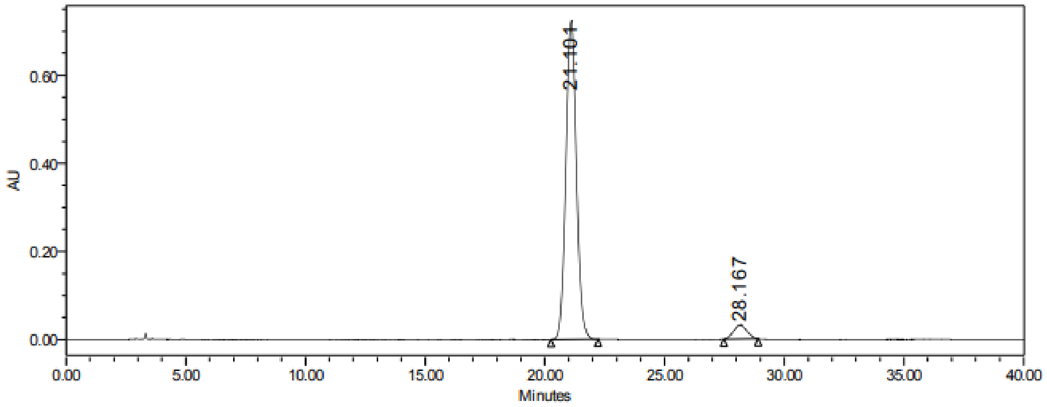

	RT	Area	% Area	Height
1	21.101	22310062	94.84	722260
2	28.167	1212626	5.16	31254

Supplementary Figure 74. HPLC spectra of compound **3h**

Rac-**3i**

SAMPLE INFORMATION

Sample Name:	zjy-5-113-10%-AD-RAC	Acquired By:	System
Sample Type:	Unknown	Sample Set Name:	0
Vial:	49	Acq. Method Set:	10%quanbo
Injection #:	1	Processing Method:	ZJY 5 113
Injection Volume:	10.00 ul	Channel Name:	254.0nm
Run Time:	30.0 Minutes	Proc. Chnl. Descr.:	2998 PDA 254.0 nm (2998
Date Acquired:	6/28/2021 5:25:27 PM CST		
Date Processed:	7/15/2021 9:46:52 PM CST		

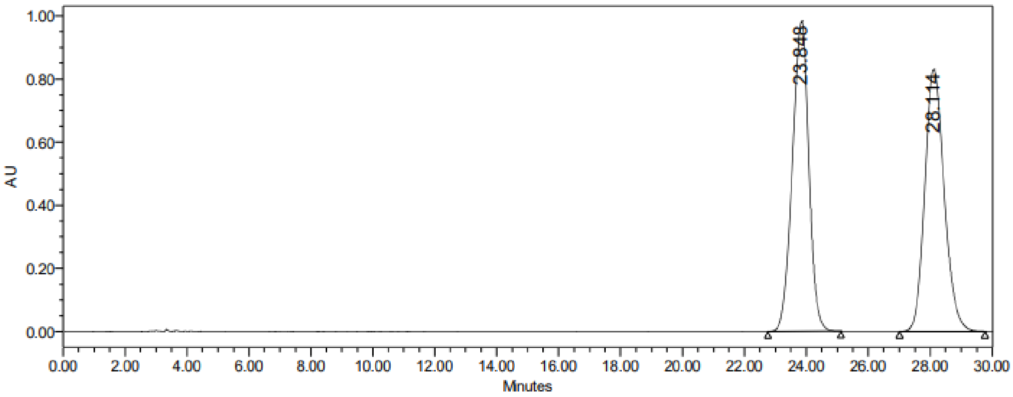

	RT	Area	% Area	Height
1	23.848	36390260	50.00	981799
2	28.114	36388000	50.00	830101

Asy-**3i**

SAMPLE INFORMATION

Sample Name:	zjy-5-113-10%-AD-asy	Acquired By:	System
Sample Type:	Unknown	Sample Set Name:	0
Vial:	50	Acq. Method Set:	10%quanbo
Injection #:	1	Processing Method:	ZJY 5 113
Injection Volume:	10.00 ul	Channel Name:	254.0nm
Run Time:	30.0 Minutes	Proc. Chnl. Descr.:	2998 PDA 254.0 nm (2998
Date Acquired:	6/28/2021 5:56:07 PM CST		
Date Processed:	7/15/2021 9:45:29 PM CST		

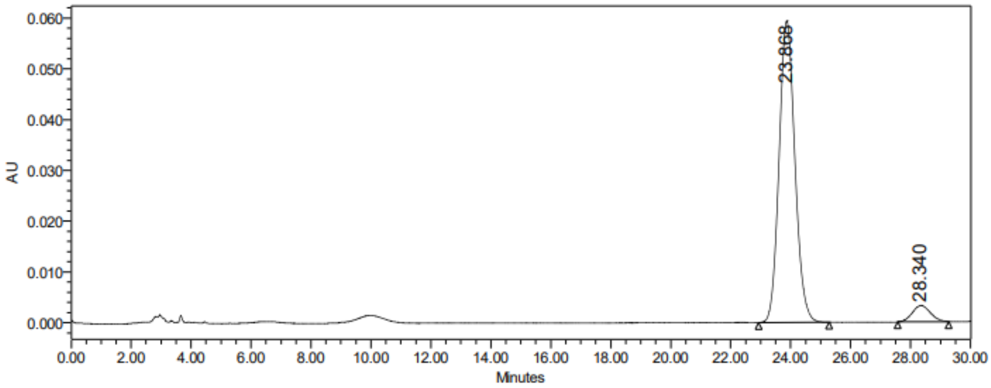

	RT	Area	% Area	Height
1	23.868	2169570	94.19	59330
2	28.340	133815	5.81	3199

Supplementary Figure 75. HPLC spectra of compound **3i**

Rac-**3j**

SAMPLE INFORMATION			
Sample Name:	zjy-5-114-30%-IE-RAC	Acquired By:	System
Sample Type:	Unknown	Sample Set Name:	0
Vial:	49	Acq. Method Set:	30%quanbo
Injection #:	1	Processing Method:	1354685746
Injection Volume:	10.00 ul	Channel Name:	254.0nm
Run Time:	24.0 Minutes	Proc. Chnl. Descr.:	2998 PDA 254.0 nm (2998
Date Acquired:	8/29/2021 9:02:30 PM CST		
Date Processed:	8/30/2021 9:40:29 PM CST		

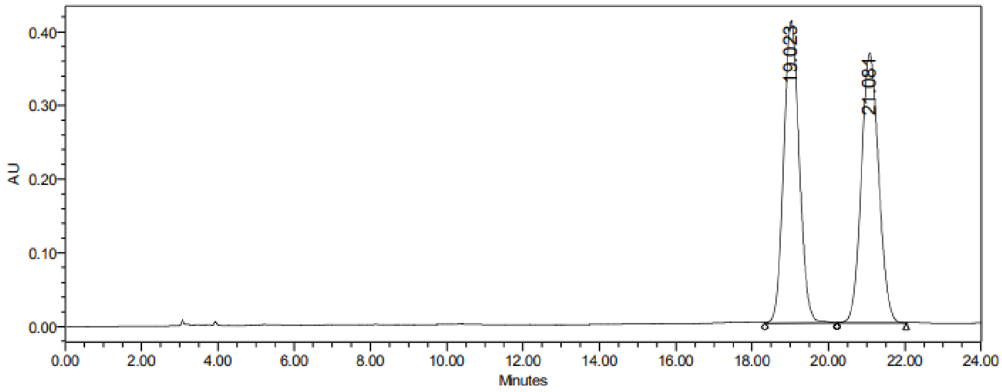

	RT	Area	% Area	Height
1	19.023	11654192	50.22	410096
2	21.081	11552172	49.78	365876

Asy-**3j**

SAMPLE INFORMATION			
Sample Name:	zjy-5-114-30%-IE-asy	Acquired By:	System
Sample Type:	Unknown	Sample Set Name:	0
Vial:	50	Acq. Method Set:	30%quanbo
Injection #:	1	Processing Method:	0
Injection Volume:	10.00 ul	Channel Name:	254.0nm
Run Time:	24.0 Minutes	Proc. Chnl. Descr.:	2998 PDA 254.0 nm (2998
Date Acquired:	8/29/2021 9:27:12 PM CST		
Date Processed:	8/30/2021 9:42:01 PM CST		

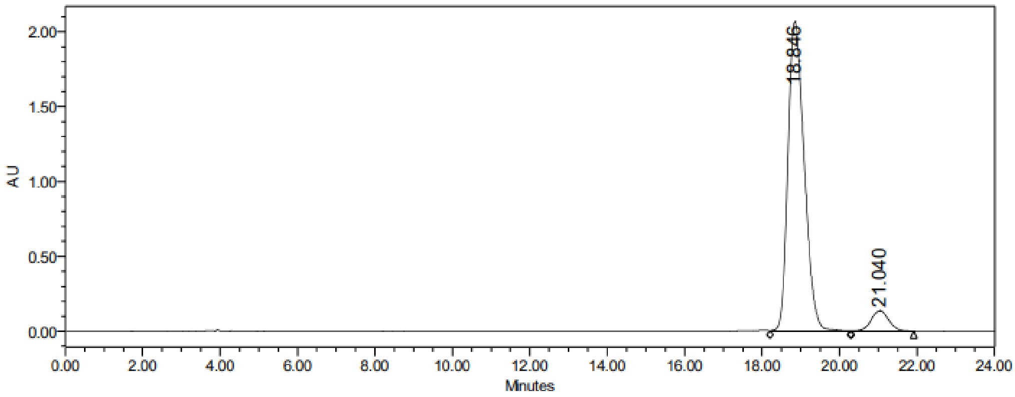

	RT	Area	% Area	Height
1	18.846	60279138	93.33	2067294
2	21.040	4305348	6.67	136604

Supplementary Figure 76. HPLC spectra of compound **3j**

Rac-**3k**

SAMPLE INFORMATION

Sample Name:	zjy-5-118-10%-AD-RAC	Acquired By:	System
Sample Type:	Unknown	Sample Set Name:	0
Vial:	54	Acq. Method Set:	10%quanbo
Injection #:	1	Processing Method:	ZJY 5 118
Injection Volume:	10.00 ul	Channel Name:	254.0nm
Run Time:	30.0 Minutes	Proc. Chnl. Descr.:	2998 PDA 254.0 nm (2998
Date Acquired:	6/29/2021 7:33:31 PM CST		
Date Processed:	7/15/2021 9:56:53 PM CST		

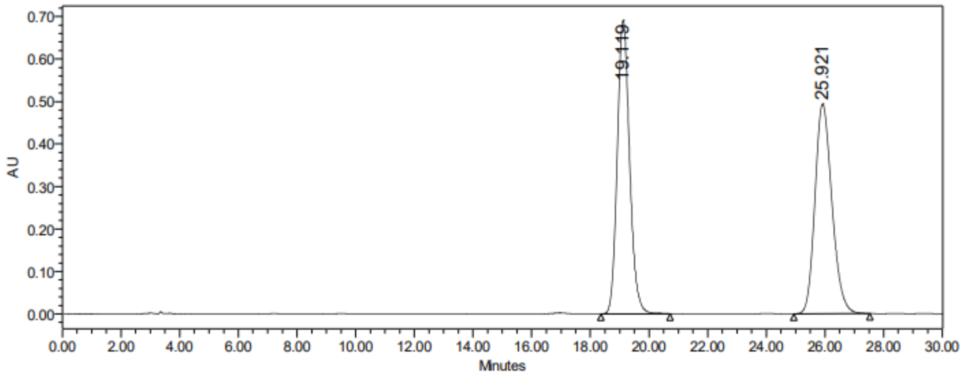

	RT	Area	% Area	Height
1	19.119	20087205	50.18	690386
2	25.921	19946841	49.82	494069

Asy-**3k**

SAMPLE INFORMATION

Sample Name:	zjy-5-118-10%-AD-asy	Acquired By:	System
Sample Type:	Unknown	Sample Set Name:	0
Vial:	52	Acq. Method Set:	10%quanbo
Injection #:	1	Processing Method:	ZJY 5 118
Injection Volume:	10.00 ul	Channel Name:	254.0nm
Run Time:	30.0 Minutes	Proc. Chnl. Descr.:	2998 PDA 254.0 nm (2998
Date Acquired:	6/29/2021 6:27:05 PM CST		
Date Processed:	7/15/2021 9:55:55 PM CST		

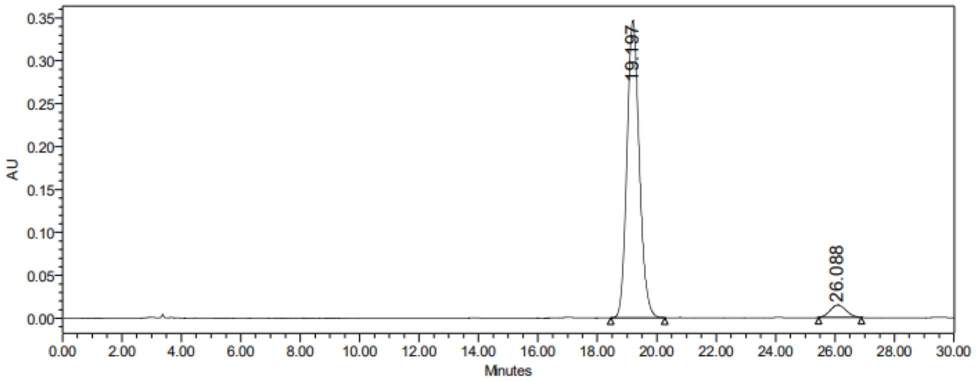

	RT	Area	% Area	Height
1	19.197	9974018	94.82	346115
2	26.088	545349	5.18	14478

Supplementary Figure 77. HPLC spectra of compound **3k**

Rac-**3l**

SAMPLE INFORMATION

Sample Name:	zjy-5-117-10%-AD-RAC	Acquired By:	System
Sample Type:	Unknown	Sample Set Name:	0
Vial:	53	Acq. Method Set:	10%quanbo
Injection #:	1	Processing Method:	ZJY 5 117 RAC
Injection Volume:	10.00 ul	Channel Name:	254.0nm
Run Time:	35.0 Minutes	Proc. Chnl. Descr.:	2998 PDA 254.0 nm (2998
Date Acquired:	6/29/2021 6:57:47 PM CST		
Date Processed:	7/15/2021 9:53:44 PM CST		

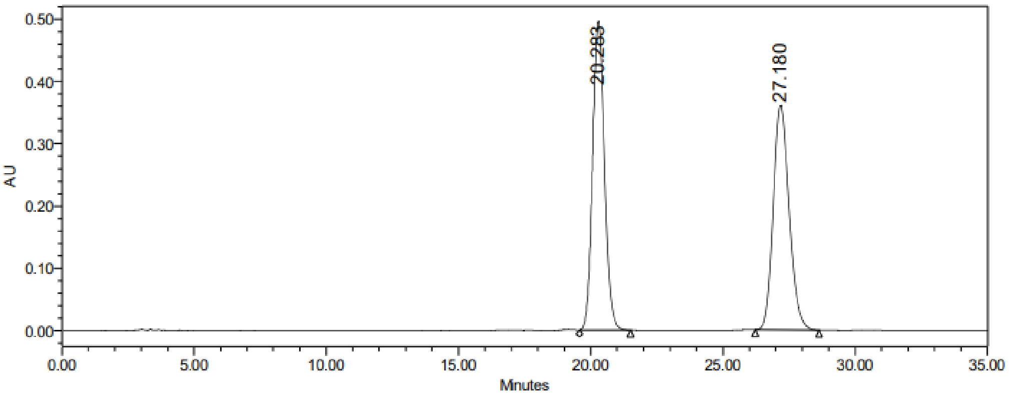

	RT	Area	% Area	Height
1	20.283	15264107	50.12	495337
2	27.180	15189901	49.88	360013

Asy-**3l**

SAMPLE INFORMATION

Sample Name:	zjy-5-117-10%-AD-asy	Acquired By:	System
Sample Type:	Unknown	Sample Set Name:	0
Vial:	51	Acq. Method Set:	10%quanbo
Injection #:	1	Processing Method:	ZJY 5 117 ASY
Injection Volume:	10.00 ul	Channel Name:	254.0nm
Run Time:	35.0 Minutes	Proc. Chnl. Descr.:	2998 PDA 254.0 nm (2998
Date Acquired:	6/29/2021 5:51:24 PM CST		
Date Processed:	7/15/2021 9:52:11 PM CST		

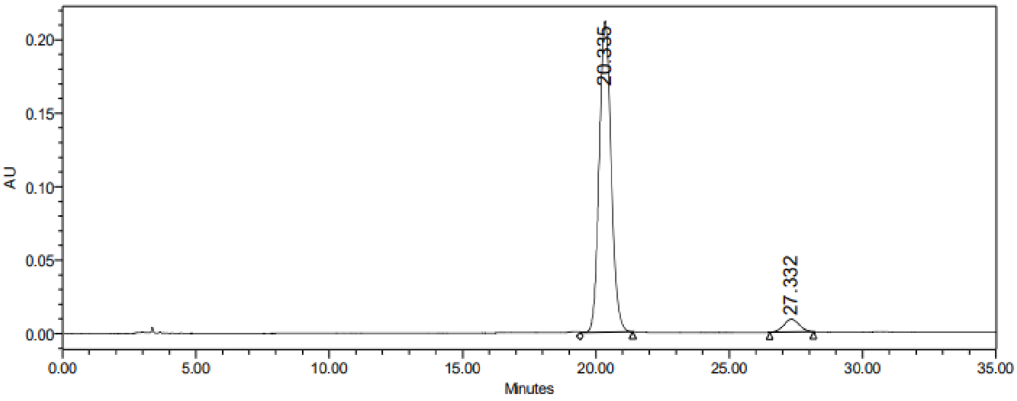

	RT	Area	% Area	Height
1	20.335	6479608	94.80	211218
2	27.332	355630	5.20	8780

Supplementary Figure 78. HPLC spectra of compound **3l**

Rac-**3m**

SAMPLE INFORMATION

Sample Name:	zjy-7-324-30%-IC-RAC	Acquired By:	System
Sample Type:	Unknown	Sample Set Name:	
Vial:	64	Acq. Method Set:	30%quanbo
Injection #:	1	Processing Method:	54165463
Injection Volume:	10.00 ul	Channel Name:	210.3nm
Run Time:	100.0 Minutes	Proc. Chnl. Descr.:	2998 PDA 210.3 nm (2998
Date Acquired:	4/15/2022 7:55:31 PM CST		
Date Processed:	6/23/2022 11:34:14 PM CST		

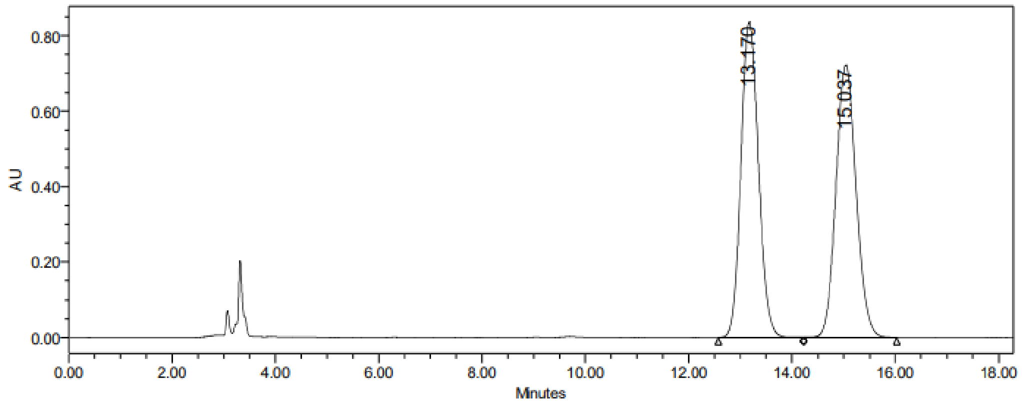

	RT	Area	% Area	Height
1	13.170	19514699	50.00	836316
2	15.037	19512178	50.00	722214

asy-**3m**

SAMPLE INFORMATION

Sample Name:	zjy-7-324-30%-IC-asy	Acquired By:	System
Sample Type:	Unknown	Sample Set Name:	0
Vial:	67	Acq. Method Set:	30%quanbo
Injection #:	1	Processing Method:	1354685746
Injection Volume:	3.00 ul	Channel Name:	254.0nm
Run Time:	18.0 Minutes	Proc. Chnl. Descr.:	2998 PDA 254.0 nm (2998
Date Acquired:	4/15/2022 9:12:51 PM CST		
Date Processed:	6/23/2022 11:36:30 PM CST		

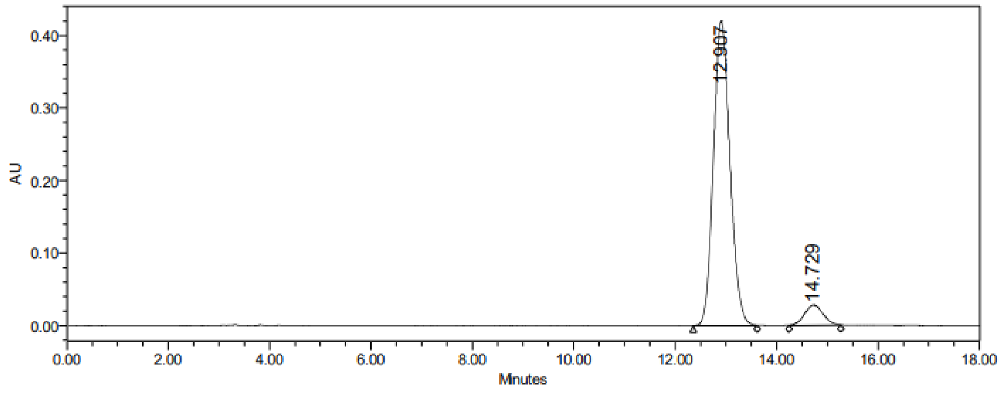

	RT	Area	% Area	Height
1	12.907	9489520	92.90	419662
2	14.729	724811	7.10	27849

Supplementary Figure 79. HPLC spectra of compound **3m**

Rac-**3n**

SAMPLE INFORMATION

Sample Name:	zjy-7-323-30%-IC-RAC	Acquired By:	System
Sample Type:	Unknown	Sample Set Name:	
Vial:	63	Acq. Method Set:	30%quanbo
Injection #:	1	Processing Method:	0
Injection Volume:	10.00 ul	Channel Name:	254.0nm
Run Time:	100.0 Minutes	Proc. Chnl. Descr.:	2998 PDA 254.0 nm (2998
Date Acquired:	4/15/2022 7:34:11 PM CST		
Date Processed:	6/23/2022 11:32:27 PM CST		

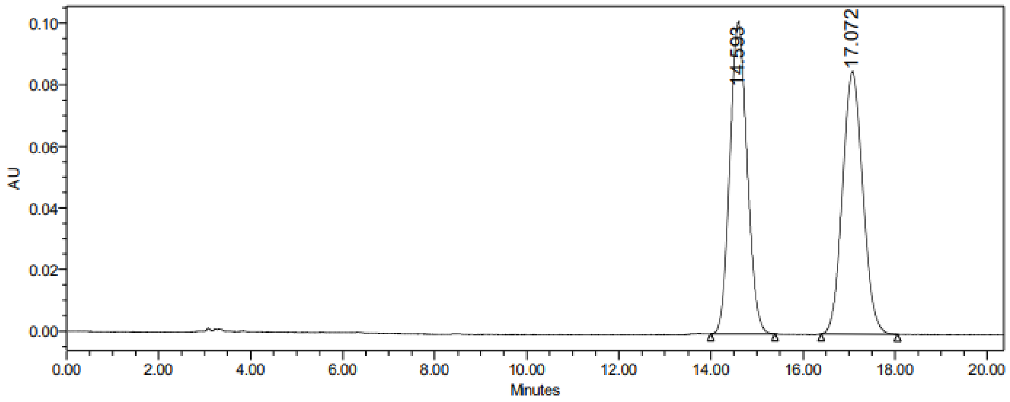

	RT	Area	% Area	Height
1	14.593	2632768	49.95	101481
2	17.072	2637937	50.05	85306

asy-**3n**

SAMPLE INFORMATION

Sample Name:	zjy-7-323-30%-IC-asy	Acquired By:	System
Sample Type:	Unknown	Sample Set Name:	0
Vial:	66	Acq. Method Set:	30%quanbo
Injection #:	1	Processing Method:	1354685746
Injection Volume:	10.00 ul	Channel Name:	254.0nm
Run Time:	20.0 Minutes	Proc. Chnl. Descr.:	2998 PDA 254.0 nm (2998
Date Acquired:	4/15/2022 8:51:35 PM CST		
Date Processed:	6/23/2022 11:35:10 PM CST		

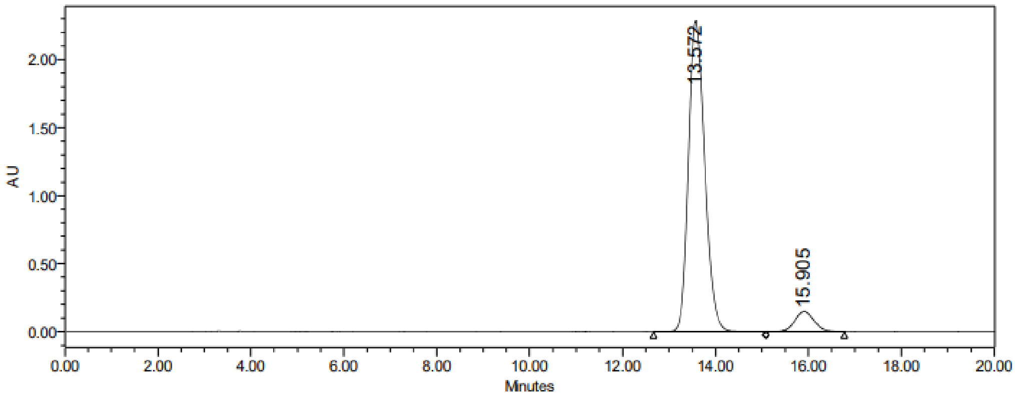

	RT	Area	% Area	Height
1	13.572	55929265	92.96	2280461
2	15.905	4233747	7.04	147869

Supplementary Figure 80. HPLC spectra of compound **3n**

Rac-**3o**

SAMPLE INFORMATION

Sample Name:	zjy-5-161-3%-AD-RAC	Acquired By:	System
Sample Type:	Unknown	Sample Set Name:	0
Vial:	69	Acq. Method Set:	3%quanbo
Injection #:	1	Processing Method:	ZJY 5 161
Injection Volume:	5.00 ul	Channel Name:	220.0nm
Run Time:	55.0 Minutes	Proc. Chnl. Descr.:	2998 PDA 220.0 nm (2998
Date Acquired:	7/19/2021 7:43:47 PM CST		
Date Processed:	7/19/2021 10:06:16 PM CST		

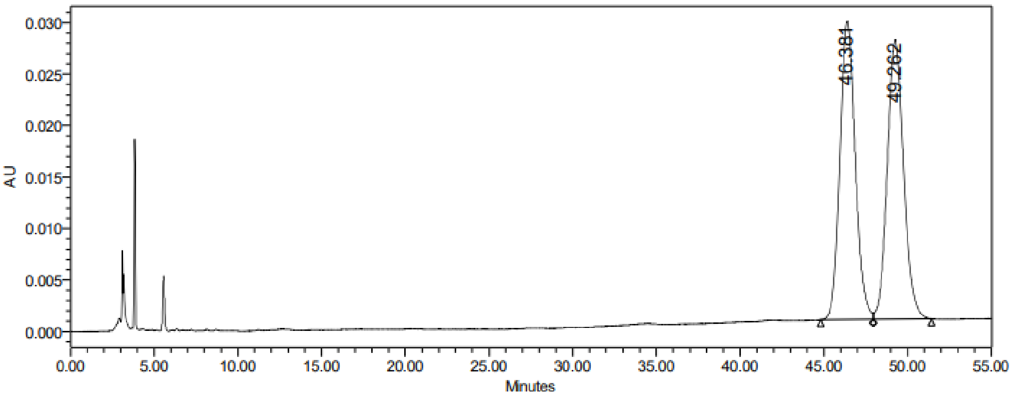

	RT	Area	% Area	Height
1	46.381	1850788	50.05	28951
2	49.262	1847220	49.95	27117

Asy-**3o**

SAMPLE INFORMATION

Sample Name:	zjy-5-161-3%-AD-asy	Acquired By:	System
Sample Type:	Unknown	Sample Set Name:	0
Vial:	68	Acq. Method Set:	3%quanbo
Injection #:	1	Processing Method:	ZJY 5 118
Injection Volume:	10.00 ul	Channel Name:	220.0nm
Run Time:	55.0 Minutes	Proc. Chnl. Descr.:	2998 PDA 220.0 nm (2998
Date Acquired:	7/19/2021 6:48:07 PM CST		
Date Processed:	7/19/2021 10:07:34 PM CST		

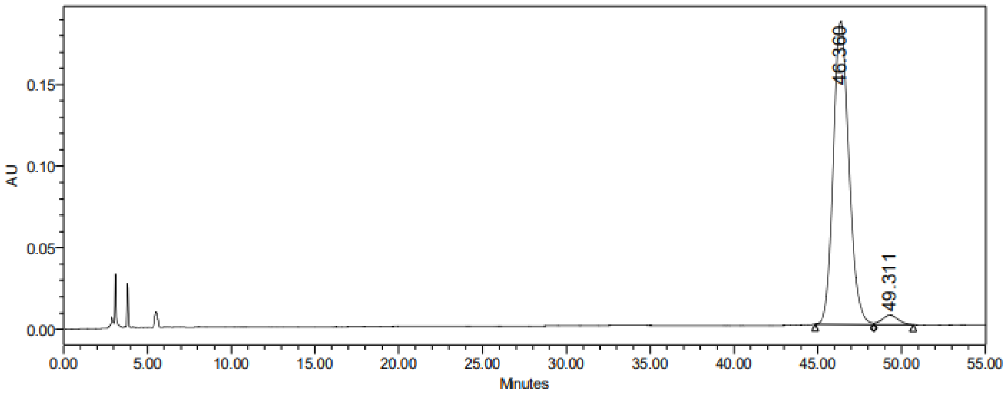

	RT	Area	% Area	Height
1	46.360	12111079	96.89	185661
2	49.311	388930	3.11	5864

Supplementary Figure 81. HPLC spectra of compound **3o**

Rac-**3p**

SAMPLE INFORMATION

Sample Name:		Acquired By:	System
Sample Type:	Unknown	Sample Set Name:	
Vial:	114	Acq. Method Set:	10% quanbo 08 mL min
Injection #:	1	Processing Method	44564
Injection Volume:	10.00 ul	Channel Name:	220.0nm
Run Time:	150.0 Minutes	Proc. Chnl. Descr.:	2998 PDA 220.0 nm (2998
Date Acquired:	9/6/2021 8:38:36 PM CST		
Date Processed:	9/8/2021 3:45:38 PM CST		

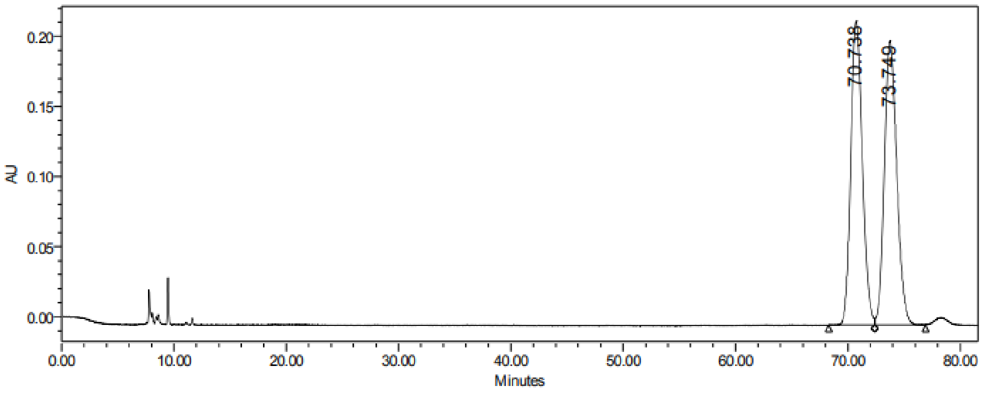

	RT	Area	% Area	Height
1	70.738	15514071	49.88	216543
2	73.749	15591433	50.12	202924

Asy-**3p**

SAMPLE INFORMATION

Sample Name:		Acquired By:	System
Sample Type:	Unknown	Sample Set Name:	0
Vial:	72	Acq. Method Set:	10% quanbo 08 mL min
Injection #:	1	Processing Method	44564
Injection Volume:	10.00 ul	Channel Name:	220.0nm
Run Time:	80.0 Minutes	Proc. Chnl. Descr.:	2998 PDA 220.0 nm (2998
Date Acquired:	9/7/2021 1:17:27 AM CST		
Date Processed:	9/8/2021 3:44:25 PM CST		

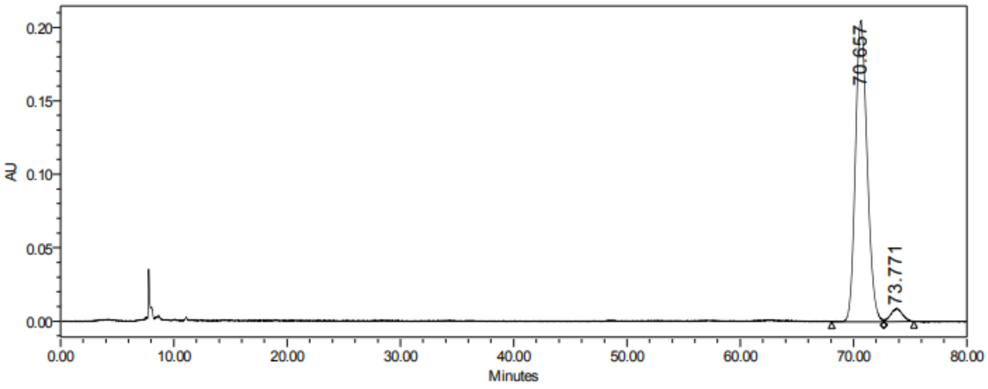

	RT	Area	% Area	Height
1	70.657	14861833	95.86	204656
2	73.771	641337	4.14	8892

Supplementary Figure 82. HPLC spectra of compound **3p**

Rac-**3q**

SAMPLE INFORMATION

Sample Name:	zjy-5-185-20%-IE-RAC	Acquired By:	System
Sample Type:	Unknown	Sample Set Name:	0
Vial:	50	Acq. Method Set:	20% quanbo
Injection #:	1	Processing Method	zjy 5 185 rac
Injection Volume:	10.00 ul	Channel Name:	220.0nm
Run Time:	50.0 Minutes	Proc. Chnl. Descr.:	2998 PDA 220.0 nm (2998
Date Acquired:	7/26/2021 12:44:34 PM CST		
Date Processed:	8/29/2021 4:04:29 PM CST		

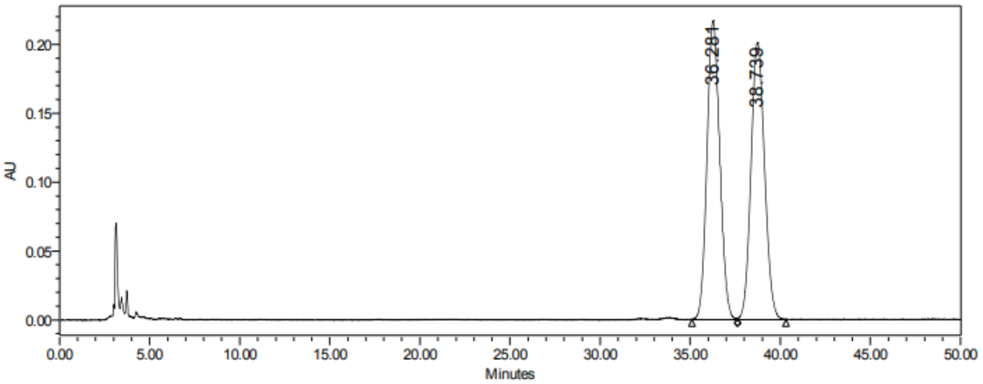

	RT	Area	% Area	Height
1	36.281	10608291	50.00	216859
2	38.739	10606765	50.00	201101

Asy-**3q**

SAMPLE INFORMATION

Sample Name:	zjy-5-185-20%-IE-asy	Acquired By:	System
Sample Type:	Unknown	Sample Set Name:	0
Vial:	49	Acq. Method Set:	20% quanbo
Injection #:	1	Processing Method	zjy 5 185 asy
Injection Volume:	10.00 ul	Channel Name:	220.0nm
Run Time:	50.0 Minutes	Proc. Chnl. Descr.:	2998 PDA 220.0 nm (2998
Date Acquired:	7/26/2021 11:53:52 AM CST		
Date Processed:	8/29/2021 4:02:45 PM CST		

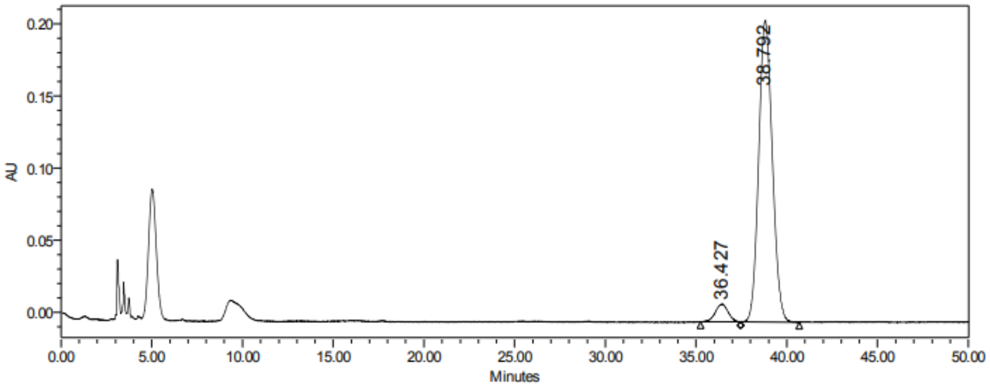

	RT	Area	% Area	Height
1	36.427	604236	5.23	12455
2	38.792	10955049	94.77	208620

Supplementary Figure 83. HPLC spectra of compound **3q**

Rac-**3r**

SAMPLE INFORMATION

Sample Name:	zjy-5-198-10%-IC-RAC	Acquired By:	System
Sample Type:	Unknown	Sample Set Name:	0
Vial:	52	Acq. Method Set:	10%quanbo
Injection #:	1	Processing Method:	ZJY 5 198 RAC
Injection Volume:	10.00 ul	Channel Name:	220.0nm
Run Time:	40.0 Minutes	Proc. Chnl. Descr.:	2998 PDA 220.0 nm (2998
Date Acquired:	7/29/2021 7:17:57 PM CST		
Date Processed:	8/29/2021 3:40:03 PM CST		

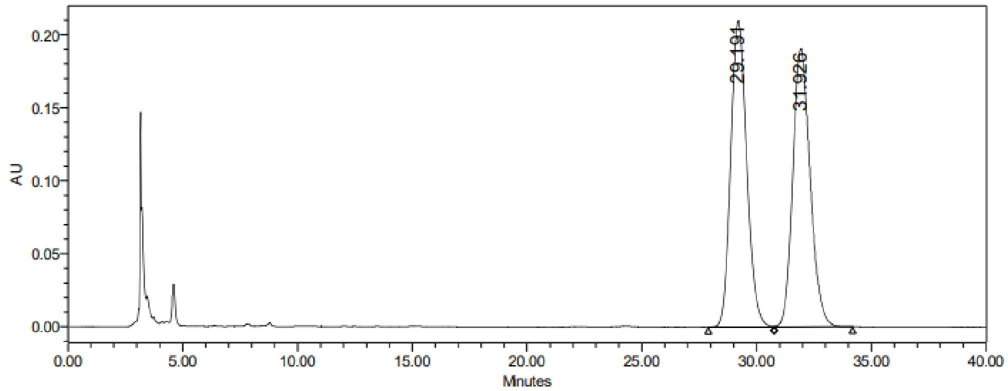

	RT	Area	% Area	Height
1	29.191	10031031	49.98	209482
2	31.926	10038772	50.02	190309

Asy-**3r**

SAMPLE INFORMATION

Sample Name:	zjy-5-198-10%-IC-asy	Acquired By:	System
Sample Type:	Unknown	Sample Set Name:	0
Vial:	51	Acq. Method Set:	10%quanbo
Injection #:	1	Processing Method:	ZJY 5 198
Injection Volume:	10.00 ul	Channel Name:	220.0nm
Run Time:	40.0 Minutes	Proc. Chnl. Descr.:	2998 PDA 220.0 nm (2998
Date Acquired:	7/29/2021 6:37:14 PM CST		
Date Processed:	8/29/2021 3:38:13 PM CST		

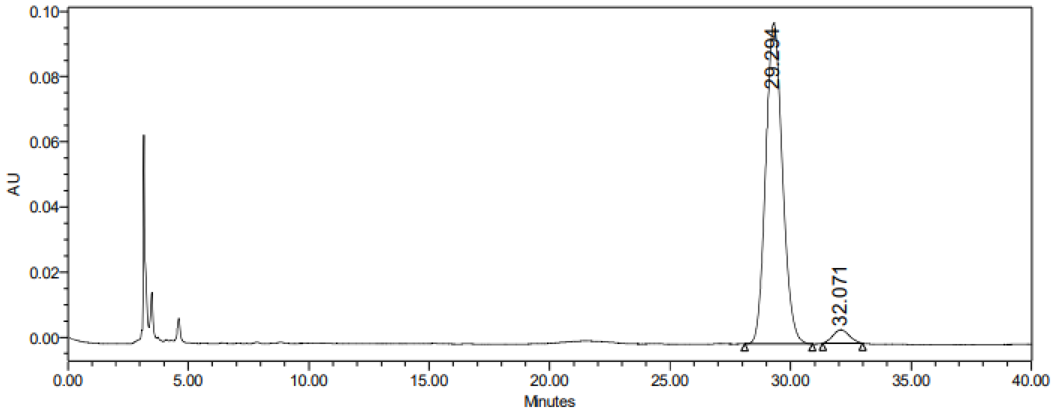

	RT	Area	% Area	Height
1	29.294	4714934	95.87	98547
2	32.071	203209	4.13	4265

Supplementary Figure 84. HPLC spectra of compound **3r**

Rac-**3s**

SAMPLE INFORMATION

Sample Name:	zjy-5-199-10%-IC-RAC	Acquired By:	System
Sample Type:	Unknown	Sample Set Name:	0
Vial:	54	Acq. Method Set:	10%quanbo
Injection #:	1	Processing Method:	ZJY 5 199 RAC
Injection Volume:	10.00 ul	Channel Name:	220.0nm
Run Time:	40.0 Minutes	Proc. Chnl. Descr.:	2998 PDA 220.0 nm (2998
Date Acquired:	7/29/2021 7:58:37 PM CST		
Date Processed:	8/29/2021 3:25:53 PM CST		

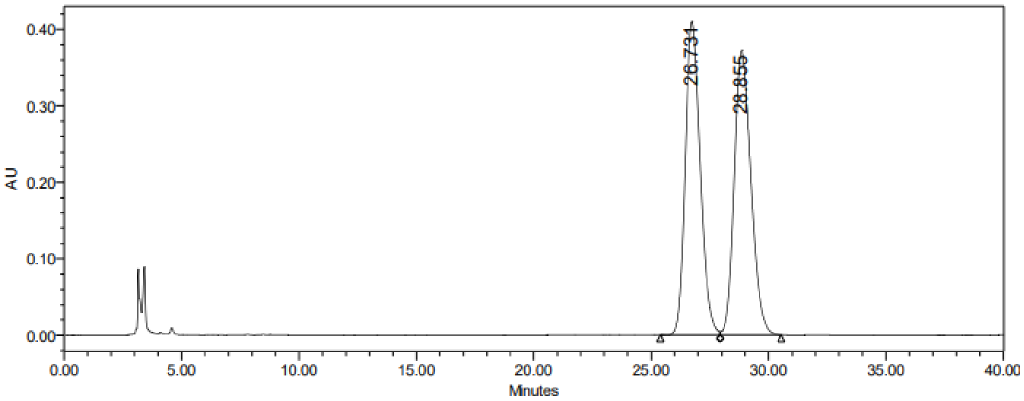

	RT	Area	% Area	Height
1	26.731	18243795	49.98	409553
2	28.855	18255210	50.02	372034

Asy-**3s**

SAMPLE INFORMATION

Sample Name:	zjy-5-199-10%-IC-asy	Acquired By:	System
Sample Type:	Unknown	Sample Set Name:	0
Vial:	51	Acq. Method Set:	10%quanbo
Injection #:	1	Processing Method:	ZJY 5 199 ASY
Injection Volume:	10.00 ul	Channel Name:	220.0nm
Run Time:	40.0 Minutes	Proc. Chnl. Descr.:	2998 PDA 220.0 nm (2998
Date Acquired:	7/29/2021 12:36:01 PM CST		
Date Processed:	8/29/2021 3:24:42 PM CST		

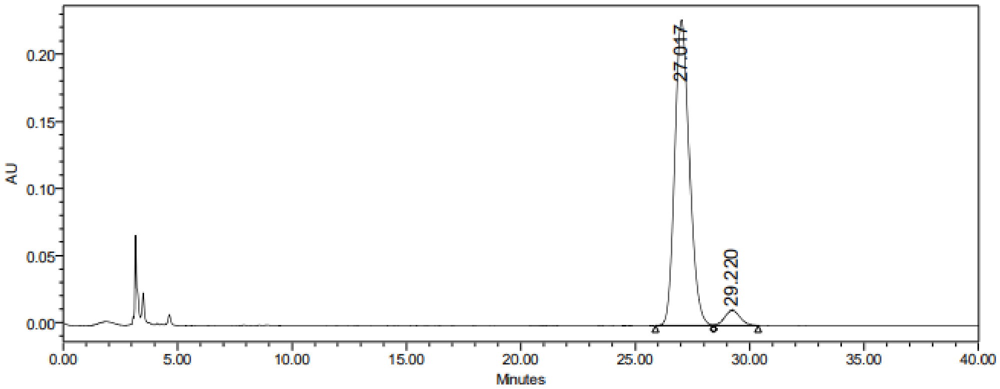

	RT	Area	% Area	Height
1	27.017	10232210	94.85	227443
2	29.220	555452	5.15	11531

Supplementary Figure 85. HPLC spectra of compound **3s**

Rac-**3t**

SAMPLE INFORMATION

Sample Name:	zjy-5-145-3%-OD-RAC	Acquired By:	System
Sample Type:	Unknown	Sample Set Name:	0
Vial:	56	Acq. Method Set:	3% quanbo
Injection #:	1	Processing Method	44564
Injection Volume:	10.00 ul	Channel Name:	220.0nm
Run Time:	14.0 Minutes	Proc. Chnl. Descr.:	2998 PDA 220.0 nm (2998
Date Acquired:	8/30/2021 12:29:43 AM CST		
Date Processed:	8/30/2021 9:33:00 PM CST		

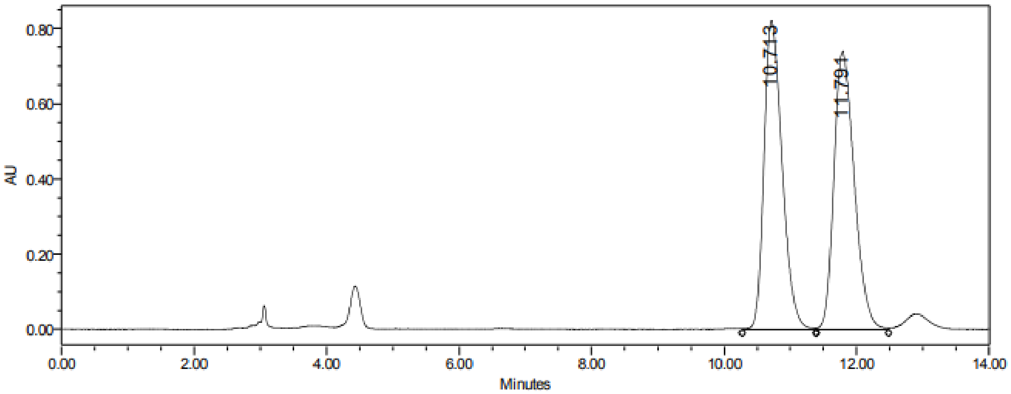

	RT	Area	% Area	Height
1	10.713	15717912	49.98	819940
2	11.791	15727623	50.02	739013

Asy-**3t**

SAMPLE INFORMATION

Sample Name:	zjy-5-145-3%-OD-asy	Acquired By:	System
Sample Type:	Unknown	Sample Set Name:	0
Vial:	57	Acq. Method Set:	3% quanbo
Injection #:	1	Processing Method	44564
Injection Volume:	10.00 ul	Channel Name:	220.0nm
Run Time:	14.0 Minutes	Proc. Chnl. Descr.:	2998 PDA 220.0 nm (2998
Date Acquired:	8/30/2021 12:44:26 AM CST		
Date Processed:	8/30/2021 9:34:42 PM CST		

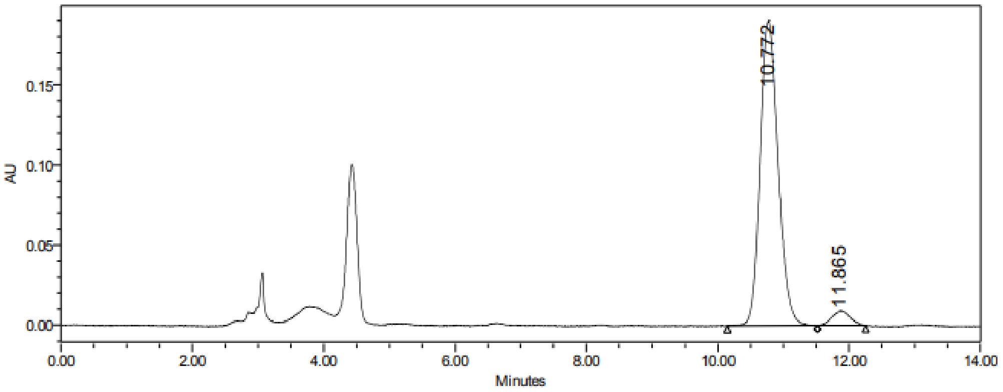

	RT	Area	% Area	Height
1	10.772	3647016	95.34	190262
2	11.865	178382	4.66	9261

Supplementary Figure 86. HPLC spectra of compound **3t**

Rac-**3u**

SAMPLE INFORMATION

Sample Name:	zjy-5-201-5%-IG-RAC	Acquired By:	System
Sample Type:	Unknown	Sample Set Name:	0
Vial:	55	Acq. Method Set:	5%quanbo
Injection #:	1	Processing Method:	LC PQ
Injection Volume:	10.00 ul	Channel Name:	220.0nm
Run Time:	80.0 Minutes	Proc. Chnl. Descr.:	2998 PDA 220.0 nm (2998
Date Acquired:	7/29/2021 11:16:02 PM CST		
Date Processed:	8/9/2021 6:28:53 PM CST		

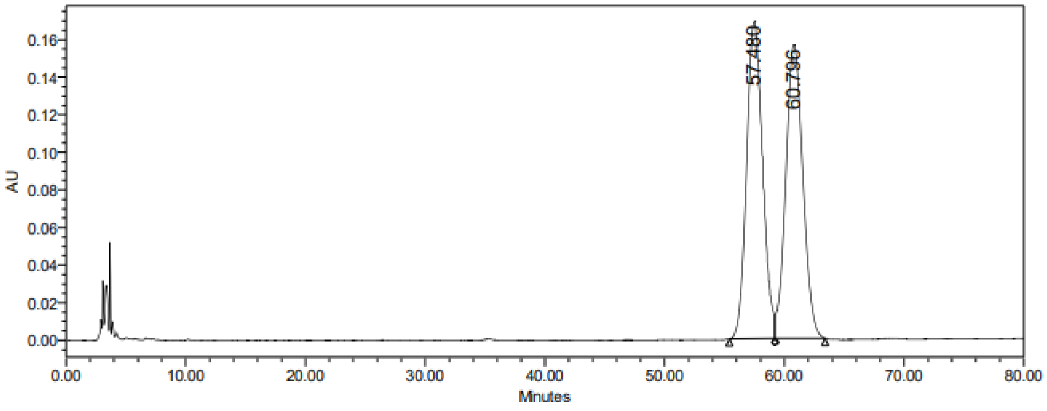

	RT	Area	% Area	Height
1	57.480	15557014	49.95	168829
2	60.796	15585523	50.05	156190

Asy-**3u**

SAMPLE INFORMATION

Sample Name:	zjy-5-201-5%-IG-asy	Acquired By:	System
Sample Type:	Unknown	Sample Set Name:	0
Vial:	56	Acq. Method Set:	5%quanbo
Injection #:	1	Processing Method:	LC PQ
Injection Volume:	10.00 ul	Channel Name:	220.0nm
Run Time:	80.0 Minutes	Proc. Chnl. Descr.:	2998 PDA 220.0 nm (2998
Date Acquired:	7/30/2021 12:36:43 AM CST		
Date Processed:	8/9/2021 6:27:12 PM CST		

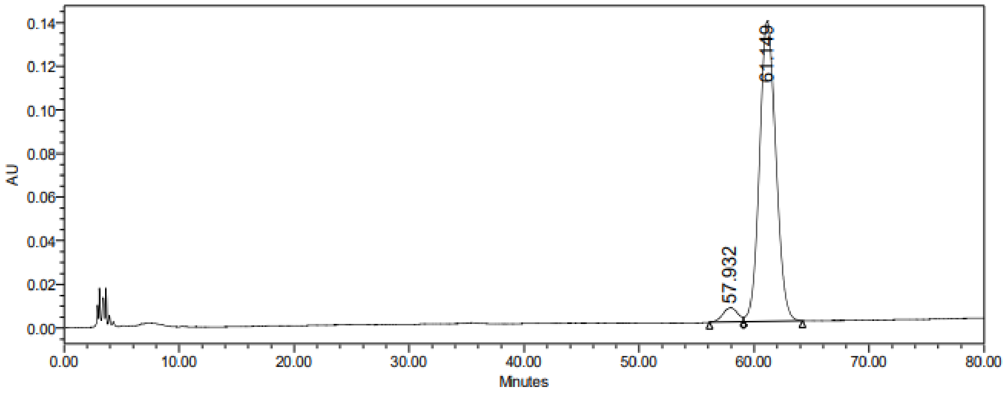

	RT	Area	% Area	Height
1	57.932	590324	4.14	6425
2	61.149	13672066	95.86	137668

Supplementary Figure 87. HPLC spectra of compound **3u**

Rac-**3v**

SAMPLE INFORMATION

Sample Name:	zjy-5-200-10%-IC-RAC	Acquired By:	System
Sample Type:	Unknown	Sample Set Name:	0
Vial:	54	Acq. Method Set:	10%quanbo
Injection #:	1	Processing Method:	LC PQ
Injection Volume:	10.00 ul	Channel Name:	220.0nm
Run Time:	30.0 Minutes	Proc. Chnl. Descr.:	2998 PDA 220.0 nm (2998
Date Acquired:	7/30/2021 12:00:23 PM CST		
Date Processed:	8/9/2021 6:22:10 PM CST		

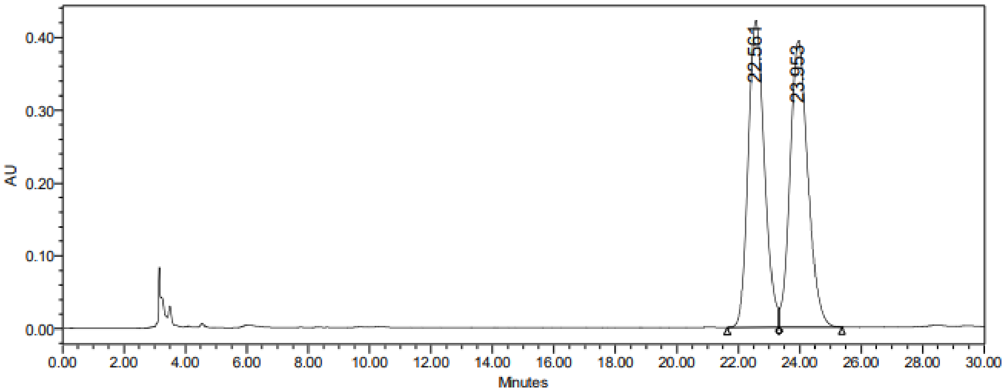

	RT	Area	% Area	Height
1	22.561	15461647	49.37	420652
2	23.953	15856307	50.63	393264

Asy-**3v**

SAMPLE INFORMATION

Sample Name:	zjy-5-200-10%-IC-asy	Acquired By:	System
Sample Type:	Unknown	Sample Set Name:	0
Vial:	55	Acq. Method Set:	10%quanbo
Injection #:	1	Processing Method:	ZJY 5 200 1
Injection Volume:	10.00 ul	Channel Name:	220.0nm
Run Time:	30.0 Minutes	Proc. Chnl. Descr.:	2998 PDA 220.0 nm (2998
Date Acquired:	7/30/2021 12:31:04 PM CST		
Date Processed:	7/30/2021 3:50:46 PM CST		

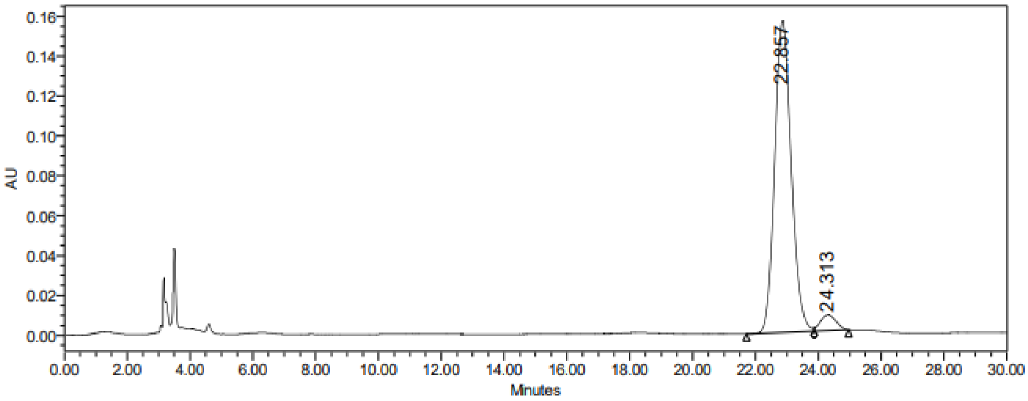

	RT	Area	% Area	Height
1	22.857	5769426	95.40	156077
2	24.313	277951	4.60	7951

Supplementary Figure 88. HPLC spectra of compound **3v**

Rac-**3w**

SAMPLE INFORMATION

Sample Name:	zjy-5-227-30%-IG-rac	Acquired By:	System
Sample Type:	Unknown	Sample Set Name:	0
Vial:	62	Acq. Method Set:	30%quanbo
Injection #:	1	Processing Method:	ZJY5 227 0
Injection Volume:	5.00 ul	Channel Name:	270.0nm
Run Time:	65.0 Minutes	Proc. Chnl. Descr.:	2998 PDA 270.0 nm (2998
Date Acquired:	8/6/2021 10:31:45 AM CST		
Date Processed:	8/6/2021 11:38:06 AM CST		

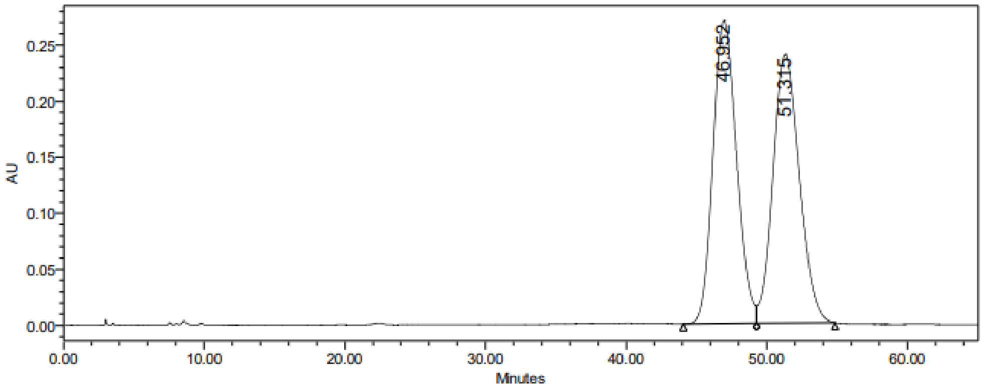

	RT	Area	% Area	Height
1	46.952	31266933	49.96	270278
2	51.315	31318240	50.04	239716

Asy-**3w**

SAMPLE INFORMATION

Sample Name:	zjy-5-227-30%-IG-asy	Acquired By:	System
Sample Type:	Unknown	Sample Set Name:	0
Vial:	61	Acq. Method Set:	30%quanbo
Injection #:	1	Processing Method:	ZJY5 227
Injection Volume:	5.00 ul	Channel Name:	270.0nm
Run Time:	65.0 Minutes	Proc. Chnl. Descr.:	2998 PDA 270.0 nm (2998
Date Acquired:	8/6/2021 9:20:44 AM CST		
Date Processed:	8/6/2021 11:33:59 AM CST		

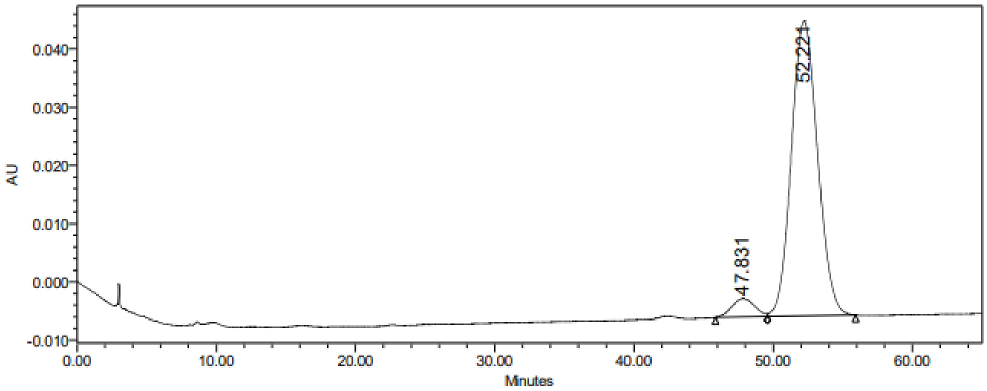

	RT	Area	% Area	Height
1	47.831	360735	5.16	3196
2	52.221	6628018	94.84	50655

Supplementary Figure 89. HPLC spectra of compound **3w**

Rac-**3x**

SAMPLE INFORMATION

Sample Name:	zjy-5-186-20%-IC-RAC	Acquired By:	System
Sample Type:	Unknown	Sample Set Name:	0
Vial:	67	Acq. Method Set:	20%quanbo
Injection #:	1	Processing Method:	ZJY 5 186 1
Injection Volume:	10.00 ul	Channel Name:	220.0nm
Run Time:	35.0 Minutes	Proc. Chnl. Descr.:	2998 PDA 220.0 nm (2998
Date Acquired:	7/26/2021 12:33:29 PM CST		
Date Processed:	8/29/2021 3:14:36 PM CST		

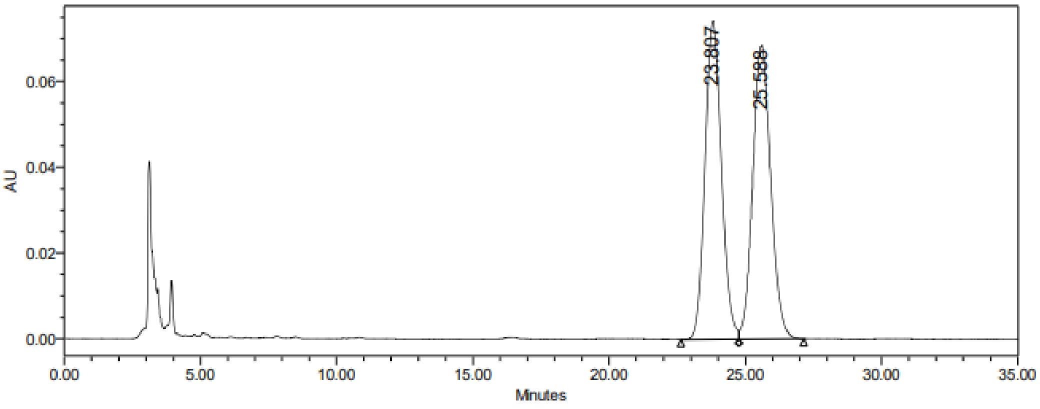

	RT	Area	% Area	Height
1	23.807	3123539	50.05	74102
2	25.588	3117851	49.95	68447

Asy-**3x**

SAMPLE INFORMATION

Sample Name:	zjy-5-186-20%-IC-asy	Acquired By:	System
Sample Type:	Unknown	Sample Set Name:	0
Vial:	66	Acq. Method Set:	20%quanbo
Injection #:	1	Processing Method:	ZJY 5 186
Injection Volume:	10.00 ul	Channel Name:	220.0nm
Run Time:	35.0 Minutes	Proc. Chnl. Descr.:	2998 PDA 220.0 nm (2998
Date Acquired:	7/26/2021 11:57:48 AM CST		
Date Processed:	8/29/2021 3:10:41 PM CST		

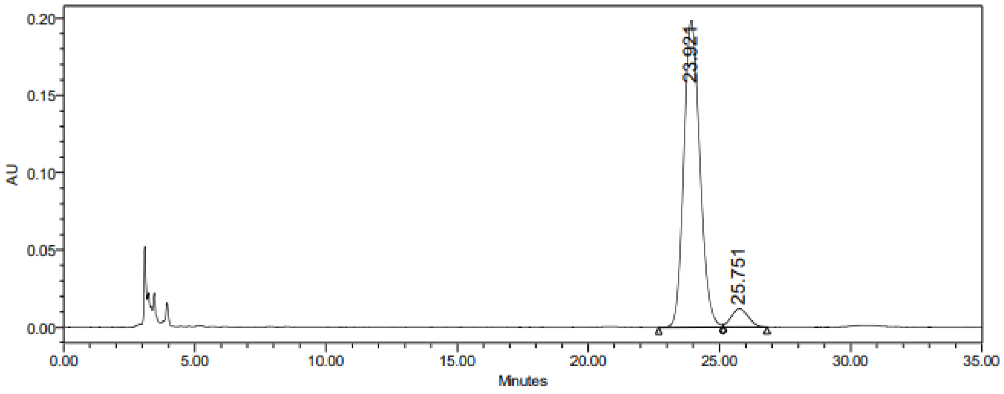

	RT	Area	% Area	Height
1	23.921	8447244	94.01	198031
2	25.751	537997	5.99	11902

Supplementary Figure 90. HPLC spectra of compound **3x**

Rac-**3y**

SAMPLE INFORMATION

Sample Name:	zjy-5-223-30%-IE-RAC	Acquired By:	System
Sample Type:	Unknown	Sample Set Name:	0
Vial:	51	Acq. Method Set:	30%quanbo
Injection #:	1	Processing Method:	ZJY5
Injection Volume:	10.00 ul	Channel Name:	241.0nm
Run Time:	55.0 Minutes	Proc. Chnl. Descr.:	2998 PDA 241.0 nm (2998
Date Acquired:	8/6/2021 3:42:55 PM CST		
Date Processed:	8/9/2021 6:19:22 PM CST		

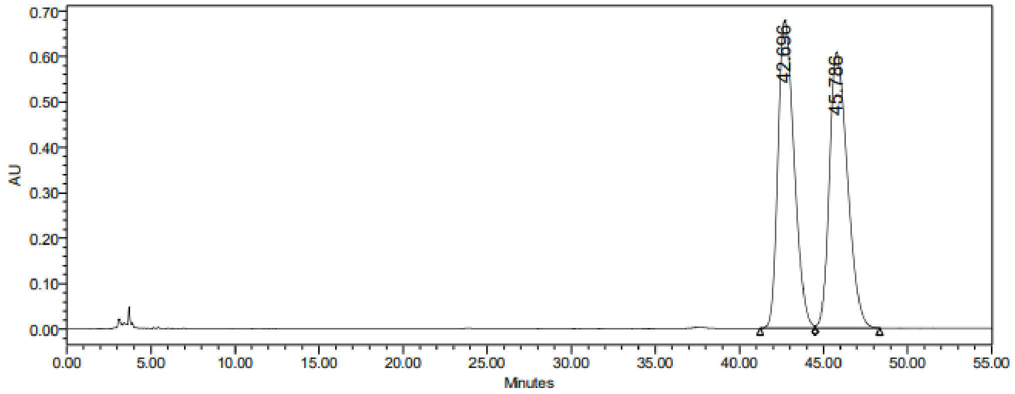

	RT	Area	% Area	Height
1	42.696	45328326	49.94	676820
2	45.786	45443063	50.06	606773

Asy-**3y**

SAMPLE INFORMATION

Sample Name:	zjy-5-223-30%-IE-asy	Acquired By:	System
Sample Type:	Unknown	Sample Set Name:	0
Vial:	50	Acq. Method Set:	30%quanbo
Injection #:	1	Processing Method:	ZJY5 223 ASY1
Injection Volume:	10.00 ul	Channel Name:	241.0nm
Run Time:	55.0 Minutes	Proc. Chnl. Descr.:	2998 PDA 241.0 nm (2998
Date Acquired:	8/6/2021 2:47:14 PM CST		
Date Processed:	8/9/2021 6:16:52 PM CST		

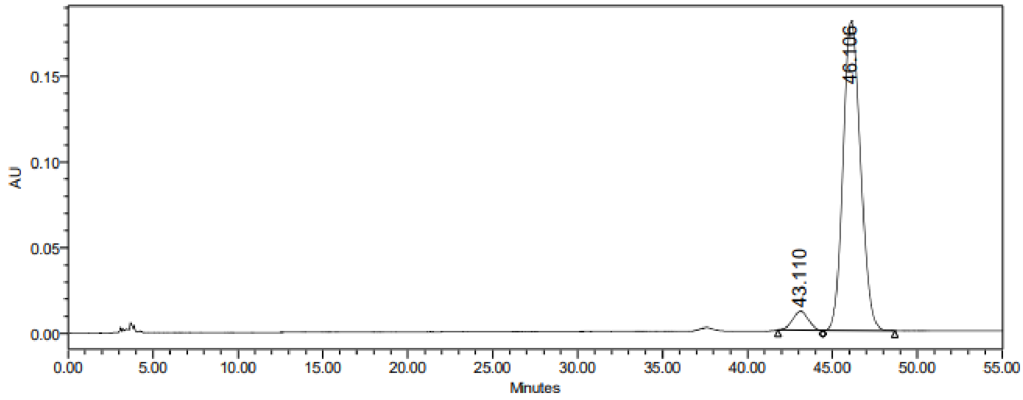

	RT	Area	% Area	Height
1	43.110	710291	5.19	10948
2	46.106	12964854	94.81	180201

Supplementary Figure 91. HPLC spectra of compound **3y**

Rac-**3z**

SAMPLE INFORMATION

Sample Name:	zjy-5-207-10%-IC-RAC	Acquired By:	System
Sample Type:	Unknown	Sample Set Name:	0
Vial:	51	Acq. Method Set:	10%quanbo
Injection #:	1	Processing Method:	ZJY 5 207 RAC
Injection Volume:	10.00 ul	Channel Name:	220.0nm
Run Time:	35.0 Minutes	Proc. Chnl. Descr.:	2998 PDA 220.0 nm (2998
Date Acquired:	7/30/2021 7:21:23 PM CST		
Date Processed:	8/29/2021 3:53:05 PM CST		

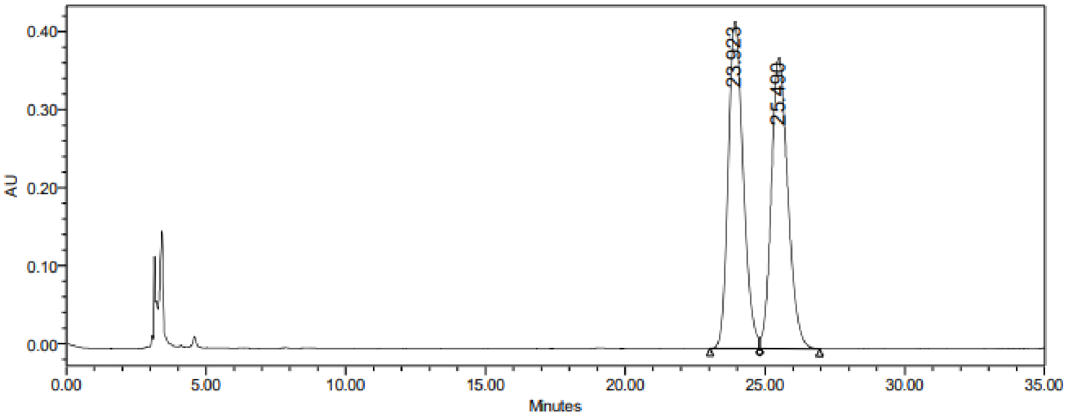

	RT	Area	% Area	Height
1	23.923	15718933	50.86	418491
2	25.490	15188208	49.14	372465

Asy-**3z**

SAMPLE INFORMATION

Sample Name:	zjy-5-207-10%-IC-asy	Acquired By:	System
Sample Type:	Unknown	Sample Set Name:	0
Vial:	50	Acq. Method Set:	10%quanbo
Injection #:	1	Processing Method:	ZJY 5 207 asy
Injection Volume:	10.00 ul	Channel Name:	220.0nm
Run Time:	35.0 Minutes	Proc. Chnl. Descr.:	2998 PDA 220.0 nm (2998
Date Acquired:	7/30/2021 6:45:40 PM CST		
Date Processed:	8/29/2021 3:54:25 PM CST		

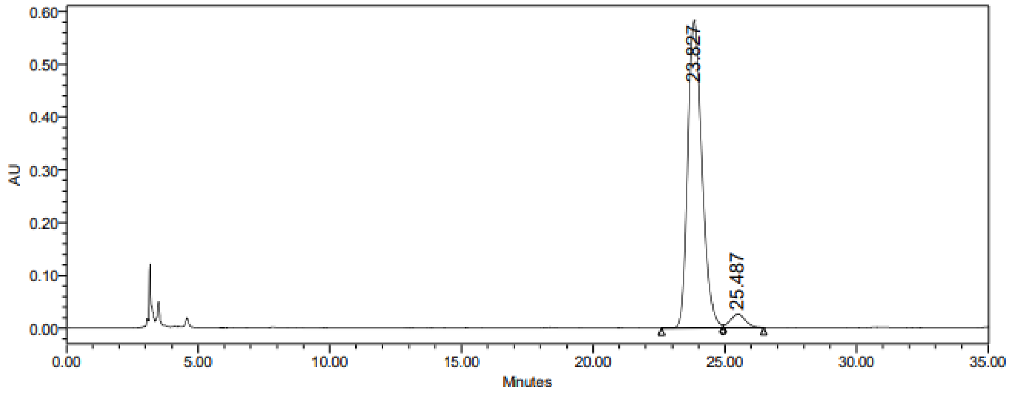

	RT	Area	% Area	Height
1	23.827	21808698	95.37	581876
2	25.487	1059097	4.63	26447

Supplementary Figure 92. HPLC spectra of compound **3z**

Rac-**3aa**

SAMPLE INFORMATION

Sample Name:	zjy-5-229-10%-AD-RAc	Acquired By:	System
Sample Type:	Unknown	Sample Set Name:	0
Vial:	56	Acq. Method Set:	10%quanbo
Injection #:	1	Processing Method:	0
Injection Volume:	10.00 ul	Channel Name:	220.0nm
Run Time:	10.0 Minutes	Proc. Chnl. Descr.:	2998 PDA 220.0 nm (2998
Date Acquired:	8/30/2021 12:44:57 AM CST		
Date Processed:	8/30/2021 9:38:15 PM CST		

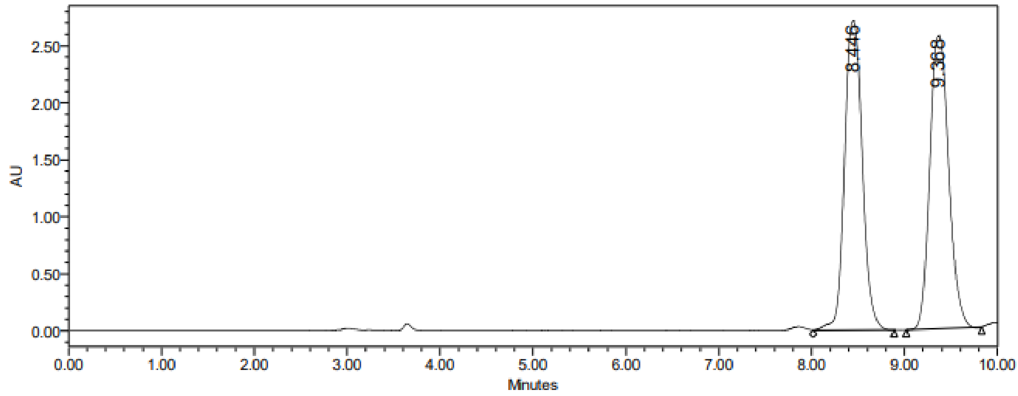

	RT	Area	% Area	Height
1	8.446	36246410	49.92	2712079
2	9.368	36368958	50.08	2568243

Asy-**3aa**

SAMPLE INFORMATION

Sample Name:	zjy-5-229-10%-AD-asy	Acquired By:	System
Sample Type:	Unknown	Sample Set Name:	0
Vial:	64	Acq. Method Set:	10%quanbo
Injection #:	1	Processing Method:	1354685746
Injection Volume:	10.00 ul	Channel Name:	220.0nm
Run Time:	10.0 Minutes	Proc. Chnl. Descr.:	2998 PDA 220.0 nm (2998
Date Acquired:	8/31/2021 11:11:45 AM CST		
Date Processed:	9/8/2021 3:49:46 PM CST		

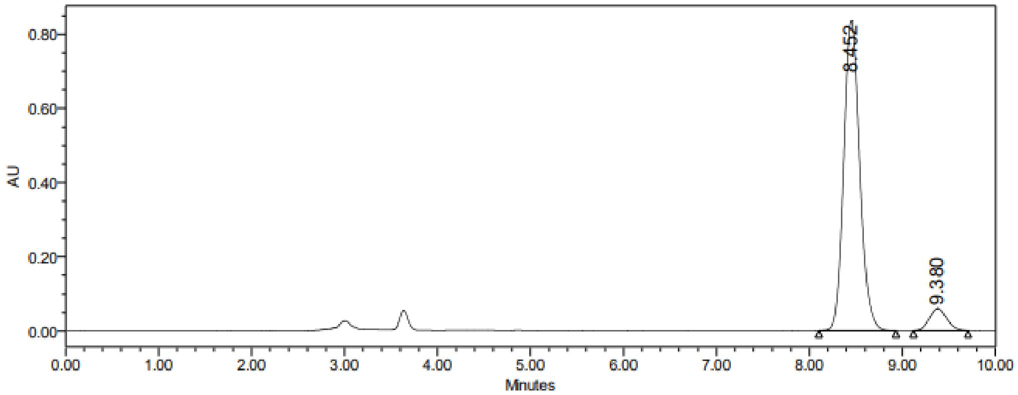

	RT	Area	% Area	Height
1	8.452	10042592	92.84	835138
2	9.380	774562	7.16	59260

Supplementary Figure 93. HPLC spectra of compound **3aa**

Rac-**3ab**

SAMPLE INFORMATION

Sample Name:	zjy-5-216-30%-IE-RAC	Acquired By:	System
Sample Type:	Unknown	Sample Set Name:	0
Vial:	57	Acq. Method Set:	30%quanbo
Injection #:	1	Processing Method:	LC PQ
Injection Volume:	5.00 ul	Channel Name:	220.0nm
Run Time:	40.0 Minutes	Proc. Chnl. Descr.:	2998 PDA 220.0 nm (2998
Date Acquired:	8/1/2021 11:51:11 PM CST		
Date Processed:	8/9/2021 6:36:34 PM CST		

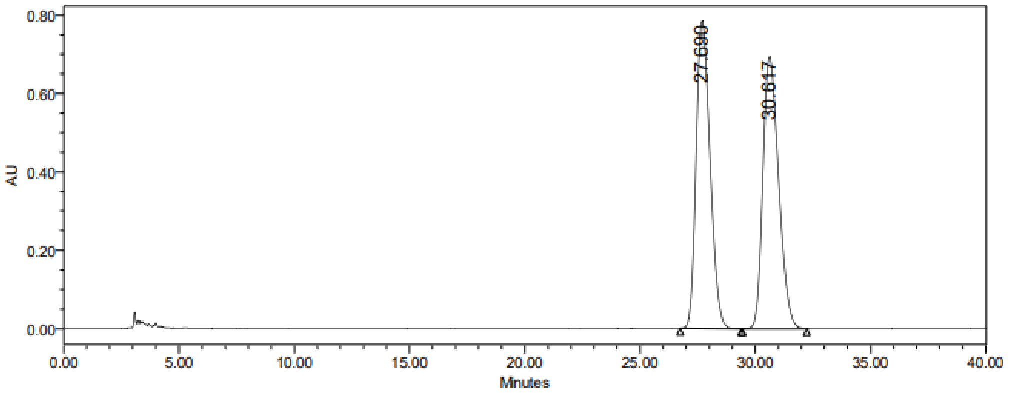

	RT	Area	% Area	Height
1	27.690	33030669	49.98	784288
2	30.617	33061543	50.02	693623

Asy-**3ab**

SAMPLE INFORMATION

Sample Name:	zjy-5-216-30%-IE-asy	Acquired By:	System
Sample Type:	Unknown	Sample Set Name:	0
Vial:	60	Acq. Method Set:	30%quanbo
Injection #:	1	Processing Method:	LC PQ
Injection Volume:	10.00 ul	Channel Name:	220.0nm
Run Time:	40.0 Minutes	Proc. Chnl. Descr.:	2998 PDA 220.0 nm (2998
Date Acquired:	8/2/2021 9:03:18 AM CST		
Date Processed:	8/9/2021 6:35:28 PM CST		

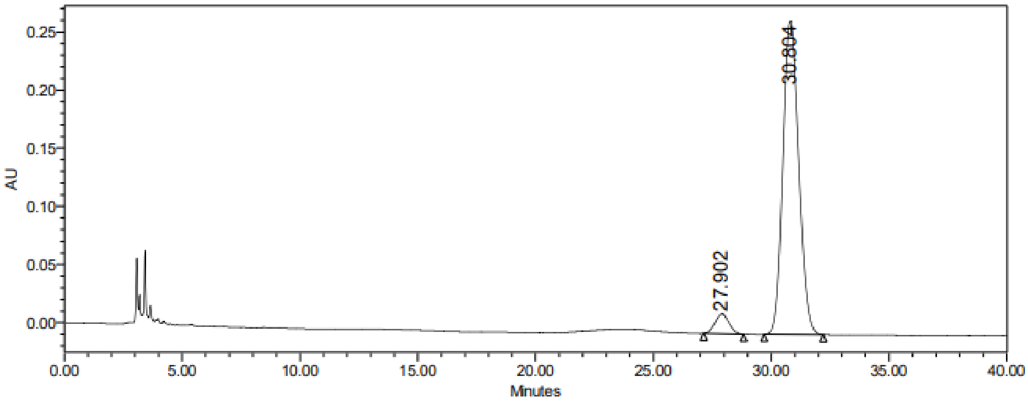

	RT	Area	% Area	Height
1	27.902	689129	5.16	16855
2	30.804	12663267	94.84	269236

Supplementary Figure 94. HPLC spectra of compound **3ab**

Rac-**3ac**

SAMPLE INFORMATION

Sample Name:	zjy-5-228-1%-OD-RAC	Acquired By:	System
Sample Type:	Unknown	Sample Set Name:	0
Vial:	50	Acq. Method Set:	1%quanbo
Injection #:	1	Processing Method:	54165463
Injection Volume:	10.00 ul	Channel Name:	231.1nm
Run Time:	30.0 Minutes	Proc. Chnl. Descr.:	2998 PDA 231.1 nm (2998
Date Acquired:	8/8/2021 6:10:58 PM CST		
Date Processed:	9/16/2021 9:19:24 AM CST		

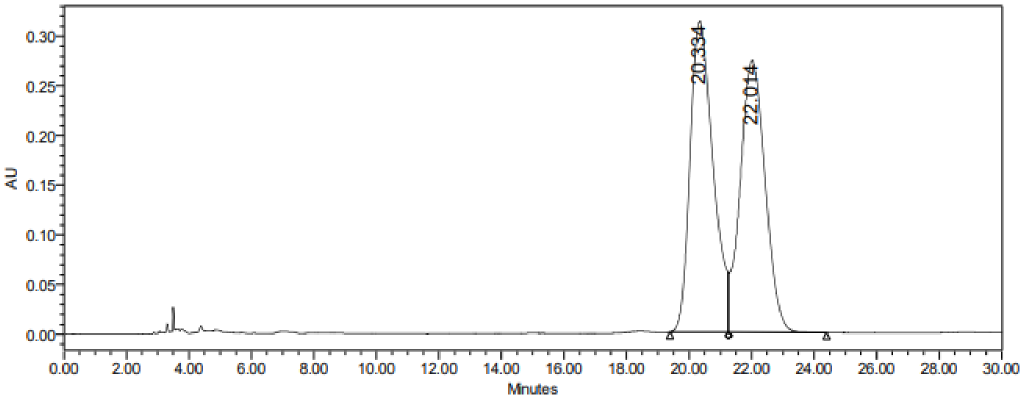

	RT	Area	% Area	Height
1	20.334	16085464	51.44	312547
2	22.014	15186405	48.56	273308

Asy-**3ac**

SAMPLE INFORMATION

Sample Name:	zjy-5-228-1%-OD-asy	Acquired By:	System
Sample Type:	Unknown	Sample Set Name:	
Vial:	51	Acq. Method Set:	1%quanbo
Injection #:	1	Processing Method:	54165463
Injection Volume:	5.00 ul	Channel Name:	220.0nm
Run Time:	100.0 Minutes	Proc. Chnl. Descr.:	2998 PDA 220.0 nm (2998
Date Acquired:	8/7/2021 9:48:27 AM CST		
Date Processed:	9/13/2021 12:51:04 PM CST		

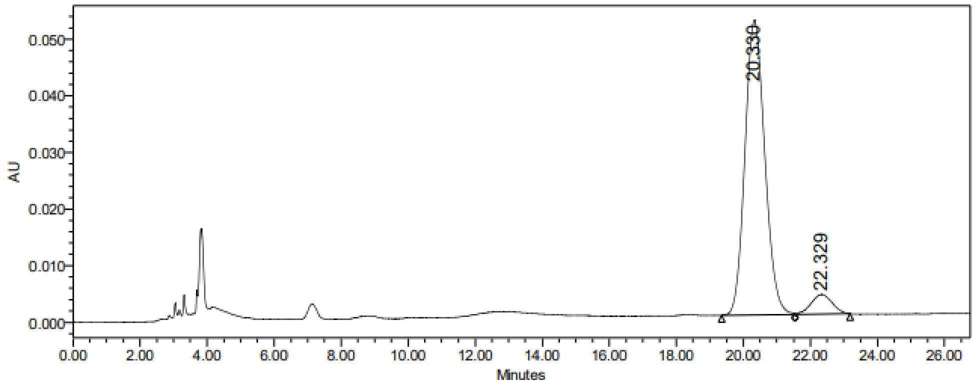

	RT	Area	% Area	Height
1	20.330	2140524	93.30	51887
2	22.329	153656	6.70	3482

Supplementary Figure 95. HPLC spectra of compound **3ac**

Rac-**5**

SAMPLE INFORMATION

Sample Name:	zjy-6-200-30%-IC-asy	Acquired By:	System
Sample Type:	Unknown	Sample Set Name:	0
Vial:	50	Acq. Method Set:	30%quanbo
Injection #:	1	Processing Method:	Default
Injection Volume:	10.00 ul	Channel Name:	230.0nm
Run Time:	22.0 Minutes	Proc. Chnl. Descr.:	2998 PDA 230.0 nm (2998
Date Acquired:	10/30/2021 10:46:59 AM CST		
Date Processed:	10/30/2021 11:22:18 AM CST		

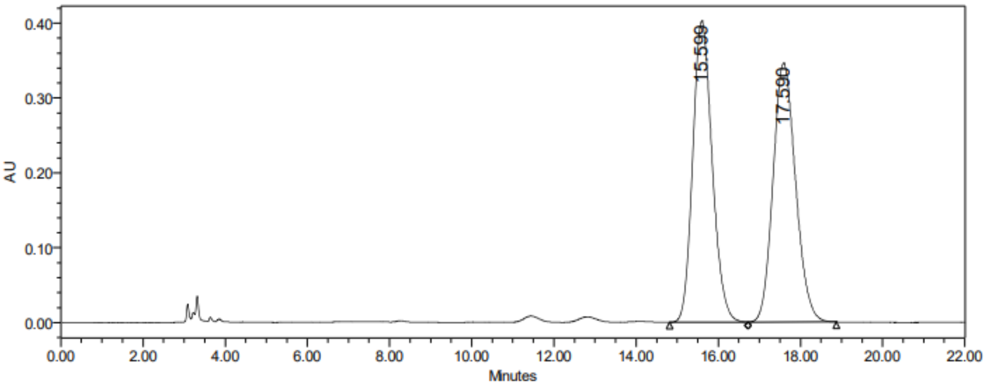

	RT	Area	% Area	Height
1	15.599	13190251	50.07	402266
2	17.590	13155539	49.93	345832

Asy-**5**

SAMPLE INFORMATION

Sample Name:	zjy-6-200-30%-IC-asy	Acquired By:	System
Sample Type:	Unknown	Sample Set Name:	0
Vial:	49	Acq. Method Set:	30%quanbo
Injection #:	1	Processing Method:	54165463
Injection Volume:	10.00 ul	Channel Name:	230.0nm
Run Time:	22.0 Minutes	Proc. Chnl. Descr.:	2998 PDA 230.0 nm (2998
Date Acquired:	10/30/2021 10:24:05 AM CST		
Date Processed:	10/30/2021 11:21:27 AM CST		

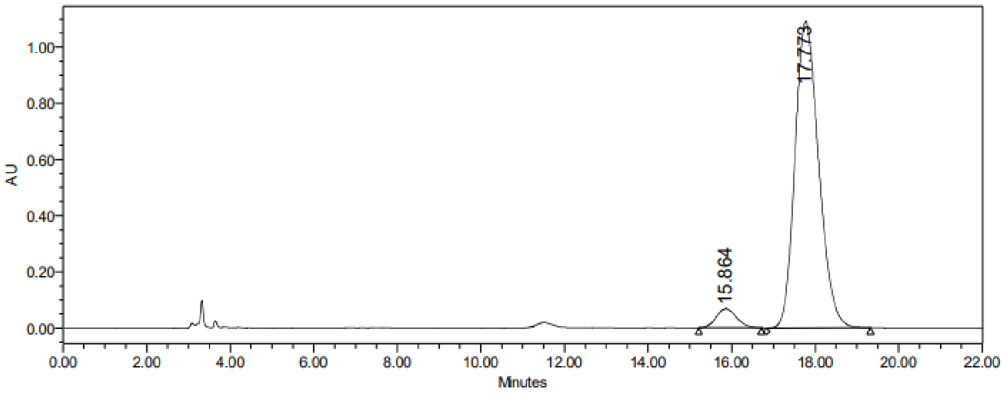

	RT	Area	% Area	Height
1	15.864	2311342	5.11	68390
2	17.773	42899990	94.89	1090049

Supplementary Figure 96. HPLC spectra of compound **5**

Rac-**6**

SAMPLE INFORMATION

Sample Name:	zjy-7-322-20%-IC-RAC	Acquired By:	System
Sample Type:	Unknown	Sample Set Name:	0
Vial:	68	Acq. Method Set:	20%quanbo
Injection #:	1	Processing Method:	132
Injection Volume:	10.00 ul	Channel Name:	220.0nm
Run Time:	35.0 Minutes	Proc. Chnl. Descr.:	2998 PDA 220.0 nm (2998
Date Acquired:	4/15/2022 10:02:26 PM CST		
Date Processed:	6/23/2022 11:37:21 PM CST		

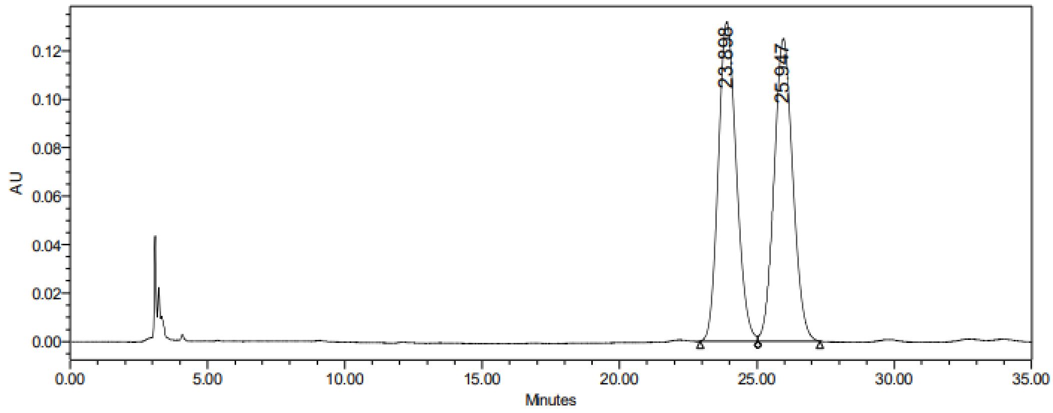

	RT	Area	% Area	Height
1	23.898	6011912	50.01	131553
2	25.947	6009740	49.99	124732

asy-**6**

SAMPLE INFORMATION

Sample Name:	zjy-7-322-large-20%-IC-asy	Acquired By:	System
Sample Type:	Unknown	Sample Set Name:	
Vial:	65	Acq. Method Set:	20%quanbo
Injection #:	1	Processing Method:	0
Injection Volume:	5.00 ul	Channel Name:	220.0nm
Run Time:	100.0 Minutes	Proc. Chnl. Descr.:	2998 PDA 220.0 nm (2998
Date Acquired:	4/25/2022 10:18:36 AM CST		
Date Processed:	6/23/2022 11:46:05 PM CST		

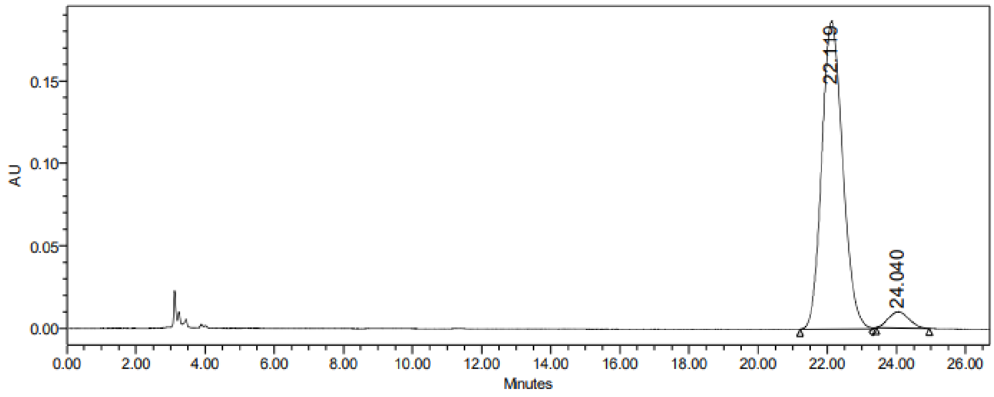

	RT	Area	% Area	Height
1	22.119	7823710	95.16	186318
2	24.040	398170	4.84	9756

Supplementary Figure 97. HPLC spectra of compound **6**

Rac-**7**

SAMPLE INFORMATION

Sample Name:	zjy-8-19-30%-AD-RAC	Acquired By:	System
Sample Type:	Unknown	Sample Set Name:	
Vial:	70	Acq. Method Set:	30% quanbo
Injection #:	1	Processing Method	44564
Injection Volume:	5.00 ul	Channel Name:	220.0nm
Run Time:	80.0 Minutes	Proc. Chnl. Descr.:	2998 PDA 220.0 nm (2998
Date Acquired:	4/25/2022 3:50:44 PM CST		
Date Processed:	6/23/2022 11:52:42 PM CST		

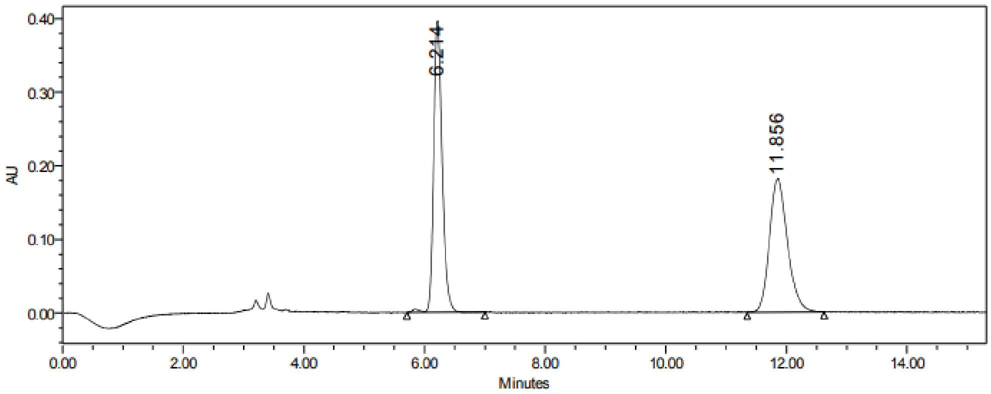

	RT	Area	% Area	Height
1	6.214	3877900	50.66	394506
2	11.856	3776787	49.34	180949

asy-**7**

SAMPLE INFORMATION

Sample Name:	zjy-8-18-30%-AD-asy	Acquired By:	System
Sample Type:	Unknown	Sample Set Name:	
Vial:	71	Acq. Method Set:	30% quanbo
Injection #:	1	Processing Method	41654
Injection Volume:	5.00 ul	Channel Name:	220.0nm
Run Time:	80.0 Minutes	Proc. Chnl. Descr.:	2998 PDA 220.0 nm (2998
Date Acquired:	4/25/2022 4:07:06 PM CST		
Date Processed:	6/23/2022 11:53:56 PM CST		

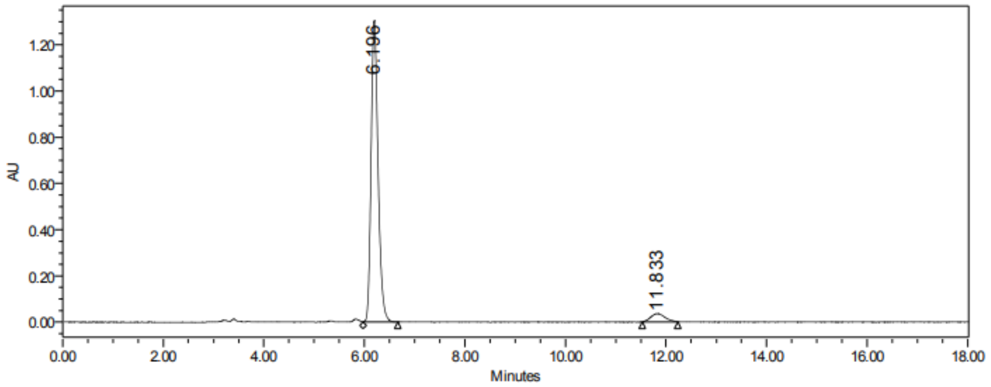

	RT	Area	% Area	Height
1	6.196	12666297	95.00	1303009
2	11.833	667038	5.00	34835

Supplementary Figure 98. HPLC spectra of compound **7**

Rac-**8**

SAMPLE INFORMATION

Sample Name:	zjy-8-47-10%-OD-RAC	Acquired By:	System
Sample Type:	Unknown	Sample Set Name:	
Vial:	66	Acq. Method Set:	10%quanbo
Injection #:	1	Processing Method:	0
Injection Volume:	5.00 ul	Channel Name:	220.0nm
Run Time:	100.0 Minutes	Proc. Chnl. Descr.:	2998 PDA 220.0 nm (2998
Date Acquired:	5/12/2022 12:36:17 PM CST		
Date Processed:	6/23/2022 11:52:20 PM CST		

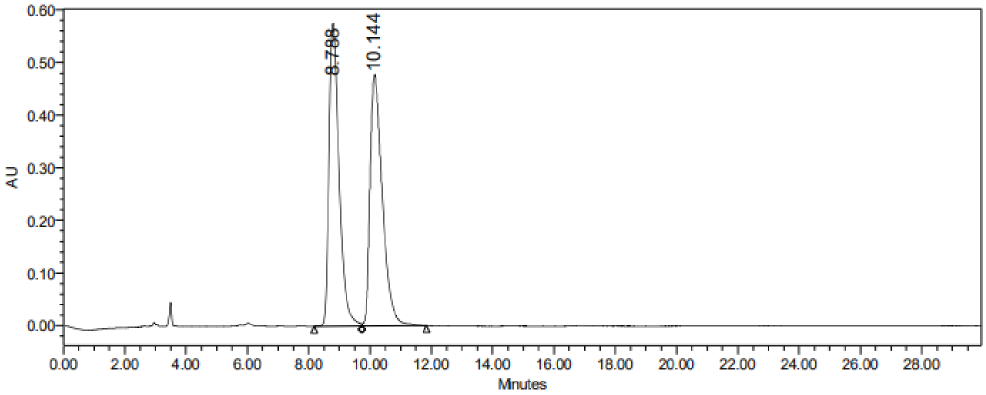

	RT	Area	% Area	Height
1	8.788	13175434	49.93	574377
2	10.144	13214108	50.07	477363

asy-**8**

SAMPLE INFORMATION

Sample Name:	zjy-8-49-10%-OD-asy	Acquired By:	System
Sample Type:	Unknown	Sample Set Name:	0
Vial:	67	Acq. Method Set:	10%quanbo
Injection #:	1	Processing Method:	0
Injection Volume:	5.00 ul	Channel Name:	220.0nm
Run Time:	15.0 Minutes	Proc. Chnl. Descr.:	2998 PDA 220.0 nm (2998
Date Acquired:	5/12/2022 12:08:31 PM CST		
Date Processed:	6/23/2022 11:54:08 PM CST		

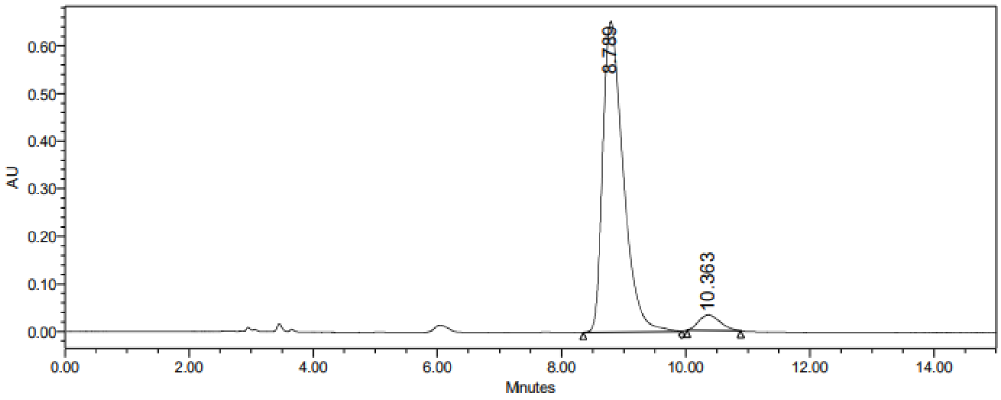

	RT	Area	% Area	Height
1	8.789	14947745	95.00	652117
2	10.363	786775	5.00	32384

Supplementary Figure 99. HPLC spectra of compound **8**

Rac-**9**

SAMPLE INFORMATION

Sample Name:	zjy-8-17-5%-AD-RAC	Acquired By:	System
Sample Type:	Unknown	Sample Set Name:	
Vial:	72	Acq. Method Set:	5% quanbo
Injection #:	1	Processing Method	44564
Injection Volume:	5.00 ul	Channel Name:	220.0nm
Run Time:	80.0 Minutes	Proc. Chnl. Descr.:	2998 PDA 220.0 nm (2998
Date Acquired:	4/25/2022 4:32:17 PM CST		
Date Processed:	6/24/2022 12:00:55 AM CST		

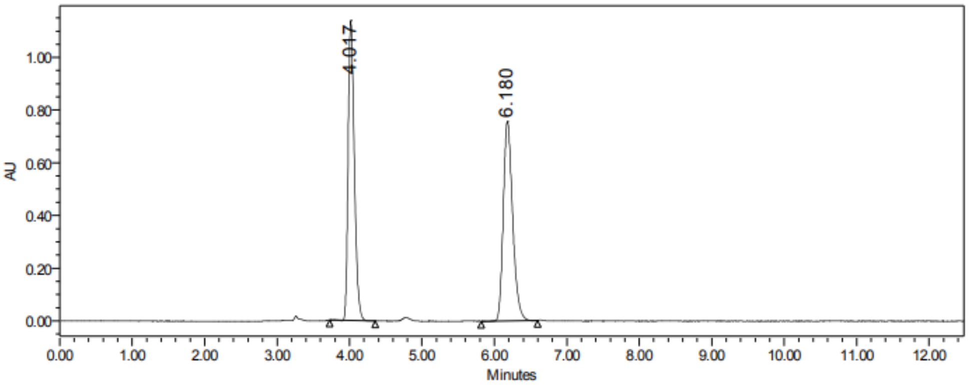

	RT	Area	% Area	Height
1	4.017	6707483	50.05	1137624
2	6.180	6693420	49.95	756559

asy-**9**

SAMPLE INFORMATION

Sample Name:	zjy-8-20-5%-AD-asy	Acquired By:	System
Sample Type:	Unknown	Sample Set Name:	
Vial:	49	Acq. Method Set:	5% quanbo
Injection #:	1	Processing Method	41654
Injection Volume:	10.00 ul	Channel Name:	220.0nm
Run Time:	80.0 Minutes	Proc. Chnl. Descr.:	2998 PDA 220.0 nm (2998
Date Acquired:	4/25/2022 10:44:43 PM CST		
Date Processed:	6/23/2022 11:58:17 PM CST		

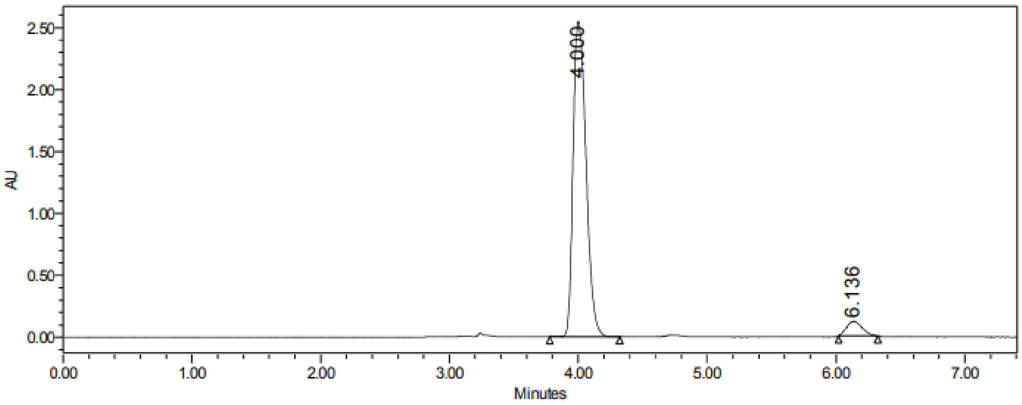

	RT	Area	% Area	Height
1	4.000	17983192	94.75	2542036
2	6.136	996625	5.25	119196

Supplementary Figure 100. HPLC spectra of compound **9**

Rac-**10**

SAMPLE INFORMATION

Sample Name:	zjy-8-28-5%-AD-RAC	Acquired By:	System
Sample Type:	Unknown	Sample Set Name:	
Vial:	67	Acq. Method Set:	5%quanbo
Injection #:	1	Processing Method:	0
Injection Volume:	5.00 ul	Channel Name:	220.0nm
Run Time:	100.0 Minutes	Proc. Chnl. Descr.:	2998 PDA 220.0 nm (2998
Date Acquired:	4/30/2022 11:06:21 AM CST		
Date Processed:	6/23/2022 11:51:11 PM CST		

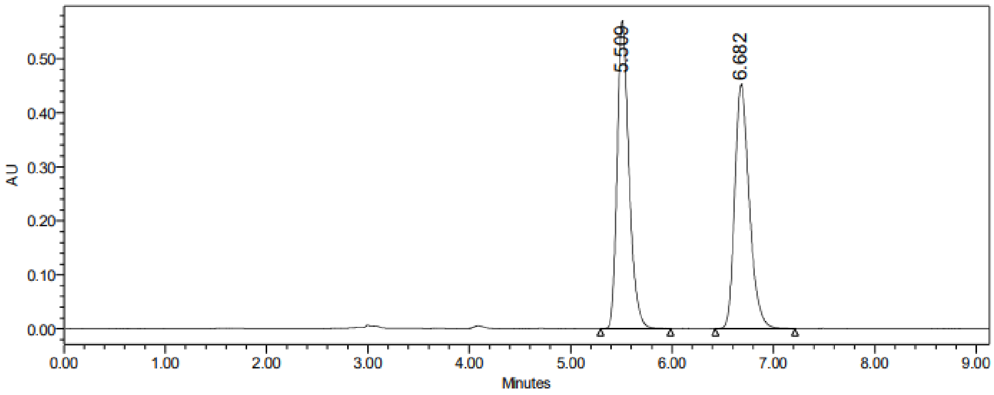

	RT	Area	% Area	Height
1	5.509	4626677	50.62	569270
2	6.682	4513794	49.38	452478

asy-**10**

SAMPLE INFORMATION

Sample Name:	zjy-8-28-5%-AD-asy	Acquired By:	System
Sample Type:	Unknown	Sample Set Name:	
Vial:	66	Acq. Method Set:	5%quanbo
Injection #:	1	Processing Method:	132
Injection Volume:	5.00 ul	Channel Name:	220.0nm
Run Time:	100.0 Minutes	Proc. Chnl. Descr.:	2998 PDA 220.0 nm (2998
Date Acquired:	4/30/2022 10:56:38 AM CST		
Date Processed:	6/23/2022 11:49:54 PM CST		

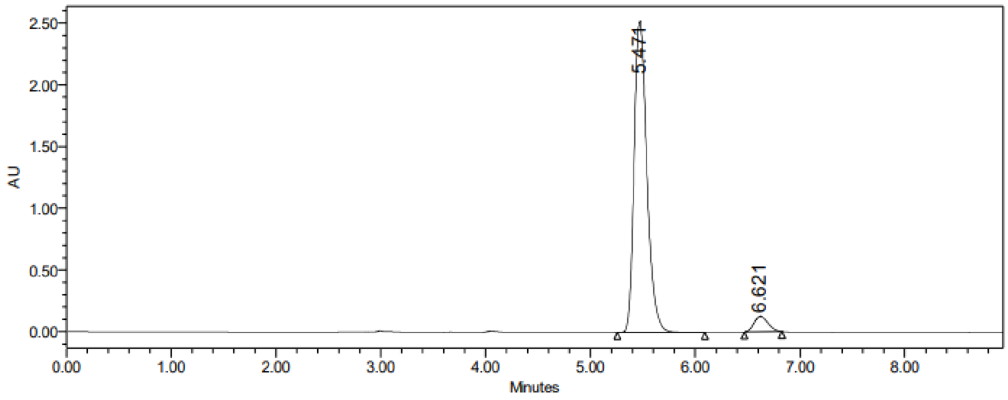

	RT	Area	% Area	Height
1	5.471	21148906	94.75	2518730
2	6.621	1171852	5.25	126917

Supplementary Figure 101. HPLC spectra of compound **10**

Rac-**11**

SAMPLE INFORMATION

Sample Name:	zjy-8-29-5%-AD-RAC	Acquired By:	System
Sample Type:	Unknown	Sample Set Name:	
Vial:	66	Acq. Method Set:	5% quanbo
Injection #:	1	Processing Method	41654
Injection Volume:	2.00 ul	Channel Name:	220.0nm
Run Time:	80.0 Minutes	Proc. Chnl. Descr.:	2998 PDA 220.0 nm (2998
Date Acquired:	4/30/2022 7:01:33 PM CST		
Date Processed:	6/24/2022 12:01:48 AM CST		

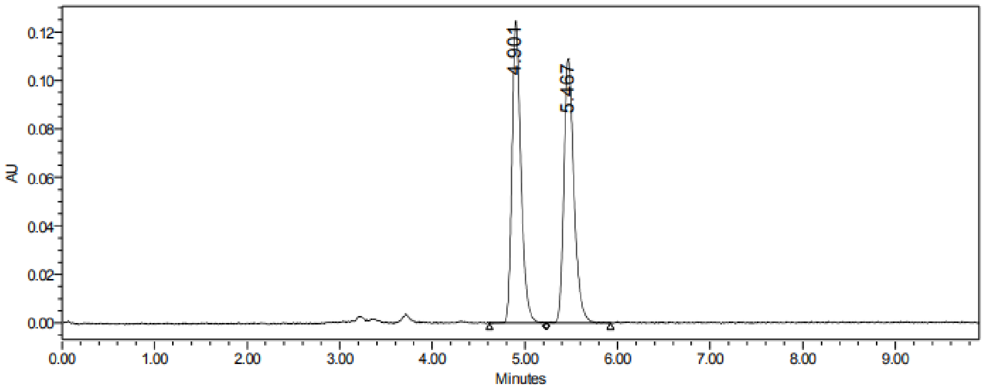

	RT	Area	% Area	Height
1	4.901	823460	50.07	124125
2	5.467	821002	49.93	108782

asy-**11**

SAMPLE INFORMATION

Sample Name:	zjy-8-29-5%-AD-asy	Acquired By:	System
Sample Type:	Unknown	Sample Set Name:	
Vial:	67	Acq. Method Set:	5% quanbo
Injection #:	2	Processing Method	41654
Injection Volume:	10.00 ul	Channel Name:	220.0nm
Run Time:	80.0 Minutes	Proc. Chnl. Descr.:	2998 PDA 220.0 nm (2998
Date Acquired:	4/30/2022 7:21:32 PM CST		
Date Processed:	6/24/2022 12:03:14 AM CST		

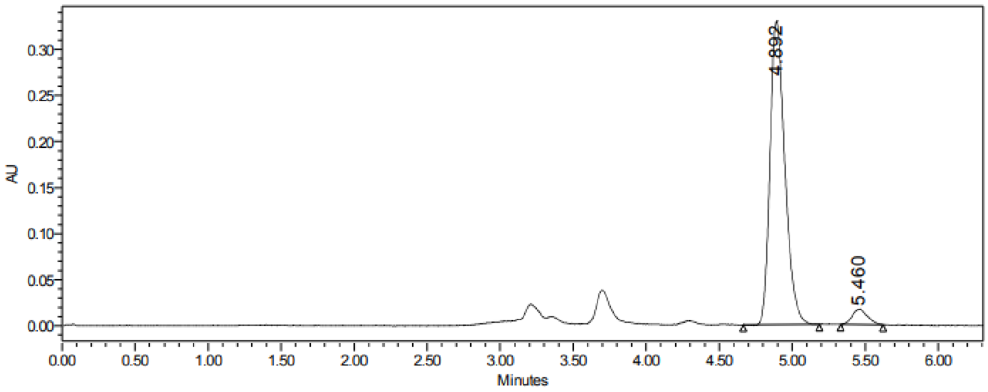

	RT	Area	% Area	Height
1	4.892	2335281	95.07	329236
2	5.460	121115	4.93	16403

Supplementary Figure 102. HPLC spectra of compound **11**

(Rac)-**1n′**

SAMPLE INFORMATION

Sample Name:	zjy-6-183(1nys)-5%-IC-RAC	Acquired By:	System
Sample Type:	Unknown	Sample Set Name:	
Vial:	65	Acq. Method Set:	5%quanbo
Injection #:	1	Processing Method:	54165463
Injection Volume:	3.00 ul	Channel Name:	258.5nm
Run Time:	100.0 Minutes	Proc. Chnl. Descr.:	2998 PDA 258.5 nm (2998
Date Acquired:	10/23/2021 1:06:20 PM CST		
Date Processed:	11/6/2021 9:47:38 AM CST		

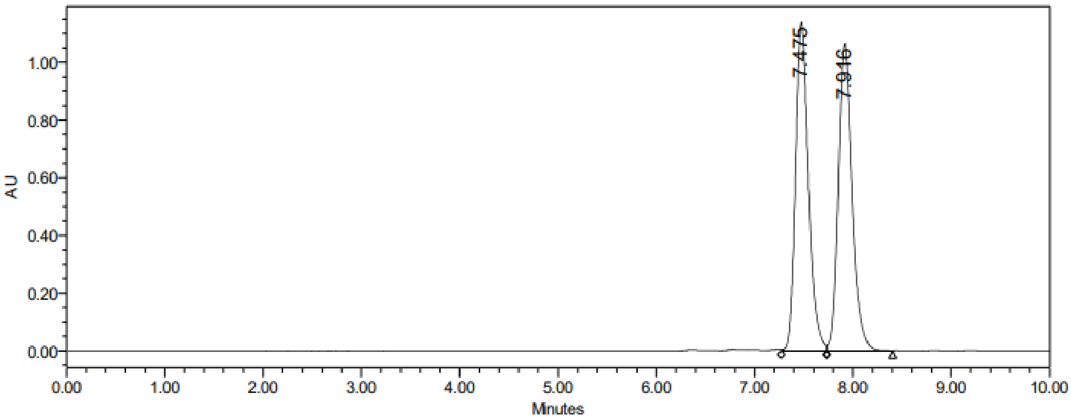

	RT	Area	% Area	Height
1	7.475	10356726	49.95	1137028
2	7.916	10376783	50.05	1062265

(*R*)-**1n′**

SAMPLE INFORMATION

Sample Name:	zjy-6-184-8h(re-R-1nys)-5%-IC	Acquired By:	System
Sample Type:	Unknown	Sample Set Name:	
Vial:	68	Acq. Method Set:	5%quanbo
Injection #:	1	Processing Method:	1354685746
Injection Volume:	10.00 ul	Channel Name:	254.0nm
Run Time:	100.0 Minutes	Proc. Chnl. Descr.:	2998 PDA 254.0 nm (2998
Date Acquired:	10/28/2021 2:38:26 PM CST		
Date Processed:	10/30/2021 10:02:28 AM CST		

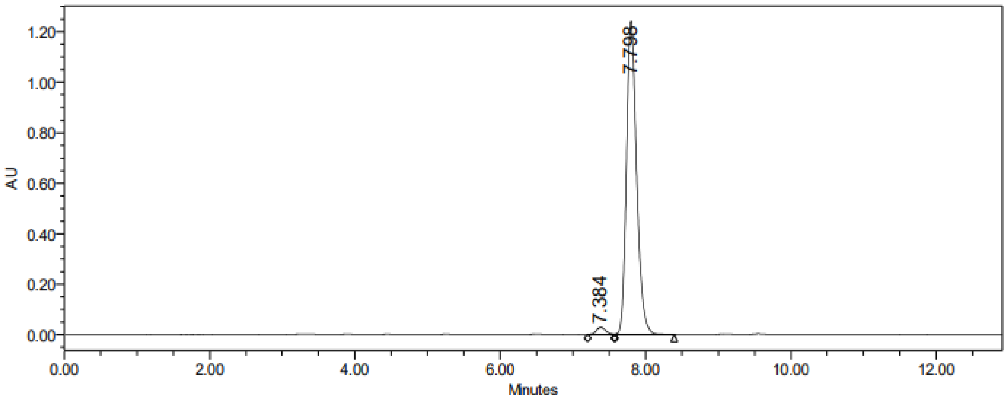

	RT	Area	% Area	Height
1	7.384	248182	2.00	27756
2	7.798	12140210	98.00	1240324

(*S*)-**1n′**

SAMPLE INFORMATION

Sample Name:	zjy-6-178(S-1nys)-5%-IC-asy	Acquired By:	System
Sample Type:	Unknown	Sample Set Name:	0
Vial:	66	Acq. Method Set:	5%quanbo
Injection #:	1	Processing Method:	54165463
Injection Volume:	10.00 ul	Channel Name:	254.0nm
Run Time:	10.0 Minutes	Proc. Chnl. Descr.:	2998 PDA 254.0 nm (2998
Date Acquired:	10/23/2021 9:04:14 PM CST		
Date Processed:	10/30/2021 9:59:28 AM CST		

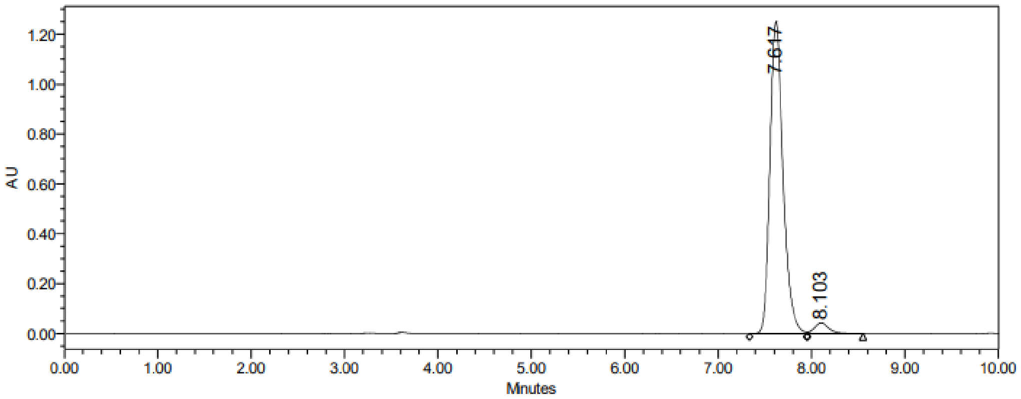

	RT	Area	% Area	Height
1	7.617	12227103	96.47	1250537
2	8.103	447440	3.53	41743

Supplementary Figure 103. HPLC spectra of compound **1n'**

4. Supplementary References

1. Kou, K. G. M., Longobardi, L. E. & Dong, V. M. Rhodium(I)-catalyzed intermolecular hydroacylation of α-keto amides and isatins with non-chelating aldehydes. *Adv. Synth. Catal.* **357**, 2233-2237 (2015).
2. Wang, Z. A., Kurra, Y., Wang, X., Zeng, Y., Lee, Y.-J., Sharma, V., Lin, H., Dai, S. Y. & Liu, W. R. A versatile approach for site-specific lysine acylation in proteins. *Angew. Chem. Int. Ed.* **56**, 1643-1647 (2017).
3. Matsumura, K., Hashiguchi, S., Ikariya, T. & Noyori, R. Asymmetric transfer hydrogenation of α, β-acetylenic ketones. *J. Am. Chem. Soc.* **119**, 8738-8739 (1997).
4. Wang, S. & Zhang, L. A highly efficient preparative method of α-ylidene-β-diketones via AuIII-catalyzed acyl migration of propargylic rsters. *J. Am. Chem. Soc.* **128**, 8414-8415 (2006).
5. He, G., Guo, H., Qian, R., Guo, Y., Fu, C. & Ma, S. Studies on highly regio- and stereoselective selenohydroxylation reaction of 1,2-allenyl phosphine oxides with PhSeCl. *Tetrahedron* **65**, 4877-4889 (2009).
6. Guo, H., Qian, R., Guo, Y. & Ma,S. Neighboring group participation of phosphine oxide functionality in the highly regio- and stereoselective iodohydroxylation of 1,2-allenylic diphenyl phosphine oxides. *J.Org.Chem.* **73**, 7934-7938 (2008).
7. Jiang, R., Ding, L., Zheng, C. &You, S.-L. Iridium-catalyzed *Z*-retentive asymmetric allylic substitution reactions. *Science* **371**, 380-386 (2021).
8. Qiu, L., Hu, W., Wu, D., Duan, Z. & Mathey, F. Regioselective synthesis of 2- or 2,7-functionalized pyrenes via migration. *Org. Lett.* **20**, 7821-7824 (2018)
9. Vanitcha, A., Damelincourt, C., Gontard, G., Vanthuyne, N., Mouriès-Mansuy, V. & Fensterbank, L. Bis-phosphine allene ligand: coordination chemistry and preliminary applications in catalysis. *Chem. Commun.* **52**,6785-6788 (2016).
10. Saito, H., Matsumoto, Y., Hashimoto, Y. & Fujii, S. Phosphine boranes as less hydrophobic building blocks than alkanes and silanes: structure-property relationship and estrogen-receptor-modulating potency of 4-phosphinophenol derivatives. *Bioorg. Med. Chem.* **28**, 115310-115316 (2020)
11. Boisselle, A. P. & Meinhardt, N. A. Acetylene-allene rearrangements reactions of trivalent phosphorus chlorides with α-acetylenic alcohols and glycols. *J. Org. Chem.* **27**, 1828-1833. (1962)
12. Guo, H., Qian, R., Guo, Y. & Ma, S. Neighboring group participation of phosphine oxide functionality in the highly regio- and stereoselective iodohydroxylation of 1,2-allenylic diphenyl phosphine oxides. *J. Org. Chem.* **73**, 7934-7938 (2008).
